# Proteogenomic Characterization of *Pseudomonas veronii* SM-20 Growing on Phenanthrene as Only Carbon and Energy Source

**DOI:** 10.3390/microorganisms12040753

**Published:** 2024-04-08

**Authors:** Sofía G. Zavala-Meneses, Andrea Firrincieli, Petra Chalova, Petr Pajer, Alice Checcucci, Ludovit Skultety, Martina Cappelletti

**Affiliations:** 1 Institute of Microbiology, Czech Academy of Sciences, Videnska 1083, 14220 Prague, Czech Republic; 2 Faculty of Science, Charles University, Vinicna 5, 12844 Prague, Czech Republic; 3 Department of Pharmacy and Biotechnology, University of Bologna, 40126 Bologna, Italy or andrea.firrincieli@unitus.it (A.F.); martina.cappelletti2@unibo.it (M.C.); 4 Biomedical Research Center, Slovak Academy of Sciences, Dubravska c. 9, 845 05 Bratislava, Slovakia; petra.chalova@savba.sk; 5 Faculty of Pharmacy, Comenius University, Odbojarov 10, 832 32 Bratislava, Slovakia; 6 Military Health Institute, Military Medical Agency, U Vojenske Nemocnice 1200, 16902 Prague, Czech Republic; petr.pajer@img.cas.cz; 7 Department of Agriculture, Food, Environment and Forestry, University of Florence, 50100 Firenze, Italy; alice.checcucci@unifi.it

**Keywords:** *Pseudomonas*, biodegradation, polycyclic aromatic hydrocarbons (PAHs), phenanthrene (PHE), proteomics, genomics

## Abstract

In this study, we conducted an extensive investigation of the biodegradation capabilities and stress response of the newly isolated strain *Pseudomonas veronii* SM-20 in order, to assess its potential for bioremediation of sites contaminated with polycyclic aromatic hydrocarbons (PAHs). Initially, phenotype microarray technology demonstrated the strain’s proficiency in utilizing various carbon sources and its resistance to certain stressors. Genomic analysis has identified numerous genes involved in aromatic hydrocarbon metabolism. Biodegradation assay analyzed the depletion of phenanthrene (PHE) when it was added as a sole carbon and energy source. We found that *P. veronii* strain SM-20 degraded approximately 25% of PHE over a 30-day period, starting with an initial concentration of 600 µg/mL, while being utilized for growth. The degradation process involved PHE oxidation to an unstable arene oxide and 9,10-phenanthrenequinone, followed by ring-cleavage. Comparative proteomics provided a comprehensive understanding of how the entire proteome responded to PHE exposure, revealing the strain’s adaptation in terms of aromatic metabolism, surface properties, and defense mechanism. In conclusion, our findings shed light on the promising attributes of *P. veronii* SM-20 and offer valuable insights for the use of *P. veronii* species in environmental restoration efforts targeting PAH-impacted sites.

## 1. Introduction

Intense industrialization in the 20th century has been particularly deleterious for the environment, resulting in many contaminated sites. Once these industrial areas have been abandoned, the contamination, dangerous for human and animal health or the environment, remains. PAHs, polychlorobiphenyls, and heavy metals are the most common pollutants in former industrial areas. These compounds are environmentally persistent and have various toxic, mutagenic, and/or carcinogenic properties, with the potential to enter the food chain, leading to biomagnification or bioaccumulation. PAHs are one of the most toxic and studied pollutants. In soil, they are highly recalcitrant, highly insoluble, and have low mobility. Their amount depends on aging or degradation processes affected by various factors, including temperature, pH, soil organic matter, and microbial contents. In recent years, such recovery sites have gained increasing attention in regard to preventing the migration of contaminants into groundwaters and developing strategies for environmental recovery [1]. 

Several chemical and physical technologies have been applied to restore PAH-contaminated soils. In the last few decades, particular attention was given to the most sustainable biological practices involving fungal, algal, and bacterial species for the degradation and mineralization of PAHs [2]. Microbial activities nowadays represent one of the primary processes by which PAHs are eliminated from the environment. The use of these microbial mechanisms offers a sustainable and ecological alternative to traditional remediation techniques. Utilizing the innate abilities of these microorganisms can not only be energy-saving, but also more cost-effective [3]. This ecological approach is in line with global efforts for greenness and sustainability in environmental management. Various species belonging to the genus *Pseudomonas*, *Burkholderia*, *Alcaligenes*, *Rhodococcus*, and *Ralstonia* are known to be involved in the degradation of PAHs [4] and have drawn attention as potential candidates for bioaugmentation [5]. *Pseudomonas* bacterial strains are ubiquitous Gram-negative aerobic chemoheterotrophs involved in different biogeochemical cycles including the nitrogen and carbon atom circulations [6]. They possess a wide range of features that confer an extensive biochemical and metabolic capacity to persist in different ecological niches and conditions [7]. *Pseudomonas* spp. strains are metabolically versatile and able to utilize various organic compounds as carbon sources [8,9]. As a result, *Pseudomonas* strains are often involved in the biodegradation and bioconversion processes of complex organic compounds including various classes of contaminants and xenobiotics. For instance, *P. putida* OR45a can degrade phenol [5], *P. aeruginosa* GOM1 metabolizes long-chain alkanes [10], while *P. veronii* 1YdBTEX2 is capable of utilizing toluene and benzene for growth [11]. Additionally, *P. veronii* strain 7–14 can degrade medium-chain n-alkanes (C_8_–C_12_) and naphthalene [12], and *P. veronii* UKR4 is tolerant/resistant to high concentrations of copper [13]. Due to these characteristics, *Pseudomonas* strains have various biotechnological applications. 

Despite the importance of *Pseudomonas* species in bioremediation, there is a scarcity of literature describing their growth on the model PAH, phenanthrene (PHE). This knowledge gap highlights the significance of our work in characterizing the newly isolated *P. veronii* strain SM-20, obtained from polluted soil sampled in Czech Republic. Genomic analysis allowed us to assess the genetic potential of the strain associated with aromatic metabolism and biodegradation of PAHs, while the metabolic characterization provided information on metabolic activities of the strain in the presence of different standard carbon sources and stressors (metals, pH, and antibiotics). We also evaluated the capacity of SM-20 to utilize PHE for growth and analyzed the dynamic changes in the proteomic profile of cells during exposure over time, leading to the elucidation of the biochemical pathway of PHE degradation by *P. veronii* SM-20. 

## 2. Materials and Methods

### 2.1. Chemicals

A comprehensive range of chemicals, including PHE, dithiothreitol (DTT), formic acid, [Glu1]-fibrinopeptide B, microbiological agar, and others essential for our procedures, were sourced primarily from Sigma-Aldrich (St. Louis, MO, USA) and Fluka (Buchs, Switzerland). Additional reagents, such as the complete mini-protease inhibitor cocktail, were procured from Roche (Mannheim, Germany), while LiChrosolv quality solvents were obtained from Merck (Darmstadt, Germany). Specific enzymes, including trypsin and benzonase, were supplied by Promega (Madison, WI, USA).

### 2.2. Microorganisms 

*Pseudomonas veronii* SM-20, provided by Dr. M. Brennerova from the Institute of Microbiology of the Czech Academy of Sciences, was originally isolated from the soil, heavily polluted with petroleum hydrocarbons near Staré Město, Moravia, Czech Republic (49°40′0″ N 18°22′12″ E) [14]. The strain was routinely cultured using a Luria–Bertani (LB) medium for the preparation of the pre-inoculum that was used in PHE biodegradation assays and for genomic DNA extraction. Solid Luria–Bertani (LB) medium with added microbiological agar (15 g/L) was used for bacterial enumeration, expressed as colony-forming units (CFU).

### 2.3. Genome Sequencing, Assembly, and Annotation

Bacterial cells grown overnight were used for DNA extraction by the Maxwell^®^ 16 Blood DNA Purification Kit and the Maxwell^®^ 16 instrument (Promega, Madison, WI, USA) following the manufacturer’s instructions with some modifications. Briefly, 3 mL of cells (2 × 10^9^ cells) was centrifuged at 4000× *g* for 10 min, resuspended in Tris-EDTA (TE) buffer (pH 7), and treated with 100 µL of lysozyme solution (25 mg/mL) at 37 °C for 1 h. Then, 20 µL of proteinase K solution (20 mg/mL) was added. After removing RNA with 5 μL of RNase A, DNA was quantified using the Qubit 2.0 fluorometer (Invitrogen, Carlsbad, CA, USA), and their quality and size were verified by gene electrophoresis [15].

Illumina MiSeq (Illumina, San Diego, CA, USA) and Oxford Nanopore Technologies (ONT, Oxford, UK) performed whole genome sequencing. For ONT sequencing, libraries were constructed using the Rapid Barcoding Kit (SQK-RBK004, Oxford Nanopore Technologies, Oxford, UK) following the manufacturer’s protocol. The ONT data were generated on the GridIONx5 platform with the R9.4.1 chemistry and base-called using ONT Guppy v. 3.2.10. Before Illumina sequencing, fragment size was verified using an Agilent Bioanalyzer 2100 (Agilent Technologies, Inc., Santa Clara, CA, USA), and samples were quantified by qPCR with the KAPA Library Quantification Kit for Illumina Platforms (Kapa Biosystems, Wilmington, MA, USA). Sequencing was carried out with 250 nt paired-end reads on the MiSeq platform by an external sequencing facility.

The genome was assembled by integrating data from both technologies to enhance the draft genome accuracy. For Illumina data, Unicycler 0.4.8, utilizing the SPAdes 3.13.1 assembler, processed forward and reverse reads [16]. Unicycler assembly mode balanced contig number and length. Reads were mapped to assembled contigs for further corrections. PhiX circular contig, an internal sequencing control, was excluded. Rapid Annotation using Subsystem Technology server version 2.0 (RAST; http://rast.theseed.org, accessed on 6 October 2021) performed functional annotation, gene calling, and gene mapping [17]. The genomic positions of each gene were obtained from the respective databases, and for visualization purposes, SnapGene was applied. It is important to note that genome annotation is inherently dependent on the identification of homology between newly discovered genes/proteins and previously annotated sequences [18].

Phylogenomic analysis employed the Type (Strain) Genome Server TYGS https://tygs.dsmz.de, accessed on 21 September 2022) [19]. The *P. veronii* SM-20 genome was compared to all type strain genomes in the TYGS database using the MASH algorithm. The 10 type strains with the smallest MASH distances were identified. The best matching type strains were then used to calculate precise phylogenetic distances using the Genome BLAST Distance Phylogeny approach (GBDP) under the algorithm ‘coverage’ and distance formula d5. The resulting tree was then visualized with iTOL.

### 2.4. Phenotype Microarray Assay

The phenotypic analysis of the *P. veronii* SM-20 strain was carried out using the fully automated Omnilog Phenotype MicroArray (PM) System (Biolog, Inc., Hayward, CA, USA) following the manufacturer’s instructions [20]. The strain was screened using Gen III MicroPlate (Biolog, Inc., Hayward, CA, USA), enabling the analysis of 94 metabolic tests, including the utilization of 71 carbon sources and 23 chemical sensitivity assays (listed in Supplementary Table S1), and drawing a “phenotypic fingerprint” of the microorganism. After overnight growth, cells were collected from a single colony on the LB agar plate using a cotton swab and inoculated to achieve a bacterial cell suspension with a cell density of T (transmittance) = 90–98%. Then, 100 µL of the cell suspension was dispensed into each well. The PM, typically containing a tetrazolium redox dye, was incubated in the dark at 27 °C under aerobic conditions. Positive metabolic activity was indicated by the appearance of a purple color, resulting from the reduction of tetrazolium dye in the wells. The color change representing the level of substrate consumption was recorded by the automated Omnilog System every 15 min for 120 h, until the plateau phase was reached. Data analysis was performed using the DuctApe software (https://combogenomics.github.io/DuctApe/index.html, accessed on 15 November 2023) [21]. 

### 2.5. Phenanthrene Biodegradation Assays

Bacteria were cultured for 48 h at 27 °C and 150 rpm until reaching the late exponential phase (OD600 = 3.0–4.0). The cells were collected by centrifugation at 10,000 rpm for 10 min and washed three times with a basic salt medium (BSM) [22]. For each 500 mL Erlenmeyer borosilicate glass flask, 1 mL of PHE stock solution (60 mg/mL in acetone) was added. Acetone was allowed to evaporate, and the PHE crystals were resuspended in 100 mL of MSM medium. The resuspension process involved sonication to ensure proper mixing and dispersion of PHE in the medium. Afterward, the flasks were equilibrated overnight under agitation. Then, the bacterial inoculum was added to reach OD600 = 0.3–0.4 (corresponding to 124 μg/mL protein). The inoculated flasks were incubated in the dark for 30 days at 27 °C and 150 rpm. 

The degradation experiment was performed in triplicate by measuring the residual PHE concentration in the total culture volume [23,24]. Due to PHE’s very low solubility, traditional methods were inadequate. An aliquot (whole content) was withdrawn and treated with 2 volumes of acidified (0.1%FA, final pH 2) methanol. After incubation at 50 °C for 30 min (Ultratherm BWT-U, Biosan, Riga, Latvia), the sample was ultrasonicated in Kraintek K-5LM ultrasonic cleaner (Podhajska, Slovakia) for 30 min and further disintegrated for 20s at 30% power using a Branson SLPe digital sonifier (Thermo Fisher Scientific, Waltham, MA, USA). After the removal of cell debris, the residual PHE was measured using UPLC (ACQUITY UPLC H-Class, Waters, Manchester, UK) fitted with a ZORBAX Eclipse XDB-C18 column (4.6 × 250 mm, 5 µm). Methanol and water (90:10, *v*/*v*) containing 0.1% (*v*/*v*) formic acid were used as the mobile phase at a flow rate of 1 mL/min. Chromatography was performed for 7.5 min at 45 °C using a 1 µL injection. The analyte was detected at 254 nm. Elution time for PHE was 4.73 min (r2 = 0.9994, linear range = 25–1000 μg/mL, LOD = 1.59 μg/mL, LOQ = 4.83 μg/mL). Controls included two sets of flasks to assess abiotic loss: one with dead bacterial cells (killed control) supplemented with PHE and another with all the materials except the inoculum. Each set comprised six flasks. 

PHE degradation intermediates were identified following Cajthaml et al. [25]. Sampling occurred on days 4, 7, 12, 15, 20, 25, and 30. Briefly, 25 mL aliquots from each flask were homogenized, acidified to pH 2, and extracted three times with 10 mL of ethyl acetate for 30 min under vigorous shaking (250 rpm). After concentration and drying, the extracts were directly analyzed without any derivatization by GC–MS (MSD 5977A, Agilent, USA). Helium carrier gas (2 mL/min) was used with a DB-1701 column (30 m × 0.32 mm; Agilent, USA). The column temperature program started at 80 °C, held for 2 min, then ramped to 180 °C at 30 °C/min, and finally to 280 °C at 4 °C/min, held for 20 min. The compounds were identified by comparing the mass spectra with the NIST library and independently by interpreting the fragmentation patterns, excluding those found in uncultivated cultures.

### 2.6. Comparative Proteomics Analysis of PHE-Exposed SM-20 Cells 

Proteins from *P. veronii* SM-20 cells harvested on the 15th and 30th days post-inoculation were compared to those from washed bacterial cells used as inoculum on day 0. Briefly, proteins from the homogenized cells (in triplicate) were purified by methanol–chloroform precipitation, solubilized (8 M urea, 2 M thiourea, 40 mM TRIS-base, 1% ASB-14, 1% Triton X-100), and quantified by the Bradford assay. These data provided information on cellular growth. Then, a filter-aided sample preparation protocol was applied to reduce, alkylate, and digest the total protein extracts as described by Nováková et al. [26]. Finally, the peptide mixture was desalted on SEP PAK C18 light columns (Waters), vacuum-concentrated to 0.5 μg/μL, and spectrophotometrically assessed by NanoDrop 2000 (ThermoFisher Scientific). 

Desalted peptides underwent separation on a nanoEaseTM M/Z HSS C18 T3 column (200 mm length, 75 μm diameter, 1.8 μm particle size) with a 90 min gradient of 3–35% acetonitrile containing 0.1% formic acid at 300 nL/min. Eluted peptides were nano-sprayed (2.65 kV capillary voltage) to a Synapt G2-Si mass spectrometer in high definition MSE mode. Ions (50–1950 *m*/*z*) were recorded in both channels at a 1s spectral acquisition scan rate. The external mass calibrant Glu1-Fibrinopeptide B (500 fmol/mL) corrected mass variations.

The peptides were then separated on a nanoEaseTM M/Z HSS C18 T3 analytical column (200 mm length, 75 μm diameter, 1.8 μm particle size) with a 90 min gradient of 3–35% acetonitrile containing 0.1% formic acid at 300 nL/min. Eluted peptides were nano-sprayed (2.65 kV capillary voltage) to a mass spectrometer Synapt G2-Si (Waters). Spectra were recorded in a data-independent manner in high definition MSE mode. Ions (50–1950 *m*/*z*) were detected in both channels, with a 1s spectral acquisition scan rate. The external mass calibrant Glu1-Fibrinopeptide B (500 fmol/mL) was infused through the reference line for mass correction. 

Data processing in Progenesis QI 3.0 (Waters) involved peak-picking and correlation of precursors and fragment ions. Label-free quantification used the measured peak areas of the three most intense precursor peptides, preferentially unique. For protein identification, the Ion Accounting (Waters) search algorithm incorporating a reverse-sequence/random decoy database and a false-positive rate threshold of 4% was applied. *P. veronii* 1YdBTEX2 protein sequences from the UniProt database (accessed on 14 December 2020, 7333 entries, http://www.uniprot.org) were selected due to taxonomy affiliation and a similar isolation source (also isolated from polluted soil in the Czech Republic). Identifications were accepted with at least two distinct reliable peptides (score ≥ 5.0, mass accuracy ≤ 15 ppm, reliability threshold ≥ 95%). Differentially accumulated proteins were chosen based on ANOVA *p* ≤ 0.05 and fold change ratio ≥ 1.8 (0.84799-fold of log2). A post hoc Tukey’s HSD test was used for significant changes. Volcano plots were generated using VolcaNoseR (version 1.0.3) [27].

Protein information was obtained from the Expasy Proteomics Server (http://au.expasy.org, accessed on 30 December 2022) and Brenda Enzyme Database (https://www.brenda-enzymes.org/, accessed on 30 December 2022). Subcellular localization predictions used PSORTb version 3.0.2 (https://www.psort.org/psortb, accessed on 2 January 2023) [28] and SOSUI-GramN (https://harrier.nagahama-i-bio.ac.jp/sosui/sosuigramn/sosuigramn_submit.html, accessed on 2 January 2023) [29]. Protein classification into COGs functional classes was carried out using EggNOG v5.0 (http://eggnog5.embl.de/#/app/home, accessed on 23 January 2023) [30], allowing a maximum of 3 most probable orthologs for each identified protein.

## 3. Results and Discussion

### 3.1. Phylogenomic and Metabolic Potential of the Newly Isolated Strain SM-20 as Compared with Other Pseudomonas Strains

**Figure 1:**
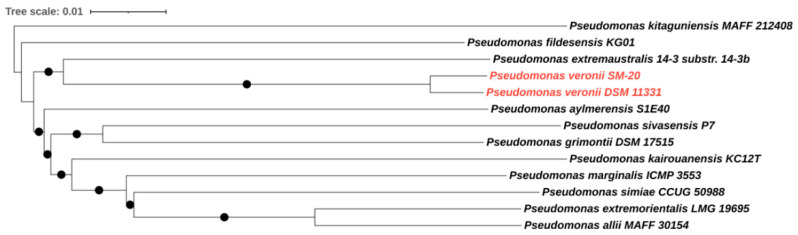
Genome BLAST Distance Phylogeny (GBDP) tree of *P. veronii* strain SM-20. The GBDP distances were calculated using the Type (Strain) Genome Server (TYGS) according to formulae d5. Only pseudo-bootstrap support values > 60% are shown (100 replications). The tree was rooted at the midpoint and visualized with iTOL. *P. veronii* strain SM-20 and the type strain of the species *P. veronii* are highlighted in red.

The complete genome of *P. veronii* isolate SM-20 was obtained by combining Illumina and Oxford Nanopore sequencing to acquire detailed information on the taxonomic classification of the newly isolated strain and to gather information on the genomic functions associated with aromatic compound degradation. The genome analysis revealed a total genome size of 6,947,583 bp with a GC content of 60.6%, which is consistent with other *Pseudomonas* species [31,32]. Our strain exhibited an 87.4% similarity (determined by digital DNA–DNA hybridization formulae d4; dDDHd4) to the type strain of *P. veronii* DSM 11,331 (Figure 1), confirming its classification within the *P. veronii* species. 

According to the RAST annotation, 88 RNA genes were identified, and 6460 protein-coding genes were predicted. Protein-coding genes were categorized into 397 subsystems. The most abundant categories corresponded to amino acids/derivatives (552), carbohydrates (313), protein metabolism (226), and vitamins, prosthetic groups, or pigments (215). Additionally, genes associated with osmotic and oxidative stress were identified. When considering genes involved in the metabolism of aromatics, we identified genes encoding proteins participating in various pathways such as the protocatechuate branch of beta-ketoadipate or benzoate degradation. Some of these genes formed clusters (Supplementary Table S2), while others were scattered throughout the genome, including those involved in benzoate, toluene, and phenol transport and metabolism. For example, the *cat* operon was situated in a cluster composed of genes encoding proteins required for benzoate degradation and the aromatic hydrocarbon utilization transcriptional regulator CatR (LysR family). Other crucial genes within this category encode catechol 2,3-dioxygenase, protocatechuate 3,4-dioxygenase alpha and beta chains, aromatic-ring-hydroxylating dioxygenase beta subunit, and the ring hydroxylating dioxygenase alpha subunit/Rieske (2Fe-2S) protein. Genes involved in the transport and uptake of mono- and polyaromatic compounds accounted for approximately 10 percent of the whole genome in the SM-20 genome. These include genes encoding transmembrane transport-related proteins such as those belonging to the major facilitator superfamily (MFS) and ATP-binding cassette (ABC) transporter superfamilies. Specifically, 52 genes were identified for MFS-type transporters and 285 genes for ABC transporters, which encompass a variety of permeases (PstA, PstC, HisP, HisM), substrate-binding proteins, ATP-binding proteins, polyol ABC transporters, and efflux ABC transporters. Additionally, genes encoding outer membrane proteins (OMPs) were found in the genome, with some, like the low permeability porin, located between transposases, mobile elements, lipoproteins, TonB-dependent receptors, and the sigma-54 regulator. Other outer membrane genes were situated near genes encoding quinone oxidoreductase and transcriptional regulators of the LysR and AraC families, as well as MFS-type transporters, to mention a few. Among the protein-encoding genes related to symporters and antiporters, multidrug efflux systems (MdtABC-TolC, MdtC, TriC, TriABC)/drug antiporters, and cation antiporters were identified. 

**Figure 2:**
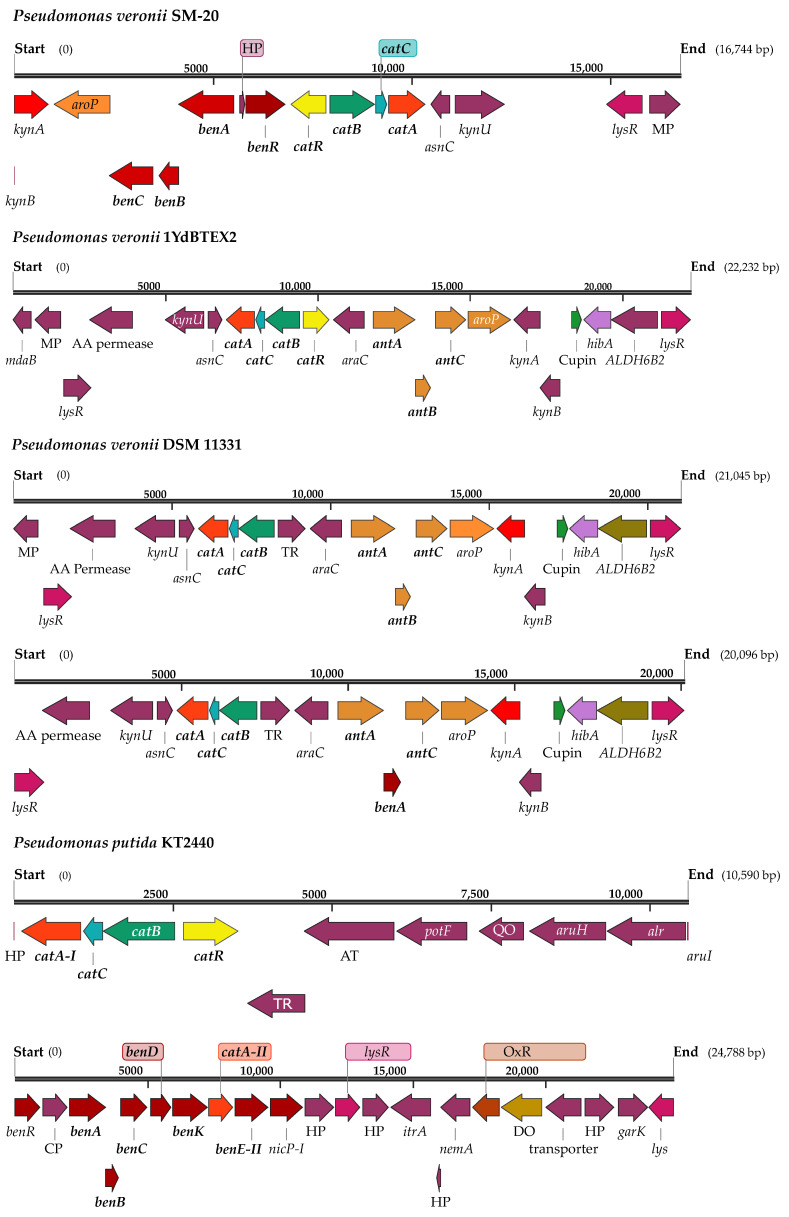
Schematic representation of the gene clusters harboring the gene encoding the catechol 1,2-dioxygenase (*catA*) in different strains of *Pseudomonas veronii* and in *P. putida* KT2440. ***kynA***—tryptophan 2,3-dioxygenase (EC 1.13.11.11); ***aroP***—aromatic amino acid transport protein; ***benC***—benzoate dioxygenase, ferredoxin reductase component (EC 1.14.12.10); ***benB***—benzoate 1,2-dioxygenase beta subunit (EC 1.14.12.10); ***benA***—benzoate 1,2-dioxygenase alpha subunit (EC 1.14.12.10); **HP**—hypothetical protein; **AT**—putative aminotransferase; ***benR***—*benABC* operon transcriptional activator; ***catR***—aromatic hydrocarbon utilization transcriptional regulator (LysR family); ***catB***—muconate cycloisomerase ( EC 5.5.1.1); ***catC***—muconolactone isomerase (EC 5.3.3.4); ***catA***—catechol 1,2-dioxygenase (EC 1.13.11.1); ***asnC***-AsnC family transcriptional regulator; ***kynU***—kynureninase (EC 3.7.1.3); ***lysR***—LysR family transcriptional regulator; **MP**—membrane protein; ***mdaB***-modulator of drug activity B; **AA permease**—amino acid permease; ***araC***—Arac family transcriptional regulator; ***antA***—anthranilate 1,2-dioxygenase large subunit (EC 1.14.12.1); ***antB***—anthranilate 1,2-dioxygenase small subunit (EC 1.14.12.1); ***antC***—anthranilate 1,2-dioxygenase electron transfer component (EC 1.14.12.1); ***kynB***- kynurenine formamidase (EC 3.5.1.9); ***hibA***—probable 3-hydroxyisobutyrate dehydrogenase (EC 1.1.1.31); ***ALDH6B2***—methylmalonate-semialdehyde dehydrogenase (EC 1.2.1.27); **TR**—transcriptional regulator; ***potF***—putrescine binding protein; **QO**—quinone oxidoreductase; ***aruH***—Arginine pyruvate transaminase (EC 2.6.1.84); ***alr***—alanine racemase (EC 5.1.1.1); ***aruI***—putative 2-ketoarginine decarboxylase (EC 4.1.1.75); **CP**—conserved protein of unknown function (PP_3160); repeat protein; ***benD***—1,2-dihydroxycyclohexa-3,5-diene-1-carboxylate dehydrogenase (EC 1.3.1.25); ***benK***—benzoate MFS transporter; ***benE***—benzoate transport protein; ***nicP***—benzoate-specific porin; ***itrA***—group II intron-encoding maturase; ***nemA***—N-ethylamide reductase; **OxR**—putative oxidoreductase; **DO**—putative dioxygenase; ***garK***—glycerate kinase (EC 2.7.1.165).

When compared to other *P. veronii* strains, the bioinformatic analyses indicated a certain level of intraspecies variation in coding sequences (CDS) involved in aromatic metabolism and its regulation. The *P. veronii* 1YdBTEX2 [30] strain was selected for comparison purposes due to its isolation from a similar environment, while the canonical strain *P. veronii* DSM 11331 reported in 1996 by Elomari and collaborators was isolated from mineral water. In contrast to the reference strain *P. veronii* 1YdBTEX2 [32], SM-20 exhibited a higher number of genes involved in benzoate degradation. For instance, 1YdBTEX2 lacked a gene encoding benzoate 1,2-dioxygenase, a function predicted in SM-20 and annotated as benzoate/toluate 1,2-dioxygenase subunit alpha (K05549). This gene flanked the catechol degradation cluster (Figure 2), suggesting SM-20′s potential to degrade benzoate through meta- or ortho-cleavage degradation pathways [33]. Another example of intraspecies variation concerns the genes encoding catechol 1,2 and catechol 2,3-dioxygenase present in SM-20 and other *P. veronii* strains, namely *P. veronii* 1YdBTEX2 [11], *P. veronii* Pvy (GenBank CP039631.3), and *P. veronii* R02 (GenBank: CP018420.1). The schematic representation in Figure 2 shows that the organization of the catechol 1,2-dioxygenase gene (*catA*) in SM-20 differs from the above-mentioned strains. In our strain, the *catABC* operon is flanked by the aromatic hydrocarbon utilization transcriptional regulator, the *benABC* operon transcriptional activator *benR*, the benzoate 1,2-dioxygenase alpha and beta subunit, transcriptional regulators, membrane proteins, and the modulator for drug activity. In contrast, the strains *P. veronii* Pvy (GenBank CP039631.3) and *P. veronii* R02 (GenBank: CP018420.1) lack genes involved in benzoate degradation (the *benABC* operon transcriptional activator *benR* and the benzoate 1,2-dioxygenase alpha and beta subunits) and genes encoding the hydrocarbon utilization regulator in the vicinity of the *catABC* operon. This genetic clustering could provide advantages in the regulation and coordination of the expression of genes involved in aromatic degradation [34], thereby enhancing biodegradation efficacy. 

Likewise, in the reference strain (*P. veronii* 1YdBTEX2), the catechol 2,3-dioxygenase gene is situated upstream of a ferredoxin-coding gene followed by the *dmpR* gene and downstream of the *bmpCD*, and the *dmpEFGH* accompanied by a series of hypothetical protein-coding genes. In strain SM-20, the catechol 2,3-dioxygenase gene was located in three distinct regions that formed two separate clusters showing a different genome organization (Supplementary Figure S1). The gene located at position 1,110,998 is situated downstream of the elements of the methylphenol operon *dmpKLMNOP* and some genes involved in the isopropylbenzene pathway *ipbG,E,F*. Downstream, the catechol 2,3-dioxygenase gene is flanked by a hypothetical protein, a positive regulator of the phenol hydroxylase, an exporter RND protein, a BNR repeat protein, a couple of hypothetical proteins, and finally the transcriptional regulator CatR (LysR family) involved in aromatic hydrocarbon utilization. Interestingly, the two genes in the second cluster are separated from each other by a segment of 5966 bp composed of the *dmpR* gene, which encodes a phenol hydroxylase positive regulator, along with two genes encoding hypothetical proteins. Upstream, the first catechol 2,3-dioxygenase gene of this cluster was proximal to the *dmpCEFGH* genes, while the second catechol gene was downstream of the genes *benA* and *benB* that were followed by genes encoding hypothetical proteins, a transposase, and mobile elements. Regarding another important player in the degradation of aromatic compounds, the 2,3-hydroxybiphenyl 1,2-dioxygenase, strain SM-20 bears one gene flanked downstream by the 2-hydroxymuconic semialdehyde hydrolase and a couple of inner membrane components of the tripartite multidrug resistance system genes (Supplementary Figure S2). Upstream, genes encoding an oxidoreductase, an indoleacetamide hydrolase, a racemase, a deacetylase, a polyprenylphenol hydrolase, hypothetical proteins, and a Rieske domain protein (2Fe-2S) are located. *P. veronii* 1YdBTEX2 had a gene encoding a 2,3-hydroxybiphenyl 1,2-dioxygenase (EC 1.13.11.39) flanked by elements of the isopropylbenzene cluster, i.e., the *ipbAa*, *Ab*, *Ac*, *Ad*, a transposase, and the *ipbB1,2,3* on one side, and the *ipbE*,*G*,*F*, an endonuclease DDE, an outer membrane protein, the *ipbD*, another endonuclease DDE, and an integrase on the other side. When comparing to different species of the *Pseudomonas* genus, *P. putida* strain KT2440 [35] bears two *catA* genes with different localization in its genome compared to the strains *P. veronii* SM-20 and *P. veronii* 1YdBTEX2. The first gene is flanked by the *benABCDKR* operon. At the same time, both the *ben* and the *catA* gene are flanked by proteins of unknown function. The second gene is part of the *catABC* operon and is flanked on one side by proteins of unknown function, a phage repressor, and a gene encoding the diguanylate cyclase, involved in secondary messenger production. The *catR* gene is in proximity to genes encoding proteins with conserved amino acid motifs and a putrescine binding periplasmic domain. Finally, *P. putida* KT2440 (GenBank AE015451.2) did not show the presence of the catechol 2,3-dioxygenase, any component of the *dmp* operon, biphenyl, or phenol pathways.

### 3.2. Phenotypic Analysis of P. veronii Strain SM-20 

Phenotype microarray analysis was conducted to evaluate the main metabolic capabilities of the novel isolated strain, as well as its resistance to stressors. *P. veronii* strain SM-20 exhibited metabolic activity under approximately half (40 out of 96 wells) of the conditions tested in the GENIII plate (Supplementary Figure S3), without demonstrating a specific metabolic trend. Most wells showed low metabolic activity (activity index (AI) = 1, orange wells), while twelve wells displayed medium metabolic activity (AI = 2 or 3, light green wells) and three wells exhibited the maximum activity values (AI = 4, dark green) (Supplementary Figure S4). The highest metabolic activity of *P. veronii* strain SM-20 was observed in the presence of D-galactose, D-glucuronic acid, and glucuronamide. Additionally, the strain demonstrated the ability to utilize several other carbon compounds, such as gentiobiose, turanose, L-rhamnose, melibiose, glucose, mannose, L-fucose, and fructose 6-phosphate, while exhibiting lower activity in the presence of glucose 6-phosphate and no activity in the presence of sugar alcohols (Supplementary Figure S3). Comparing the phenotypic analysis performed in this work with that of the reference strain [36], *P. veronii* SM-20 exhibited a higher metabolic activity on mono- and disaccharides, such as L-rhamnose (see well C08 in Supplementary Figure S3). Consistent with this, we identified genes coding a dTDP-4-dehydrorhamnose reductase (EC 1.1.1.133) and a dTDP-4-dehydrorhamnose 3,5-epimerase (EC 5.1.3.13) related to rhamnose synthesis. Similar to a previously studied *P. aeruginosa* strain [37], the SM-20 strain was unable to use D-serine as a carbon source, suggesting that this amino acid can only be utilized as a nitrogen source. 

In the wells testing different stressors, growth was detected to some extent in most conditions, indicating some level of resistance of this strain to some stress conditions. Particularly, the SM-20 strain exhibited tolerance to tetrazolium violet. Conversely, it was sensitive to rifamycin, lithium chloride, and guanidine HCl, contrary to what was reported in other species of *Pseudomonas* genus, such as *P. citronellolis* [38]. This is in line with well-known variability in stress resistance capacity among different *Pseudomonas* species due to the broad genetic variability [39]. Regarding genes associated with resistance to stressors, several genes of the SM-20 genome are involved in resistance mechanisms to antibiotics and other toxicants (e.g., bleomycin). Among these, seven genes encoding β-lactamases (class C, metallo-beta-lactamase family protein) were predicted, along with genes related to resistance to fluoroquinolones, minocycline, fosfomycin, fosmidomycin, aztreonam, streptothricin, and polymyxin. Two genes encoding metallo-β-lactamase family proteins (particularly, A0A1D3JY58 and A0A1D3JVR7) may be involved in the resistance of this strain to aztreonam [40]. SM-20 also possesses several genes related to multidrug efflux systems (DMT superfamily; RND type; MExB-MexA-OprM; MATE; OMF; tetrapartite efflux system) known to mediate resistance to antibiotics in Gram-negative bacteria [41,42]. These efflux systems prevent the entrance of antibiotics into the cell by controlling outer membrane permeability or by increasing the effectiveness of the extruding toxic molecules [43]. Consistent with this, the proteomic analysis conducted in this study showed the expression of the toluene efflux pump outer membrane protein (TtgA, TtgC), a multidrug ABC transporter, ABC transporter permease, an efflux pump membrane transporter, and several ABC transporter substrate-binding proteins during SM-20 cellular growth on PHE.

### 3.3. Growth of P. veronii Isolate SM-20 in the Presence of PHE as the Only Carbon and Energy Source and Identification of PHE Catabolic Intermediates

**Figure 3:**
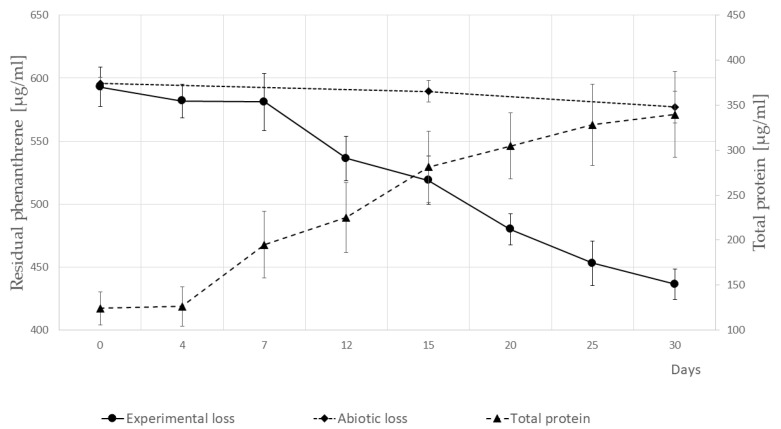
Degradation of phenanthrene and growth curve of the *P. veronii* strain SM-20 in the presence of phenanthrene as the sole carbon and energy source. The experiments were conducted at a constant temperature of 27 °C, and the average pH observed was 6.7. The abiotic loss is represented by the control (no inoculum). Error bars represent the ± standard deviation (number of replicates = 3). The two-tail Student’s *t*-test assessed the statistical significance of differences.

The capacity of the newly isolated strain SM-20 to utilize a model PAH was tested by inoculating the isolate in liquid minimal medium containing PHE as the sole carbon and energy source at a concentration of 600 µg/mL. The growth curve and PHE removal were monitored over time. PHE depletion occurred gradually, resulting in a reduction of the contaminant by approximately 25% within 30 days (Figure 3). The biodegradation performance observed in the inoculated assay was about 8.5 times higher than that in the controls lacking cellular biomass, with 3% of the removal attributed to abiotic loss. Although bacterial biomass increased during the initial seven days, PHE removal was limited. Ultimately, after one month of cultivation, the fold increase in biomass was 2.5 times in terms of protein content (Figure 3).

Several *Pseudomonas* strains have previously been described as capable of degrading PAHs such as PHE [44,45] or naphthalene [12]. Depending on the strain and the initial PHE concentrations, previous studies have reported biodegradation times ranging from two to fifteen days in liquid cultures [46] and from forty to sixty days in soil or reactor systems [44,45,46,47]. Additionally, concerning biomass growth*, Pseudomonas* strain LZ-Q and *P. stutzeri* ZP2 achieved a 10-fold increase in biomass production [44,48] in the presence of 1000 and 250 mg/L of PHE, respectively. On the other hand, strains of *P. pseudoalcaligenes* and *P. aeruginosa* did not exhibit significant growth over a 30-day period in experiments with an initial PHE concentration of 400 mg/L [49]. In our study, SM-20 reduced PHE concentration by 25% over a 30-day period, accompanied by a 2.5-fold increase in biomass. This performance is comparable to that described for *Pseudomonas* strain PB-1, which metabolized around 26% of naphthalene, a double-ring aromatic compound, and was considered an efficient degrader [50]. The variation in biodegradation performance and biomass growth might be attributed to differences in the strain’s capacities to cope with the extremely low solubility of PHE in water, coupled with differences in genetic functions involved in its metabolism and transport. We therefore hypothesized that the extended time required for PHE degradation by SM-20 may be attributed to the initial period needed for cellular adaptation to the toxic molecule and for the expression and regulation of proteins involved in its metabolism/degradation (as indicated by proteomic analysis). Moreover, Volkering and collaborators [51] demonstrated that the mass transfer of weakly soluble substrates can limit microbial degradation, leading to non-exponential growth of microorganisms [46] and low PHE metabolism. Hua and Wang [52] also reported that the degradation rate of PHE in *Pseudomonas* species significantly depends on its dissolution rate. Alternatively, the low rate of PHE degradation may result from the formation of dead-end products that could be toxic to the cells [4].

In our study, we also determined the metabolic intermediates produced by SM-20 during growth on the model PAH. Over the 30-day course of the experiment, we identified five metabolic intermediates. Table 1 shows their retention times and mass spectra characteristics, including fragment ions (corresponding mass spectra and fragmentation schemes are shown in Supplementary Figures S5–S9).

Notably, phenathrenedione (product no. 2) was consistently observed from day 20 till the end of the experiment. By day 30, all the other intermediates were detectable. These were identified as phenanthrene 9,10-oxide (1), o-hydroxybiphenyl (3), phthalic anhydride (4), and 2-coumaranone (5). Compound 1 is a K-region arene oxide generated by the epoxidation of PHE. Compound 3 was probably produced by the decarboxylation and hydroxylation of biphenyl-2-carboxylic acid, an intermediate in diphenic acid metabolism. The last two compounds, 4 and 5, might have been formed during GC/MS analysis from phthalic acid and 2-hydroxymethyl-benzoic acid, respectively. To verify that these oxidation products resulted from the biodegradation of PHE, the control samples were similarly analyzed. None of the identified compounds were detected in the extracts of the control samples without inoculum or in the *P. veronii* cells inactivated by boiling (120 °C for 5 min). These results demonstrate that the metabolites detected in the target samples are associated with microbial activity/degradation of the aromatic compound (PHE).

**Figure 4:**
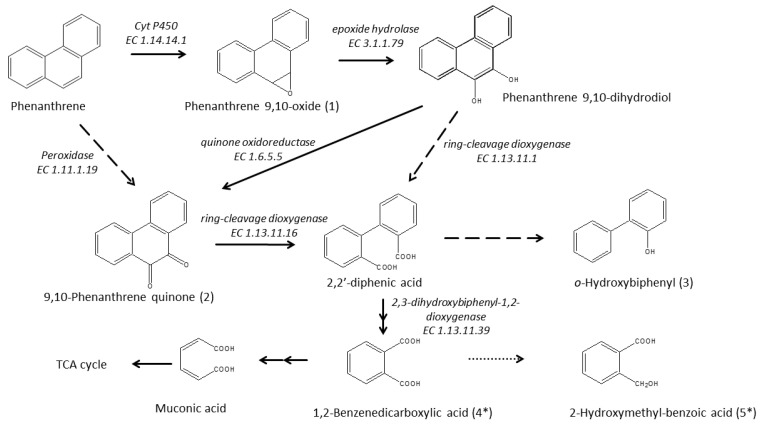
Proposed pathway of PHE transformation by *P. veronii* isolate SM-20. Intermediates labeled with Asterisk were identified in their dehydrated forms, produced during GC/MS analysis.

The time-dependent formulation of PHE metabolites in the liquid media, identified by GCMS, provides insight into the degradation reactions carried out by *P. veronii* isolate SM-20. Figure 4 illustrates the proposed pathway, resembling the PHE degradation observed under ligninolytic conditions in fungi such as *Pleurotus ostreatus* [53], *Polyporus* sp. S133 [54], *Trametes hirsuta* [55], *Phanerochaete chrysosporium* [56], and *Aspergillus niger* [57], as well as in some bacteria like *Ensifer meliloti* [58], *Mycobacterium vanbaalenii* [59], *Mycobacterium aromativorans* [60], and *P. anguilliseptica* [61].

### 3.4. Proteomic Profiling of P. veronii SM-20 in Response to PHE Exposure

To offer a dynamic overview of the cellular processes ongoing under PHE exposure, we analyzed the proteomic armory of the bacterial cells (in triplicate) before (sample named control) and after being exposed to PHE for 15 and 30 days (samples named 15d-SM-20 and 30d-SM-20) using a gel-free proteomic approach. Proteomic analysis resulted in the identification and quantitation of 1145 proteins (13,119 peptides) in total. Among these, 984 proteins showed more than two matching peptides and satisfactory scores (95–100% confidence level) (Supplementary Figure S10A and Supplementary Tables S3 and S4). The distribution of these proteins among 15d-SM-20, 30d-SM-20, and the control sample is displayed in Supplementary Figure S10B. The Principal Component Analysis revealed proper clustering of experimental replicates in two-dimensional space, indicating the robustness and confidence of our findings (Supplementary Figure S10C). Differences in proteome profiles are predominant between the control and the PHE-exposed cells, while proteomes of bacteria exposed to PHE for 15 and 30 days clustered more closely together.

We identified 162 differentially abundant proteins (fold change ratio > 1.8), which met the ANOVA *p*-value and post hoc Tukey’s HSD test thresholds (Supplementary Tables S3 and S5). The heatmap in Supplementary Figure S11A shows their distribution. These proteins were then assigned to different subcellular localizations (Supplementary Figure S11B) and functional categories according to COG (Supplementary Figure S11C and Supplementary Table S4). Upon examining the group of upregulated proteins, we observed a noticeable increase in the proportion of proteins localized to the outer membrane, compared to their presence in the entirety of identified or downregulated proteins (Supplementary Figure S11B). Consistently, within the upregulated proteins, there was a notably higher proportion classified under category M (cell wall, membrane, and envelope formation) as opposed to the downregulated proteins (Supplementary Figure S11C). This is particularly evident in the cells exposed to PHE for 30 days compared to the control. Interestingly, proteins related to COG categories P (inorganic ion transport and metabolism), Q (secondary metabolites biosynthesis, transport, and catabolism), and V (defense mechanisms) were also significantly accumulated. These proteins might be involved in mechanisms of tolerance, adaptation, and survival of *P. veronii* SM-20 during growth on the contaminant.

### 3.5. Enzymes Involved in Aromatic Hydrocarbon Metabolism and Transportation

The proteomic repertoire of *P. veronii* SM-20 (Supplementary Table S4) was analyzed for proteins identified after PHE exposure, focusing on those with potential functions in metabolism, transport, and/or regulation of aromatic hydrocarbons. Our study uncovered a diverse array of enzymes, as listed in Table 2, likely involved in the degradation of aromatic and polyaromatic compounds in strain SM-20. These proteins were further correlated with gene IDs obtained from our genomic annotation to elucidate their genetic basis.

The detection of catechol 1,2-dioxygenase (C12DO, EC 1.13.11.1—A0A1D3K3R6; encoded by *catA* gene—peg.4971), catechol 2,3-dioxygenase (CatO2ase, EC 1.13.11.2.—A0A1D3K906; C23O gene—peg.984, 3084, 3088), protocatechuate 3,4-dioxygenase (EC 1.13.11.3.—A0A1D3JTA7; *pcaG*), and muconolactone delta-isomerase (EC 5.3.3.4.—A0A1D3K3L8; *catC*—peg.4970) suggests that strain SM-20 may utilize aromatic and polyaromatic compounds through the beta-ketoadipate pathway. The first two enzymes indicate the potential utilization of both ortho- and meta-cleavage pathways in aromatic compound degradation [62]. Homogentisate 1,2-dioxygenase (EC 1.13.11.5.—A0A1D3JSA5; *hmgA—*peg.2743) [63] has the same function as C12DO in different *Pseudomonas* species, including *P. chlororaphis* strain UFB2 [64]. The third and fourth enzymes facilitate the conversion of protocatechuate or muconolactone to β-ketoadipate. Furthermore, the presence of 4-hydroxy-4-methyl-2-oxoglutarate aldolase (EC 4.1.3.17—A0A1D3K2B2; peg.5665) indicates the strain’s ability to use the 4,5-protocatechuate cleavage pathway [65]. Thus, the presence of these enzymes suggests the possibility of two degradation branches through catechol or protocatechuate, as observed in *P. putida* N6 [62,66]. These enzymes also play a role in the lower degradation pathways of aromatic hydrocarbons in bacteria when the compounds have more than one benzene ring [67]. 

Additionally, the 2,3-dihydroxyphenylpropionate 1,2-dioxygenase (EC 1.13.11.16.—A0A1D3JZS8; *mhpB1—*peg.1056, 1440) is a well-known extradiol catechol dioxygenase, which is grouped in the metabolism of aromatic compounds. This enzyme catalyzes the cleavage of the aromatic ring of 2,3-dihydroxyphenylpropionate into 2-hydroxy-6-ketononatrienedioate, a key step in the degradation of aromatic compounds, particularly those derived from lignin breakdown or other phenolic compounds. Also, phenol/toluene 2-monooxygenase/hydroxylase (NADH) P1/A1 (EC 1.14.13.7.—A0A1D3JYN7; *dmpL*—peg.982) and phenol/toluene 2-monooxygenase/hydroxylase (NADH) P4/A4 (EC 1.14.13.7.—A0A1D3JYZ4; peg.979) catalyze the hydroxylation of phenol or toluene. This enzyme was, for instance, identified in the *P. stutzeri* genome [68]. Furthermore, 2,3-dihydroxybiphenyl 1,2-dioxygenase (EC 1.14.12.18.; *bphC*—peg.4843), assigned to the protein identifier (A0A1D3K930), provides insight into the strain’s potential to utilize biphenyl [69,70]. On the other hand [65], quercetin 2,3-dioxygenase (EC 1.13.11.24.—A0A1D3JYG5; peg.937) demonstrates a wide range of enzymatic reactions, including dioxygenase function [71]. Interestingly, we also detected an analogous enzyme with a different identifier (A0A1D3K115; PIR gene—peg.1907), which corresponds to a pirin from the cupin superfamily. A similar pirin-like protein showing quercetinase activity was reported in *P. stutzeri* [72] and *E. coli* [73]. Thus, these diverse enzymes underline the metabolic versatility of the strain in metabolizing various aromatic substrates, which has significant implications for environmental bioremediation and biotechnological applications. 

In addition to enzymatic degradation, the transportation of these aromatic compounds within the cell is equally crucial. Our proteomic analysis revealed significant upregulation of two TonB-dependent receptors (A0A1D3JXF4, A0A1D3JZA0) by the 30th day. These receptors are recognized as mediators of substrate-specific transport across the outer membrane [74,75], demonstrating a wide range of substrate utilization potential. TonB systems are generally known for their involvement in the uptake of siderophores, vitamin B12, and saccharides. However, their role in the uptake and catabolism of aromatic compounds remains unclear. Fujita et al. [76] showed the participation of the TonB system in the uptake and transport of a biphenyl compound in *Sphingobium* sp. strain SYK-6. The overproduction of these proteins in the presence of xenobiotic compounds was also confirmed by Samantarrai et al. [77], indicating their importance in nutrient-limited environments [78]. Furthermore, among the ABC transporters, a branched-chain amino acid ABC transporter substrate-binding protein (A0A1D3JVQ9) was found to be significantly accumulated on both sampling days. Homologs of this protein were identified from *R. palustris* binding specific small aromatic molecules typically produced during lignin degradation [79]. In a broader sense, Michalska et al. [79] demonstrated the involvement of some ABC transporters in the uptake of benzoate derivatives in various members of the *Alphaproteobacteria* class.

### 3.6. Enzymatic Pathway for PHE Catabolism

The pathway presented in Figure 4 involves the activity of the significantly accumulated (on day 15) cytochrome P450 monooxygenase enzyme (EC 1.14.14.1—A0A1D3JPQ1; encoded by *cyp450* gene—peg.3915) in the initial oxidation of PHE, which begins in its “K-Region” [61]. This enzyme catalyzes a ring epoxidation, resulting in the formation of an unstable arene oxide intermediate—phenanthrene 9,10-oxide (intermediate 1), which is further transformed to phenanthrene 9,10-dihydrodiol through an epoxide-hydrolase reaction, as proposed by Jerina [80] and Ghosal et al. [4]. It is probable that *P veronii* isolate SM-20 catalyzes the hydration of the arene oxide via alpha/beta hydrolase (EC 3.1.1.79—A0A1D3JXH6; *ephA—*peg.538), exhibiting epoxide hydrolase activity. This enzyme was particularly identified only on day 30 of the study. The formation of 9,10-phenanthrenequinone (intermediate 2) is then facilitated by the highly accumulated quinone oxidoreductase (EC 1.6.5.5—A0A1D3K0Y9; TP53I3 gene—peg.1742). Alternatively, intermediate 2 can also result from the direct oxidation of the phenanthrene ring, mediated by cytochrome P450, and two-electron oxidation by a “DyP-type” family peroxidase (EC 1.11.1.19—A0A1D3JW33, detected only on day 30; *yfeX*—peg.6376). Subsequently, the isolate converts 9,10-phenanthrene quinone to 2,2′-diphenic acid via ring-cleavage, potentially involving 2,3-dihydroxyphenylpropionate/2_3-dihydroxycinnamic acid 1_2-dioxygenase (EC 1.13.11.16—A0A1D3JZS8; *mhpB1—*peg.1440), also identified only on day 30. Alternatively, diphenic acid could also be produced by the ring-cleavage of phenanthrene 9,10-dihydrodiol, catalyzed by dioxygenases like catechol 1,2-dioxygenase (EC 1.13.11.1—A0A1D3K3R6; *catA—*peg.4971) or protocatechuate 3,4-dioxygenase (EC 1.13.11.3—A0A1D3JTA7; *pcaG*) [81]. These enzymes are known to exhibit broad substrate specificity [82]. It is noteworthy that diphenic acid is approximately 1000 times more soluble than PHE [83] and is more susceptible to further degradation by other organisms present in soils or sediments [61]. However, this compound was not detected in any culture; only its decarboxylated and hydroxylated form, o-hydroxybiphenyl (intermediate 3), was observed. Finally, at the end of incubation, the dehydrated forms of 1,2-benzenedicarboxylic acid (4) and 2-hydroxymethylbenzoic acid (5) were found. These compounds likely arose from the transformation of 2,2′-diphenic acid, possibly via the biphenyl pathway, with CO_2_ and H_2_O as the final products. This transformation may have been facilitated by the involvement of 2,3-dihydroxybiphenyl 1,2-dioxygenase (EC 1.13.11.39; *bphC—*peg.4843). On the other hand, o-hydroxybiphenyl can undergo further degradation by a monooxygenase [84], likely phenol hydroxylase (EC 1.14.13.7—A0A1D3JYN7, A0A1D3JYZ4; peg.982, peg.979) [85], identified in PHE-exposed cells, which shares homology with 2-hydroxybiphenyl 3-monooxygenase (encoded by *hbpA*).

### 3.7. Proteins Involved in Bacterial Surface Remodeling and Stress Response

In this study, numerous integral membrane proteins exhibited significant accumulation in PHE-exposed cells (Supplementary Table S5). For example, the SurA chaperone, one of the key chaperones in the outer membrane protein (OMP) biogenesis network in Gram-negative bacteria, was prominently observed. Its importance relies on its genetic interaction with the β-barrel assembly machinery complex and its ability to prevent unfolded OMP aggregation that might affect physiological roles [86]. Therefore, these findings may indicate that the primary resistance strategy to PHE exposure is the defensive remodeling of the cell surface by making it less permeable. Indeed, the bacterial membrane is the first defense barrier, and the phospholipid bilayer creates a hydrophobic region that can accumulate hydrophobic compounds like PAHs [87,88]. In line with this, studies on alkane-degrading bacteria have reported microstructural modifications on intracellular membranes and the formation of hydrocarbon inclusions within the cells [89]. Possible resistance mechanisms towards toxic hydrophobic compounds are also mediated by bacterial efflux pumps that can play crucial roles in resistance mechanisms by controlling intracellular and membrane concentrations of hydrophobic compounds [90]. Bugg et al. [87] suggested that *P. fluorescens* uses an active efflux system to regulate the concentration of cell-associated PAHs. In this study, the two detected toluene efflux pump outer membrane proteins (A0A1D3JTJ5, A0A1D3JTE0), efflux pump membrane transporter (A0A1D3JTP5), and the TolC family protein (A0A1D3JPT9) were found uniquely present or more abundant in the cells exposed to PHE. Also, the pyrroloquinoline quinone (PQQ) synthase (A0A1D3K565) was found to be significantly accumulated. This enzyme is essential for PQQ biosynthesis, initially characterized as a redox cofactor for membrane-bound dehydrogenases in the bacterial system. Subsequently, PQQ was shown to be an antioxidant protecting living cells from oxidative damage in vivo. It maintains the redox homeostasis and preserves biomolecules from reactive oxygen species, such as superoxide, singlet oxygen, hydroxyl, or per-hydroxyl radicals, formed during growth under stress conditions. Nevertheless, PQQ can also function as a nutrient or vitamin (member of the B-group) that can support the development of living cells under stress conditions [91]. Likewise, a ferripyoverdine receptor (A0A1D3JZE2) was associated with growth stimulation [92]. This outer membrane receptor mediates the uptake of the pyoverdine-Fe (III) complex [93], which may also act as a virulence factor in many Gram-negative bacteria [94].

## 4. Conclusions

Our study elucidated the genomic and proteomic characteristics of *P. veronii* strain SM-20, a novel isolate obtained from a polluted soil sample. Phenotypic analysis underlined the metabolic versatility of SM-20, as evidenced by its ability to utilize various carbon compounds and show resistance to stressors. Particularly noteworthy is the metabolic activity of the strain in the presence of D-galactose, D-glucuronic acid, and glucuronamide, among others, indicating its potential for diverse substrate utilization.

Whole genome sequencing and comparative genomics revealed intraspecies variation, highlighting the strain’s unique genomic features, particularly related to aromatic metabolism, transport, and regulation. Our findings demonstrate the ability of SM-20 to degrade PHE, as evidenced by a significant reduction in its concentration over a 30-day period. Metabolite analysis identified key intermediates, such as 9,10—phenanthrene quinone and hydroxybiphenyl, suggesting the activation of specific catabolic pathways.

Furthermore, comparative proteomic analysis provided insights into the cellular response/metabolism induced in SM-20 cells during PHE exposure. Upregulation of enzymes involved in outer membrane remodeling, transportation, and defense mechanisms indicates the activation of specific stress responses. Moreover, accumulation of enzymes like cytochrome P450 monooxygenase and quinone oxidoreductase suggests their key roles in the biodegradation process. Therefore, integration of these changes with analytical data allowed us to elucidate the degradation pathways of PHE.

This multi-faceted approach represents one of the few studies within the *Pseudomonas* genus to comprehensively analyze the growth and degradation of PHE. It contributes not only to the broader knowledge of microbial diversity and adaptation strategies but also depicts *P. veronii* strain SM-20 as a promising candidate for potential applications in bioremediation and bioaugmentation strategies on polluted sites. Future studies will focus on the analysis of mutants to define the genetic functions essential in PHE metabolism.

## Figures and Tables

**Table 1:** Retention data and electron impact mass spectral characteristics of PHE metabolites.

Product No.	tR [min]	MW	*m*/*z* of Fragment Ions (Relative Intensity in %)	Structural Suggestion
1	13.1	194	194 (22.6), 178 (7.5), 165 (99.9), 139 (4.1), 115 (2.2)	Phenanthrene 9,10-oxide
2	12.49	208	208 (99.9), 180 (82.3), 152 (59.2), 126 (9.8), 76 (19.4)	9,10-Phenanthrene quinone
3	7.06	170	170 (99.9), 141 (31.7), 115 (22.4), 89 (3.8), 63 (4.1)	o-Hydroxybiphenyl
4	6.19	166	148 (26.2), 104 (99.9), 76 (63.9), 50 (38.0)	Phthalic anhydride
5	5.44	152	134 (99.9), 106 (44.4), 78 (90.1), 51 (14.6)	2-Coumaranone

**Table 2:** Protein candidates of *P. veronii* isolate SM-20 involved in the degradation/catabolism of aromatic compounds.

Accession ID	Gene ID	EC Number	Name
Oxidoreductases			
A0A1D3JSA5	peg.2743	1.13.11.5	Homogentisate 1,2-dioxygenase
A0A1D3JXN6	peg.644	1.13.11.27	4-hydroxyphenylpyruvate dioxygenase
A0A1D3K3P0	peg.4964	1.14.12.1	Anthranilate 1,2-dioxygenase small subunit
A0A1D3K3R6	peg.4971	1.13.11.1	Catechol 1,2-dioxygenase
A0A1D3JYJ0	peg.977	1.2.1.85	2-hydroxymuconic semialdehyde dehydrogenase
A0A1D3K906	peg.984,3084,3088	1.13.11.2	CatO2ase
A0A1D3K930	peg.4843	1.14.12.18	Isopropylbenzene dioxygenase iron-sulfur protein small subunit
A0A1D3JYR3	peg.1053	1.14.12.19	3-phenylpropionate dioxygenase
A0A1D3K1V5	peg.2936	1.13.11.39	Extradiol dioxygenase
A0A1D3JYZ4	peg.979	1.14.13.7	Phenol hydroxylase
A0A1D3JYN7	peg.982	1.14.13.7	Phenol hydroxylase P1 protein
A0A1D3JWX5	peg.342	1.3.5.1	Acetoin:2,6-dichlorophenolindophenol oxidoreductase subunit alpha
A0A1D3JX27	peg.343	1.3.5.1	Acetoin:2,6-dichlorophenolindophenol oxidoreductase subunit beta
A0A1D3JRD8	peg.4571	1.14.14.1	Monooxygenase
A0A1D3JTA7		1.13.11.3	Protocatechuate 3, 4-dioxygenase subunit alpha
A0A1D3JSV4	peg.2577	1.18.1.2	Ferredoxin--NADP reductase
A0A1D3JVD3	peg.6254,4787	1.2.1.3	Aldehyde dehydrogenase
A0A1D3K5B8	peg.3524	1.2.1.3	Aldehyde dehydrogenase
A0A1D3JYP8	peg.1022, 3705	1.2.1.3	Aldehyde dehydrogenase PuuC
A0A1D3K973	peg.1047,4821	1.2.1.10	Acetaldehyde dehydrogenase
A0A1D3K0Y9	peg.1742	1.6.5.5	Quinone oxidoreductase
A0A1D3JVV0	peg.6390	7.1.1.-	NADH-quinone oxidoreductase
A0A1D3JVY4	peg. 6394	7.1.1.-	NADH-quinone oxidoreductase subunit B
A0A1D3JVV9	peg.6393	7.1.1.-	NADH-quinone oxidoreductase subunit C/D
A0A1D3JVX6	peg.6391	7.1.1.2	NADH-quinone oxidoreductase subunit F
A0A1D3JW33	peg.6376	1.11.1.19	DyP type peroxidase
A0A1D3JPQ1	peg.3915	1.14.14.1	Cytochrome P450
A0A1D3JYG5	peg.937	1.13.11.24	Quercetin 2,3-dioxygenase
A0A1D3K115	peg.1907	1.13.11.24	Pirin
A0A1D3JZS8	peg.1440	1.13.11.16	2,3-dihydroxyphenyl propionate/2,3-dihydroxicinnamic acid 1,2-dioxygenase
A0A1D3K298	peg.5553	1.1.1.100	3-oxoacyl-[acyl-c arrier-protein] reductase
A0A1D3JWU0	peg.304		Oxidoreductase
A0A1D3K7I1	peg.3568	1.14.11.-	Fe2OG dioxygenase domain-containing protein
Lyases			
A0A1D3K2B2	peg.5665	4.1.3.17	4-hydroxy-4-methyl-2-oxoglutarate aldolase
A0A1D3K4Q4	peg.3296	4.1.3.27	Anthranilate synthase component 1
A0A1D3K4Q5	peg.3295	4.1.3.27	Anthranilate synthase component 2
A0A1D3K908	peg.1046,1445,4823	4.1.3.39	4-hydroxy-2-oxovalerate aldolase
A0A1D3JYJ6	peg.976,3081	4.2.1.132	2-hydroxyhexa-2,4-dienoate hydratase
A0A1D3K0Q9	peg.1455	4.1.2.61	Hydroxycinnamoyl-CoA hydratase-lyase
Isomerases			
A0A1D3JYR4	peg.970	5.3.2.6	2-hydroxymuconate tautomerase
A0A1D3K3L8	peg.4970	5.3.3.4	Muconolactone Delta-isomerase
A0A1D3JX36	peg.428	5.5.1.5	3-carboxymuconate cyclase
Transferases			
A0A1D3K077	peg.1572	2.8.3.6	3-oxoadipate CoA-transferase
A0A1D3K0A2	peg.1573	2.8.3.6	3-oxoadipate CoA-transferase
Hydrolases			
A0A1D3JTI3	peg.2407	3.1.1.24	3-oxoadipate enol-lactonase
A0A1D3JXH6	peg.538	3.1.1.79	Alpha/beta hydrolase

## Data Availability

Data are contained within the article and Supplementary Materials.

## Supplementary Materials

The following supporting information can be downloaded at: https://www.mdpi.com/article/10.3390/microorganisms12040753/s1, Chapter S1: Supplementary Table S1: GenIII microplate composition; Supplementary Table S2: Metabolic clusters and functions involved in aromatic hydrocarbon degradation in *P. veronii* SM-20 from RAST annotation; Supplementary Figure S1: Schematic representation of the gene clusters harboring the gene encoding the catechol 2,3-dioxygenase in *P. veronii* SM-20 and *P. veronii* 1YdBTEX2; Supplementary Figure S2: Schematic representation of the gene clusters harboring the gene encoding the 2,3-hydroxybiphenyl 1,2-dioxygenase in *P. veronii* SM-20 and *P. veronii* 1YdBTEX2; Supplementary Figure S3: Metabolic profile of the strain: (a) Growth curves for every well and (b) heatmap of the activity indexes assigned for each substrate; Supplementary Figure S4: Scatter plot relationships between curve parameters and curve clusterization in *P. veronii* phenomic analysis; Supplementary Figures S5–S9: Intermediates of the phenanthrene depletion by *P. veronii* isolate: Phenanthrene 9,10-oxide (S5); 9,10-Phenanthrenedione (S6); o-Hydroxybiphenyl (S7); Phthalic anhydride (S8); 2-Coumaranone (S9). Chapter S2: Supplementary Table S3: Full list of *P. veronii* proteins identified and quantified using comprehensive reverse phase UHPLC profiling and label-free mass spectrometry. Supplementary Table S4: Annotations of identified and quantified proteins of *P. veronii*. Supplementary Table S5: Annotations of significantly changed (fold > 1.8; ANOVA *p* < 0.05; fitted Tukey’s HSD thresholds) proteins of *P. veronii*. Supplementary Figure S10: Distribution of identified proteins of *P. veronii* among sampling points. Supplementary Figure S11: Heat map of statistically significant upregulated and downregulated proteins of *P. veronii* and their classification based on subcellular localization and clusters of orthologous groups (COGs). Chapter S3: Supplementary Files S1–S3: Genomic data of *P. veronii* SM-20: aa sequence file (S1), GFF file (S2), and DNA contigs file (S3).
