# Supplementary material for: Proteogenomic Characterization of Pseudomonas veronii SM-20 Growing on Phenanthrene as Only Carbon and Energy Source
Source: Microorganisms. 2024 Apr 8;12(4):753. doi: 10.3390/microorganisms12040753 (PMC11052242; doi:10.3390/microorganisms12040753)

Chapter S1

Supplementary materials

Proteogenomic characterization of *Pseudomonas veronii* SM-20 growing on phenanthrene as only carbon and energy source

Sofía G. Zavala-Meneses[*1,2], Andrea Firrincieli[3], Petra Chalova[4,5], Petr Pajer[6], Alice Checcucci[7], Ludovit Skultety [*1,4], Martina Cappelletti[3]

[1] Institute of Microbiology, Czech Academy of Sciences, Videnska 1083, 14220, Prague, Czech Republic
[2] Charles University, Faculty of Science, Vinicna 5, 12844, Prague, Czech Republic
[3] Department of Pharmacy and Biotechnology, University of Bologna, 40126 Bologna, Italy; andrea.firrincieli3@unibo.it or andrea.firrincieli@unitus.it (A.F.); martina.cappelletti2@unibo.it (M.C.)
[4] Biomedical Research Center, Slovak Academy of Sciences, Dubravska c. 9, 845 05 Bratislava, Slovakia; petra.chalova@savba.sk
[5] Faculty of Pharmacy, Comenius University, Odbojarov 10, 832 32 Bratislava, Slovakia
[6] Military Health Institute, Military Medical Agency, U Vojenske nemocnice 1200, 169 02 Prague, Czech Republic; petr.pajer@img.cas.cz
[7] Department of Agriculture, Food, Environment and Forestry, University of Florence, 50100 Firenze, Italy; alice.checcucci@unifi.it

* Correspondence: szavala.meneses@biomed.cas.cz (S.G.Z.-M.); viruludo@savba.sk (L.S.)

Supplementary Table S1. GenIII microplate composition

well_id	chemical	category of metabolic source
A01	Negative control	C-Source, negative control
A02	Dextrin	C-Source, carbohydrate
A03	D-Maltose	C-Source, carbohydrate
A04	D-Trehalose	C-Source, carboxylic acid
A05	D-Cellobiose	C-Source, carboxylic acid
A06	Gentobiose	C-Source, carbohydrate
A07	Sucrose	C-Source, amino acid
A08	D-turanose	C-Source, amino acid
A09	Stachyose	C-Source, amino acid
A10	Positive control	Resistance
A11	pH 6	Resistance
A12	pH 5	Resistance
B01	D-Raffinose	C-Source, amino acid
B02	α--D-Lactose	C-Source, carbohydrate
B03	D-Melibiose	C-Source, carbohydrate
B04	β-Methyl-D-Glucoside	C-Source, carbohydrate
B05	D-salicin	C-Source, carboxylic acid
B06	N-Acetyl-D-Glucosamine	C-Source, carboxylic acid
B07	N-Acetyl-B-D-Mannosamine	C-Source, carbohydrate
B08	N-Acetyl-D-Galactosamine	C-Source, carbohydrate
B09	N-Acetyl Neuroaminic acid	C-Source, carboxylic acid
B10	1% NaCl	Resistance
B11	4% NaCl	Resistance
B12	8% NaCl	Resistance
C01	A-D-Glucose	C-Source, carbohydrate
C02	D-Mannose	C-Source, carboxylic acid
C03	D-Fructose	C-Source, carboxylic acid
C04	D-Galactose	C-Source, carbohydrate
C05	3-Methyl Glucose	C-Source, fatty acid

C06	D-Fucose	C-Source, carbohydrate
C07	L-Fucose	C-Source, carbohydrate
C08	L-Rhamnose	C-Source, carboxylic acid
C09	Inosine	C-Source, carbohydrate
C10	1% Sodium Lactate	Resistance
C11	D-Sorbitol	Resistance
C12	D-Serine	Resistance
D01	D-Sorbitol	C-Source, amino acid
D02	D-Mannitol	C-Source, amino acid
D03	D-Arabitol	C-Source, carboxylic acid
D04	myo-Inositol	C-Source, alcohol
D05	Glycerol	C-Source, fatty acid
D06	D-Glucose-6-PO4	C-Source, carboxylic acid
D07	D-Fructose-6-PO4	C-Source, carboxylic acid
D08	D-Asparic Acid	C-Source, carbohydrate
D09	D-Serine	C-Source, carbohydrate
D10	Troleondomycin	Resistance
D11	Rifamycin SV	Resistance
D12	Minocycline	Resistance
E01	Gelatin	C-Source, amino acid
E02	Glycil-L-Proline	C-Source, carboxylic acid
E03	L-Alanine	C-Source, carbohydrate
E04	L-Arginine	C-Source, carbohydrate
E05	L-Aspartic acid	C-Source, fatty acid
E06	L-Glutamic acid	C-Source, carboxylic acid
E07	L-Histidine	C-Source, carboxylic acid
E08	L-Pyroglutamic acid	C-Source, carbohydrate
E09	L-Serine	C-Source, carbohydrate
E10	Lincomycin	Resistance
E11	Guanidine HCl	Resistance
E12	Niaproof 4	Resistance

F01	Pectin	C-Source, amino acid
F02	D-Galacturonic acid	C-Source, carboxylic acid
F03	L-galactonic acid lactone	C-Source, carbohydrate
F04	D-gluconic acid	C-Source, amino acid
F05	D- glucuronic acid	C-Source, carboxylic acid
F06	Glucuronamide	C-Source, carboxylic acid
F07	Mucic acid	C-Source, carboxylic acid
F08	Quinic acid	C-Source, carboxylic acid
F09	D-Saccharic acid	C-Source, carboxylic acid
F10	Vancomycin	Resistance
F11	Tetrazolium violet	Resistance
F12	Tetrazolium blue	Resistance
G01	p-Hydroxy-phenylacetic acid	C-Source, amino acid
G02	Methyl pyruvate	C-Source, carboxylic acid
G03	D-Lactic acid methyl ester	C-Source, amino acid
G04	L-Lactic acid	C-Source, amino acid
G05	Citric acid	C-Source, amino acid
G06	α--Keto-glutaric acid	C-Source, amino acid
G07	D-Malic acid	C-Source, carboxylic acid
G08	L-Malic acid	C-Source, carbohydrate
G09	Bromo-succinic acid	C-Source, carboxylic acid
G10	Nalidixic acid	Resistance
G11	Lithium chloride	Resistance
G12	Potassium tellurite	Resistance
H01	Tween 40	C-Source, amino acid
H02	G-amino-butyric acid	C-Source, carboxylic acid
H03	α-hydroxy-butyric acid	C-Source, carboxylic acid
H04	β-hydroxy-D-L-Butyric acid	C-Source, amine
H05	α-keto-butyric acid	C-Source, carbohydrate
H06	Acetoacetic acid	C-Source, carbohydrate
H07	Propionic acid	C-Source, amide

H08	Acetic acid	C-Source, carboxylic acid
H09	Formic acid	C-Source, carboxylic acid
H10	Aztreonam	Resistance
H11	Sodium Butyrate	Resistance
H12	Sodium Bromate	Resistance

Supplementary Table S2. Metabolic clusters and functions involved in aromatic hydrocarbon degradation in *Pseudomonas veronii* SM-20 from RAST annotation

Feature ID	Function	Subsystem
fig|76761.49.peg.970	Possible 4-oxalocrotonate tautomerase	
fig|76761.49.peg.971	Putative short-chain dehydrogenase	
fig|76761.49.peg.972	4-hydroxy-2-oxovalerate aldolase (EC 4.1.3.39)	Cinnamic Acid Degradation
fig|76761.49.peg.973	Acetaldehyde dehydrogenase, acetylating, (EC 1.2.1.10) in gene cluster for degradation of phenols, cresols, catechol	
fig|76761.49.peg.974	Oxaloacetate tautomerase (EC:5.3.2.2)	
fig|76761.49.peg.975	4-oxalocrotonate decarboxylase (EC 4.1.1.77)	
fig|76761.49.peg.976	2-hydroxyhexa-2,4-dienoate hydratase (EC 4.2.1.132)	
fig|76761.49.peg.977	Putative 5-carboxymethyl-2-hydroxymuconate semialdehyde dehydrogenase oxidoreductase protein (EC 1.2.1.60)	Aromatic Amino Acid Degradation
fig|76761.49.peg.978	Phenol hydroxylase, FAD- and [2Fe-2S]-containing reductase component dmpp	Phenol Hydroxylase
fig|76761.49.peg.979	Phenol hydroxylase, P4 oxygenase component dmpo (EC 1.14.13.7)	Phenol Hydroxylase
fig|76761.49.peg.983	Phenol hydroxylase, assembly protein dmpk	Phenol Hydroxylase
fig|76761.49.peg.984	Catechol 2,3-dioxygenase (EC 1.13.11.2)	
fig|76761.49.peg.985	Hypothetical protein	
fig|76761.49.peg.986	Positive regulator of phenol hydroxylase	
fig|76761.49.peg.987	Hypothetical protein	
fig|76761.49.peg.988	Exporter protein, RND family	
fig|76761.49.peg.989	BNR repeat protein	
fig|76761.49.peg.990	Hypothetical protein	
fig|76761.49.peg.991	Hypothetical protein	
fig|76761.49.peg.992	Aromatic hydrocarbon utilization transcriptional regulator CatR (LysR family)	DNA-binding regulatory proteins, strays
fig|76761.49.peg.993	Hypothetical protein	
fig|76761.49.peg.1044	Methyl-accepting chemotaxis sensor/transducer protein	
fig|76761.49.peg.1045	2-polyprenylphenol hydroxylase and related flavodoxin oxidoreductases / CDP-6-deoxy-delta-3,4-glucoseen reductase-like	
fig|76761.49.peg.1046	4-hydroxy-2-oxovalerate aldolase (EC 4.1.3.39)	Cinammic Acid Degradation
fig|76761.49.peg.1047	Acetaldehyde dehydrogenase, acetylating, (EC 1.2.1.10) in gene cluster for degradation of phenols, cresols, catechol	
fig|76761.49.peg.1048	Long-chain-fatty-acid--coa ligase (EC 6.2.1.3)	n-phenyl Alkanoic Acid Degraddation
fig|76761.49.peg.1049	Aromatic--coa ligase	
fig|76761.49.peg.1050	2-keto-4-pentenoate hydratase (EC 4.2.1.80)	Cinammic Acid Degradation
fig|76761.49.peg.1051	Putative oxidoreductase, nitronate monooxygenase family	
fig|76761.49.peg.1052	2,3-dihydroxy-2,3-dihydro-phenylpropionate dehydrogenase (EC 1.3.1.87)	
fig|76761.49.peg.1053	3-phenylpropionate dioxygenase, beta subunit (EC 1.14.12.19)	Dioxygenases (EC 1.14.12.-), Aromatic dioxygenase mess, Phenylpropionate, cinamic acid degradation

fig|76761.49.peg.1054	3-phenylpropionate dioxygenase, alpha subunit (EC 1.14.12.19)	Dioxygenases (EC 1.14.12.-), Aromatic dioxygenase mess, Phenylpropionate, cinamic acid degradation
fig|76761.49.peg.1055	3-phenylpropionate dioxygenase ferredoxin subunit	Dioxygenases (EC 1.14.12.-), Aromatic dioxygenase mess, Phenylpropionate, cinamic acid degradation
fig|76761.49.peg.1056	3-carboxyethylcatechol 2,3-dioxygenase (EC 1.13.11.16)	Dioxygenases (EC 1.14.12.-), Aromatic dioxygenase mess, cinamic acid degradation
fig|76761.49.peg.1057	Hypothetical protein	
fig|76761.49.peg.1058	2-hydroxymuconic semialdehyde hydrolase (EC 3.7.1.9)	
fig|76761.49.peg.1059	Transcriptional regulator, acrr family	
fig|76761.49.peg.1060	Hypothetical protein	
fig|76761.49.peg.1061	ABC transporter, permease protein 1 (cluster 4, leucine/isoleucine/valine/benzoate)	
fig|76761.49.peg.1062	ABC transporter, permease protein 2 (cluster 4, leucine/isoleucine/valine/benzoate) / ABC transporter, ATP-binding protein 1 (cluster 4, leucine/isoleucine/valine/benzoate)	
fig|76761.49.peg.1063	Branched-chain amino acid transport ATP-binding protein livf (TC 3.A.1.4.1)	
fig|76761.49.peg.1064	ABC transporter, substrate-binding protein (cluster 4, leucine/isoleucine/valine/benzoate)	
fig|76761.49.peg.1410	Xenobiotic reductase A	
fig|76761.49.peg.1411	Transcriptional regulator, arsr family	
fig|76761.49.peg.1439	3-(3-hydroxyphenyl) propanoate hydroxylase (EC 1.14.13.127)	
fig|76761.49.peg.1440	3-carboxyethylcatechol 2,3-dioxygenase (EC 1.13.11.16)	Dioxygenases (EC 1.14.12.-), Aromatic dioxygenase mess, cinamic acid degradation
fig|76761.49.peg.1441	2-hydroxy-6-oxonona-2,4-dienedioate hydrolase (EC 3.7.1.14)	
fig|76761.49.peg.1442	2-keto-4-pentenoate hydratase (EC 4.2.1.80)	Cinnamic Acid Degradadtion
fig|76761.49.peg.1443	Acetaldehyde dehydrogenase, acetylating, (EC 1.2.1.10) in gene cluster for degradation of phenols, cresols, catechol	
fig|76761.49.peg.1444	Acetaldehyde dehydrogenase, acetylating, (EC 1.2.1.10) in gene cluster for degradation of phenols, cresols, catechol	
fig|76761.49.peg.1445	4-hydroxy-2-oxovalerate aldolase (EC 4.1.3.39)	Cinnamic Acid Degradadtion
fig|76761.49.peg.1446	3-(3-hydroxyphenyl)propionate transporter, MFS-type	
fig|76761.49.peg.1447	Outer membrane low permeability porin, oprd family	
fig|76761.49.peg.1460	Protein involved in meta-pathway of phenol degradation	
fig|76761.49.peg.1462	Putative benzaldehyde dehydrogenase oxidoreductase protein (EC 1.2.1.28)	
fig|76761.49.peg.1673	Protein involved in meta-pathway of phenol degradation	
fig|76761.49.peg.1674	Transcriptional regulator, arac family	

fig|76761.49.peg.1720	FMNH2-dependent alkanesulfonate monooxygenase (EC 1.14.14.5)	
fig|76761.49.peg.1722	Transcriptional regulator clustered with alkanesulfonate monooxygenase	
fig|76761.49.peg.1725	Glyoxalase/bleomycin resistance protein/dioxygenase	
fig|76761.49.peg.1813	Oxidoreductase, short-chain dehydrogenase/reductase family	
fig|76761.49.peg.1814	Aromatic-ring-hydroxylating dioxygenase, beta subunit	Dioxygenases (1.14.12.-), Aromatic dioxygenase mess
fig|76761.49.peg.1815	Ring hydroxylating dioxygenase, alpha subunit/Rieske (2Fe-2S) protein (EC 1.14.12.18)	Dioxygenases (1.14.12.-), Aromatic dioxygenase mess
fig|76761.49.peg.1821	ABC transporter, substrate-binding protein (cluster 4, leucine/isoleucine/valine/benzoate)	
fig|76761.49.peg.1822	ABC transporter, permease protein 1 (cluster 4, leucine/isoleucine/valine/benzoate)	
fig|76761.49.peg.1823	ABC transporter, permease protein 2 (cluster 4, leucine/isoleucine/valine/benzoate) / ABC transporter, ATP-binding protein 1 (cluster 4, leucine/isoleucine/valine/benzoate)	
fig|76761.49.peg.1824	ABC transporter, ATP-binding protein 2 (cluster 4, leucine/isoleucine/valine/benzoate)	
fig|76761.49.peg.1830	Protein involved in meta-pathway of phenol degradation	
fig|76761.49.peg.1831	Phenylacetaldehyde dehydrogenase (EC 1.2.1.39)	Aromatic Aminoacid Catabolism
fig|76761.49.peg.1832	Transcriptional regulator, arac family	
fig|76761.49.peg.1833	Putative ABC transporter, substrate binding protein	
fig|76761.49.peg.1834	Hypothetical protein	
fig|76761.49.peg.1835	Transcriptional regulator, marr family	
fig|76761.49.peg.1836	Phage-related integrase	
fig|76761.49.peg.1837	Protocatechuate 3,4-dioxygenase alpha chain (EC 1.13.11.3)	
fig|76761.49.peg.1838	Protocatechuate 3,4-dioxygenase beta chain (EC 1.13.11.3)	Protocatechuate branch of beta-ketoadipate pathway
fig|76761.49.peg.1839	Pca operon transcriptional activator pcaq	Protocatechuate branch of beta-ketoadipate pathway
fig|76761.49.peg.2406	4-carboxymuconolactone decarboxylase (EC 4.1.1.44)	
fig|76761.49.peg.2407	Beta-ketoadipate enol-lactone hydrolase (EC 3.1.1.24)	Protocatechuate branch of beta-ketoadipate pathway
fig|76761.49.peg.2408	3-carboxy-cis,cis-muconate cycloisomerase (EC 5.5.1.2)	Protocatechuate branch of beta-ketoadipate pathway
fig|76761.49.peg.2409	Dicarboxylic acid transporter pcat	Protocatechuate branch of beta-ketoadipate pathway
fig|76761.49.peg.2410	Protocatechuate 3,4-dioxygenase alpha chain (EC 1.13.11.3)	Protocatechuate branch of beta-ketoadipate pathway
fig|76761.49.peg.2411	Protocatechuate 3,4-dioxygenase beta chain (EC 1.13.11.3)	Protocatechuate branch of beta-ketoadipate pathway
fig|76761.49.peg.2412	3-oxoadipyl-coa thiolase (EC 2.3.1.174)	Protocatechuate branch of beta-ketoadipate pathway
fig|76761.49.peg.2413	3-oxoadipate coa-transferase subunit B (EC 2.8.3.6)	
fig|76761.49.peg.2414	3-oxoadipate coa-transferase subunit A (EC 2.8.3.6)	Catechol branch of beta-ketoadipate pathway, Protocatechuate branch of beta-ketoadipate pathway

fig|76761.49.peg.2415	Hypothetical protein	Catechol branch of beta-ketoadipate pathway, Protocatechuate branch of beta-ketoadipate pathway
fig|76761.49.peg.2416	4-hydroxybenzoate transporter	
fig|76761.49.peg.2417	Pca regulon regulatory protein pcar	Cinnamic Acid Degradation, Gentisate degradation, p-Hydroxybenzoate degradation
fig|76761.49.peg.2418	Hypothetical protein	Cinnamic Acid Degradation, Gentisate degradation, p-Hydroxybenzoate degradation
fig|76761.49.peg.2742	Fumarylacetoacetase (EC 3.7.1.2)	Homogentisate pathway of aromatic compound degradation
fig|76761.49.peg.2743	Homogentisate 1,2-dioxygenase (EC 1.13.11.5)	Homogentisate pathway of aromatic compound degradation
fig|76761.49.peg.2744	Homogentisate pathway transcriptional regulator hmgr	
fig|76761.49.peg.3077	4-oxalocrotonate tautomerase (EC 5.3.2.-)	
fig|76761.49.peg.3078	4-oxalocrotonate decarboxylase (EC 4.1.1.77)	
fig|76761.49.peg.3079	4-hydroxy-2-oxovalerate aldolase (EC 4.1.3.39)	Cinnamic Acid Degradation
fig|76761.49.peg.3080	Acetaldehyde dehydrogenase, acetylating, (EC 1.2.1.10) in gene cluster for degradation of phenols, cresols, catechol	
fig|76761.49.peg.3081	2-hydroxyhexa-2,4-dienoate hydratase (EC 4.2.1.132)	
fig|76761.49.peg.3082	2-hydroxymuconic semialdehyde hydrolase (EC 3.7.1.9)	
fig|76761.49.peg.3083	Putative 5-carboxymethyl-2-hydroxymuconate semialdehyde dehydrogenase oxidoreductase protein (EC 1.2.1.60)	Aromatic Amino Acid Degraddation
fig|76761.49.peg.3084	Catechol 2,3-dioxygenase (EC 1.13.11.2)	
fig|76761.49.peg.3085	Positive regulator of phenol hydroxylase, dmpr	Phenol Hydroxylase
fig|76761.49.peg.3086	Hypothetical protein	
fig|76761.49.peg.3087	Hypothetical protein	
fig|76761.49.peg.3088	Catechol 2,3-dioxygenase (EC 1.13.11.2)	
fig|76761.49.peg.3089	Benzoate 1,2-dioxygenase alpha subunit (EC 1.14.12.10)	Dioxygenases (EC 1.14.12.-), Benzoate degradataion, Aromatic dioxygenase mess
fig|76761.49.peg.3090	Benzoate 1,2-dioxygenase beta subunit (EC 1.14.12.10)	Dioxygenases (EC 1.14.12.-), Benzoate degradataion, Aromatic dioxygenase mess
fig|76761.49.peg.3091	Hypothetical protein	
fig|76761.49.peg.3092	Putative benzaldehyde dehydrogenase oxidoreductase protein (EC 1.2.1.28)	
fig|76761.49.peg.3294	Anthranilate phosphoribosyltransferase (EC 2.4.2.18)	3-hydroxyanthranilate, PAPA antibiotics
fig|76761.49.peg.3295	Anthranilate synthase, amidotransferase component (EC 4.1.3.27) @ Para-aminobenzoate synthase, amidotransferase component (EC 2.6.1.85)	3-hydroxyanthranilate, PAPA antibiotics
fig|76761.49.peg.3296	Anthranilate synthase, aminase component (EC 4.1.3.27)	3-hydroxyanthranilate, PAPA antibiotics
fig|76761.49.peg.3627	2-polyprenylphenol hydroxylase and related flavodoxin oxidoreductases / CDP-6-deoxy-delta-3,4-glucoseen reductase-like	
fig|76761.49.peg.3628	3-polyprenyl-4-hydroxybenzoate carboxy-lyase	

	(EC 4.1.1.98)	
fig|76761.49.peg.3938	Quinone oxidoreductase (EC 1.6.5.5)	
fig|76761.49.peg.3939	Coproporphyrinogen III oxidase, aerobic (EC 1.3.3.3)	
fig|76761.49.peg.3940	Shikimate 5-dehydrogenase I alpha (EC 1.1.1.25)	
fig|76761.49.peg.3941	Sulfate permease	
fig|76761.49.peg.4762	4-hydroxyphenylpyruvate dioxygenase (EC 1.13.11.27)	Homogentisate pathway of aromatic compound degradation, Aromatic Amino Acid degraddation
fig|76761.49.peg.4763	Transcriptional regulator, acrr family	
fig|76761.49.peg.4764	Quinate/shikimate 5-dehydrogenase I delta (EC 1.1.1.25)	Common pathway for Synthesis of Aromatic Compounds
fig|76761.49.peg.4765	3-dehydroquinate dehydratase II (EC 4.2.1.10)	Common pathway for Synthesis of Aromatic Compounds, Quinate degradation
fig|76761.49.peg.4820	2-keto-4-pentenoate hydratase (EC 4.2.1.80)	Cinnamic Acid Degradation
fig|76761.49.peg.4821	Acetaldehyde dehydrogenase, acetylating, (EC 1.2.1.10) in gene cluster for degradation of phenols, cresols, catechol	
fig|76761.49.peg.4822	4-hydroxy-2-oxovalerate aldolase (EC 4.1.3.39)	Cinnamic Acid Degradation
fig|76761.49.peg.4823	Hypothetical protein	
fig|76761.49.peg.4830	Transcriptional regulator, arac family	
fig|76761.49.peg.4831	Rieske (2Fe-2S) domain protein	
fig|76761.49.peg.4834	Putative arac-family transcriptional regulator	
fig|76761.49.peg.4835	Putative oxygenase subunit	
fig|76761.49.peg.4836	Hypothetical protein	
fig|76761.49.peg.4837	Hypothetical protein	
fig|76761.49.peg.4838	2-polyprenylphenol hydroxylase and related flavodoxin oxidoreductases	
fig|76761.49.peg.4839	Uncharacterized deacetylase	
fig|76761.49.peg.4840	Hydantoin racemase (EC 5.1.99.5)	
fig|76761.49.peg.4842	Oxidoreductase, short-chain dehydrogenase/reductase family	
fig|76761.49.peg.4843	2,3-dihydroxybiphenyl 1,2-dioxygenase	
fig|76761.49.peg.4844	2-hydroxymuconic semialdehyde hydrolase (EC 3.7.1.9)	
fig|76761.49.peg.4961	Tryptophan 2,3-dioxygenase (EC 1.13.11.11)	Aromatic Amino Acid Degradation
fig|76761.49.peg.4962	Aromatic amino acid transport protein arop	Aromatic Amino Acid Degradation
fig|76761.49.peg.4963	Benzoate dioxygenase, ferredoxin reductase component; Anthranilate dioxygenase reductase	Dioxygenases (EC 1.14.12.-), Aromatic dioxygenase mess, Aromatic Amino Acid Degradation
fig|76761.49.peg.4964	Benzoate 1,2-dioxygenase beta subunit (EC 1.14.12.10)	Dioxygenases (EC 1.14.12.-), Benzoate degradataion, Aromatic dioxygenase mess
fig|76761.49.peg.4965	Benzoate 1,2-dioxygenase alpha subunit (EC 1.14.12.10)	Dioxygenases (EC 1.14.12.-), Benzoate degradataion, Aromatic dioxygenase mess

fig|76761.49.peg.4966	Hypothetical protein	
fig|76761.49.peg.4967	Benabc operon transcriptional activator benr	Benzoate degradation
fig|76761.49.peg.4968	Aromatic hydrocarbon utilization transcriptional regulator catr (lysr family)	DNA-binding regulatory proteins
fig|76761.49.peg.4969	Muconate cycloisomerase (EC 5.5.1.1)	Catechol branch of beta-ketoadipate pathway, Muconate lactonizing enzyme family
fig|76761.49.peg.4970	Muconolactone isomerase (EC 5.3.3.4)	Catechol branch of beta-ketoadipate pathway
fig|76761.49.peg.4971	Catechol 1,2-dioxygenase (EC 1.13.11.1)	Catechol branch of beta-ketoadipate pathway
fig|76761.49.peg.4972	Transcriptional regulator, asnc family	
fig|76761.49.peg.4973	Kynureninase (EC 3.7.1.3)	NAD and NADP cofactor biosynthesis global
fig|76761.49.peg.4974	Transcriptional regulator, lysr family	
fig|76761.49.peg.4975	Putative membrane protein	
fig|76761.49.peg.6134	Putative diheme cytochrome c-553	Soluble cytochromes and functionally related electron carriers
fig|76761.49.peg.6135	Transcriptional regulator, lysr family	
fig|76761.49.peg.6136	2-hydroxychromene-2-carboxylate isomerase/dsba-like thioredoxin domain	
fig|76761.49.peg.6137	NADH:flavin oxidoreductases, Old Yellow Enzyme family	
fig|76761.49.peg.6138	2-polyprenyl-6-methoxyphenol hydroxylase and related FAD-dependent oxidoreductases	
fig|76761.49.peg.6139	Hypothetical protein	
fig|76761.49.peg.6140	2-amino-3-carboxymuconate-6-semialdehyde decarboxylase (EC 4.1.1.45)	
fig|76761.49.peg.6141	Transcriptional regulator, lysr family	
fig|76761.49.peg.412	Alkane-1 monooxygenase (EC 1.14.15.3)	
fig|76761.49.peg.842	Cyclohexanone monooxygenase (EC 1.14.13.22)	
fig|76761.49.peg.1070	L-lysine 6-monooxygenase [NADPH] (EC 1.14.13.59), aerobactin biosynthesis protein iucd @ Siderophore biosynthesis protein, monooxygenase	
fig|76761.49.peg.1305	Putative monooxygenase PA5393	
fig|76761.49.peg.1370	Nitrilotriacetate monooxygenase component A (EC 1.14.13.-)	
fig|76761.49.peg.1551	Tryptophan 2-monooxygenase (EC 1.13.12.3)	Aromatic Amino Acid degradation
fig|76761.49.peg.1773	Ammonia monooxygenase	
fig|76761.49.peg.2356	Flavin-dependent monooxygenase arso associated with arsenic resistance	
fig|76761.49.peg.3284	Putative oxidoreductase, nitronate monooxygenase family	
fig|76761.49.peg.621	Alpha-ketoglutarate-dependent dioxygenase alkb (EC 1.14.11.33)	
fig|76761.49.peg.644	4-hydroxyphenylpyruvate dioxygenase (EC 1.13.11.27)	Aromatic amino acid degradation, homogentisate pathway of aromatic compound degradation , pterin carbinolamine dehydratase
fig|76761.49.peg.1104	Fe(2+)/alpha-ketoglutarate-dependent dioxygenase lpxo	

fig|76761.49.peg.1482	Glyoxalase/bleomycin resistance protein/dioxygenase	
fig|76761.49.peg.5025	Flavohemoglobin / Nitric oxide dioxygenase (EC 1.14.12.17)	
fig|76761.49.peg.5955	1,2-dihydroxy-3-keto-5-methylthiopentene dioxygenase (EC 1.13.11.54)	
fig|76761.49.peg.338	4-carboxymuconolactone decarboxylase domain/alkylhydroperoxidase ahpd family core domain protein	
fig|76761.49.peg.428	3-carboxymuconate cyclase	
fig|76761.49.peg.514	3-demethylubiquinone-9 3-methyltransferase	
fig|76761.49.peg.688	Putative cytochrome P450 hydroxylase	
fig|76761.49.peg.818	2,3-dihydroxybenzoate-AMP ligase (EC 2.7.7.58) [pyochelin] siderophore @ 2,3-dihydroxybenzoate-AMP ligase (EC 2.7.7.58) of siderophore biosynthesis	Siderophore pyochelin
fig|76761.49.peg.1278	Transcriptional regulator, iclr family	Homogentisate pathway of aromatic compound degradation
fig|76761.49.peg.1362	Cysteine dioxygenase (EC 1.13.11.20)	Aromatic dioxygenase mess, Dioxygenases (EC. 1.13.11.-)
fig|76761.49.peg.1541	2-keto-3-deoxy-D-arabino-heptulosonate-7-phosphate synthase I alpha (EC 2.5.1.54)	Chorismate synthesis
fig|76761.49.peg.1594	Shikimate 5-dehydrogenase I gamma (EC 1.1.1.25)	Chorismate synthesis
fig|76761.49.peg.2002	2-keto-3-deoxy-D-arabino-heptulosonate-7-phosphate synthase II (EC 2.5.1.54)	Chorismate synthesis
fig|76761.49.peg.2097	3-demethylubiquinol 3-O-methyltransferase (EC 2.1.1.64) @ 2-polyprenyl-6-hydroxyphenyl methylase (EC 2.1.1.222)	
fig|76761.49.peg.2248	P-hydroxybenzoate hydroxylase (EC 1.14.13.2)	P-hydroxybenzoate degradation
fig|76761.49.peg.2258	Transcriptional regulator, lysr family	
fig|76761.49.peg.2259	Probable 5-carboxymethyl-2-hydroxymuconate delta isomerase	Aromatic amino acid degradation
fig|76761.49.peg.2904	4-carboxymuconolactone decarboxylase (EC 4.1.1.44)	Protochatechuate branch of beta-ketoadipate pathway
fig|76761.49.peg.3405	Predicted hydrocarbon binding protein (contains V4R domain)	
fig|76761.49.peg.3562	Fumarylacetoacetate hydrolase family protein	Gentisate degradation
fig|76761.49.peg.3611	2-polyprenylphenol hydroxylase	
fig|76761.49.peg.3612	2-polyprenyl-6-methoxyphenol hydroxylase	
fig|76761.49.peg.3684	Benzoate transport protein	Benzoate degradation
fig|76761.49.peg.3769	4-hydroxybenzoate polyprenyltransferase (EC 2.5.1.39)	
fig|76761.49.peg.4167	Uncharacterized protein, possibly involved in aromatic compounds catabolism	
fig|76761.49.peg.4183	Putative 4-hydroxybenzoyl-coa thioesterase	Gentisate degradation
fig|76761.49.peg.4339	Shikimate kinase I (EC 2.7.1.71)	Chorysmate synthesis, common pathway for synthesis of aromatic compounds)
fig|76761.49.peg.4340	3-dehydroquinate synthase (EC 4.2.3.4)	Chorysmate synthesis, common pathway for synthesis of aromatic compounds)
fig|76761.49.peg.4545	3-dehydroquinate dehydratase II (EC 4.2.1.10)	
fig|76761.49.peg.4847	Inner membrane component of tripartite multidrug resistance system	
fig|76761.49.peg.5641	Para-aminobenzoate synthase, aminase component (EC 2.6.1.85)	Chorysmate biosynthesis, 3-hydroxyanthranilate, triptophan biosynthesis
fig|76761.49.peg.6060	Biosynthetic Aromatic amino acid aminotransferase alpha (EC 2.6.1.57) @ Aromatic-amino-acid aminotransferase (EC 2.6.1.57)	Homogentisate pathway of aromatic compound degradation, phenylalanine and

		tyrsine branches from chorismate, pterin carbinolamine dehydratase
fig|76761.49.peg.6367	ABC transporter, ATP-binding protein 2 (cluster 4, leucine/isoleucine/valine/benzoate)	

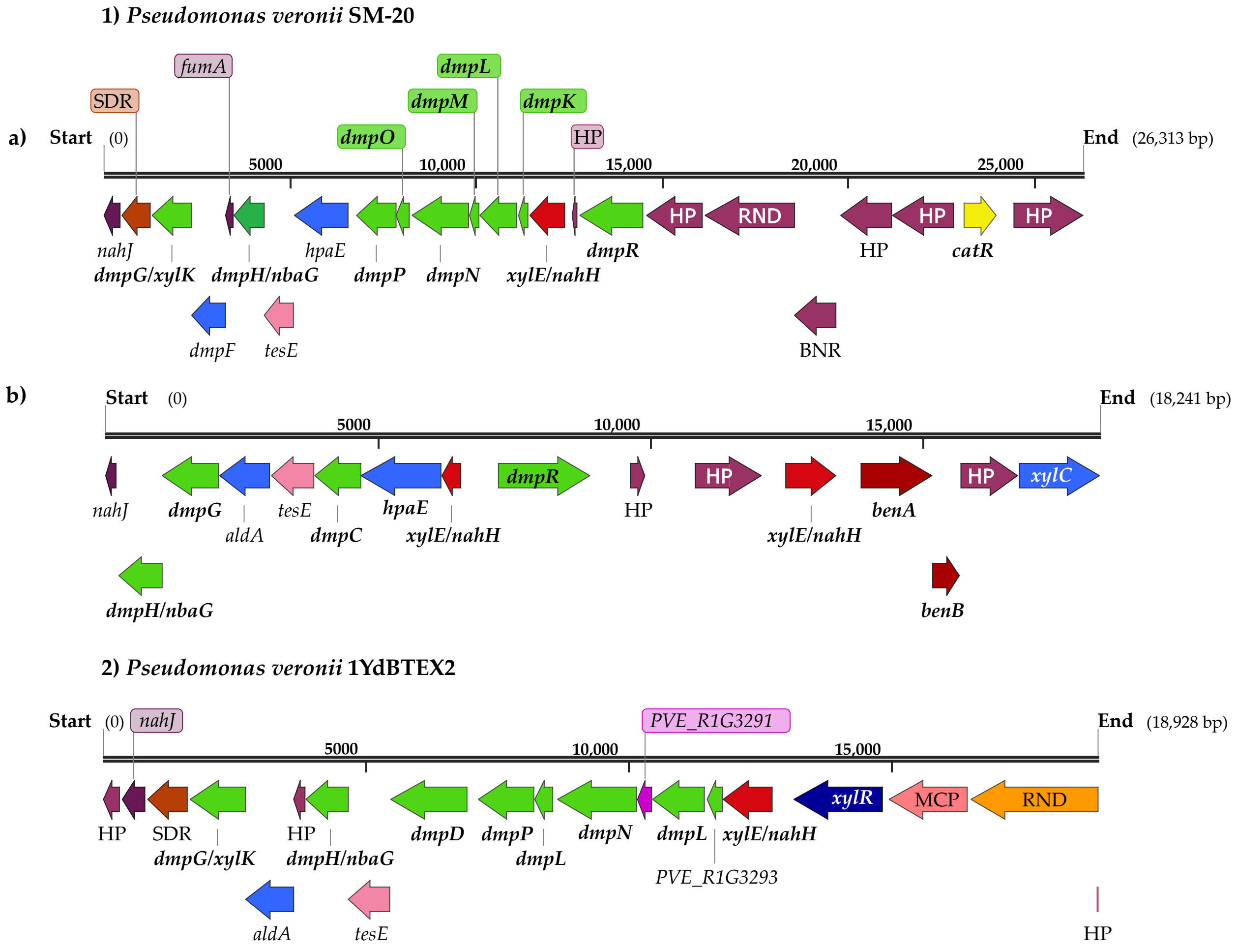

Supplementary Figure S1. Schematic representation of the gene clusters harboring the gene encoding the catechol 2,3-dioxygenase in *Pseudomonas veronii* SM-20 and *P. veronii* 1YdBTEX2. ***nahJ***-4-oxalocrotonate tautomerase (EC 5.3.2.6); **SDR**-short chain dehydrogenase; ***dmpG/xylK***- 4-hydroxy-2-oxovalerate aldolase (EC 4.1.3.39); **HP**- hypothetical protein; ***dmpF/aldA***-acetaldehyde dehydrogenase (EC 1.2.1.10); ***fumA***-oxaloacetate tautomerase (EC5.3.2.2); ***dmpH/nbaG***- 4-oxalocrotonate decarboxylase (EC 4.1.1.77); ***tesE***-2-hydroxyhexa-2,4-dienoate hydratase (EC 4.2.1.132); ***dmpC***-2-hydroxymuconic semialdehyde hydrolase (EC 3.7.1.9); ***dmpD***-2-hydroxymuconic semialdehyde dehydrogenase (EC 1.2.1.85); ***hpaE***- 5-carboxymethyl-2-hydroxymuconate semialdehyde dehydrogenase oxidoreductase protein (EC1.2.1.60); ***dmpP*** - phenol hydroxylase P5 protein (EC 1.14.13.7); ***dmpO***-phenol hydroxylase P4 oxygenase component (EC 1.14.13.7); ***dmpN***-phenol hydroxylase P3 oxygenase component (EC 1.14.13.7); ***dmpM*** -phenol hydroxylase P2 oxygenase component (EC 1.14.13.7); ***dmpL*** - phenol hydroxylase P1 oxygenase component (EC 1.14.13.7); ***dmpK***-phenol hydroxylase protein (EC 1.14.13.7); ***xylE/ nahH*** -catechol 2,3-dioxygenase (EC 1.13.11.2); ***dmpR***-positive regulator of phenol hydroxylase; **RND**-Exporter protein RND family; **BNR**- BNR repeat protein; ***catR***-Aromatic hydrocarbon utilization transcriptional regulator (LysR family); ***benA***- benzoate 1,2-dioxygenase alpha subunit (EC 1.14.12.10); ***benB***- benzoate 1,2-dioxygenase beta subunit; ***xylC***- benzaldehyde dehydrogenase oxidoreductase protein (EC 1.2.1.28); ***PVE_R1G3291***- monooxygenase; ***PVE_R1G3293***-multi-component phenol hydroxylase; ***xylR***-Transcriptional regulatory protein; **MCP**- methyl-accepting chemotaxis protein; **RND**-RND transporter.

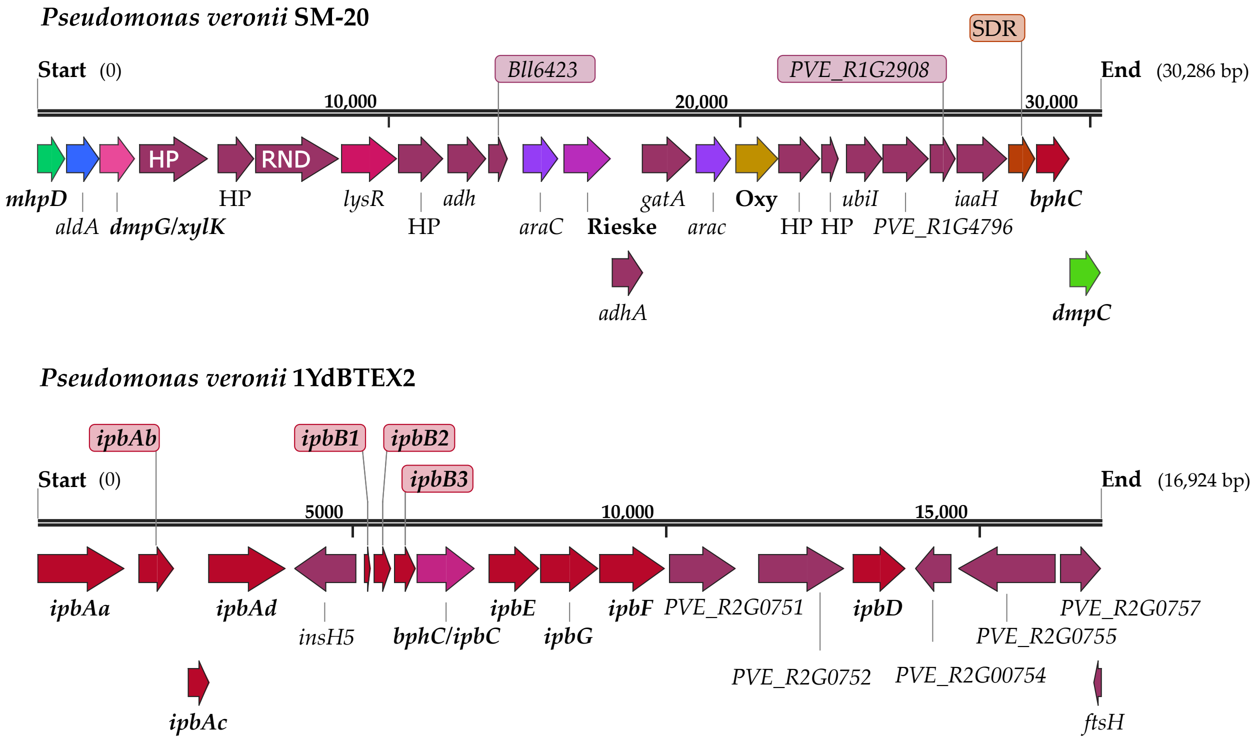

Supplementary Figure S2. Schematic representation of the gene clusters harboring the gene encoding the 2,3-hydroxybihenyl 1,2-dioxygenase in *Pseudomonas veronii* SM-20 and *P. veronii* 1YdBTEX2. ***mhpD***-2-keto-4-pentenoate hydratase (EC 4.2.1.80); ***aldA***- acetaldehyde dehydrogenase (EC 1.2.1.10); ***dmpG/xylK***-4-hydroxy-2-oxovalerate aldolase (EC 4.1.3.39); **HP**-hypothetical protein; **RND**-exporter protein, RND family; ***lysR***- regulatory protein LysR; ***adh***- Zn-containing alcohol dehydrogenase superfamily; ***BII6423***- BII6423 protein; ***araC***-transcriptional regulator, AraC family; ***Rieske/PVE_R1G5552***-Rieske (2Fe-2S) domain protein; ***adhA***- hydrolase, alpha/beta fold family; ***gatA***- glutamyl-tRNA (Gln) amidotransferase subunit A-like protein (EC 3.5.-); **Oxy**-oxygenase (EC 1.13.-/1.14.-); ***ubiI***-2-polyprenylphenol hydroxylase (EC 1.14.13.240); ***PVE_R1G4796***- uncharacterized deacetylase; ***PVE_G2908*** - hydantoin racemase (EC 5.1.99.5); ***iaaH***- indoleacetamide hydrolase (EC 3.5.1.-); **SDR**- short-chain dehydrogenase/reductase; ***ipbC/bphC***-2,3-dihydroxybiphenyl 1,2-dioxygenase (EC1.13.11.39); ***dmpC***- 2-hydroxymuconic semialdehyde dehydrolase (EC 3.7.1.9); ***ipbAa***- isopropylbenzene dioxygenase, iron-sulfur protein, large subunit; ***ipbAb***- isopropylbenzene dioxygenase, iron-sulfur protein, small subunit; ***ipbAc***- isopropylbenzene dioxygenase, ferredoxin subunit; ***ipbAd***- isopropylbenzene dioxygenase, ferredoxin reductase subunit; ***PVE_R2G0743/insH5***-transposase; ***ipbB1***- cis-2,3-dihydroxy-2,3-dihydroisopropylbenzene dehydrogenase; ***ipbB2***- cis-2,3-dihydroxy-2,3-dihydroisopropylbenzene dehydrogenase; ***ipbB3***- cis-2,3-cis-2,3-dihydroxy-2, 3-dihydroisopropylbenzene dehydrogenase-2,3-diol dehydrogenase; ***ipbE***- 2-hydroxypenta-2,4-dienoate hydratase (EC 4.2.1.132); ***ipbG***-Acetaldehyde dehydrogenase (EC1.2.1.10); ***ipbF*** -4-hydroxy-2-oxovalerate aldolase (EC 4.1.3.39); ***PVE_R2G0751/ PVE_R2G0754***- endonuclease DDE; ***PVE_R2G0752***- outer membrane protein; ***ipbD***- 2-hydroxy-6-oxo-7-methylocta-2,4-dienoate hydrolase; ***PVE_R2G0755***- transposase for insertion sequence element IS1353; ***PVE_R2G0757***- integrase; ***PVE_R2G0756/ftsH***-ATP-dependent Zn protease.

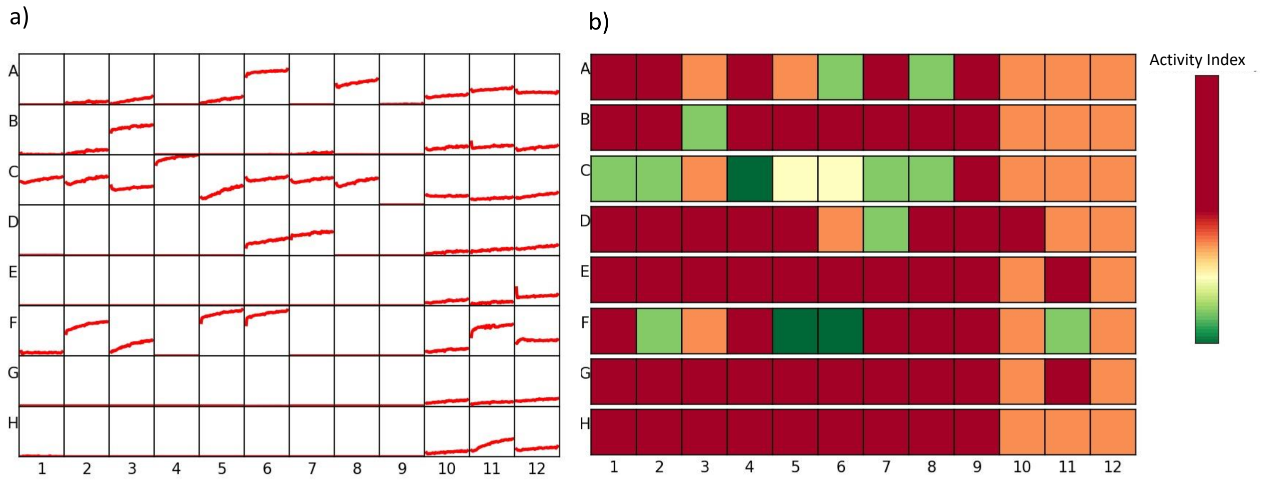

Figure S3. Metabolic profile of the strain: (a) Growth curves for every well and (b) heatmap of the activity indexes assigned for each substrate.

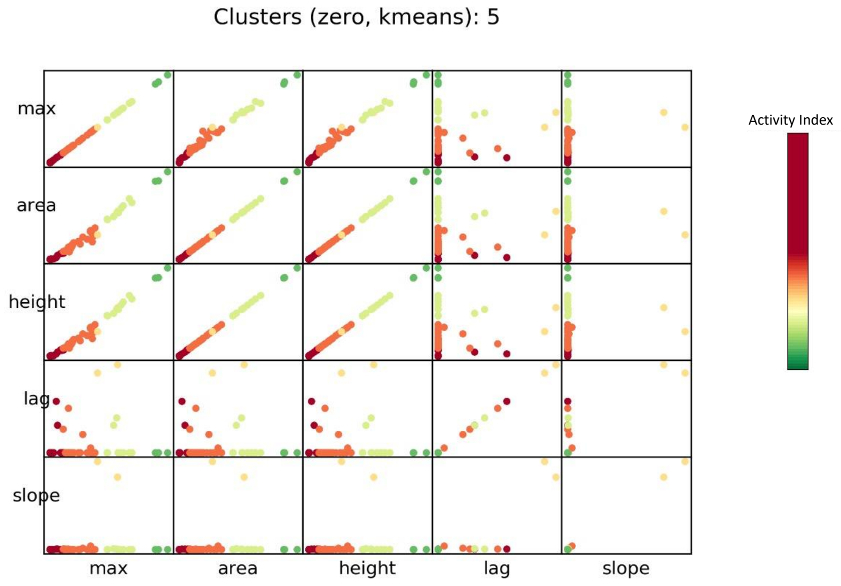

Figure S4. Scatter plot relationships between curve parameters and curve clusterization in *P. veronii* phenomic analysis. Each dot represents distinct curves and the colors referred to the assigned activity index. The clusters are ordered by the area parameter.

Supplementary Figure S5. Intermediates of the phenanthrene depletion by *P. veronii* isolate SM-20: **Phenanthrene 9,10-oxide**

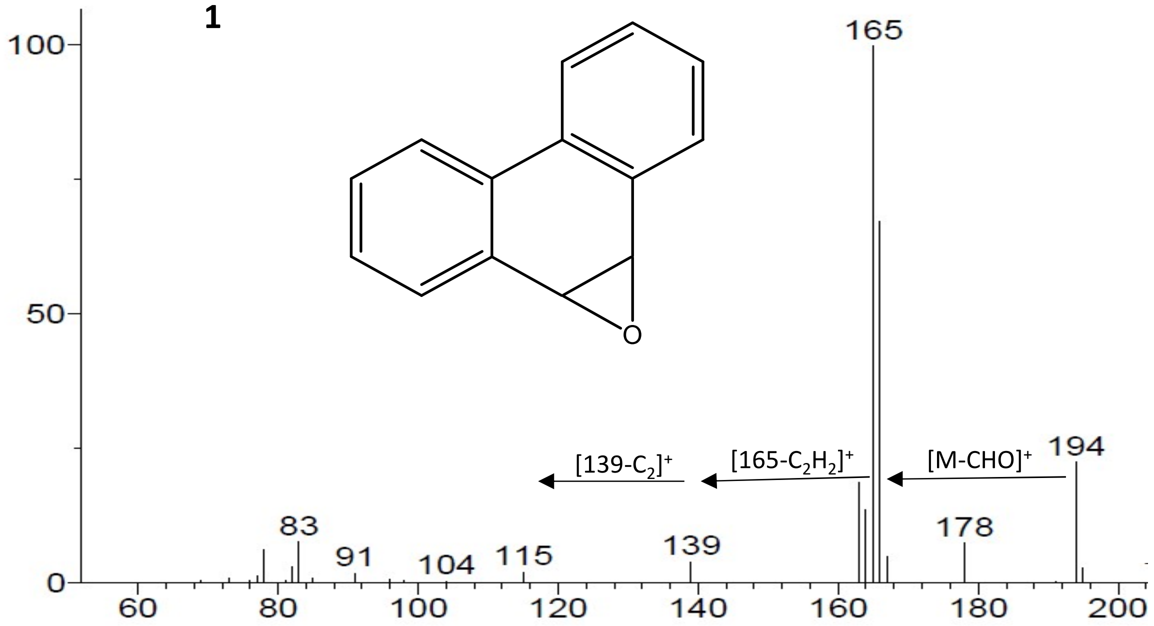

Supplementary Figure S6. Intermediates of the phenanthrene depletion by *P. veronii* isolate SM-20: **9,10-Phenanthrenedione**

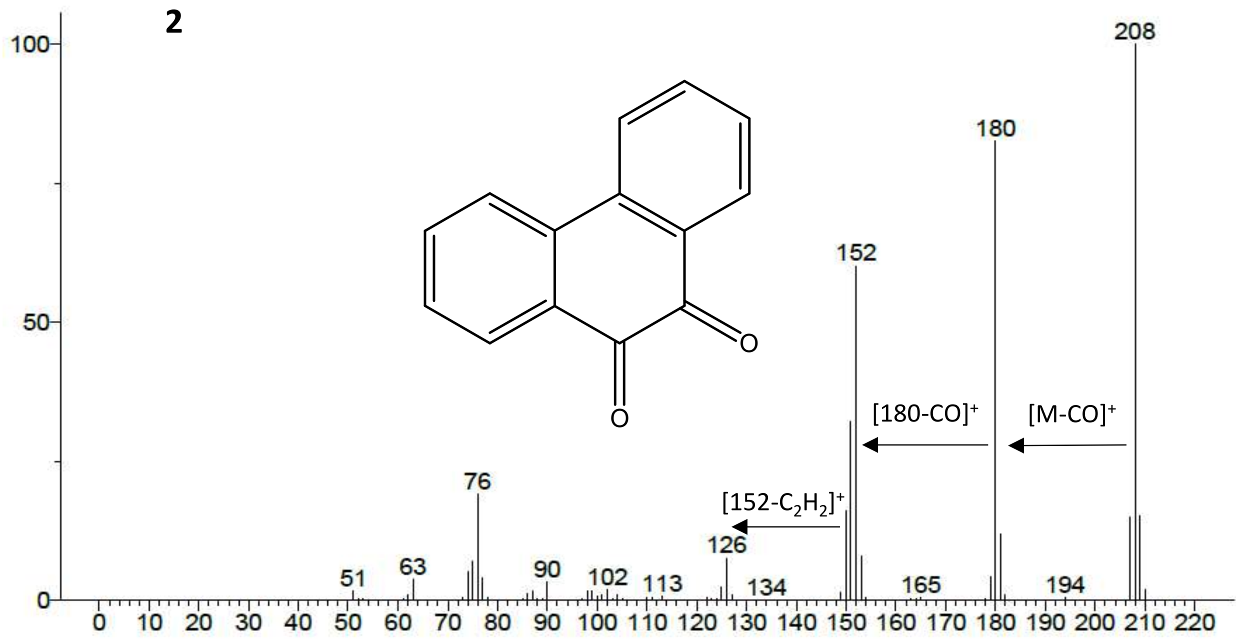

Supplementary Figure S7. Intermediates of the phenanthrene depletion by *P. veronii* isolate SM-20: **o-Hydroxybiphenyl**

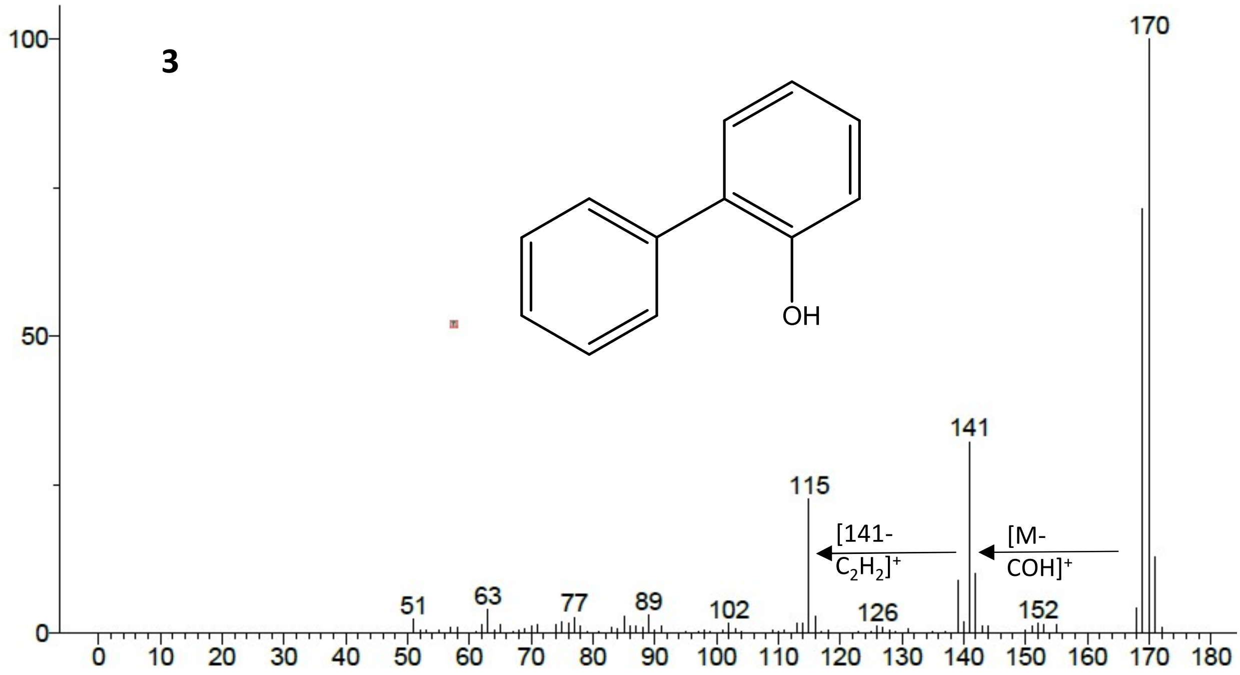

Supplementary Figure S8. Intermediates of the phenanthrene depletion by *P. veronii* isolate SM-20: **Phthalic anhydride**

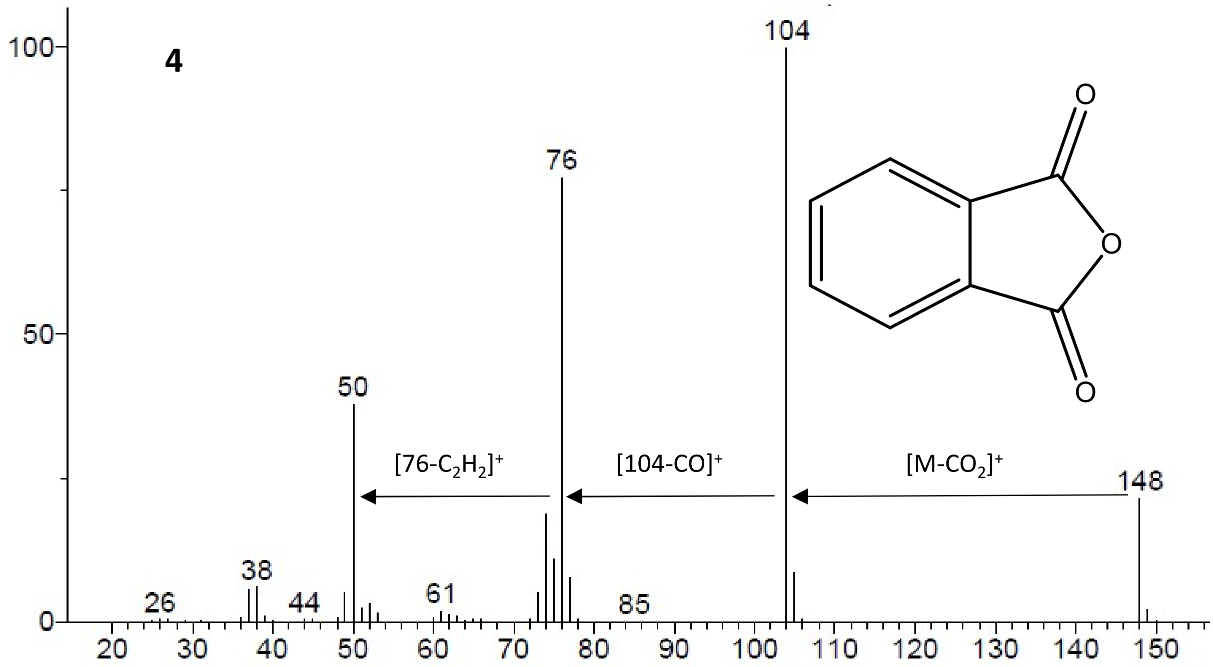

Supplementary Figure S9. Intermediates of the phenanthrene depletion by *P. veronii* isolate SM-20: **2-Coumaranone**

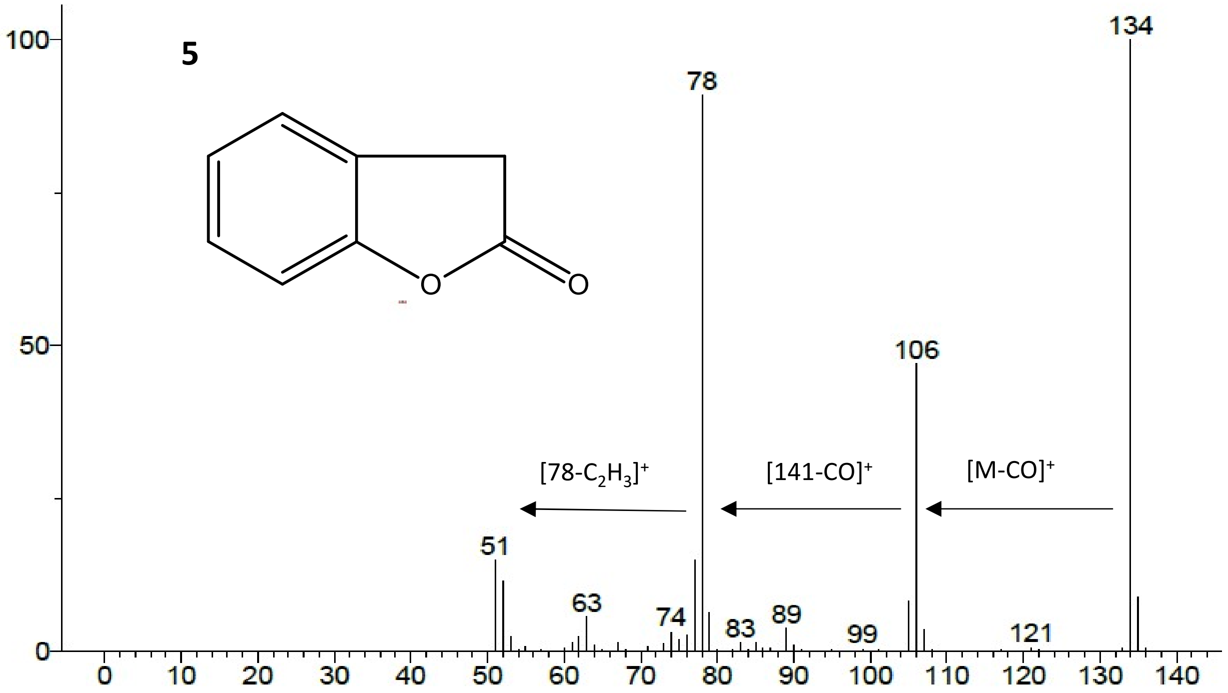

Supplement: Supplementary file 1 [file microorganisms-12-00753-s001.zip › Supplementary Materials_Chapter S1.pdf]
