# Supplementary material for: Proteogenomic Characterization of Pseudomonas veronii SM-20 Growing on Phenanthrene as Only Carbon and Energy Source
Source: Microorganisms. 2024 Apr 8;12(4):753. doi: 10.3390/microorganisms12040753 (PMC11052242; doi:10.3390/microorganisms12040753)
Supplement: Supplementary file 1 [file microorganisms-12-00753-s001.zip › Supplementary Materials_Chapter S2.pdf]

Chapter S2

Supplementary materials

Proteogenomic characterization of *Pseudomonas veronii* SM-20 growing on phenanthrene as only carbon and energy source

Sofía G. Zavala-Meneses[*1,2], Andrea Firrincieli[3], Petra Chalova[4,5], Petr Pajer[6], Alice Checcucci[7], Ludovit Skultety [*1,4], Martina Cappelletti[3]

[1] Institute of Microbiology, Czech Academy of Sciences, Videnska 1083, 14220, Prague, Czech Republic
[2] Charles University, Faculty of Science, Vinicna 5, 12844, Prague, Czech Republic
[3] Department of Pharmacy and Biotechnology, University of Bologna, 40126 Bologna, Italy; andrea.firrincieli3@unibo.it or andrea.firrincieli@unitus.it (A.F.); martina.cappelletti2@unibo.it (M.C.)
[4] Biomedical Research Center, Slovak Academy of Sciences, Dubravska c. 9, 845 05 Bratislava, Slovakia; petra.chalova@savba.sk
[5] Faculty of Pharmacy, Comenius University, Odbojarov 10, 832 32 Bratislava, Slovakia
[6] Military Health Institute, Military Medical Agency, U Vojenske nemocnice 1200, 169 02 Prague, Czech Republic; petr.pajer@img.cas.cz
[7] Department of Agriculture, Food, Environment and Forestry, University of Florence, 50100 Firenze, Italy; alice.checcucci@unifi.it

* Correspondence: szavala.meneses@biomed.cas.cz (S.G.Z.-M.); viruludo@savba.sk (L.S.)

Supplementary Table S3. Full list of *P. veronii* proteins identified and quantified using comprehensive reverse phase UHPLC profiling and label-free mass spectrometry. It shows identification details, quantitative measures, and statistic scores. ANOVA and Tukey´s HSD that satisfied the thresholds are highlighted in green. Log2 transformed ratios of absolute fold change above 1.8 and Accessions, which fitted the Tukey´s HSD thresholds and the fold chane ratio are highlighted in orange)

UNIPROT Accession	Peptides (unique)	Confidence score	Anova (p)*	Normalised abundance									Turkey's HSD test			Fold change (log2) Ratios		
				Control			Exposed_15days			Exposed_30dpi								
				R1	R2	R3	R1	R2	R3	R1	R2	R3	15days vs. C	30days vs. C	15 vs. 30days	15days/C	30days/C	15/30days
A0A1D3JPF4	12 (4)	78.8	1.90E-01	3.49E+05	4.37E+05	4.44E+05	2.84E+05	3.37E+05	3.88E+05	3.52E+05	3.76E+05	3.67E+05	1.72E-01	4.60E-01	7.06E-01	-0.29	-0.17	0.12
A0A1D3JPI1	10 (2)	50.6	NA	NA	NA	NA	NA	NA	NA	NA	NA	NA	NA	NA	NA	**NA**	**NA**	**NA**
A0A1D3JPJ0	7 (2)	51.2	9.56E-01	1.65E+05	1.42E+05	1.72E+05	1.39E+05	1.43E+05	1.11E+05	3.97E+05	0.00E+00	0.00E+00	9.62E-01	9.65E-01	1.00E+00	-0.29	-0.27	0.01
A0A1D3JPK6	3 (1)	10.8	1.31E-01	4.41E+03	4.38E+03	5.21E+03	5.18E+03	5.41E+03	6.63E+03	4.97E+03	5.25E+03	5.43E+03	1.14E-01	4.77E-01	5.07E-01	0.30	0.16	-0.14
A0A1D3JPL1	4 (1)	32.9	4.47E-03	1.65E+04	1.16E+04	1.33E+04	2.29E+04	1.98E+04	2.39E+04	2.18E+04	2.24E+04	1.99E+04	5.88E-03	9.56E-03	8.83E-01	0.69	0.63	-0.05
A0A1D3JPL2	10 (1)	52.0	7.07E-04	4.92E+03	5.04E+03	5.75E+03	4.74E+03	4.62E+03	4.06E+03	1.77E+03	2.56E+03	9.55E+02	3.00E-01	7.35E-04	2.76E-03	-0.23	**-1.57**	**-1.34**
A0A1D3JPL6	4 (1)	24.2	7.09E-01	1.82E+04	3.09E+04	2.57E+04	2.67E+04	2.29E+04	4.03E+04	1.56E+04	3.47E+04	2.41E+04	7.57E-01	1.00E+00	7.45E-01	0.27	-0.01	-0.27
A0A1D3JPN3	6 (1)	37.9	NA	NA	NA	NA	NA	NA	NA	NA	NA	NA	NA	NA	NA	**NA**	**NA**	**NA**
A0A1D3JPP4	7 (3)	44.4	9.90E-03	3.32E+04	3.60E+04	3.43E+04	8.10E+04	7.89E+04	1.15E+05	4.10E+04	4.64E+04	7.22E+04	8.86E-03	3.55E-01	4.85E-02	**1.41**	0.62	-0.79
A0A1D3JPP9	2 (0)	11.8	NA	NA	NA	NA	NA	NA	NA	NA	NA	NA	NA	NA	NA	**NA**	**NA**	**NA**
A0A1D3JPQ0	5 (1)	34.9	NA	NA	NA	NA	NA	NA	NA	NA	NA	NA	NA	NA	NA	**NA**	**NA**	**NA**
A0A1D3JPQ1	4 (1)	58.6	2.04E-03	6.63E+04	5.86E+04	8.54E+04	1.39E+05	1.33E+05	1.18E+05	9.10E+04	1.08E+05	9.34E+04	1.64E-03	5.89E-02	3.03E-02	**0.89**	0.48	-0.42
A0A1D3JPQ6|	2 (1)	65.7	NA	NA	NA	NA	NA	NA	NA	NA	NA	NA	NA	NA	NA	**NA**	**NA**	**NA**
A0A1D3JPR1	2 (1)	12.1	1.82E-02	4.37E+04	2.92E+04	3.54E+04	4.94E+04	5.32E+04	4.31E+04	5.34E+04	5.20E+04	5.24E+04	5.60E-02	1.81E-02	6.29E-01	0.43	0.54	0.11
A0A1D3JPR5	4 (0)	24.9	NA	NA	NA	NA	NA	NA	NA	NA	NA	NA	NA	NA	NA	**NA**	**NA**	**NA**
A0A1D3JPS1	3 (1)	14.4	1.01E-01	5.90E+02	3.67E+02	0.00E+00	0.00E+00	0.00E+00	0.00E+00	0.00E+00	0.00E+00	0.00E+00	1.37E-01	1.37E-01	1.00E+00	0.00	0.00	0.00
A0A1D3JPS8	3 (1)	43.7	NA	NA	NA	NA	NA	NA	NA	NA	NA	NA	NA	NA	NA	**NA**	**NA**	**NA**
A0A1D3JPT2	6 (1)	39.1	3.94E-01	2.05E+02	1.63E+03	7.43E+02	4.38E+02	6.89E+02	3.58E+03	7.25E-01	5.52E+02	1.64E+02	7.24E-01	7.77E-01	3.65E-01	**0.87**	**-1.85**	**-2.72**
A0A1D3JPT7	20 (3)	85.7	NA	NA	NA	NA	NA	NA	NA	NA	NA	NA	NA	NA	NA	**NA**	**NA**	**NA**
A0A1D3JPT9	4 (1)	21.0	3.28E-05	1.47E+02	1.67E+02	1.22E+02	2.18E+02	1.37E+02	1.78E+02	6.33E+02	6.95E+02	8.09E+02	7.85E-01	5.04E-05	7.05E-05	-0.35	**2.80**	**3.15**
A0A1D3JPU4	13 (4)	85.9	5.86E-02	4.58E+05	4.00E+05	4.20E+05	2.86E+05	3.06E+05	1.99E+05	4.04E+05	2.96E+05	2.09E+05	5.83E-02	1.43E-01	7.69E-01	-0.69	-0.49	0.20
A0A1D3JPU5	15 (3)	85.9	7.27E-01	1.93E+03	1.04E+04	4.50E+03	4.45E+03	3.28E+03	1.19E+04	0.00E+00	3.29E+03	7.82E+03	9.63E-01	8.54E-01	7.15E-01	0.22	-0.60	-0.82
A0A1D3JPU8	2 (1)	9.9	6.90E-01	9.88E+03	8.24E+03	9.22E+03	8.94E+03	8.71E+03	7.15E+03	8.41E+03	7.57E+03	1.20E+04	7.90E-01	9.83E-01	6.93E-01	-0.14	0.03	0.17
A0A1D3JPX3	31 (7)	200.5	1.86E-01	2.22E+05	1.80E+05	2.16E+05	1.73E+05	1.96E+05	1.58E+05	2.27E+05	2.01E+05	1.91E+05	2.37E-01	1.00E+00	2.35E-01	-0.23	0.00	0.23
A0A1D3JPY5	3 (2)	10.7	8.78E-01	5.99E+03	1.13E+04	8.93E+03	6.33E+03	5.08E+03	1.38E+04	4.22E+03	8.31E+03	9.46E+03	9.93E-01	8.77E-01	9.26E-01	-0.06	-0.25	-0.20
A0A1D3JPY6	6 (2)	23.9	3.59E-03	1.58E+05	1.34E+05	1.64E+05	2.50E+05	2.56E+05	2.18E+05	2.08E+05	2.28E+05	2.57E+05	4.56E-03	8.29E-03	8.22E-01	0.67	0.60	-0.06
A0A1D3JPY9	16 (7)	136.2	4.14E-01	1.33E+06	1.22E+06	1.11E+06	1.26E+06	0.00E+00	1.25E+06	8.47E+05	7.84E+05	6.16E+05	5.50E-01	4.24E-01	9.67E-01	-0.55	-0.70	-0.16
A0A1D3JPZ3	9 (3)	45.3	5.59E-02	1.82E+05	1.60E+05	1.68E+05	1.21E+05	1.30E+05	1.07E+05	1.70E+05	1.19E+05	1.13E+05	5.27E-02	1.60E-01	6.71E-01	-0.51	-0.34	0.17
A0A1D3JPZ6	7 (2)	47.0	2.36E-01	1.45E+03	7.18E+03	5.49E+03	1.23E+03	1.18E+03	4.41E+03	2.40E+03	3.77E+02	1.91E+03	3.90E-01	2.37E-01	9.10E-01	**-1.05**	**-1.59**	-0.54
A0A1D3JQ04	4 (1)	26.6	2.49E-01	9.75E+03	1.23E+04	1.37E+04	8.41E+03	8.94E+03	5.81E+03	0.00E+00	1.20E+04	7.00E+03	4.18E-01	2.46E-01	8.96E-01	-0.63	**-0.91**	-0.29
A0A1D3JQ11	6 (1)	44.1	2.29E-02	2.42E+03	3.41E+03	2.43E+03	4.14E+03	3.07E+03	4.20E+03	8.50E+03	5.81E+03	4.90E+03	5.54E-01	2.15E-02	8.04E-02	**1.93**	0.72	**-1.21**
A0A1D3JQ20	4 (1)	22.9	8.64E-01	3.02E+03	6.19E+03	5.18E+03	3.88E+03	4.08E+03	1.07E+04	1.73E+02	6.24E+03	8.51E+03	8.73E-01	9.98E-01	9.01E-01	0.37	0.05	-0.32
A0A1D3JQ23	2 (2)	10.5	2.84E-02	8.02E+04	6.43E+04	6.93E+04	4.95E+04	5.01E+04	3.62E+04	6.07E+04	3.79E+04	4.91E+04	3.22E-02	6.12E-02	8.62E-01	-0.65	-0.53	0.12
A0A1D3JQ27	17 (4)	134.6	3.83E-03	5.22E+04	4.90E+04	4.70E+04	1.21E+05	1.42E+05	1.98E+05	6.51E+04	6.63E+04	8.23E+04	4.03E-03	5.31E-01	1.26E-02	**1.64**	0.53	**-1.11**
A0A1D3JQ35	7 (1)	31.2	5.20E-01	2.02E+04	1.57E+04	2.08E+04	1.66E+04	1.75E+04	1.18E+04	2.22E+04	1.28E+04	9.86E+03	6.09E-01	5.51E-01	9.94E-01	-0.30	-0.34	-0.04
A0A1D3JQ49	2 (1)	9.1	6.57E-03	3.11E+04	2.22E+04	2.71E+04	3.30E+04	3.28E+04	2.99E+04	4.51E+04	4.48E+04	3.71E+04	3.00E-01	5.85E-03	3.51E-02	0.25	0.66	0.41
A0A1D3JQ52	41 (6)	86.5	NA	NA	NA	NA	NA	NA	NA	NA	NA	NA	NA	NA	NA	**NA**	**NA**	**NA**
A0A1D3JQ54	3 (0)	14.5	NA	NA	NA	NA	NA	NA	NA	NA	NA	NA	NA	NA	NA	**NA**	**NA**	**NA**
A0A1D3JQ60	2 (0)	9.5	NA	NA	NA	NA	NA	NA	NA	NA	NA	NA	NA	NA	NA	**NA**	**NA**	**NA**
A0A1D3JQ61	3 (2)	21.1	6.86E-01	1.43E+04	1.62E+04	1.94E+04	1.46E+04	1.85E+04	1.31E+04	2.71E+04	1.81E+04	1.17E+04	9.50E-01	8.37E-01	6.70E-01	-0.11	0.19	0.30
A0A1D3JQ62	15 (6)	111.9	1.82E-01	1.57E+06	1.21E+06	1.54E+06	1.07E+06	1.23E+06	9.09E+05	1.53E+06	1.33E+06	1.01E+06	1.64E-01	6.84E-01	4.60E-01	-0.43	-0.16	0.27
A0A1D3JQ68	27 (12)	212.4	3.10E-03	1.48E+06	1.22E+06	1.35E+06	8.97E+05	9.89E+05	8.13E+05	9.78E+05	8.21E+05	7.45E+05	6.92E-03	4.05E-03	8.48E-01	-0.59	-0.67	-0.09
A0A1D3JQ71	4 (1)	21.9	1.46E-01	2.23E+04	2.03E+04	2.53E+04	1.67E+04	1.71E+04	1.57E+04	2.30E+04	1.61E+04	1.18E+04	1.73E-01	2.10E-01	9.87E-01	-0.46	-0.42	0.04
A0A1D3JQ78	3 (1)	15.5	2.36E-03	4.14E+03	4.36E+03	4.46E+03	1.07E+04	9.47E+03	1.25E+04	8.16E+03	9.55E+03	1.18E+04	2.71E-03	6.68E-03	6.26E-01	**1.34**	**1.19**	-0.15
A0A1D3JQ85	20 (6)	170.4	3.39E-04	2.65E+05	2.12E+05	2.37E+05	3.56E+05	3.90E+05	3.51E+05	2.01E+05	1.96E+05	2.32E+05	1.13E-03	3.52E-01	3.83E-04	0.62	-0.18	-0.80

A0A1D3JQ86	2 (0)	10.2	NA	NA	NA	NA	NA	NA	NA	NA	NA	NA	NA	NA	NA	**NA**	**NA**	**NA**
A0A1D3JQ87	15 (2)	77.9	9.32E-01	7.73E+04	7.23E+04	8.24E+04	8.79E+04	9.02E+04	8.43E+04	1.37E+05	1.36E+05	0.00E+00	9.60E-01	9.30E-01	9.95E-01	0.18	0.23	0.06
A0A1D3JQA3	8 (0)	51.5	NA	NA	NA	NA	NA	NA	NA	NA	NA	NA	NA	NA	NA	**NA**	**NA**	**NA**
A0A1D3JQA5	9 (1)	76.9	NA	NA	NA	NA	NA	NA	NA	NA	NA	NA	NA	NA	NA	**NA**	**NA**	**NA**
A0A1D3JQA7	11 (1)	56.5	4.55E-01	3.89E+03	6.77E+03	3.59E+03	3.27E+03	4.54E+03	5.88E+03	0.00E+00	5.61E+03	2.53E+03	9.93E-01	4.88E-01	5.46E-01	-0.06	-0.81	-0.75
A0A1D3JQB0	21 (7)	119.1	2.85E-01	4.32E+05	4.37E+05	4.07E+05	7.07E+05	5.90E+05	3.78E+05	5.36E+05	5.75E+05	5.01E+05	3.00E-01	4.06E-01	9.64E-01	0.39	0.34	-0.06
A0A1D3JQB2	59 (25)	338.4	8.50E-04	2.31E+06	1.80E+06	2.16E+06	2.50E+06	2.69E+06	2.27E+06	4.79E+06	4.45E+06	6.07E+06	6.49E-01	1.06E-03	2.24E-03	0.25	**1.29**	**1.04**
A0A1D3JQC6	37 (7)	145.7	2.44E-04	5.56E+03	3.05E+03	3.91E+03	8.94E+03	7.77E+03	7.58E+03	1.22E+04	1.07E+04	1.18E+04	5.70E-03	1.93E-04	1.03E-02	**0.96**	**0.94**	-0.02
A0A1D3JQD1	14 (7)	76.7	4.48E-01	3.18E+05	2.74E+05	2.96E+05	3.51E+05	3.64E+05	3.09E+05	3.76E+05	3.36E+05	2.55E+05	4.22E-01	7.24E-01	8.44E-01	0.21	0.12	-0.08
A0A1D3JQD4	2 (1)	19.5	2.22E-01	1.35E+03	1.70E+03	1.23E+03	2.04E+03	1.83E+03	4.69E+03	1.39E+03	1.93E+03	1.68E+03	2.34E-01	9.50E-01	3.40E-01	**1.00**	0.22	-0.78
A0A1D3JQE3	14 (3)	72.8	9.73E-01	4.40E+04	5.06E+04	5.19E+04	5.43E+04	5.34E+04	6.52E+04	0.00E+00	0.00E+00	1.50E+05	9.75E-01	1.00E+00	9.81E-01	0.24	0.03	-0.21
A0A1D3JQE4	17 (3)	100.2	1.83E-01	5.11E+05	3.75E+05	4.94E+05	3.66E+05	4.07E+05	2.65E+05	4.34E+05	3.64E+05	3.26E+05	1.81E-01	3.39E-01	8.69E-01	-0.41	-0.30	0.11
A0A1D3JQE6	12 (3)	74.5	6.93E-01	2.56E+05	2.04E+05	2.27E+05	2.58E+05	2.77E+05	1.94E+05	2.85E+05	2.31E+05	2.44E+05	8.67E-01	6.72E-01	9.31E-01	0.59	0.50	-0.09
A0A1D3JQE9	9 (1)	53.6	6.82E-01	1.15E+04	8.76E+03	1.39E+04	7.49E+03	6.94E+03	3.38E+03	0.00E+00	2.33E+04	4.04E+03	6.60E-01	9.25E-01	8.64E-01	**-0.94**	-0.32	0.62
A0A1D3JQG4	5 (2)	28.6	9.84E-01	8.50E+04	7.63E+04	8.95E+04	7.95E+04	8.82E+04	4.90E+04	0.00E+00	2.46E+05	0.00E+00	9.85E-01	1.00E+00	9.88E-01	-0.21	-0.03	0.18
A0A1D3JQH2	3 (1)	20.5	6.89E-01	3.58E+03	3.85E+03	4.06E+03	2.71E+03	2.44E+03	3.19E+03	6.30E+02	5.38E+03	4.69E+03	6.85E-01	9.75E-01	8.04E-01	-0.46	-0.10	0.36
A0A1D3JQH4	14 (2)	84.9	1.73E-03	4.94E+03	5.66E+03	7.99E+03	5.29E+03	4.93E+03	4.34E+03	1.15E+03	1.10E+03	1.24E+03	2.75E-01	1.66E-03	8.01E-03	-0.35	**-2.41**	**-2.06**
A0A1D3JQJ5	4 (0)	21.0	NA	NA	NA	NA	NA	NA	NA	NA	NA	NA	NA	NA	NA	**NA**	**NA**	**NA**
A0A1D3JQK4	15 (4)	85.6	7.28E-01	1.16E+05	9.21E+04	1.19E+05	1.13E+05	1.20E+05	9.23E+04	1.39E+05	1.12E+05	1.03E+05	9.99E-01	7.77E-01	7.59E-01	-0.01	0.12	0.12
A0A1D3JQK6	4 (0)	18.7	NA	NA	NA	NA	NA	NA	NA	NA	NA	NA	NA	NA	NA	**NA**	**NA**	**NA**
A0A1D3JQL2	15 (8)	101.3	3.85E-01	1.24E+05	1.65E+05	1.47E+05	1.38E+05	1.31E+05	2.09E+05	1.07E+05	1.49E+05	1.09E+05	8.43E-01	6.49E-01	3.62E-01	0.13	-0.25	-0.38
A0A1D3JQL3	6 (2)	46.8	4.06E-01	9.06E+02	1.93E+03	1.12E+03	2.18E+03	9.71E+02	4.65E+03	5.25E+02	2.25E+03	1.30E+03	4.57E-01	9.99E-01	4.77E-01	**0.98**	0.04	**-0.94**
A0A1D3JQM4	9 (1)	47.6	1.15E-05	3.26E+03	2.82E+03	3.79E+03	0.00E+00	8.91E+01	0.00E+00	1.09E+02	9.72E+01	0.00E+00	1.99E-05	2.14E-05	9.85E-01	**-6.79**	**-5.58**	**1.21**
A0A1D3JQM5	21 (2)	115.1	7.97E-01	5.19E+04	4.88E+04	4.86E+04	3.83E+04	4.18E+04	3.38E+04	7.37E+04	4.97E+04	0.00E+00	7.92E-01	8.81E-01	9.83E-01	-0.39	-0.28	0.12
A0A1D3JQN4	7 (3)	45.3	1.48E-02	4.59E+04	3.37E+04	3.82E+04	2.47E+04	2.60E+04	1.89E+04	2.81E+04	2.17E+04	1.54E+04	2.81E-02	1.93E-02	9.44E-01	-0.76	**-0.85**	-0.09
A0A1D3JQP1	6 (2)	55.9	5.06E-01	3.44E+04	2.90E+04	3.41E+04	5.85E+04	5.77E+04	5.57E+04	9.97E+04	0.00E+00	8.56E+04	6.18E-01	5.23E-01	9.84E-01	0.82	**0.93**	0.11
A0A1D3JQP4	51 (11)	311.9	3.39E-01	2.84E+05	2.37E+05	2.67E+05	3.38E+05	3.53E+05	2.85E+05	3.62E+05	2.54E+05	2.19E+05	3.36E-01	9.23E-01	5.13E-01	0.31	0.08	-0.23
A0A1D3JQP5	28 (8)	218.3	3.38E-02	4.07E+05	4.09E+05	4.56E+05	4.70E+05	4.67E+05	4.83E+05	3.65E+05	4.37E+05	3.74E+05	1.65E-01	4.02E-01	2.91E-02	0.16	-0.11	-0.27
A0A1D3JQQ0	22 (3)	114.8	6.87E-01	1.94E+04	1.58E+04	1.58E+04	1.40E+04	1.47E+04	1.05E+04	2.07E+04	1.70E+04	0.00E+00	7.60E-01	7.07E-01	9.95E-01	-0.38	-0.43	-0.06
A0A1D3JQR5	8 (1)	40.7	6.47E-01	6.56E+01	1.79E+03	1.10E+02	2.08E+02	3.26E+01	2.41E+03	0.00E+00	0.00E+00	4.21E+02	9.55E-01	7.96E-01	6.34E-01	0.43	**-2.23**	**-2.66**
A0A1D3JQT2	18 (3)	104.9	4.98E-02	2.49E+04	4.04E+04	2.93E+04	1.22E+04	1.15E+04	2.76E+04	1.24E+04	1.65E+04	1.33E+04	1.04E-01	5.41E-02	8.64E-01	**-0.88**	**-1.16**	-0.28
A0A1D3JQT8	7 (3)	43.5	1.91E-01	1.72E+05	1.29E+05	1.55E+05	1.16E+05	1.36E+05	7.67E+04	2.25E+05	1.64E+05	1.22E+05	3.85E-01	8.16E-01	1.82E-01	-0.47	0.16	0.64
A0A1D3JQU0	2 (0)	11.8	NA	NA	NA	NA	NA	NA	NA	NA	NA	NA	NA	NA	NA	**NA**	**NA**	**NA**
A0A1D3JQU3	8 (1)	12.5	5.39E-01	1.09E+04	6.45E+03	6.95E+03	7.08E+03	5.70E+03	6.65E+03	5.24E+03	7.64E+03	8.21E+03	5.20E-01	7.39E-01	9.20E-01	-0.32	-0.20	0.12
A0A1D3JQU4	16 (6)	98.3	3.00E-02	3.19E+05	2.69E+05	3.09E+05	3.41E+05	3.47E+05	3.26E+05	4.30E+05	3.48E+05	3.77E+05	2.98E-01	2.51E-02	1.94E-01	0.18	0.37	0.19
A0A1D3JQU6	6 (2)	19.2	1.75E-01	5.88E+04	4.86E+04	6.63E+04	4.56E+04	5.12E+04	3.27E+04	5.49E+04	4.70E+04	4.10E+04	1.66E-01	3.65E-01	8.03E-01	-0.42	-0.28	0.14
A0A1D3JQU8	5 (1)	36.0	3.44E-01	3.01E+02	9.19E+02	6.34E+02	4.76E+02	3.24E+02	1.00E+03	0.00E+00	4.53E+02	3.42E+02	9.97E-01	3.90E-01	4.21E-01	-0.04	**-1.22**	**-1.18**
A0A1D3JQV8	18 (3)	67.9	NA	NA	NA	NA	NA	NA	NA	NA	NA	NA	NA	NA	NA	**NA**	**NA**	**NA**
A0A1D3JQW0	2 (0)	9.5	NA	NA	NA	NA	NA	NA	NA	NA	NA	NA	NA	NA	NA	**NA**	**NA**	**NA**
A0A1D3JQW6	2 (0)	14.3	NA	NA	NA	NA	NA	NA	NA	NA	NA	NA	NA	NA	NA	**NA**	**NA**	**NA**
A0A1D3JQW8	2 (0)	11.3	NA	NA	NA	NA	NA	NA	NA	NA	NA	NA	NA	NA	NA	**NA**	**NA**	**NA**
A0A1D3JQX6	10 (6)	62.6	8.18E-01	6.81E+05	5.12E+05	6.40E+05	5.80E+05	4.71E+05	5.24E+05	3.56E+05	5.21E+05	9.56E+05	8.46E-01	1.00E+00	8.46E-01	-0.22	0.00	0.22
A0A1D3JQX8	18 (5)	124.2	5.52E-02	3.55E+04	3.58E+04	3.92E+04	4.42E+04	4.53E+04	5.82E+04	4.38E+04	5.15E+04	5.69E+04	9.70E-02	6.60E-02	9.50E-01	0.42	0.46	0.04
A0A1D3JQZ1	5 (2)	30.7	8.26E-01	2.96E+03	3.35E+03	3.57E+03	3.24E+03	2.83E+03	5.60E+03	0.00E+00	5.15E+03	3.83E+03	9.12E-01	9.77E-01	8.16E-01	0.24	-0.14	-0.38
A0A1D3JQZ2	13 (3)	70.5	1.51E-01	1.10E+05	9.41E+04	9.88E+04	7.54E+04	8.31E+04	6.83E+04	9.84E+04	5.95E+04	9.36E+04	1.41E-01	3.47E-01	7.57E-01	-0.42	-0.27	0.15
A0A1D3JQZ5	28 (9)	262.9	1.31E-03	8.15E+05	6.45E+05	7.14E+05	1.25E+06	1.37E+06	1.09E+06	1.12E+06	1.19E+06	1.14E+06	1.51E-03	3.87E-03	5.58E-01	0.77	0.67	-0.10
A0A1D3JR00	29 (8)	167.8	7.59E-01	2.30E+05	1.83E+05	2.05E+05	2.19E+05	2.19E+05	2.08E+05	2.42E+05	1.85E+05	1.71E+05	9.00E-01	9.47E-01	7.41E-01	0.06	-0.05	-0.11
A0A1D3JR12	17 (7)	106.5	5.21E-01	2.44E+05	1.77E+05	2.42E+05	1.99E+05	2.24E+05	2.13E+05	2.40E+05	2.21E+05	2.47E+05	8.97E-01	7.47E-01	4.98E-01	-0.06	0.09	0.15
A0A1D3JR13	4 (0)	29.4	NA	NA	NA	NA	NA	NA	NA	NA	NA	NA	NA	NA	NA	**NA**	**NA**	**NA**
A0A1D3JR14	17 (6)	130.9	1.01E-01	3.00E+05	3.00E+05	3.06E+05	3.45E+05	3.32E+05	4.13E+05	3.26E+05	4.18E+05	3.83E+05	1.84E-01	1.08E-01	9.10E-01	0.27	0.32	0.05
A0A1D3JR17	4 (2)	18.2	6.88E-01	1.49E+05	1.56E+05	1.66E+05	1.39E+05	1.36E+05	1.85E+05	1.16E+05	1.65E+05	1.44E+05	9.77E-01	6.85E-01	8.00E-01	-0.03	-0.15	-0.11
A0A1D3JR22	5 (2)	43.1	3.03E-01	2.05E+04	3.24E+04	1.81E+04	1.41E+04	2.51E+04	2.96E+04	1.75E+04	0.00E+00	2.00E+04	9.94E-01	3.42E-01	3.85E-01	-0.05	**-0.92**	**-0.88**

A0A1D3JR26	24 (9)	123.1	5.60E-02	2.92E+05	2.79E+05	3.06E+05	2.85E+05	2.95E+05	3.30E+05	2.80E+05	2.40E+05	2.24E+05	8.21E-01	1.25E-01	5.80E-02	0.06	-0.23	-0.29
A0A1D3JR27	10 (1)	59.7	4.49E-01	9.59E+01	5.17E+01	3.90E+03	2.46E+01	1.32E+01	2.47E+01	4.38E+02	1.51E+01	3.16E+02	4.60E-01	5.79E-01	9.73E-01	**-6.02**	**-2.40**	**3.62**
A0A1D3JR37	5 (2)	25.8	5.67E-03	2.83E+05	2.30E+05	2.64E+05	1.35E+05	1.52E+05	9.06E+04	2.33E+05	1.80E+05	1.69E+05	4.58E-03	9.51E-02	7.90E-02	**-1.04**	-0.41	0.63
A0A1D3JR39	12 (3)	64.0	3.61E-01	1.68E+05	1.13E+05	1.30E+05	1.44E+05	1.65E+05	1.09E+05	2.04E+05	1.41E+05	1.66E+05	9.95E-01	4.02E-01	4.45E-01	0.02	0.31	0.29
A0A1D3JR45	3 (2)	16.1	5.40E-01	4.42E+04	3.41E+04	4.69E+04	3.47E+04	3.95E+04	2.72E+04	4.95E+04	3.30E+04	2.81E+04	5.16E-01	7.68E-01	8.95E-01	-0.30	-0.18	0.13
A0A1D3JR46	5 (0)	31.7	NA	NA	NA	NA	NA	NA	NA	NA	NA	NA	NA	NA	NA	**NA**	**NA**	**NA**
A0A1D3JR49	10 (2)	62.6	4.76E-02	1.66E+05	1.37E+05	1.43E+05	1.63E+05	1.64E+05	1.35E+05	2.33E+05	1.72E+05	1.98E+05	9.53E-01	5.78E-02	8.39E-02	0.05	0.43	0.38
A0A1D3JR62	4 (1)	22.6	5.06E-01	3.05E+03	2.30E+04	3.17E+03	7.48E+03	7.66E+03	3.70E+04	7.94E+01	6.44E+03	9.20E+03	7.34E-01	8.94E-01	4.83E-01	0.83	**-0.90**	**-1.73**
A0A1D3JR63	14 (2)	115.9	7.64E-01	2.18E+03	7.15E+03	3.66E+03	4.77E+03	3.00E+03	1.19E+04	5.23E+02	8.49E+03	5.27E+03	7.68E-01	9.90E-01	8.40E-01	0.60	0.14	-0.46
A0A1D3JR74	5 (1)	56.9	NA	NA	NA	NA	NA	NA	NA	NA	NA	NA	NA	NA	NA	**NA**	**NA**	**NA**
A0A1D3JR75	2 (0)	13.4	NA	NA	NA	NA	NA	NA	NA	NA	NA	NA	NA	NA	NA	**NA**	**NA**	**NA**
A0A1D3JR76	3 (2)	21.2	8.12E-03	3.90E+05	3.31E+05	4.01E+05	3.05E+05	3.09E+05	2.57E+05	2.76E+05	2.21E+05	2.53E+05	4.16E-02	7.25E-03	3.28E-01	-0.36	-0.58	-0.22
A0A1D3JR85	6 (1)	63.9	NA	NA	NA	NA	NA	NA	NA	NA	NA	NA	NA	NA	NA	**NA**	**NA**	**NA**
A0A1D3JR88	45 (13)	291.1	2.05E-05	6.16E+05	5.57E+05	6.43E+05	6.98E+05	7.03E+05	7.15E+05	9.09E+05	9.43E+05	9.23E+05	1.01E-02	1.80E-05	1.60E-04	0.22	0.61	0.39
A0A1D3JR94	4 (1)	24.0	1.73E-05	1.59E+05	1.53E+05	1.87E+05	2.90E+05	3.12E+05	2.88E+05	1.10E+05	1.30E+05	1.02E+05	1.12E-04	1.41E-02	1.58E-05	0.84	-0.54	**-1.38**
A0A1D3JRA5	3 (0)	9.9	NA	NA	NA	NA	NA	NA	NA	NA	NA	NA	NA	NA	NA	**NA**	**NA**	**NA**
A0A1D3JRA8	12 (1)	34.8	NA	NA	NA	NA	NA	NA	NA	NA	NA	NA	NA	NA	NA	**NA**	**NA**	**NA**
A0A1D3JRA9	9 (0)	49.6	NA	NA	NA	NA	NA	NA	NA	NA	NA	NA	NA	NA	NA	**NA**	**NA**	**NA**
A0A1D3JRB0	2 (0)	8.5	NA	NA	NA	NA	NA	NA	NA	NA	NA	NA	NA	NA	NA	**NA**	**NA**	**NA**
A0A1D3JRB1	16 (8)	125.7	1.40E-01	2.55E+05	1.92E+05	1.85E+05	2.92E+05	3.06E+05	2.80E+05	2.61E+05	2.75E+05	4.36E+05	3.00E-01	1.34E-01	8.04E-01	0.47	0.62	0.15
A0A1D3JRB2	8 (2)	44.7	6.82E-01	2.65E+02	6.47E+03	2.93E+02	3.03E+02	1.72E+02	5.72E+03	1.01E+02	9.59E+02	2.94E+02	9.92E-01	6.96E-01	7.65E-01	-0.18	**-2.38**	**-2.19**
A0A1D3JRB4	2 (1)	20.0	3.12E-04	1.67E+02	1.40E+02	1.51E+02	4.48E+02	4.80E+02	5.61E+02	3.07E+02	4.02E+02	3.10E+02	2.48E-04	6.21E-03	1.46E-02	**1.70**	**1.16**	-0.55
A0A1D3JRB5	10 (4)	50.9	7.35E-01	6.76E+04	6.04E+04	6.64E+04	7.34E+04	7.60E+04	7.90E+04	0.00E+00	1.02E+05	6.66E+04	8.91E-01	9.38E-01	7.15E-01	0.23	-0.20	-0.43
A0A1D3JRB8	13 (5)	91.4	4.08E-01	2.49E+05	2.76E+05	2.89E+05	2.37E+05	2.22E+05	3.08E+05	2.18E+05	3.71E+05	3.81E+05	9.48E-01	5.68E-01	4.08E-01	-0.08	0.25	0.34
A0A1D3JRC4	11 (1)	45.8	NA	NA	NA	NA	NA	NA	NA	NA	NA	NA	NA	NA	NA	**NA**	**NA**	**NA**
A0A1D3JRC8	5 (2)	27.0	1.39E-01	6.05E+04	6.07E+04	6.15E+04	7.46E+04	6.88E+04	1.07E+05	6.78E+04	1.07E+05	1.15E+05	3.68E-01	1.25E-01	6.77E-01	0.45	0.67	0.21
A0A1D3JRD8	15 (1)	65.9	8.07E-03	1.47E+04	1.71E+04	1.14E+04	2.08E+04	2.39E+04	2.31E+04	2.05E+04	2.61E+04	2.39E+04	1.68E-02	1.04E-02	9.01E-01	0.65	0.71	0.06
A0A1D3JRE5	3 (2)	9.6	1.33E-02	1.09E+05	7.59E+04	9.13E+04	2.31E+05	2.63E+05	1.62E+05	2.00E+05	1.65E+05	1.43E+05	1.13E-02	8.37E-02	2.80E-01	**1.24**	**0.88**	-0.37
A0A1D3JRF7	4 (1)	24.7	2.22E-01	1.21E+04	1.61E+04	1.57E+04	1.88E+04	1.83E+04	2.07E+04	1.19E+04	2.07E+04	1.32E+04	2.39E-01	9.61E-01	3.31E-01	0.40	0.07	-0.33
A0A1D3JRI0	11 (3)	77.3	4.08E-03	1.02E+05	1.20E+05	1.21E+05	9.48E+04	9.50E+04	1.09E+05	8.06E+04	7.49E+04	6.60E+04	1.93E-01	3.50E-03	2.82E-02	-0.20	-0.63	-0.43
A0A1D3JRI3	2 (0)	9.4	NA	NA	NA	NA	NA	NA	NA	NA	NA	NA	NA	NA	NA	**NA**	**NA**	**NA**
A0A1D3JRJ4	2 (0)	9.4	NA	NA	NA	NA	NA	NA	NA	NA	NA	NA	NA	NA	NA	**NA**	**NA**	**NA**
A0A1D3JRL0	3 (0)	23.0	NA	NA	NA	NA	NA	NA	NA	NA	NA	NA	NA	NA	NA	**NA**	**NA**	**NA**
A0A1D3JRL1	6 (5)	23.3	1.24E-01	3.33E+05	2.32E+05	7.40E+04	7.28E+04	1.01E+05	5.27E+04	1.05E+05	8.06E+04	4.69E+04	1.61E-01	1.67E-01	9.99E-01	**-1.50**	**-1.46**	0.04
A0A1D3JRL2	4 (1)	45.9	NA	NA	NA	NA	NA	NA	NA	NA	NA	NA	NA	NA	NA	**NA**	**NA**	**NA**
A0A1D3JRL7	15 (5)	110.8	5.84E-01	2.73E+05	2.14E+05	2.38E+05	2.20E+05	2.55E+05	2.00E+05	2.77E+05	1.37E+05	1.87E+05	9.01E-01	5.59E-01	8.03E-01	-0.10	-0.27	-0.17
A0A1D3JRM3	11 (5)	61.0	1.40E-01	8.45E+04	1.09E+05	1.02E+05	1.34E+05	1.26E+05	1.82E+05	9.88E+04	1.58E+05	1.32E+05	1.28E-01	3.56E-01	7.04E-01	0.58	0.40	-0.18
A0A1D3JRM8	6 (1)	38.3	6.74E-02	6.57E+03	7.40E+03	7.01E+03	2.04E+04	1.58E+04	3.10E+04	9.99E+03	2.63E+04	2.56E+04	7.93E-02	1.17E-01	9.51E-01	**1.68**	**1.56**	-0.12
A0A1D3JRN4	54 (16)	438.4	5.26E-05	1.96E+06	1.72E+06	1.77E+06	1.60E+06	1.64E+06	1.65E+06	1.08E+06	1.02E+06	9.58E+05	6.98E-02	5.33E-05	2.47E-04	-0.16	-0.83	-0.68
A0A1D3JRP9	2 (1)	5.2	7.49E-03	1.47E+03	1.49E+03	2.35E+03	8.09E+02	5.78E+02	1.62E+02	6.41E+02	5.48E+01	0.00E+00	2.09E-02	8.20E-03	6.80E-01	**-1.78**	**-2.93**	**-1.16**
A0A1D3JRQ8	4 (2)	19.7	2.11E-03	7.20E+04	6.74E+04	6.70E+04	9.63E+04	9.68E+04	1.03E+05	8.78E+04	1.08E+05	1.04E+05	3.97E-03	3.20E-03	9.70E-01	0.52	0.54	0.02
A0A1D3JRR2	6 (0)	41.3	NA	NA	NA	NA	NA	NA	NA	NA	NA	NA	NA	NA	NA	**NA**	**NA**	**NA**
A0A1D3JRS0	11 (3)	79.6	2.60E-01	2.48E+04	2.00E+04	2.20E+04	3.05E+04	3.18E+04	1.91E+04	2.67E+04	2.92E+04	2.98E+04	4.20E-01	2.60E-01	9.15E-01	0.28	0.36	0.08
A0A1D3JRS4	16 (6)	97.2	1.60E-02	6.92E+04	7.50E+04	7.28E+04	1.37E+05	1.29E+05	1.60E+05	1.53E+05	2.21E+05	3.05E+05	2.18E-01	1.33E-02	1.31E-01	**0.97**	**1.65**	0.67
A0A1D3JRS8	19 (8)	149.7	2.13E-03	6.50E+05	5.95E+05	6.63E+05	8.45E+05	8.86E+05	8.57E+05	6.63E+05	7.65E+05	7.79E+05	1.72E-03	7.06E-02	2.74E-02	0.44	0.21	-0.23
A0A1D3JRT8	11 (1)	70.2	9.80E-02	6.26E+03	9.55E+03	8.45E+03	7.09E+03	6.29E+03	8.11E+03	9.02E+03	9.47E+03	1.12E+04	6.74E-01	2.71E-01	8.96E-02	-0.17	0.29	0.47
A0A1D3JRT9	4 (2)	21.7	3.33E-03	3.83E+04	4.41E+04	4.39E+04	2.29E+04	2.20E+04	2.84E+04	2.51E+04	2.77E+04	1.60E+04	6.88E-03	4.58E-03	9.10E-01	-0.78	**-0.88**	-0.09
A0A1D3JRU4	5 (1)	29.3	5.55E-02	1.48E+05	1.14E+05	1.36E+05	1.27E+05	1.40E+05	1.13E+05	1.80E+05	1.44E+05	1.77E+05	8.95E-01	1.09E-01	6.17E-02	-0.07	0.33	0.40
A0A1D3JRU5	8 (2)	55.6	1.16E-02	1.37E+05	1.39E+05	1.50E+05	1.96E+05	2.11E+05	1.75E+05	1.32E+05	1.38E+05	8.13E+04	5.44E-02	3.82E-01	1.04E-02	0.45	-0.28	-0.73
A0A1D3JRW1	13 (7)	116.7	5.36E-03	1.02E+05	6.23E+04	6.43E+04	1.06E+05	1.18E+05	1.07E+05	1.92E+05	1.63E+05	2.56E+05	4.08E-01	5.14E-03	2.20E-02	0.54	**1.42**	**0.88**
A0A1D3JRW6	15 (4)	116.6	3.58E-01	2.34E+05	1.96E+05	2.39E+05	1.88E+05	2.12E+05	1.57E+05	2.38E+05	1.70E+05	1.65E+05	3.77E-01	4.76E-01	9.76E-01	-0.26	-0.22	0.04
A0A1D3JRW8	3 (2)	15.1	2.74E-02	5.02E+04	4.25E+04	5.07E+04	5.76E+04	5.87E+04	5.41E+04	5.43E+04	6.27E+04	6.29E+04	8.32E-02	2.66E-02	6.42E-01	0.25	0.33	0.08

A0A1D3JRX0	4 (2)	22.4	6.12E-02	5.72E+04	5.51E+04	5.87E+04	7.13E+04	7.49E+04	6.60E+04	7.96E+04	7.31E+04	1.14E+05	4.41E-01	5.25E-02	2.74E-01	0.31	0.64	0.33
A0A1D3JRX2	11 (2)	60.7	5.12E-02	8.11E+04	6.08E+04	6.88E+04	9.91E+04	1.03E+05	9.03E+04	9.29E+04	7.75E+04	6.48E+04	4.78E-02	6.42E-01	1.55E-01	0.47	0.16	-0.31
A0A1D3JRX5	5 (1)	89.7	NA	NA	NA	NA	NA	NA	NA	NA	NA	NA	NA	NA	NA	NA	NA	NA
A0A1D3JRX9	12 (3)	79.0	NA	NA	NA	NA	NA	NA	NA	NA	NA	NA	NA	NA	NA	NA	NA	NA
A0A1D3JRZ9	11 (1)	75.5	3.70E-04	3.70E+04	2.73E+04	3.26E+04	5.26E+04	5.09E+04	5.01E+04	5.79E+04	5.53E+04	5.19E+04	1.17E-03	4.28E-04	4.07E-01	0.67	0.77	0.10
A0A1D3JS01	12 (3)	63.1	3.68E-03	2.97E+04	3.12E+04	3.30E+04	4.01E+04	3.84E+04	3.89E+04	4.79E+04	4.12E+04	5.22E+04	6.69E-02	2.96E-03	6.17E-02	0.32	0.59	0.27
A0A1D3JS08	4 (0)	20.9	NA	NA	NA	NA	NA	NA	NA	NA	NA	NA	NA	NA	NA	NA	NA	NA
A0A1D3JS14	10 (3)	62.6	2.20E-03	2.21E+05	1.69E+05	2.16E+05	3.36E+05	3.71E+05	3.09E+05	2.51E+05	2.04E+05	2.11E+05	2.63E-03	6.81E-01	5.87E-03	0.75	0.14	-0.61
A0A1D3JS15	23 (8)	173.3	8.78E-04	4.87E+05	3.99E+05	4.73E+05	5.83E+05	6.23E+05	5.54E+05	8.32E+05	8.04E+05	1.00E+06	1.31E-01	7.89E-04	5.57E-03	0.37	**0.96**	0.59
A0A1D3JS16	3 (0)	15.9	NA	NA	NA	NA	NA	NA	NA	NA	NA	NA	NA	NA	NA	NA	NA	NA
A0A1D3JS21	3 (0)	15.3	NA	NA	NA	NA	NA	NA	NA	NA	NA	NA	NA	NA	NA	NA	NA	NA
A0A1D3JS27	2 (1)	10.4	5.49E-03	2.77E+03	3.37E+03	2.99E+03	2.41E+03	3.18E+03	2.29E+03	1.11E+03	1.75E+03	6.78E+02	5.32E-01	5.64E-03	1.85E-02	-0.21	**-1.37**	**-1.16**
A0A1D3JS30	26 (10)	174.6	5.96E-02	1.13E+05	1.25E+05	1.16E+05	2.22E+05	2.22E+05	3.76E+05	1.38E+05	2.50E+05	2.38E+05	5.15E-02	2.55E-01	4.60E-01	**1.21**	0.82	-0.39
A0A1D3JS31	9 (0)	59.8	NA	NA	NA	NA	NA	NA	NA	NA	NA	NA	NA	NA	NA	NA	NA	NA
A0A1D3JS34	5 (0)	41.7	NA	NA	NA	NA	NA	NA	NA	NA	NA	NA	NA	NA	NA	NA	NA	NA
A0A1D3JS35	2 (1)	11.5	7.42E-01	2.41E+04	2.20E+04	2.60E+04	2.14E+04	2.17E+04	2.06E+04	3.04E+04	2.15E+04	1.63E+04	7.21E-01	9.32E-01	9.04E-01	-0.18	-0.08	0.10
A0A1D3JS37	5 (2)	49.5	2.05E-01	6.06E+05	4.53E+05	5.34E+05	4.26E+05	4.63E+05	3.44E+05	6.14E+05	4.59E+05	4.59E+05	2.16E-01	9.45E-01	3.21E-01	-0.37	-0.06	0.31
A0A1D3JS38	4 (0)	22.9	NA	NA	NA	NA	NA	NA	NA	NA	NA	NA	NA	NA	NA	NA	NA	NA
A0A1D3JS41	16 (4)	97.2	5.19E-02	8.37E+04	8.72E+04	8.51E+04	8.25E+04	9.07E+04	9.33E+04	7.67E+04	8.19E+04	6.77E+04	7.13E-01	1.39E-01	5.03E-02	0.06	-0.18	-0.24
A0A1D3JS51	3 (1)	15.4	4.76E-03	6.40E+03	4.76E+03	7.42E+03	2.13E+03	1.61E+03	1.60E+03	3.13E+03	3.77E+02	4.02E+02	1.02E-02	6.24E-03	8.83E-01	**-1.80**	**-2.25**	-0.45
A0A1D3JS52	12 (3)	83.9	3.90E-04	5.66E+04	5.58E+04	6.46E+04	7.89E+04	8.09E+04	7.75E+04	4.28E+04	5.34E+04	5.01E+04	3.08E-03	6.27E-02	3.40E-04	0.42	-0.27	-0.70
A0A1D3JS54	7 (2)	49.8	1.28E-03	1.39E+04	1.69E+04	1.27E+04	4.95E+03	4.45E+03	5.21E+03	5.36E+03	9.30E+03	5.74E+03	1.38E-03	4.39E-03	4.31E-01	**-1.57**	**-1.09**	0.48
A0A1D3JS77	2 (1)	11.1	7.42E-02	1.43E+03	1.37E+03	1.35E+03	2.74E+03	2.83E+03	1.49E+03	2.71E+03	4.05E+03	2.24E+03	2.75E-01	6.49E-02	5.26E-01	0.77	**1.12**	0.35
A0A1D3JS79	5 (1)	56.8	NA	NA	NA	NA	NA	NA	NA	NA	NA	NA	NA	NA	NA	NA	NA	NA
A0A1D3JSA4	2 (0)	10.0	NA	NA	NA	NA	NA	NA	NA	NA	NA	NA	NA	NA	NA	NA	NA	NA
A0A1D3JSA5	20 (10)	171.4	2.70E-02	3.89E+05	3.22E+05	3.84E+05	5.65E+05	5.59E+05	5.29E+05	5.87E+05	5.12E+05	3.89E+05	2.51E-02	9.47E-02	5.57E-01	0.59	0.44	-0.15
A0A1D3JSB3	3 (0)	14.4	NA	NA	NA	NA	NA	NA	NA	NA	NA	NA	NA	NA	NA	NA	NA	NA
A0A1D3JSC3	12 (7)	109.9	1.11E-03	1.44E+05	1.53E+05	1.41E+05	2.21E+05	2.36E+05	2.66E+05	3.64E+05	3.57E+05	4.86E+05	8.50E-02	9.34E-04	9.89E-03	0.72	**1.46**	0.74
A0A1D3JSC7	4 (1)	45.8	NA	NA	NA	NA	NA	NA	NA	NA	NA	NA	NA	NA	NA	NA	NA	NA
A0A1D3JSD1	10 (2)	68.9	4.35E-01	6.27E+04	5.35E+04	5.72E+04	6.59E+04	6.38E+04	6.07E+04	5.36E+04	6.72E+04	6.44E+04	4.22E-01	6.34E-01	9.16E-01	0.14	0.10	-0.04
A0A1D3JSD2	16 (7)	155.8	4.88E-02	3.39E+05	3.37E+05	3.74E+05	5.23E+05	4.93E+05	5.27E+05	3.85E+05	5.53E+05	6.29E+05	7.65E-02	6.49E-02	9.91E-01	0.55	0.58	0.02
A0A1D3JSD5	7 (3)	44.1	5.07E-01	3.21E+05	2.38E+05	2.56E+05	2.29E+05	2.53E+05	2.09E+05	3.47E+05	2.40E+05	2.28E+05	5.63E-01	1.00E+00	5.68E-01	-0.24	0.00	0.24
A0A1D3JSE9	4 (0)	21.6	NA	NA	NA	NA	NA	NA	NA	NA	NA	NA	NA	NA	NA	NA	NA	NA
A0A1D3JSF1	23 (10)	175.2	3.61E-01	3.68E+05	4.32E+05	3.10E+05	4.39E+05	4.15E+05	4.51E+05	5.08E+05	4.38E+05	3.53E+05	4.12E-01	4.32E-01	9.99E-01	0.23	0.23	-0.01
A0A1D3JSF6	2 (0)	10.8	NA	NA	NA	NA	NA	NA	NA	NA	NA	NA	NA	NA	NA	NA	NA	NA
A0A1D3JSF8	123 (11)	543.7	NA	NA	NA	NA	NA	NA	NA	NA	NA	NA	NA	NA	NA	NA	NA	NA
A0A1D3JSG1	4 (1)	22.4	1.43E-02	8.78E+03	1.18E+04	7.50E+03	3.56E+03	2.73E+03	5.90E+03	4.48E+03	4.98E+03	2.67E+03	2.24E-02	2.20E-02	1.00E+00	**-1.20**	**-1.21**	-0.01
A0A1D3JSG8	3 (0)	18.6	NA	NA	NA	NA	NA	NA	NA	NA	NA	NA	NA	NA	NA	NA	NA	NA
A0A1D3JSG9	5 (1)	25.6	5.06E-02	4.92E+04	4.05E+04	5.07E+04	7.30E+04	7.95E+04	5.98E+04	7.08E+04	4.87E+04	5.85E+04	4.28E-02	2.90E-01	3.46E-01	0.60	0.34	-0.25
A0A1D3JSH2	7 (2)	30.8	1.20E-02	7.62E+04	7.58E+04	8.47E+04	5.09E+04	4.85E+04	5.66E+04	5.86E+04	6.59E+04	4.22E+04	1.44E-02	2.66E-02	8.55E-01	-0.60	-0.51	0.09
A0A1D3JSJ1	31 (4)	123.7	NA	NA	NA	NA	NA	NA	NA	NA	NA	NA	NA	NA	NA	NA	NA	NA
A0A1D3JSJ6	8 (1)	56.8	NA	NA	NA	NA	NA	NA	NA	NA	NA	NA	NA	NA	NA	NA	NA	NA
A0A1D3JSK7	9 (4)	59.4	2.29E-01	3.31E+04	4.90E+04	3.70E+04	3.27E+04	3.54E+04	5.47E+04	2.61E+04	3.31E+04	2.57E+04	9.84E-01	3.17E-01	2.57E-01	0.04	-0.49	-0.53
A0A1D3JSM2	3 (1)	15.5	1.03E-01	8.51E+03	5.57E+03	7.87E+03	6.42E+03	5.53E+03	5.48E+03	5.66E+03	5.35E+03	5.14E+03	2.09E-01	1.04E-01	8.52E-01	-0.33	-0.44	-0.11
A0A1D3JSM7	16 (4)	96.8	3.33E-01	1.86E+05	1.85E+05	1.99E+05	1.74E+05	1.79E+05	1.65E+05	2.08E+05	1.85E+05	1.66E+05	3.34E-01	9.34E-01	4.95E-01	-0.14	-0.03	0.11
A0A1D3JSM9	3 (0)	16.3	NA	NA	NA	NA	NA	NA	NA	NA	NA	NA	NA	NA	NA	NA	NA	NA
A0A1D3JSP6	28 (8)	206.4	5.61E-03	9.70E+05	1.19E+06	8.65E+05	7.58E+05	7.92E+05	7.36E+05	5.21E+05	5.87E+05	6.08E+05	5.61E-02	4.60E-03	1.35E-01	-0.41	-0.82	-0.41
A0A1D3JSP7	8 (2)	67.9	1.19E-01	1.79E+04	1.16E+04	3.15E+04	1.09E+04	1.14E+04	8.49E+03	1.30E+04	5.13E+03	6.56E+03	2.10E-01	1.28E-01	9.21E-01	**-0.99**	**-1.31**	-0.32
A0A1D3JSR6	2 (1)	9.9	2.16E-01	2.60E+03	5.41E+03	3.44E+03	3.61E+03	2.90E+03	6.80E+03	3.19E+01	2.37E+03	2.91E+03	9.00E-01	3.68E-01	2.16E-01	0.22	**-1.11**	**-1.32**
A0A1D3JSR8	18 (6)	113.2	1.36E-02	2.38E+05	2.07E+05	2.36E+05	2.85E+05	2.85E+05	2.64E+05	2.37E+05	1.86E+05	2.17E+05	3.93E-02	6.67E-01	1.41E-02	0.29	-0.09	-0.39
A0A1D3JSR9	14 (4)	86.8	6.26E-01	4.00E+04	4.74E+04	3.52E+04	3.83E+04	4.59E+04	6.14E+04	3.63E+04	4.28E+04	5.43E+04	6.00E-01	8.88E-01	8.57E-01	0.25	0.12	-0.13
A0A1D3JSV2	12 (3)	90.6	8.75E-02	5.07E+04	5.87E+04	5.66E+04	4.82E+04	4.89E+04	5.14E+04	5.26E+04	3.99E+04	3.73E+04	4.34E-01	7.52E-02	3.91E-01	-0.16	-0.36	-0.19

A0A1D3JSV4	8 (2)	38.1	3.21E-02	1.42E+04	1.02E+04	1.37E+04	1.72E+04	2.15E+04	1.41E+04	2.68E+04	1.84E+04	2.35E+04	2.70E-01	2.68E-02	2.31E-01	0.47	**0.85**	0.38
A0A1D3JSV7	16 (5)	113.4	3.33E-01	1.10E+05	1.13E+05	1.19E+05	9.74E+04	9.24E+04	1.29E+05	7.58E+04	1.05E+05	1.02E+05	8.17E-01	3.10E-01	6.04E-01	-0.10	-0.28	-0.18
A0A1D3JSW3	3 (1)	22.0	2.89E-01	2.42E+03	1.02E+04	2.76E+03	5.02E+03	6.98E+03	2.27E+04	0.00E+00	5.56E+03	2.78E+03	4.76E-01	8.94E-01	2.83E-01	**1.17**	**-0.89**	**-2.06**
A0A1D3JSW4	2 (1)	10.3	1.94E-01	4.72E+03	3.75E+03	3.94E+03	3.90E+03	3.41E+03	3.32E+03	3.81E+03	4.20E+03	4.83E+03	3.21E-01	9.23E-01	2.00E-01	-0.22	0.05	0.27
A0A1D3JSW6	27 (8)	250.3	9.97E-04	6.87E+05	5.54E+05	6.27E+05	1.12E+06	1.20E+06	1.08E+06	7.29E+05	7.84E+05	9.59E+05	8.24E-04	6.31E-02	1.06E-02	**0.86**	0.40	-0.46
A0A1D3JSX5	7 (2)	56.0	4.89E-02	6.73E+04	7.96E+04	7.70E+04	1.41E+05	1.21E+05	2.02E+05	1.17E+05	2.19E+05	2.42E+05	1.61E-01	4.47E-02	5.95E-01	**1.05**	**1.37**	0.32
A0A1D3JSX6	15 (3)	82.9	3.27E-01	2.83E+04	1.30E+04	2.31E+04	2.49E+04	3.45E+04	2.40E+04	3.24E+04	2.22E+04	4.16E+04	6.17E-01	3.03E-01	7.94E-01	0.37	0.58	0.21
A0A1D3JSY3	12 (3)	102.2	1.96E-01	5.98E+04	4.16E+04	6.24E+04	2.82E+04	3.79E+04	2.88E+04	6.12E+04	2.18E+04	4.50E+04	1.75E-01	5.59E-01	6.05E-01	-0.79	-0.36	0.43
A0A1D3JSY5	28 (1)	101.6	8.70E-01	1.45E+04	1.15E+04	1.15E+04	1.93E+04	2.16E+04	1.79E+04	0.00E+00	6.36E+04	0.00E+00	9.13E-01	8.73E-01	9.95E-01	0.65	0.76	0.11
A0A1D3JSZ2	3 (2)	15.7	5.19E-01	1.25E+04	1.68E+04	1.54E+04	1.65E+04	1.47E+04	2.52E+04	9.40E+03	1.96E+04	1.57E+04	5.78E-01	1.00E+00	5.74E-01	0.34	0.00	-0.34
A0A1D3JSZ5	2 (0)	13.0	NA	NA	NA	NA	NA	NA	NA	NA	NA	NA	NA	NA	NA	**NA**	**NA**	**NA**
A0A1D3JSZ7	2 (0)	13.4	NA	NA	NA	NA	NA	NA	NA	NA	NA	NA	NA	NA	NA	**NA**	**NA**	**NA**
A0A1D3JT01	3 (1)	17.0	3.77E-01	2.06E+03	6.55E+03	1.72E+03	6.03E+03	3.77E+03	1.72E+04	2.36E+02	3.50E+03	7.99E+03	4.13E-01	9.93E-01	4.68E-01	**1.38**	0.18	**-1.20**
A0A1D3JT07	5 (1)	28.5	1.29E-03	8.86E+03	6.80E+03	9.37E+03	4.23E+03	4.20E+03	4.57E+03	9.78E+03	8.33E+03	9.37E+03	3.85E-03	5.44E-01	1.47E-03	**-0.95**	0.13	**1.08**
A0A1D3JT12	14 (6)	96.8	6.92E-02	1.66E+05	1.64E+05	2.38E+05	1.22E+05	1.25E+05	1.19E+05	1.63E+05	1.25E+05	9.63E+04	8.36E-02	1.15E-01	9.66E-01	-0.63	-0.56	0.07
A0A1D3JT19	8 (1)	48.1	6.41E-01	1.81E+03	1.00E+04	3.25E+03	2.73E+03	1.77E+03	1.41E+04	1.44E+02	3.34E+03	3.78E+03	9.53E-01	7.94E-01	6.28E-01	0.30	**-1.05**	**-1.35**
A0A1D3JT24	3 (0)	14.9	NA	NA	NA	NA	NA	NA	NA	NA	NA	NA	NA	NA	NA	**NA**	**NA**	**NA**
A0A1D3JT28	16 (2)	88.6	7.27E-01	7.72E+04	6.38E+04	8.34E+04	7.03E+04	7.41E+04	6.68E+04	8.58E+04	6.87E+04	7.16E+04	7.90E-01	9.97E-01	7.46E-01	-0.09	0.01	0.10
A0A1D3JT33	5 (2)	44.8	2.39E-02	8.15E+04	6.53E+04	7.27E+04	7.24E+04	8.36E+04	6.58E+04	5.87E+04	3.19E+04	4.48E+04	9.96E-01	3.82E-02	3.43E-02	0.02	-0.70	-0.71
A0A1D3JT34	10 (6)	60.4	3.83E-01	9.34E+04	1.15E+05	1.01E+05	8.17E+04	8.76E+04	1.13E+05	9.65E+04	8.83E+04	7.82E+04	6.69E-01	3.57E-01	8.19E-01	-0.13	-0.23	-0.10
A0A1D3JT35	10 (5)	74.4	4.13E-01	3.16E+05	2.38E+05	2.68E+05	2.93E+05	3.14E+05	2.44E+05	3.29E+05	2.89E+05	3.15E+05	9.29E-01	4.05E-01	5.94E-01	0.05	0.18	0.13
A0A1D3JT43	16 (7)	113.0	4.33E-03	3.54E+05	3.68E+05	3.31E+05	2.89E+05	2.71E+05	2.48E+05	2.88E+05	2.79E+05	2.46E+05	6.71E-03	7.56E-03	9.92E-01	-0.38	-0.37	0.01
A0A1D3JT44	6 (0)	25.9	NA	NA	NA	NA	NA	NA	NA	NA	NA	NA	NA	NA	NA	**NA**	**NA**	**NA**
A0A1D3JT50	2 (0)	10.3	NA	NA	NA	NA	NA	NA	NA	NA	NA	NA	NA	NA	NA	**NA**	**NA**	**NA**
A0A1D3JT61	16 (6)	102.4	9.56E-01	3.88E+05	3.50E+05	4.53E+05	1.73E+05	3.37E+04	1.00E+06	5.42E+05	4.60E+05	3.93E+05	1.00E+00	9.60E-01	9.67E-01	0.02	0.23	0.21
A0A1D3JT64	6 (1)	44.0	2.24E-03	8.35E+03	6.06E+03	5.82E+03	2.61E+04	2.81E+04	2.12E+04	1.98E+04	2.90E+04	3.23E+04	4.96E-03	2.99E-03	8.52E-01	**1.90**	**2.00**	0.11
A0A1D3JT65	25 (10)	225.5	3.26E-03	1.90E+06	1.46E+06	1.75E+06	1.04E+06	1.08E+06	8.49E+05	1.22E+06	1.22E+06	1.29E+06	2.82E-03	2.23E-02	1.84E-01	-0.78	-0.46	0.33
A0A1D3JT69	4 (3)	23.2	1.23E-01	3.16E+04	2.73E+04	2.84E+04	1.98E+04	1.97E+04	2.07E+04	2.95E+04	1.91E+04	1.27E+04	1.54E-01	1.73E-01	9.95E-01	-0.54	-0.51	0.03
A0A1D3JT71	28 (8)	173.8	1.43E-01	1.31E+05	1.32E+05	1.18E+05	1.42E+05	1.58E+05	1.77E+05	1.24E+05	1.67E+05	1.38E+05	1.25E-01	5.09E-01	5.14E-01	0.32	0.17	-0.15
A0A1D3JT78	14 (5)	74.4	4.56E-01	1.72E+05	1.43E+05	1.86E+05	1.77E+05	1.89E+05	1.82E+05	1.88E+05	1.60E+05	1.56E+05	4.97E-01	9.97E-01	5.38E-01	0.13	0.01	-0.12
A0A1D3JT86	2 (0)	11.7	NA	NA	NA	NA	NA	NA	NA	NA	NA	NA	NA	NA	NA	**NA**	**NA**	**NA**
A0A1D3JT88	12 (2)	82.6	1.23E-03	9.13E+05	7.63E+05	9.34E+05	1.69E+06	1.80E+06	1.58E+06	2.14E+06	2.42E+06	3.08E+06	3.18E-02	9.81E-04	2.57E-02	**0.96**	**1.55**	0.59
A0A1D3JT92	31 (12)	247.7	1.48E-05	1.70E+06	1.54E+06	1.65E+06	3.85E+06	4.22E+06	4.22E+06	2.18E+06	2.61E+06	2.64E+06	1.23E-05	4.74E-03	1.45E-04	**1.33**	0.60	-0.73
A0A1D3JTA1	2 (0)	89.7	NA	NA	NA	NA	NA	NA	NA	NA	NA	NA	NA	NA	NA	**NA**	**NA**	**NA**
A0A1D3JTA7	21 (4)	121.5	3.52E-03	3.60E+04	3.50E+04	5.94E+04	7.88E+04	8.05E+04	7.77E+04	7.34E+04	7.27E+04	7.16E+04	3.91E-03	1.03E-02	6.13E-01	**0.85**	0.79	-0.06
A0A1D3JTC1	2 (0)	11.5	NA	NA	NA	NA	NA	NA	NA	NA	NA	NA	NA	NA	NA	**NA**	**NA**	**NA**
A0A1D3JTE0	13 (5)	86.3	6.95E-04	2.13E+05	1.86E+05	2.10E+05	2.72E+05	2.86E+05	2.41E+05	3.15E+05	3.46E+05	3.65E+05	2.70E-02	5.54E-04	1.25E-02	0.39	0.75	0.36
A0A1D3JTE5	15 (5)	82.5	8.96E-01	2.89E+05	2.30E+05	2.58E+05	2.69E+05	3.04E+05	2.03E+05	3.54E+05	2.26E+05	2.48E+05	1.00E+00	9.13E-01	9.13E-01	0.00	0.09	0.09
A0A1D3JTF4	23 (10)	149.5	7.55E-01	7.01E+05	5.95E+05	6.68E+05	7.10E+05	8.15E+05	6.17E+05	8.17E+05	6.32E+05	5.13E+05	7.94E-01	1.00E+00	7.88E-01	0.13	0.00	-0.13
A0A1D3JTF9	4 (1)	54.7	NA	NA	NA	NA	NA	NA	NA	NA	NA	NA	NA	NA	NA	**NA**	**NA**	**NA**
A0A1D3JTG6	4 (2)	21.6	7.62E-01	2.02E+04	1.36E+04	2.14E+04	1.86E+04	2.04E+04	2.00E+04	1.28E+04	2.05E+04	1.94E+04	8.94E-01	9.54E-01	7.46E-01	0.10	-0.07	-0.16
A0A1D3JTI3	2 (0)	9.4	NA	NA	NA	NA	NA	NA	NA	NA	NA	NA	NA	NA	NA	**NA**	**NA**	**NA**
A0A1D3JTJ5	5 (1)	24.7	3.09E-03	4.37E+03	4.71E+03	4.78E+03	8.34E+03	7.65E+03	1.13E+04	9.35E+03	9.92E+03	1.05E+04	8.30E-03	3.61E-03	6.85E-01	**0.98**	**0.89**	-0.09
A0A1D3JTK4	2 (1)	10.0	3.84E-02	9.18E+04	8.07E+04	1.00E+05	7.27E+04	7.67E+04	6.83E+04	7.88E+04	5.41E+04	5.21E+04	1.63E-01	3.36E-02	4.62E-01	-0.33	-0.56	-0.23
A0A1D3JTK8	14 (7)	107.6	8.86E-03	2.36E+05	2.58E+05	2.51E+05	2.36E+05	2.34E+05	2.68E+05	3.55E+05	4.19E+05	5.42E+05	9.99E-01	1.45E-02	1.38E-02	-0.01	0.82	0.83
A0A1D3JTL2	12 (4)	87.1	4.43E-01	9.60E+04	6.44E+04	7.12E+04	6.18E+04	7.09E+04	4.94E+04	8.57E+04	5.00E+04	4.94E+04	4.85E-01	5.24E-01	9.97E-01	-0.35	-0.32	0.02
A0A1D3JTM0	2 (0)	9.2	NA	NA	NA	NA	NA	NA	NA	NA	NA	NA	NA	NA	NA	**NA**	**NA**	**NA**
A0A1D3JTP0	5 (1)	26.8	4.49E-01	0.00E+00	4.56E+01	0.00E+00	0.00E+00	0.00E+00	0.00E+00	5.55E+02	0.00E+00	0.00E+00	9.94E-01	5.37E-01	4.85E-01	0.00	**3.61**	0.00
A0A1D3JTP5	9 (2)	51.8	4.98E-01	3.94E+04	4.49E+04	4.39E+04	2.99E+04	2.92E+04	4.25E+04	2.21E+04	4.20E+04	4.64E+04	4.80E-01	7.05E-01	9.13E-01	-0.33	-0.21	0.12
A0A1D3JTR0	5 (0)	24.0	NA	NA	NA	NA	NA	NA	NA	NA	NA	NA	NA	NA	NA	**NA**	**NA**	**NA**
A0A1D3JTS7	4 (0)	24.6	NA	NA	NA	NA	NA	NA	NA	NA	NA	NA	NA	NA	NA	**NA**	**NA**	**NA**
A0A1D3JTT2	4 (0)	20.3	NA	NA	NA	NA	NA	NA	NA	NA	NA	NA	NA	NA	NA	**NA**	**NA**	**NA**

A0A1D3JTT4	5 (1)	32.8	4.00E-01	8.32E+03	1.28E+04	1.22E+04	1.37E+04	1.15E+04	1.42E+04	4.26E+03	1.05E+04	1.34E+04	7.20E-01	7.90E-01	3.72E-01	0.24	-0.24	-0.49
A0A1D3JTT6	2 (0)	11.1	NA	NA	NA	NA	NA	NA	NA	NA	NA	NA	NA	NA	NA	**NA**	**NA**	**NA**
A0A1D3JTU6	5 (1)	23.3	9.67E-02	1.98E+04	1.30E+04	1.51E+04	1.90E+04	2.05E+04	1.15E+04	2.82E+04	3.49E+04	1.88E+04	9.74E-01	1.15E-01	1.52E-01	0.09	0.77	0.68
A0A1D3JTU9	12 (1)	56.8	NA	NA	NA	NA	NA	NA	NA	NA	NA	NA	NA	NA	NA	**NA**	**NA**	**NA**
A0A1D3JTV5	3 (2)	7.9	7.21E-02	1.55E+05	1.14E+05	1.48E+05	1.88E+05	2.10E+05	1.63E+05	1.71E+05	1.38E+05	1.47E+05	6.92E-02	7.34E-01	1.84E-01	0.43	0.13	-0.30
A0A1D3JTV9	5 (1)	19.9	6.60E-01	2.50E+05	2.02E+05	2.44E+05	2.60E+05	2.88E+05	1.92E+05	2.80E+05	2.50E+05	2.45E+05	8.63E-01	6.36E-01	9.10E-01	0.09	0.15	0.07
A0A1D3JTW0	18 (5)	106.1	2.04E-01	1.20E+05	1.01E+05	1.06E+05	8.77E+04	8.80E+04	1.00E+05	7.17E+04	1.06E+05	9.33E+04	2.83E-01	2.35E-01	9.88E-01	-0.25	-0.27	-0.02
A0A1D3JTW1	2 (0)	8.6	NA	NA	NA	NA	NA	NA	NA	NA	NA	NA	NA	NA	NA	**NA**	**NA**	**NA**
A0A1D3JTX7	3 (0)	14.6	NA	NA	NA	NA	NA	NA	NA	NA	NA	NA	NA	NA	NA	**NA**	**NA**	**NA**
A0A1D3JTY1	3 (0)	20.0	NA	NA	NA	NA	NA	NA	NA	NA	NA	NA	NA	NA	NA	**NA**	**NA**	**NA**
A0A1D3JTY9	2 (0)	14.5	NA	NA	NA	NA	NA	NA	NA	NA	NA	NA	NA	NA	NA	**NA**	**NA**	**NA**
A0A1D3JTZ0	5 (0)	24.4	NA	NA	NA	NA	NA	NA	NA	NA	NA	NA	NA	NA	NA	**NA**	**NA**	**NA**
A0A1D3JTZ7	2 (0)	4.9	NA	NA	NA	NA	NA	NA	NA	NA	NA	NA	NA	NA	NA	**NA**	**NA**	**NA**
A0A1D3JTZ9	6 (1)	41.6	7.04E-01	1.86E+03	4.55E+03	2.59E+03	1.80E+03	1.55E+03	4.48E+03	1.06E+03	2.47E+03	2.66E+03	9.33E-01	6.84E-01	8.74E-01	-0.20	-0.54	-0.34
A0A1D3JU04	5 (1)	41.2	7.87E-02	1.71E+05	1.20E+05	1.35E+05	1.56E+05	1.71E+05	1.22E+05	2.10E+05	1.67E+05	2.11E+05	9.27E-01	8.83E-02	1.42E-01	0.08	0.47	0.39
A0A1D3JU12	6 (3)	31.6	2.45E-01	4.66E+03	1.27E+04	5.96E+03	1.14E+04	1.35E+04	3.50E+04	2.51E+03	1.19E+04	1.34E+04	2.68E-01	9.75E-01	3.46E-01	**1.37**	0.26	**-1.11**
A0A1D3JU16	2 (0)	10.5	NA	NA	NA	NA	NA	NA	NA	NA	NA	NA	NA	NA	NA	**NA**	**NA**	**NA**
A0A1D3JU17	17 (2)	119.6	9.01E-03	3.07E+05	2.77E+05	3.56E+05	2.48E+05	2.49E+05	2.61E+05	2.23E+05	2.27E+05	1.96E+05	5.93E-02	7.67E-03	2.47E-01	-0.31	-0.54	-0.23
A0A1D3JU31	7 (5)	38.7	8.55E-03	7.21E+05	5.84E+05	6.62E+05	1.23E+06	1.37E+06	9.38E+05	1.09E+06	1.01E+06	9.91E+05	8.02E-03	3.54E-02	4.28E-01	**0.85**	0.65	-0.20
A0A1D3JU39	9 (2)	59.7	1.41E-02	3.70E+05	2.96E+05	3.68E+05	2.29E+05	2.42E+05	2.03E+05	3.28E+05	2.79E+05	2.58E+05	1.16E-02	1.81E-01	1.33E-01	-0.62	-0.26	0.36
A0A1D3JU43	7 (3)	53.9	1.56E-01	4.26E+04	4.08E+04	3.25E+04	2.44E+04	3.46E+04	2.32E+04	3.72E+04	2.16E+04	1.67E+04	2.59E-01	1.66E-01	9.33E-01	-0.49	-0.62	-0.12
A0A1D3JU50	8 (2)	54.2	1.58E-02	6.96E+04	6.04E+04	7.37E+04	8.87E+04	8.88E+04	9.69E+04	1.07E+05	8.69E+04	8.64E+04	2.89E-02	2.11E-02	9.61E-01	0.43	0.46	0.03
A0A1D3JU53	6 (2)	33.4	2.19E-01	3.76E+04	2.88E+04	4.81E+04	2.24E+04	2.54E+04	1.58E+04	5.29E+04	2.21E+04	3.05E+04	2.30E-01	9.44E-01	3.41E-01	**-0.85**	-0.12	0.73
A0A1D3JU60	2 (1)	34.7	NA	NA	NA	NA	NA	NA	NA	NA	NA	NA	NA	NA	NA	**NA**	**NA**	**NA**
A0A1D3JU80	26 (3)	182.3	3.65E-01	2.24E+04	3.03E+04	1.63E+04	2.11E+04	2.97E+04	6.39E+04	1.20E+04	3.17E+04	1.99E+04	4.65E-01	9.88E-01	3.94E-01	0.73	-0.12	**-0.85**
A0A1D3JU98	38 (9)	139.9	9.02E-02	3.15E+06	2.42E+06	3.01E+06	3.64E+06	3.92E+06	3.22E+06	6.62E+06	3.13E+06	6.33E+06	7.30E-01	8.52E-02	2.28E-01	0.33	**0.91**	0.58
A0A1D3JUA2	4 (1)	21.7	1.47E-01	3.20E+03	1.95E+03	1.52E+03	2.14E+03	1.77E+03	2.27E+03	2.69E+03	2.96E+03	4.28E+03	9.58E-01	2.31E-01	1.63E-01	-0.11	0.58	0.69
A0A1D3JUB3	35 (10)	227.6	1.62E-01	4.73E+05	4.26E+05	4.66E+05	3.53E+05	3.66E+05	3.56E+05	5.09E+05	3.61E+05	3.53E+05	1.43E-01	5.50E-01	5.27E-01	-0.34	-0.16	0.19
A0A1D3JUB5	12 (3)	81.7	7.61E-01	1.50E+05	1.37E+05	1.51E+05	1.38E+05	1.35E+05	1.23E+05	1.78E+05	1.29E+05	8.72E+04	8.05E-01	7.89E-01	9.99E-01	-0.15	-0.15	-0.01
A0A1D3JUB6	12 (4)	62.5	2.40E-01	3.36E+05	2.79E+05	3.26E+05	2.66E+05	2.74E+05	2.14E+05	3.29E+05	2.21E+05	2.14E+05	2.81E-01	3.11E-01	9.96E-01	-0.32	-0.30	0.02
A0A1D3JUC7	2 (0)	5.0	NA	NA	NA	NA	NA	NA	NA	NA	NA	NA	NA	NA	NA	**NA**	**NA**	**NA**
A0A1D3JUD1	9 (4)	48.4	5.53E-05	2.09E+05	1.85E+05	2.04E+05	2.42E+05	2.59E+05	2.54E+05	3.04E+05	3.07E+05	2.91E+05	1.68E-03	4.33E-05	2.53E-03	0.34	0.60	0.26
A0A1D3JUF4	4 (1)	25.0	2.34E-02	1.38E+04	1.48E+04	1.58E+04	3.17E+04	3.03E+04	3.68E+04	2.47E+04	4.78E+04	5.08E+04	8.95E-02	2.14E-02	5.07E-01	**1.15**	**1.47**	0.32
A0A1D3JUH6	2 (0)	11.5	NA	NA	NA	NA	NA	NA	NA	NA	NA	NA	NA	NA	NA	**NA**	**NA**	**NA**
A0A1D3JUH9	7 (0)	36.8	NA	NA	NA	NA	NA	NA	NA	NA	NA	NA	NA	NA	NA	**NA**	**NA**	**NA**
A0A1D3JUJ4	5 (1)	29.1	2.25E-01	3.53E+04	2.17E+04	2.42E+04	4.18E+04	5.03E+04	3.47E+04	6.26E+04	3.59E+04	3.20E+04	3.04E-01	2.58E-01	9.90E-01	0.64	0.68	0.04
A0A1D3JUJ8	5 (2)	30.5	1.52E-02	6.94E+04	4.16E+04	5.99E+04	3.92E+04	4.96E+04	3.30E+04	2.93E+04	1.48E+04	1.69E+04	2.18E-01	1.26E-02	1.23E-01	-0.49	**-1.49**	**-1.00**
A0A1D3JUN6	9 (1)	51.5	NA	NA	NA	NA	NA	NA	NA	NA	NA	NA	NA	NA	NA	**NA**	**NA**	**NA**
A0A1D3JUP1	3 (0)	17.6	NA	NA	NA	NA	NA	NA	NA	NA	NA	NA	NA	NA	NA	**NA**	**NA**	**NA**
A0A1D3JUP9	2 (0)	10.4	NA	NA	NA	NA	NA	NA	NA	NA	NA	NA	NA	NA	NA	**NA**	**NA**	**NA**
A0A1D3JUR6	2 (0)	9.5	NA	NA	NA	NA	NA	NA	NA	NA	NA	NA	NA	NA	NA	**NA**	**NA**	**NA**
A0A1D3JUR8	2 (2)	6.0	2.04E-04	2.59E+03	2.54E+03	3.14E+03	1.02E+04	9.46E+03	1.01E+04	8.74E+03	5.99E+03	8.43E+03	1.85E-04	1.37E-03	6.07E-02	**1.84**	**1.49**	-0.36
A0A1D3JUR9	2 (0)	10.7	NA	NA	NA	NA	NA	NA	NA	NA	NA	NA	NA	NA	NA	**NA**	**NA**	**NA**
A0A1D3JUS2	3 (0)	19.3	NA	NA	NA	NA	NA	NA	NA	NA	NA	NA	NA	NA	NA	**NA**	**NA**	**NA**
A0A1D3JUS8	13 (3)	94.1	8.60E-01	3.55E+05	1.57E+05	2.20E+05	2.16E+05	2.24E+05	2.13E+05	2.32E+05	2.05E+05	2.89E+05	8.74E-01	9.99E-01	8.91E-01	-0.16	-0.01	0.15
A0A1D3JUT5	5 (1)	23.4	4.70E-02	1.20E+05	8.63E+04	9.98E+04	1.05E+05	1.33E+05	7.75E+04	7.40E+04	4.09E+04	5.01E+04	9.84E-01	7.61E-02	6.12E-02	0.04	**-0.89**	**-0.93**
A0A1D3JUT7	6 (3)	48.3	1.78E-01	3.76E+05	3.01E+05	4.04E+05	2.69E+05	2.87E+05	2.05E+05	3.97E+05	2.92E+05	2.43E+05	1.58E-01	6.00E-01	5.22E-01	-0.50	-0.21	0.29
A0A1D3JUT9	9 (0)	57.9	NA	NA	NA	NA	NA	NA	NA	NA	NA	NA	NA	NA	NA	**NA**	**NA**	**NA**
A0A1D3JUU2	4 (0)	30.4	NA	NA	NA	NA	NA	NA	NA	NA	NA	NA	NA	NA	NA	**NA**	**NA**	**NA**
A0A1D3JUV1	13 (2)	78.6	5.35E-02	3.11E+04	2.49E+04	3.22E+04	1.51E+04	1.86E+04	1.31E+04	2.74E+04	1.14E+04	1.23E+04	6.44E-02	9.38E-02	9.53E-01	**-0.91**	-0.79	0.13
A0A1D3JUW4	9 (1)	54.1	6.89E-01	3.37E+04	5.56E+04	4.57E+04	4.50E+04	3.96E+04	7.30E+04	3.01E+04	5.80E+04	3.85E+04	8.13E-01	9.69E-01	6.81E-01	0.22	-0.09	-0.32
A0A1D3JUY1	2 (0)	21.4	NA	NA	NA	NA	NA	NA	NA	NA	NA	NA	NA	NA	NA	**NA**	**NA**	**NA**

A0A1D3JUY3	2 (1)	10.2	2.38E-01	9.34E+03	5.19E+03	5.13E+03	9.52E+03	9.82E+03	7.56E+03	8.48E+03	1.01E+04	7.73E+03	2.70E-01	3.21E-01	9.89E-01	0.45	0.42	-0.03
A0A1D3JV13	16 (4)	88.9	7.77E-03	3.41E+05	2.86E+05	2.93E+05	5.51E+05	6.59E+05	3.46E+05	1.55E+06	1.17E+06	8.29E+05	5.26E-01	7.75E-03	2.70E-02	0.76	**1.95**	**1.19**
A0A1D3JV15	3 (1)	34.7	NA	NA	NA	NA	NA	NA	NA	NA	NA	NA	NA	NA	NA	**NA**	**NA**	**NA**
A0A1D3JV23	3 (0)	14.4	NA	NA	NA	NA	NA	NA	NA	NA	NA	NA	NA	NA	NA	**NA**	**NA**	**NA**
A0A1D3JV29	4 (0)	20.5	NA	NA	NA	NA	NA	NA	NA	NA	NA	NA	NA	NA	NA	**NA**	**NA**	**NA**
A0A1D3JV34	11 (1)	69.6	1.08E-04	8.83E+03	5.79E+03	7.38E+03	1.08E+03	1.23E+03	1.10E+03	5.56E+00	1.03E+02	0.00E+00	3.29E-04	1.31E-04	3.41E-01	**-2.69**	**-7.67**	**-4.98**
A0A1D3JV37	2 (0)	8.0	NA	NA	NA	NA	NA	NA	NA	NA	NA	NA	NA	NA	NA	**NA**	**NA**	**NA**
A0A1D3JV41	17 (1)	85.0	6.60E-01	1.68E+05	1.28E+05	1.54E+05	1.34E+05	1.50E+05	1.11E+05	1.89E+05	1.40E+05	1.19E+05	6.98E-01	9.99E-01	7.17E-01	-0.18	-0.01	0.18
A0A1D3JV48	3 (0)	17.8	NA	NA	NA	NA	NA	NA	NA	NA	NA	NA	NA	NA	NA	**NA**	**NA**	**NA**
A0A1D3JV62	5 (0)	25.2	NA	NA	NA	NA	NA	NA	NA	NA	NA	NA	NA	NA	NA	**NA**	**NA**	**NA**
A0A1D3JV63	3 (0)	14.7	NA	NA	NA	NA	NA	NA	NA	NA	NA	NA	NA	NA	NA	**NA**	**NA**	**NA**
A0A1D3JV75	23 (8)	217.5	2.28E-03	7.25E+05	8.02E+05	8.59E+05	9.63E+05	9.74E+05	1.13E+06	5.49E+05	6.97E+05	5.71E+05	3.23E-02	6.54E-02	1.84E-03	0.36	-0.39	-0.76
A0A1D3JV78	4 (0)	16.9	NA	NA	NA	NA	NA	NA	NA	NA	NA	NA	NA	NA	NA	**NA**	**NA**	**NA**
A0A1D3JV86	19 (8)	127.1	4.01E-02	7.50E+05	6.80E+05	7.57E+05	5.87E+05	5.98E+05	6.01E+05	7.10E+05	6.34E+05	5.62E+05	3.73E-02	1.29E-01	6.08E-01	-0.29	-0.20	0.09
A0A1D3JV89	3 (0)	17.2	NA	NA	NA	NA	NA	NA	NA	NA	NA	NA	NA	NA	NA	**NA**	**NA**	**NA**
A0A1D3JV93	3 (0)	18.9	NA	NA	NA	NA	NA	NA	NA	NA	NA	NA	NA	NA	NA	**NA**	**NA**	**NA**
A0A1D3JVA1	26 (10)	166.4	5.11E-04	4.39E+05	3.67E+05	3.94E+05	1.31E+06	1.40E+06	1.45E+06	2.52E+06	2.92E+06	3.87E+06	5.43E-02	4.32E-04	4.80E-03	**1.80**	**2.96**	**1.16**
A0A1D3JVA7	25 (11)	165.5	8.43E-02	1.70E+06	1.53E+06	1.82E+06	1.84E+06	1.97E+06	1.69E+06	2.79E+06	2.74E+06	1.77E+06	8.63E-01	8.77E-02	1.71E-01	0.12	0.53	0.41
A0A1D3JVA8	14 (8)	96.9	5.98E-05	1.88E+05	1.94E+05	2.06E+05	5.40E+05	5.40E+05	6.73E+05	6.14E+05	6.19E+05	6.74E+05	1.59E-04	7.86E-05	4.52E-01	**1.58**	**1.70**	0.12
A0A1D3JVA9	4 (0)	19.4	NA	NA	NA	NA	NA	NA	NA	NA	NA	NA	NA	NA	NA	**NA**	**NA**	**NA**
A0A1D3JVB2	8 (2)	65.1	9.48E-01	1.20E+04	1.97E+04	1.36E+04	1.22E+04	1.39E+04	1.49E+04	2.36E+04	7.62E+03	1.27E+04	9.45E-01	9.94E-01	9.75E-01	-0.14	-0.05	0.09
A0A1D3JVB3	8 (1)	41.7	2.39E-03	1.09E+04	1.15E+04	1.15E+04	2.11E+04	2.02E+04	2.58E+04	2.16E+04	2.81E+04	3.01E+04	1.11E-02	2.27E-03	2.96E-01	**0.99**	**1.23**	0.25
A0A1D3JVC4	2 (1)	12.5	6.91E-01	1.33E+04	5.64E+03	6.78E+03	1.05E+04	1.24E+04	8.54E+03	1.09E+04	1.02E+04	8.76E+03	6.84E-01	8.13E-01	9.71E-01	0.29	0.22	-0.07
A0A1D3JVC5	6 (2)	37.8	3.95E-02	1.39E+04	1.12E+04	1.08E+04	3.00E+04	2.54E+04	2.28E+04	2.73E+04	3.49E+04	6.10E+04	2.99E-01	3.31E-02	2.60E-01	**1.12**	**1.78**	0.66
A0A1D3JVD0	7 (5)	66.6	4.48E-03	1.02E+05	1.10E+05	1.13E+05	4.07E+05	4.13E+05	4.53E+05	1.99E+05	4.41E+05	3.51E+05	4.14E-03	2.16E-02	3.19E-01	**1.97**	**1.61**	-0.36
A0A1D3JVD1	105 (28)	588.2	1.04E-02	1.13E+06	9.13E+05	1.04E+06	3.14E+06	3.35E+06	4.21E+06	4.57E+06	7.18E+06	1.03E+07	2.33E-01	8.75E-03	7.40E-02	**1.79**	**2.83**	**1.04**
A0A1D3JVD3	30 (6)	214.3	3.20E-06	4.29E+04	4.69E+04	4.73E+04	2.65E+05	2.51E+05	2.49E+05	2.86E+05	2.51E+05	2.40E+05	5.71E-06	5.07E-06	9.43E-01	**2.48**	**2.50**	0.02
A0A1D3JVD5	4 (0)	22.2	NA	NA	NA	NA	NA	NA	NA	NA	NA	NA	NA	NA	NA	**NA**	**NA**	**NA**
A0A1D3JVF7	3 (1)	14.2	7.32E-01	8.13E+01	1.52E+03	9.01E+02	4.44E+02	2.30E+02	1.09E+03	0.00E+00	4.84E+02	9.27E+02	8.58E-01	7.19E-01	9.63E-01	-0.50	-0.82	-0.33
A0A1D3JVI5	19 (6)	152.3	3.12E-03	8.28E+05	8.09E+05	9.32E+05	6.42E+05	6.53E+05	5.95E+05	5.92E+05	4.44E+05	3.52E+05	3.36E-02	2.55E-03	1.02E-01	-0.44	**-0.89**	-0.44
A0A1D3JVK4	23 (7)	147.8	8.91E-04	6.63E+05	5.68E+05	6.72E+05	9.08E+05	9.98E+05	8.36E+05	1.06E+06	1.28E+06	1.26E+06	2.30E-02	7.07E-04	2.12E-02	0.53	**0.92**	0.39
A0A1D3JVL2	15 (6)	109.4	1.35E-04	3.40E+05	4.04E+05	3.99E+05	2.25E+05	2.24E+05	2.06E+05	2.17E+05	2.24E+05	2.19E+05	2.32E-04	2.49E-04	9.93E-01	-0.81	-0.79	0.01
A0A1D3JVM1	28 (9)	146.2	1.29E-01	2.39E+05	2.02E+05	2.37E+05	2.44E+05	2.50E+05	2.42E+05	2.72E+05	2.36E+05	2.95E+05	5.39E-01	1.13E-01	4.43E-01	0.12	0.25	0.13
A0A1D3JVM7	2 (0)	54.7	NA	NA	NA	NA	NA	NA	NA	NA	NA	NA	NA	NA	NA	**NA**	**NA**	**NA**
A0A1D3JVN9	18 (3)	113.3	2.36E-01	2.43E+03	9.54E+03	2.43E+03	1.33E+04	7.41E+03	4.85E+04	4.23E+03	6.34E+03	6.96E+03	2.75E-01	9.95E-01	3.10E-01	**2.27**	0.28	**-1.98**
A0A1D3JVP1	15 (1)	94.5	5.31E-01	6.22E+04	4.53E+04	5.44E+04	5.33E+04	6.51E+04	4.39E+04	9.54E+04	4.14E+04	6.93E+04	1.00E+00	5.85E-01	5.90E-01	0.00	0.35	0.34
A0A1D3JVP7	16 (3)	74.2	3.21E-01	1.62E+05	1.31E+05	1.54E+05	1.78E+05	1.76E+05	1.55E+05	1.79E+05	1.60E+05	1.44E+05	2.96E-01	6.27E-01	7.74E-01	0.19	0.11	-0.08
A0A1D3JVQ0	6 (1)	38.1	6.28E-01	3.17E+02	4.76E+03	5.72E+02	6.35E+02	3.46E+02	1.34E+04	3.05E+02	9.16E+02	2.61E+03	7.31E-01	9.86E-01	6.39E-01	**1.34**	-0.56	**-1.91**
A0A1D3JVQ9	6 (1)	38.7	2.80E-03	1.43E+03	1.30E+03	1.02E+03	6.28E+03	7.59E+03	1.28E+04	1.86E+04	1.19E+04	1.45E+04	3.52E-02	2.27E-03	8.17E-02	**3.24**	**3.37**	0.13
A0A1D3JVR7	7 (3)	52.5	2.52E-01	1.53E+05	1.25E+05	1.39E+05	1.25E+05	1.39E+05	1.29E+05	1.71E+05	1.40E+05	1.42E+05	7.43E-01	5.45E-01	2.30E-01	-0.09	0.12	0.21
A0A1D3JVR8	3 (0)	19.2	NA	NA	NA	NA	NA	NA	NA	NA	NA	NA	NA	NA	NA	**NA**	**NA**	**NA**
A0A1D3JVS3	18 (4)	111.8	5.31E-02	2.54E+05	2.29E+05	2.60E+05	1.95E+05	1.93E+05	1.90E+05	2.47E+05	2.06E+05	1.80E+05	4.80E-02	1.78E-01	5.83E-01	-0.36	-0.23	0.13
A0A1D3JVS5	2 (1)	10.0	8.56E-02	0.00E+00	0.00E+00	0.00E+00	3.26E+02	2.75E+02	3.62E+02	1.12E+03	2.65E+03	7.03E+03	9.73E-01	1.03E-01	1.37E-01	**3.65**	**6.40**	**3.49**
A0A1D3JVS6	21 (7)	117.6	4.41E-02	3.60E+05	2.92E+05	3.50E+05	2.46E+05	2.55E+05	1.98E+05	3.40E+05	2.72E+05	2.59E+05	3.75E-02	3.85E-01	2.26E-01	-0.52	-0.20	0.32
A0A1D3JVT4	6 (2)	20.4	3.54E-01	1.47E+04	2.23E+04	1.35E+04	1.55E+04	2.06E+04	4.42E+04	1.62E+04	3.95E+04	4.19E+04	6.11E-01	3.32E-01	8.40E-01	0.67	**0.95**	0.28
A0A1D3JVT8	3 (0)	14.4	NA	NA	NA	NA	NA	NA	NA	NA	NA	NA	NA	NA	NA	**NA**	**NA**	**NA**
A0A1D3JVU1	3 (1)	25.0	2.87E-06	3.62E+03	3.41E+03	3.43E+03	2.05E+03	2.39E+03	2.20E+03	1.42E+03	1.32E+03	1.22E+03	5.17E-05	2.15E-06	3.90E-04	-0.66	**-1.40**	-0.74
A0A1D3JVU7	7 (1)	34.2	4.75E-01	3.93E+04	5.59E+04	4.85E+04	3.65E+04	4.42E+04	2.08E+04	5.91E+04	2.97E+04	2.38E+04	4.69E-01	6.46E-01	9.43E-01	-0.50	-0.35	0.15
A0A1D3JVV0	5 (1)	37.4	5.81E-01	2.87E+02	6.40E+03	6.98E+02	1.06E+03	6.89E+02	7.85E+03	0.00E+00	3.13E+02	1.32E+03	9.54E-01	7.37E-01	5.72E-01	0.38	**-2.18**	**-2.56**
A0A1D3JVV9	13 (6)	81.9	6.70E-03	1.04E+05	1.12E+05	1.08E+05	1.66E+05	1.61E+05	1.88E+05	1.43E+05	1.94E+05	1.70E+05	9.86E-03	1.20E-02	9.81E-01	0.67	0.65	-0.02
A0A1D3JVW1	7 (1)	44.3	3.63E-02	1.77E+05	1.46E+05	1.93E+05	1.08E+05	1.19E+05	8.99E+04	2.15E+05	1.67E+05	1.38E+05	5.53E-02	9.98E-01	5.17E-02	-0.71	0.01	0.72
A0A1D3JVX6	3 (1)	11.1	5.45E-02	6.83E+03	4.79E+03	5.66E+03	3.83E+03	3.75E+03	2.44E+03	4.77E+03	1.73E+03	2.32E+03	1.04E-01	6.19E-02	9.13E-01	-0.79	**-0.97**	-0.18

A0A1D3JVY0	2 (1)	6.4	3.00E-01	1.06E+05	1.03E+05	1.24E+05	9.41E+04	1.01E+05	8.92E+04	1.14E+05	9.76E+04	7.81E+04	3.28E-01	4.00E-01	9.84E-01	-0.23	-0.20	0.03
A0A1D3JVY4	3 (0)	20.1	NA	NA	NA	NA	NA	NA	NA	NA	NA	NA	NA	NA	NA	NA	NA	NA
A0A1D3JVY8	2 (1)	13.2	9.98E-01	0.00E+00	0.00E+00	2.93E+05	1.04E+05	9.27E+04	1.03E+05	1.08E+05	1.12E+05	8.61E+04	1.00E+00	9.98E-01	1.00E+00	0.04	0.07	0.03
A0A1D3JVZ1	5 (1)	28.5	5.32E-01	1.16E+04	1.06E+04	6.06E+03	8.99E+03	1.13E+04	3.23E+04	9.19E+03	8.78E+03	2.00E+04	5.07E-01	8.86E-01	7.70E-01	0.90	0.43	-0.47
A0A1D3JW15	3 (1)	21.8	9.21E-01	2.55E+04	2.58E+04	3.31E+04	2.44E+04	2.41E+04	3.01E+04	3.58E+04	2.57E+04	1.96E+04	9.13E-01	9.71E-01	9.83E-01	-0.10	-0.06	0.04
A0A1D3JW24	7 (1)	67.3	NA	NA	NA	NA	NA	NA	NA	NA	NA	NA	NA	NA	NA	NA	NA	NA
A0A1D3JW27	4 (2)	30.5	5.35E-01	2.34E+04	2.73E+04	2.54E+04	2.04E+04	2.00E+04	2.70E+04	2.33E+04	2.67E+04	2.05E+04	5.15E-01	7.45E-01	9.12E-01	-0.18	-0.11	0.07
A0A1D3JW33	5 (1)	56.8	NA	NA	NA	NA	NA	NA	NA	NA	NA	NA	NA	NA	NA	NA	NA	NA
A0A1D3JW46	24 (9)	186.9	1.05E-02	2.99E+05	2.46E+05	2.82E+05	4.77E+05	5.14E+05	4.26E+05	5.02E+05	4.21E+05	6.32E+05	2.89E-02	1.14E-02	7.01E-01	0.78	0.91	0.13
A0A1D3JW48	14 (7)	124.7	3.83E-01	1.13E+06	1.14E+06	1.27E+06	1.15E+06	1.14E+06	1.26E+06	1.06E+06	1.23E+06	9.18E+05	9.97E-01	4.63E-01	4.27E-01	0.01	-0.14	-0.15
A0A1D3JW51	3 (0)	15.6	NA	NA	NA	NA	NA	NA	NA	NA	NA	NA	NA	NA	NA	NA	NA	NA
A0A1D3JW53	23 (11)	155.8	3.34E-04	1.76E+05	1.52E+05	1.52E+05	2.57E+05	2.75E+05	2.59E+05	2.17E+05	1.86E+05	1.85E+05	2.86E-04	4.89E-02	2.90E-03	0.72	0.29	-0.43
A0A1D3JW69	7 (0)	38.4	NA	NA	NA	NA	NA	NA	NA	NA	NA	NA	NA	NA	NA	NA	NA	NA
A0A1D3JW75	2 (1)	11.7	3.28E-02	2.04E+04	2.99E+04	2.59E+04	3.99E+04	3.55E+04	5.61E+04	1.31E+04	2.93E+04	2.08E+04	7.72E-02	8.04E-01	3.50E-02	0.79	-0.27	-1.06
A0A1D3JW78	3 (0)	16.8	NA	NA	NA	NA	NA	NA	NA	NA	NA	NA	NA	NA	NA	NA	NA	NA
A0A1D3JW79	3 (0)	14.1	NA	NA	NA	NA	NA	NA	NA	NA	NA	NA	NA	NA	NA	NA	NA	NA
A0A1D3JW89	7 (2)	53.5	1.49E-02	8.37E+03	8.54E+03	6.99E+03	1.02E+04	1.02E+04	1.43E+04	7.20E+03	3.54E+03	4.93E+03	1.13E-01	2.32E-01	1.24E-02	0.54	-0.61	-1.14
A0A1D3JWA4	3 (1)	18.7	5.22E-01	1.12E+04	3.55E+04	1.60E+04	1.91E+04	1.56E+04	5.18E+04	4.32E+03	2.41E+04	1.44E+04	7.97E-01	8.51E-01	4.94E-01	0.46	-0.55	-1.02
A0A1D3JWA5	2 (1)	32.6	NA	NA	NA	NA	NA	NA	NA	NA	NA	NA	NA	NA	NA	NA	NA	NA
A0A1D3JWB7	2 (1)	15.3	4.21E-01	1.65E+02	2.56E+03	6.59E+02	7.75E+02	7.69E+02	4.20E+03	0.00E+00	6.62E+02	3.27E+02	7.71E-01	7.65E-01	3.91E-01	0.76	-1.78	-2.54
A0A1D3JWC1	8 (1)	45.8	NA	NA	NA	NA	NA	NA	NA	NA	NA	NA	NA	NA	NA	NA	NA	NA
A0A1D3JWC4	2 (0)	9.9	NA	NA	NA	NA	NA	NA	NA	NA	NA	NA	NA	NA	NA	NA	NA	NA
A0A1D3JWC5	30 (7)	215.6	1.47E-02	3.39E+05	4.13E+05	3.52E+05	2.70E+05	3.50E+05	3.46E+05	2.06E+05	2.47E+05	2.58E+05	3.61E-01	1.29E-02	7.45E-02	-0.19	-0.63	-0.44
A0A1D3JWD4	6 (1)	65.9	NA	NA	NA	NA	NA	NA	NA	NA	NA	NA	NA	NA	NA	NA	NA	NA
A0A1D3JWE2	2 (0)	10.2	NA	NA	NA	NA	NA	NA	NA	NA	NA	NA	NA	NA	NA	NA	NA	NA
A0A1D3JWF2	3 (1)	12.9	4.37E-01	4.25E+04	5.01E+04	4.94E+04	4.05E+04	4.15E+04	5.37E+04	4.49E+04	4.39E+04	3.22E+04	9.17E-01	4.24E-01	6.35E-01	-0.06	-0.23	-0.17
A0A1D3JWI4	10 (1)	40.8	1.16E-02	2.19E+03	2.94E+03	3.28E+03	1.94E+03	2.04E+03	1.32E+03	2.49E+02	1.52E+03	3.43E+02	1.43E-01	9.49E-03	1.33E-01	-0.67	-1.99	-1.33
A0A1D3JWJ9	3 (1)	65.3	NA	NA	NA	NA	NA	NA	NA	NA	NA	NA	NA	NA	NA	NA	NA	NA
A0A1D3JWK9	2 (1)	12.1	4.15E-01	5.22E+03	8.99E+03	3.79E+03	2.37E+03	3.27E+03	6.43E+03	4.96E+01	4.27E+03	5.06E+03	6.26E-01	4.00E-01	9.01E-01	-0.58	-0.94	-0.37
A0A1D3JWL0	3 (0)	15.6	NA	NA	NA	NA	NA	NA	NA	NA	NA	NA	NA	NA	NA	NA	NA	NA
A0A1D3JWL4	2 (1)	10.4	2.43E-01	5.61E+02	6.25E+02	4.19E+02	2.38E+03	1.88E+03	6.62E+02	5.49E+02	2.28E+03	1.04E+03	2.30E-01	4.60E-01	8.30E-01	1.62	1.27	-0.35
A0A1D3JWM0	6 (1)	56.5	NA	NA	NA	NA	NA	NA	NA	NA	NA	NA	NA	NA	NA	NA	NA	NA
A0A1D3JWM5	3 (0)	14.7	NA	NA	NA	NA	NA	NA	NA	NA	NA	NA	NA	NA	NA	NA	NA	NA
A0A1D3JWM7	2 (0)	11.1	NA	NA	NA	NA	NA	NA	NA	NA	NA	NA	NA	NA	NA	NA	NA	NA
A0A1D3JWN2	5 (0)	31.0	NA	NA	NA	NA	NA	NA	NA	NA	NA	NA	NA	NA	NA	NA	NA	NA
A0A1D3JWQ7	6 (1)	45.8	NA	NA	NA	NA	NA	NA	NA	NA	NA	NA	NA	NA	NA	NA	NA	NA
A0A1D3JWQ8	4 (1)	22.0	3.25E-03	2.55E+03	2.99E+03	2.76E+03	4.91E+03	4.93E+03	3.75E+03	4.63E+03	4.98E+03	5.63E+03	1.24E-02	3.25E-03	4.22E-01	0.71	0.88	0.17
A0A1D3JWR0	24 (5)	101.3	NA	NA	NA	NA	NA	NA	NA	NA	NA	NA	NA	NA	NA	NA	NA	NA
A0A1D3JWR4	3 (3)	21.7	3.30E-03	3.48E+04	4.30E+04	4.64E+04	4.01E+04	4.12E+04	4.50E+04	2.57E+04	2.72E+04	2.24E+04	9.75E-01	6.10E-03	4.96E-03	0.02	-0.72	-0.75
A0A1D3JWR5	2 (0)	9.9	NA	NA	NA	NA	NA	NA	NA	NA	NA	NA	NA	NA	NA	NA	NA	NA
A0A1D3JWT3	3 (0)	15.5	NA	NA	NA	NA	NA	NA	NA	NA	NA	NA	NA	NA	NA	NA	NA	NA
A0A1D3JWU0	4 (1)	67.9	NA	NA	NA	NA	NA	NA	NA	NA	NA	NA	NA	NA	NA	NA	NA	NA
A0A1D3JWU1	2 (1)	4.4	1.51E-01	1.76E+04	1.67E+04	1.98E+04	2.81E+04	2.83E+04	3.21E+04	1.82E+04	2.69E+04	4.11E+04	1.81E-01	2.15E-01	9.90E-01	0.71	0.67	-0.04
A0A1D3JWU2	8 (1)	74.7	NA	NA	NA	NA	NA	NA	NA	NA	NA	NA	NA	NA	NA	NA	NA	NA
A0A1D3JWU4	21 (9)	128.6	2.04E-01	7.44E+05	5.02E+05	6.73E+05	8.04E+05	8.94E+05	7.70E+05	8.44E+05	5.87E+05	6.09E+05	2.06E-01	9.07E-01	3.47E-01	0.36	0.09	-0.28
A0A1D3JWU5	3 (1)	16.1	2.63E-01	4.14E+04	4.20E+04	5.02E+04	3.42E+04	3.04E+04	3.80E+04	3.57E+04	4.80E+04	2.65E+04	2.60E-01	4.31E-01	9.04E-01	-0.38	-0.28	0.10
A0A1D3JWV4	14 (2)	89.1	1.24E-05	1.14E+04	8.89E+03	8.57E+03	1.70E+04	1.43E+04	1.75E+04	5.18E+04	4.58E+04	5.68E+04	1.22E-01	1.44E-05	4.05E-05	0.76	2.42	1.66
A0A1D3JWV7	4 (3)	29.7	2.33E-01	9.48E+04	8.00E+04	7.42E+04	6.60E+04	6.46E+04	6.72E+04	9.45E+04	7.77E+04	6.24E+04	2.25E-01	8.60E-01	4.22E-01	-0.33	-0.09	0.25
A0A1D3JWX1	3 (0)	16.4	NA	NA	NA	NA	NA	NA	NA	NA	NA	NA	NA	NA	NA	NA	NA	NA
A0A1D3JWX5	3 (1)	15.5	3.92E-01	9.25E+02	3.38E+02	2.15E+02	1.16E+03	6.82E+02	4.07E+02	1.56E+03	7.01E+02	7.34E+02	7.46E-01	3.63E-01	7.54E-01	0.60	1.02	0.42
A0A1D3JWX6	3 (2)	15.1	6.25E-01	1.08E+05	1.18E+05	1.37E+05	1.09E+05	1.10E+05	1.11E+05	9.76E+04	1.43E+05	1.25E+05	6.94E-01	9.98E-01	6.58E-01	-0.13	0.01	0.14
A0A1D3JWY3	3 (1)	45.8	NA	NA	NA	NA	NA	NA	NA	NA	NA	NA	NA	NA	NA	NA	NA	NA

A0A1D3JWY7	3 (2)	15.0	4.41E-01	9.46E+04	8.38E+04	1.06E+05	8.53E+04	8.33E+04	6.35E+04	1.52E+05	8.11E+04	8.28E+04	6.88E-01	8.71E-01	4.18E-01	-0.29	0.15	0.44
A0A1D3JWZ7	12 (4)	80.3	5.97E-02	3.16E+04	3.37E+04	3.16E+04	3.50E+04	3.40E+04	4.07E+04	4.70E+04	4.12E+04	6.75E+04	8.10E-01	6.11E-02	1.36E-01	0.18	0.69	0.51
A0A1D3JX08	12 (0)	70.4	NA	NA	NA	NA	NA	NA	NA	NA	NA	NA	NA	NA	NA	**NA**	**NA**	**NA**
A0A1D3JX27	3 (0)	16.5	NA	NA	NA	NA	NA	NA	NA	NA	NA	NA	NA	NA	NA	**NA**	**NA**	**NA**
A0A1D3JX36	12 (2)	101.5	NA	NA	NA	NA	NA	NA	NA	NA	NA	NA	NA	NA	NA	**NA**	**NA**	**NA**
A0A1D3JX42	3 (1)	9.2	1.54E-03	1.05E+04	7.75E+03	1.05E+04	1.04E+04	1.07E+04	8.16E+03	2.74E+04	3.30E+04	2.11E+04	9.99E-01	2.56E-03	2.67E-03	0.02	**1.50**	**1.48**
A0A1D3JX46	53 (15)	310.2	5.94E-01	1.02E+06	8.50E+05	9.98E+05	8.88E+05	9.20E+05	8.34E+05	1.03E+06	9.69E+05	8.08E+05	5.85E-01	9.58E-01	7.44E-01	-0.12	-0.03	0.09
A0A1D3JX49	35 (12)	264.2	2.36E-02	6.72E+05	6.12E+05	6.99E+05	5.79E+05	5.80E+05	5.41E+05	5.87E+05	4.83E+05	4.61E+05	1.17E-01	2.04E-02	3.85E-01	-0.22	-0.37	-0.15
A0A1D3JX53	3 (2)	11.3	9.31E-01	6.10E+04	8.93E+04	6.88E+04	6.93E+04	6.12E+04	1.06E+05	5.96E+04	9.43E+04	7.36E+04	9.25E-01	9.82E-01	9.78E-01	0.11	0.05	-0.06
A0A1D3JX55	2 (0)	5.7	NA	NA	NA	NA	NA	NA	NA	NA	NA	NA	NA	NA	NA	**NA**	**NA**	**NA**
A0A1D3JXE2	11 (4)	53.5	2.55E-02	1.06E+05	8.24E+04	1.05E+05	5.38E+04	5.69E+04	4.43E+04	8.67E+04	5.09E+04	8.80E+04	2.12E-02	2.29E-01	2.11E-01	**-0.92**	-0.38	0.54
A0A1D3JXE5	2 (0)	10.2	NA	NA	NA	NA	NA	NA	NA	NA	NA	NA	NA	NA	NA	**NA**	**NA**	**NA**
A0A1D3JXF1	3 (0)	11.2	NA	NA	NA	NA	NA	NA	NA	NA	NA	NA	NA	NA	NA	**NA**	**NA**	**NA**
A0A1D3JXF4	12 (4)	69.1	3.48E-03	2.01E+04	8.68E+03	1.44E+04	2.32E+04	2.70E+04	1.67E+04	5.07E+04	3.76E+04	5.92E+04	4.66E-01	3.56E-03	1.25E-02	0.63	**1.77**	**1.14**
A0A1D3JXG1	13 (10)	85.7	9.99E-04	4.14E+05	3.47E+05	4.06E+05	5.34E+05	6.12E+05	4.56E+05	8.88E+05	7.21E+05	9.02E+05	1.26E-01	8.86E-04	6.69E-03	0.46	**1.11**	0.65
A0A1D3JXG4	3 (0)	16.3	NA	NA	NA	NA	NA	NA	NA	NA	NA	NA	NA	NA	NA	**NA**	**NA**	**NA**
A0A1D3JXH4	2 (0)	16.2	NA	NA	NA	NA	NA	NA	NA	NA	NA	NA	NA	NA	NA	**NA**	**NA**	**NA**
A0A1D3JXH6	6 (1)	56.8	NA	NA	NA	NA	NA	NA	NA	NA	NA	NA	NA	NA	NA	**NA**	**NA**	**NA**
A0A1D3JXK1	5 (1)	19.8	2.22E-03	6.80E+04	6.93E+04	7.55E+04	2.94E+04	2.94E+04	2.46E+04	4.68E+04	3.35E+04	1.66E+04	2.90E-03	5.06E-03	8.25E-01	**-1.35**	**-1.13**	0.22
A0A1D3JXK8	2 (1)	9.7	1.16E-01	6.32E+03	1.50E+04	7.26E+03	1.63E+04	1.16E+04	2.99E+04	3.81E+03	7.20E+03	9.62E+03	2.27E-01	8.69E-01	1.19E-01	**1.02**	-0.47	**-1.49**
A0A1D3JXL3	4 (1)	39.0	5.51E-03	5.81E+03	3.72E+03	3.93E+03	7.44E+03	8.39E+03	5.44E+03	9.28E+03	9.07E+03	9.31E+03	6.14E-02	4.48E-03	1.19E-01	0.66	**1.04**	0.38
A0A1D3JXM3	12 (6)	123.5	4.27E-03	2.39E+06	2.49E+06	2.08E+06	2.08E+06	2.45E+06	1.95E+06	1.51E+06	1.14E+06	1.45E+06	6.74E-01	4.85E-03	1.18E-02	-0.10	-0.76	-0.66
A0A1D3JXN6	38 (5)	247.2	4.12E-03	1.09E+06	9.08E+05	9.90E+05	6.65E+05	7.46E+05	5.64E+05	6.81E+05	5.29E+05	4.48E+05	1.51E-02	4.14E-03	4.58E-01	-0.60	**-0.85**	-0.25
A0A1D3JXQ1	13 (6)	93.6	1.67E-01	5.35E+05	4.03E+05	4.50E+05	5.48E+05	6.31E+05	4.84E+05	6.12E+05	5.13E+05	5.88E+05	2.67E-01	1.80E-01	9.47E-01	0.26	0.30	0.04
A0A1D3JXQ3	2 (1)	12.0	7.96E-01	1.74E+03	5.90E+03	2.80E+03	3.45E+03	2.20E+03	1.04E+04	6.70E+01	7.59E+03	3.66E+03	8.05E-01	9.95E-01	8.55E-01	0.62	0.12	-0.50
A0A1D3JXQ4	6 (2)	39.8	4.16E-01	6.59E+03	7.12E+03	8.32E+03	9.49E+03	8.56E+03	8.96E+03	1.05E+04	6.87E+03	6.15E+03	4.03E-01	9.12E-01	6.16E-01	0.29	0.09	-0.20
A0A1D3JXR9	7 (3)	48.3	2.18E-02	1.68E+05	1.70E+05	1.72E+05	2.53E+05	2.43E+05	2.92E+05	2.07E+05	2.53E+05	3.04E+05	2.81E-02	4.02E-02	9.51E-01	0.63	0.58	-0.04
A0A1D3JXS0	12 (6)	76.0	2.21E-04	2.64E+05	2.64E+05	2.97E+05	1.78E+05	1.83E+05	1.68E+05	1.55E+05	1.32E+05	1.04E+05	1.61E-03	1.97E-04	5.42E-02	-0.64	**-1.08**	-0.44
A0A1D3JXT0	14 (3)	56.9	NA	NA	NA	NA	NA	NA	NA	NA	NA	NA	NA	NA	NA	**NA**	**NA**	**NA**
A0A1D3JXT3	11 (3)	54.7	NA	NA	NA	NA	NA	NA	NA	NA	NA	NA	NA	NA	NA	**NA**	**NA**	**NA**
A0A1D3JXT7	15 (2)	89.5	4.40E-02	6.71E+04	5.80E+04	6.73E+04	3.78E+04	4.20E+04	2.60E+04	6.01E+04	3.42E+04	2.83E+04	4.64E-02	1.00E-01	8.18E-01	**-0.86**	-0.65	0.21
A0A1D3JXU0	8 (2)	40.5	1.56E-01	7.04E+04	5.25E+04	6.22E+04	5.16E+04	5.64E+04	3.40E+04	7.98E+04	7.95E+04	5.19E+04	4.00E-01	6.89E-01	1.41E-01	-0.38	0.19	0.57
A0A1D3JXW0	12 (1)	45.7	NA	NA	NA	NA	NA	NA	NA	NA	NA	NA	NA	NA	NA	**NA**	**NA**	**NA**
A0A1D3JXW3	24 (6)	148.7	1.10E-01	3.13E+05	2.75E+05	3.15E+05	2.56E+05	2.76E+05	2.15E+05	3.68E+05	3.01E+05	2.83E+05	2.29E-01	8.33E-01	1.09E-01	-0.27	0.08	0.35
A0A1D3JXX3	2 (0)	10.4	NA	NA	NA	NA	NA	NA	NA	NA	NA	NA	NA	NA	NA	**NA**	**NA**	**NA**
A0A1D3JXY5	2 (1)	11.1	3.57E-03	4.10E+03	5.30E+03	3.49E+03	1.29E+03	2.18E+03	3.16E+03	2.44E+01	9.49E+02	5.94E+02	4.32E-02	2.89E-03	9.34E-02	**-0.96**	**-3.04**	**-2.08**
A0A1D3JY00	10 (1)	65.4	7.44E-01	5.36E+03	6.15E+02	4.70E+02	1.23E+03	4.66E+03	1.14E+03	1.17E+03	1.44E+03	8.39E+02	9.92E-01	8.17E-01	7.53E-01	0.12	**-0.90**	**-1.03**
A0A1D3JY17	7 (2)	53.6	1.03E-01	9.18E+03	1.01E+04	1.22E+04	1.29E+04	1.33E+04	1.56E+04	1.15E+04	1.36E+04	1.60E+04	1.25E-01	1.57E-01	9.82E-01	0.41	0.38	-0.03
A0A1D3JY18	10 (4)	65.8	6.87E-02	8.32E+05	6.53E+05	7.68E+05	6.00E+05	2.75E+05	5.55E+05	6.06E+05	5.51E+05	5.23E+05	6.41E-02	1.93E-01	6.78E-01	-0.65	-0.42	0.23
A0A1D3JY20	10 (1)	67.8	NA	NA	NA	NA	NA	NA	NA	NA	NA	NA	NA	NA	NA	**NA**	**NA**	**NA**
A0A1D3JY28	15 (7)	102.0	2.03E-04	1.82E+05	1.70E+05	1.82E+05	1.61E+05	1.73E+05	1.55E+05	3.06E+05	3.22E+05	3.82E+05	7.32E-01	4.73E-04	2.84E-04	-0.13	**0.92**	**1.05**
A0A1D3JY32	9 (1)	34.8	NA	NA	NA	NA	NA	NA	NA	NA	NA	NA	NA	NA	NA	**NA**	**NA**	**NA**
A0A1D3JY35	2 (0)	11.9	NA	NA	NA	NA	NA	NA	NA	NA	NA	NA	NA	NA	NA	**NA**	**NA**	**NA**
A0A1D3JY37	24 (7)	161.9	7.35E-02	4.38E+05	3.56E+05	4.35E+05	4.81E+05	5.40E+05	4.32E+05	4.02E+05	3.09E+05	3.90E+05	2.45E-01	5.79E-01	6.55E-02	0.24	-0.16	-0.40
A0A1D3JY38	2 (0)	16.5	NA	NA	NA	NA	NA	NA	NA	NA	NA	NA	NA	NA	NA	**NA**	**NA**	**NA**
A0A1D3JY39	49 (11)	412.3	1.20E-02	1.31E+06	1.05E+06	1.21E+06	1.29E+06	1.30E+06	1.32E+06	1.60E+06	1.81E+06	2.25E+06	7.85E-01	1.36E-02	2.93E-02	0.13	0.67	0.54
A0A1D3JY42	2 (1)	11.9	6.53E-02	6.28E+03	3.26E+03	3.23E+03	1.60E+03	4.40E+02	1.36E+03	3.44E+03	1.42E+03	7.29E+02	6.55E-02	1.52E-01	7.91E-01	**-1.91**	**-1.19**	0.72
A0A1D3JY45	12 (3)	74.2	3.16E-01	1.03E+04	9.54E+03	1.25E+04	8.68E+03	8.35E+03	9.46E+03	8.84E+03	1.06E+04	1.44E+04	4.58E-01	9.50E-01	3.24E-01	-0.29	0.06	0.35
A0A1D3JY51	4 (1)	21.7	3.97E-01	2.67E+04	2.17E+04	2.84E+04	2.56E+04	2.74E+04	2.12E+04	2.74E+04	1.67E+04	1.81E+04	9.66E-01	4.07E-01	5.32E-01	-0.05	-0.30	-0.25
A0A1D3JY52	4 (1)	27.0	2.45E-04	9.34E+03	8.70E+03	1.02E+04	1.00E+03	9.77E+02	9.92E+02	5.47E+03	2.34E+03	2.38E+03	2.28E-04	1.45E-03	8.60E-02	**-3.25**	**-1.47**	**1.78**
A0A1D3JY58	5 (1)	21.9	7.41E-02	2.45E+02	1.12E+03	1.35E+03	1.99E+03	1.81E+03	1.27E+03	0.00E+00	1.15E+03	1.87E+02	2.47E-01	5.79E-01	6.60E-02	**0.91**	**-1.02**	**-1.92**
A0A1D3JY66	23 (7)	146.8	1.54E-03	4.58E+05	4.61E+05	4.34E+05	2.74E+05	3.24E+05	2.98E+05	1.86E+05	2.94E+05	1.66E+05	1.18E-02	1.32E-03	1.21E-01	-0.59	**-1.07**	-0.47

A0A1D3JY68	5 (1)	35.4	2.36E-03	1.16E+05	8.93E+04	9.47E+04	1.32E+05	1.33E+05	1.04E+05	3.96E+05	2.36E+05	3.49E+05	8.34E-01	3.09E-03	5.33E-03	0.30	**1.71**	**1.41**
A0A1D3JY71	5 (3)	29.4	1.82E-01	8.09E+03	1.27E+04	1.19E+04	1.95E+04	1.51E+04	3.40E+04	3.48E+03	1.67E+04	1.72E+04	2.00E-01	9.65E-01	2.75E-01	**1.06**	0.19	**-0.87**
A0A1D3JY75	7 (1)	58.0	1.63E-04	1.75E+05	1.19E+05	1.44E+05	6.19E+05	7.34E+05	5.14E+05	1.42E+05	1.43E+05	1.62E+05	2.85E-04	9.98E-01	2.95E-04	**2.09**	0.03	**-2.06**
A0A1D3JY85	2 (2)	12.0	1.49E-01	7.52E+03	1.45E+04	7.89E+03	5.32E+03	6.57E+03	1.11E+04	5.00E+03	1.20E+03	5.81E+03	6.65E-01	1.34E-01	4.01E-01	-0.38	**-1.32**	**-0.94**
A0A1D3JY92	13 (6)	92.9	8.88E-03	9.79E+04	1.65E+05	1.43E+05	1.49E+05	1.42E+05	1.91E+05	5.75E+04	7.56E+04	5.63E+04	4.93E-01	3.28E-02	8.62E-03	0.25	**-1.10**	**-1.35**
A0A1D3JYA8	5 (1)	36.3	4.58E-02	3.98E+05	2.74E+05	3.48E+05	4.70E+05	6.46E+05	4.03E+05	7.57E+05	5.60E+05	5.48E+05	2.12E-01	3.94E-02	4.28E-01	0.57	**0.87**	0.30
A0A1D3JYB7	10 (4)	50.7	9.19E-03	9.82E+04	8.74E+04	9.64E+04	6.25E+04	6.23E+04	6.06E+04	6.99E+04	4.37E+04	3.01E+04	4.04E-02	8.45E-03	4.00E-01	-0.61	**-0.97**	-0.37
A0A1D3JYC9	10 (2)	49.8	2.32E-01	1.37E+05	1.36E+05	1.57E+05	1.32E+05	1.35E+05	1.43E+05	1.47E+05	1.08E+05	9.03E+04	9.13E-01	2.33E-01	3.81E-01	-0.06	-0.31	-0.25
A0A1D3JYD7	7 (1)	45.3	NA	NA	NA	NA	NA	NA	NA	NA	NA	NA	NA	NA	NA	**NA**	**NA**	**NA**
A0A1D3JYG5	8 (1)	67.5	2.05E-03	3.54E+04	3.47E+04	3.64E+04	3.71E+04	4.27E+04	4.12E+04	4.98E+04	6.35E+04	5.83E+04	4.20E-01	2.12E-03	7.45E-03	0.18	0.69	0.51
A0A1D3JYH3	5 (0)	21.7	NA	NA	NA	NA	NA	NA	NA	NA	NA	NA	NA	NA	NA	**NA**	**NA**	**NA**
A0A1D3JYH4	2 (0)	10.1	NA	NA	NA	NA	NA	NA	NA	NA	NA	NA	NA	NA	NA	**NA**	**NA**	**NA**
A0A1D3JYJ0	2 (0)	10.7	NA	NA	NA	NA	NA	NA	NA	NA	NA	NA	NA	NA	NA	**NA**	**NA**	**NA**
A0A1D3JYJ6	2 (1)	9.9	6.63E-01	5.39E+02	1.97E+03	3.80E+02	5.95E+02	8.47E+02	6.74E+02	5.37E+02	5.03E+02	3.72E+03	9.62E-01	8.01E-01	6.52E-01	-0.45	0.72	**1.17**
A0A1D3JYN7	5 (1)	78.4	NA	NA	NA	NA	NA	NA	NA	NA	NA	NA	NA	NA	NA	**NA**	**NA**	**NA**
A0A1D3JYP8	19 (5)	138.2	1.42E-02	7.31E+04	6.97E+04	7.80E+04	5.90E+04	5.99E+04	4.67E+04	6.17E+04	5.08E+04	4.98E+04	2.51E-02	1.98E-02	9.77E-01	-0.42	-0.44	-0.03
A0A1D3JYP9	6 (1)	31.0	1.88E-01	3.44E+03	1.21E+04	9.95E+03	7.90E+03	8.94E+03	1.71E+04	0.00E+00	4.61E+03	6.84E+03	7.22E-01	4.44E-01	1.71E-01	0.42	**-1.15**	**-1.57**
A0A1D3JYR3	32 (4)	78.3	NA	NA	NA	NA	NA	NA	NA	NA	NA	NA	NA	NA	NA	**NA**	**NA**	**NA**
A0A1D3JYR4	13 (2)	98.5	3.02E-02	8.23E+02	9.41E+02	1.39E+03	1.15E+03	1.04E+03	1.13E+03	1.65E+03	1.41E+03	1.91E+03	9.51E-01	3.79E-02	5.48E-02	0.05	0.63	0.58
A0A1D3JYS7	2 (1)	10.4	4.57E-01	1.39E+03	1.56E+03	1.53E+03	0.00E+00	1.72E+02	1.26E+02	7.62E+02	0.00E+00	1.41E+04	9.27E-01	6.45E-01	4.46E-01	**-3.91**	**1.73**	**5.64**
A0A1D3JYS9	4 (2)	20.3	4.52E-01	5.81E+04	3.93E+04	4.55E+04	5.55E+04	6.47E+04	4.54E+04	8.58E+04	4.74E+04	5.61E+04	7.95E-01	4.23E-01	7.77E-01	0.21	0.40	0.19
A0A1D3JYX3	13 (3)	74.3	5.69E-01	8.23E+04	6.91E+04	8.35E+04	7.89E+04	8.71E+04	7.24E+04	8.41E+04	6.42E+04	6.78E+04	9.86E-01	6.75E-01	5.84E-01	0.02	-0.12	-0.14
A0A1D3JYX6	2 (1)	17.6	4.87E-01	9.43E+04	6.12E+04	7.68E+04	8.77E+04	9.12E+04	7.35E+04	7.55E+04	7.61E+04	6.48E+04	7.67E-01	8.45E-01	4.59E-01	0.12	-0.10	-0.22
A0A1D3JYY5	39 (9)	103.6	NA	NA	NA	NA	NA	NA	NA	NA	NA	NA	NA	NA	NA	**NA**	**NA**	**NA**
A0A1D3JYZ2	2 (1)	11.0	1.51E-02	2.14E+05	1.73E+05	2.01E+05	3.40E+05	3.55E+05	3.20E+05	6.39E+05	3.97E+05	4.03E+05	1.61E-01	1.24E-02	1.63E-01	0.79	**1.29**	0.50
A0A1D3JYZ4	8 (1)	89.5	6.65E-01	0.00E+00	1.06E+04	1.18E+04	7.21E+03	8.63E+03	7.21E+03	1.09E+04	1.02E+04	9.22E+03	9.98E-01	6.94E-01	7.31E-01	0.04	0.43	0.39
A0A1D3JYZ7	15 (7)	75.3	5.22E-02	1.55E+05	1.28E+05	1.36E+05	1.68E+05	1.73E+05	1.43E+05	1.99E+05	1.62E+05	1.85E+05	3.03E-01	4.42E-02	3.41E-01	0.21	0.38	0.17
A0A1D3JYZ8	21 (4)	67.8	NA	NA	NA	NA	NA	NA	NA	NA	NA	NA	NA	NA	NA	**NA**	**NA**	**NA**
A0A1D3JZ06	5 (1)	30.0	2.86E-03	4.91E+04	4.09E+04	4.56E+04	7.42E+04	7.35E+04	7.04E+04	5.41E+04	6.81E+04	7.02E+04	2.58E-03	1.58E-02	2.38E-01	0.69	0.51	-0.18
A0A1D3JZ09	2 (0)	10.7	NA	NA	NA	NA	NA	NA	NA	NA	NA	NA	NA	NA	NA	**NA**	**NA**	**NA**
A0A1D3JZ13	4 (1)	18.9	4.22E-01	7.51E+03	8.40E+03	7.13E+03	5.43E+03	4.85E+03	7.78E+03	5.08E+03	7.80E+03	8.88E+03	4.16E-01	9.36E-01	5.96E-01	-0.35	-0.08	0.27
A0A1D3JZ21	4 (1)	46.2	3.42E-01	9.33E+04	1.09E+05	1.37E+05	1.17E+05	1.25E+05	1.22E+05	9.68E+04	1.15E+05	9.38E+04	7.87E-01	6.45E-01	3.16E-01	0.10	-0.15	-0.25
A0A1D3JZ23	14 (5)	84.1	4.08E-02	4.48E+04	4.93E+04	5.06E+04	8.67E+04	8.13E+04	1.17E+05	5.35E+04	9.33E+04	9.22E+04	3.70E-02	1.44E-01	5.56E-01	**0.98**	0.72	-0.25
A0A1D3JZ40	3 (1)	21.2	2.57E-01	2.56E+01	0.00E+00	7.60E+01	8.49E+01	3.45E+02	5.99E+02	0.00E+00	9.98E+01	4.57E+02	2.32E-01	6.55E-01	6.35E-01	**3.34**	**2.45**	**-0.89**
A0A1D3JZ43	17 (1)	5.0	NA	NA	NA	NA	NA	NA	NA	NA	NA	NA	NA	NA	NA	**NA**	**NA**	**NA**
A0A1D3JZ60	4 (1)	25.1	5.11E-01	2.27E+03	1.41E+03	1.77E+03	3.08E+03	3.26E+03	4.69E+03	8.08E+01	3.62E+03	7.07E+03	5.55E-01	5.83E-01	9.99E-01	**1.02**	**0.98**	-0.03
A0A1D3JZ66	7 (0)	40.5	NA	NA	NA	NA	NA	NA	NA	NA	NA	NA	NA	NA	NA	**NA**	**NA**	**NA**
A0A1D3JZ78	2 (0)	10.8	NA	NA	NA	NA	NA	NA	NA	NA	NA	NA	NA	NA	NA	**NA**	**NA**	**NA**
A0A1D3JZ92	2 (1)	49.5	NA	NA	NA	NA	NA	NA	NA	NA	NA	NA	NA	NA	NA	**NA**	**NA**	**NA**
A0A1D3JZA0	6 (1)	26.6	7.36E-03	5.78E+01	0.00E+00	2.20E+02	2.24E+02	4.62E+01	1.22E+02	7.31E+03	4.19E+03	1.16E+04	1.00E+00	1.17E-02	1.20E-02	0.50	**6.38**	**5.88**
A0A1D3JZE2	31 (13)	257.0	3.07E-02	8.05E+05	7.63E+05	7.61E+05	9.75E+05	9.14E+05	4.41E+05	4.14E+06	2.11E+06	1.83E+06	1.00E+00	4.56E-02	4.57E-02	0.00	**1.79**	**1.79**
A0A1D3JZG0	2 (1)	38.9	NA	NA	NA	NA	NA	NA	NA	NA	NA	NA	NA	NA	NA	**NA**	**NA**	**NA**
A0A1D3JZG1	9 (1)	58.5	NA	NA	NA	NA	NA	NA	NA	NA	NA	NA	NA	NA	NA	**NA**	**NA**	**NA**
A0A1D3JZI1	4 (1)	84.2	NA	NA	NA	NA	NA	NA	NA	NA	NA	NA	NA	NA	NA	**NA**	**NA**	**NA**
A0A1D3JZI9	6 (0)	36.5	NA	NA	NA	NA	NA	NA	NA	NA	NA	NA	NA	NA	NA	**NA**	**NA**	**NA**
A0A1D3JZJ4	5 (1)	24.7	1.94E-01	3.84E+03	4.04E+03	4.88E+03	7.78E+03	7.71E+03	9.02E+03	3.02E+03	1.09E+04	6.58E+03	1.80E-01	4.20E-01	7.70E-01	**0.94**	0.68	-0.26
A0A1D3JZL5	8 (1)	55.7	1.23E-01	1.03E+05	7.47E+04	9.20E+04	8.02E+04	9.97E+04	6.38E+04	1.31E+05	9.89E+04	1.09E+05	7.93E-01	2.72E-01	1.18E-01	-0.15	0.33	0.48
A0A1D3JZN3	4 (2)	27.7	9.07E-01	8.98E+04	7.62E+04	9.08E+04	9.43E+04	1.03E+05	7.14E+04	1.02E+05	8.59E+04	8.00E+04	9.17E-01	9.29E-01	9.99E-01	0.07	0.06	-0.01
A0A1D3JZN4	2 (0)	10.4	NA	NA	NA	NA	NA	NA	NA	NA	NA	NA	NA	NA	NA	**NA**	**NA**	**NA**
A0A1D3JZR0	8 (1)	44.3	9.82E-01	8.16E+03	7.05E+03	8.47E+03	8.17E+03	9.61E+03	5.64E+03	1.03E+04	7.45E+03	6.42E+03	9.98E-01	9.91E-01	9.81E-01	-0.02	0.03	0.05
A0A1D3JZS8	9 (1)	45.7	NA	NA	NA	NA	NA	NA	NA	NA	NA	NA	NA	NA	NA	**NA**	**NA**	**NA**
A0A1D3JZT1	6 (1)	56.3	NA	NA	NA	NA	NA	NA	NA	NA	NA	NA	NA	NA	NA	**NA**	**NA**	**NA**

A0A1D3JZT7	12 (3)	65.8	1.42E-02	1.55E+04	1.34E+04	1.04E+04	1.58E+04	1.36E+04	1.65E+04	2.00E+04	2.15E+04	1.82E+04	4.10E-01	1.28E-02	6.37E-02	0.22	0.60	0.38
A0A1D3JZV6	42 (5)	67.4	NA	NA	NA	NA	NA	NA	NA	NA	NA	NA	NA	NA	NA	**NA**	**NA**	**NA**
A0A1D3JZV9	4 (0)	18.1	NA	NA	NA	NA	NA	NA	NA	NA	NA	NA	NA	NA	NA	**NA**	**NA**	**NA**
A0A1D3JZX4	34 (5)	56.8	NA	NA	NA	NA	NA	NA	NA	NA	NA	NA	NA	NA	NA	**NA**	**NA**	**NA**
A0A1D3JZX8	4 (0)	13.5	NA	NA	NA	NA	NA	NA	NA	NA	NA	NA	NA	NA	NA	**NA**	**NA**	**NA**
A0A1D3JZZ0	9 (1)	45.0	6.11E-02	1.09E+04	2.39E+04	2.11E+04	1.56E+04	1.38E+04	2.20E+04	2.69E+04	3.16E+04	2.67E+04	9.28E-01	1.12E-01	7.00E-02	-0.12	0.61	0.73
A0A1D3JZZ6	11 (3)	73.7	5.32E-03	5.66E+04	5.64E+04	3.56E+04	6.63E+04	7.47E+04	9.88E+04	2.71E+04	3.07E+04	2.47E+04	4.95E-02	1.45E-01	4.39E-03	0.69	**-0.85**	**-1.54**
A0A1D3K008	22 (3)	67.4	NA	NA	NA	NA	NA	NA	NA	NA	NA	NA	NA	NA	NA	**NA**	**NA**	**NA**
A0A1D3K015	13 (2)	59.4	NA	NA	NA	NA	NA	NA	NA	NA	NA	NA	NA	NA	NA	**NA**	**NA**	**NA**
A0A1D3K049	10 (2)	63.7	1.40E-03	1.03E+04	5.89E+03	5.23E+03	1.92E+04	1.74E+04	1.65E+04	1.14E+04	1.34E+04	1.26E+04	1.11E-03	2.96E-02	3.35E-02	**1.31**	0.81	-0.50
A0A1D3K060	15 (2)	106.4	1.20E-01	1.56E+04	2.09E+04	1.66E+04	1.24E+04	1.29E+04	2.26E+04	1.12E+04	1.01E+04	1.02E+04	8.37E-01	1.19E-01	2.47E-01	-0.15	-0.75	-0.60
A0A1D3K076	10 (2)	59.8	1.77E-03	2.32E+03	2.37E+03	2.07E+03	1.94E+03	1.90E+03	2.38E+03	8.44E+02	1.35E+03	1.10E+03	6.41E-01	2.09E-03	4.87E-03	-0.12	**-1.04**	**-0.92**
A0A1D3K077	9 (4)	57.9	1.01E-01	7.82E+05	7.39E+05	8.52E+05	7.16E+05	7.43E+05	6.53E+05	7.20E+05	7.11E+05	6.19E+05	1.93E-01	1.05E-01	8.86E-01	-0.17	-0.21	-0.04
A0A1D3K079	5 (1)	33.4	8.44E-03	1.86E+02	1.11E+03	9.05E+02	1.41E+03	1.32E+03	1.34E+03	3.84E+03	2.82E+03	5.82E+03	7.01E-01	9.29E-03	2.30E-02	**0.89**	**2.50**	**1.62**
A0A1D3K096	20 (5)	53.7	NA	NA	NA	NA	NA	NA	NA	NA	NA	NA	NA	NA	NA	**NA**	**NA**	**NA**
A0A1D3K097	5 (3)	34.4	8.43E-02	3.14E+04	3.57E+04	2.55E+04	2.60E+04	4.02E+04	3.69E+04	2.05E+04	2.67E+04	1.70E+04	7.63E-01	2.03E-01	8.15E-02	0.15	-0.53	-0.68
A0A1D3K0A2	8 (2)	63.8	1.21E-01	3.81E+05	3.39E+05	3.90E+05	3.42E+05	3.44E+05	4.51E+05	3.02E+05	3.05E+05	3.11E+05	9.60E-01	1.95E-01	1.38E-01	0.03	-0.27	-0.31
A0A1D3K0A7	3 (0)	20.3	NA	NA	NA	NA	NA	NA	NA	NA	NA	NA	NA	NA	NA	**NA**	**NA**	**NA**
A0A1D3K0C0	2 (0)	10.3	NA	NA	NA	NA	NA	NA	NA	NA	NA	NA	NA	NA	NA	**NA**	**NA**	**NA**
A0A1D3K0C8	10 (3)	58.6	NA	NA	NA	NA	NA	NA	NA	NA	NA	NA	NA	NA	NA	**NA**	**NA**	**NA**
A0A1D3K0D4	2 (0)	10.1	NA	NA	NA	NA	NA	NA	NA	NA	NA	NA	NA	NA	NA	**NA**	**NA**	**NA**
A0A1D3K0E9	21 (3)	112.8	4.45E-01	1.21E+05	1.12E+05	1.21E+05	1.14E+05	1.29E+05	1.00E+05	1.40E+05	1.26E+05	1.14E+05	9.31E-01	6.27E-01	4.36E-01	-0.04	0.11	0.15
A0A1D3K0F0	3 (1)	23.6	6.28E-01	8.79E+03	5.48E+03	6.71E+03	8.38E+03	9.58E+03	5.62E+03	8.76E+03	8.11E+03	7.82E+03	7.81E-01	6.16E-01	9.54E-01	0.17	0.23	0.07
A0A1D3K0G0	2 (0)	10.6	NA	NA	NA	NA	NA	NA	NA	NA	NA	NA	NA	NA	NA	**NA**	**NA**	**NA**
A0A1D3K0I8	3 (1)	15.8	5.49E-01	4.51E+04	2.92E+04	3.18E+04	3.41E+04	4.14E+04	2.51E+04	5.80E+04	3.55E+04	3.45E+04	9.75E-01	6.76E-01	5.55E-01	-0.08	0.27	0.35
A0A1D3K0K8	4 (0)	18.4	NA	NA	NA	NA	NA	NA	NA	NA	NA	NA	NA	NA	NA	**NA**	**NA**	**NA**
A0A1D3K0L5	16 (9)	75.1	1.68E-02	1.48E+05	1.58E+05	1.66E+05	1.46E+05	1.53E+05	1.44E+05	1.23E+05	1.36E+05	1.38E+05	3.33E-01	1.45E-02	9.26E-02	-0.09	-0.25	-0.16
A0A1D3K0M4	3 (1)	17.5	1.13E-01	5.89E+03	5.67E+03	6.72E+03	5.09E+03	5.20E+03	3.94E+03	5.72E+03	2.59E+03	1.44E+03	4.93E-01	9.81E-02	4.33E-01	-0.36	**-0.91**	-0.54
A0A1D3K0N5	9 (2)	45.8	NA	NA	NA	NA	NA	NA	NA	NA	NA	NA	NA	NA	NA	**NA**	**NA**	**NA**
A0A1D3K0N9	2 (0)	10.9	NA	NA	NA	NA	NA	NA	NA	NA	NA	NA	NA	NA	NA	**NA**	**NA**	**NA**
A0A1D3K0P5	16 (6)	115.1	1.15E-01	1.50E+05	1.96E+05	1.97E+05	3.01E+05	2.80E+05	3.36E+05	1.35E+05	2.71E+05	3.24E+05	1.00E-01	4.67E-01	4.65E-01	0.76	0.43	-0.33
A0A1D3K0P6	19 (6)	58.7	NA	NA	NA	NA	NA	NA	NA	NA	NA	NA	NA	NA	NA	**NA**	**NA**	**NA**
A0A1D3K0Q4	18 (11)	128.0	5.96E-03	1.60E+05	1.52E+05	1.58E+05	3.00E+05	3.00E+05	3.70E+05	2.49E+05	2.89E+05	3.70E+05	7.36E-03	1.37E-02	8.30E-01	**1.04**	**0.95**	-0.09
A0A1D3K0Q5	2 (0)	10.4	NA	NA	NA	NA	NA	NA	NA	NA	NA	NA	NA	NA	NA	**NA**	**NA**	**NA**
A0A1D3K0Q8	2 (0)	15.7	NA	NA	NA	NA	NA	NA	NA	NA	NA	NA	NA	NA	NA	**NA**	**NA**	**NA**
A0A1D3K0Q9	3 (1)	16.2	5.52E-03	9.86E+01	8.99E+01	8.11E+01	2.03E+02	2.41E+02	2.90E+02	2.08E+02	1.94E+02	2.90E+02	7.35E-03	1.14E-02	9.07E-01	**1.63**	**1.13**	-0.49
A0A1D3K0S4	6 (2)	58.6	NA	NA	NA	NA	NA	NA	NA	NA	NA	NA	NA	NA	NA	**NA**	**NA**	**NA**
A0A1D3K0T2	4 (1)	69.5	NA	NA	NA	NA	NA	NA	NA	NA	NA	NA	NA	NA	NA	**NA**	**NA**	**NA**
A0A1D3K0T9	2 (1)	10.4	4.73E-02	4.04E+03	1.82E+03	2.99E+03	6.48E+03	6.50E+03	3.77E+03	3.25E+03	1.89E+03	3.10E+03	7.85E-02	9.76E-01	6.03E-02	**0.92**	-0.10	**-1.02**
A0A1D3K0V2	4 (2)	17.9	7.10E-02	6.85E+04	8.61E+04	8.71E+04	7.00E+04	7.34E+04	6.49E+04	8.01E+04	8.91E+04	9.11E+04	2.35E-01	5.86E-01	6.35E-02	-0.21	0.11	0.32
A0A1D3K0W5	2 (0)	6.0	NA	NA	NA	NA	NA	NA	NA	NA	NA	NA	NA	NA	NA	**NA**	**NA**	**NA**
A0A1D3K0Y3	2 (1)	11.3	7.80E-01	1.40E+04	6.03E+03	7.28E+03	7.28E+03	8.90E+03	4.39E+03	1.86E+04	3.00E+03	8.11E+03	8.70E-01	9.81E-01	7.75E-01	-0.41	0.12	0.53
A0A1D3K0Y4	7 (1)	57.5	5.08E-02	1.84E+03	1.63E+03	1.47E+03	3.09E+03	2.21E+03	4.90E+03	7.33E+02	1.89E+03	1.25E+03	1.05E-01	8.71E-01	5.55E-02	**1.04**	-0.35	**-1.40**
A0A1D3K0Y9	3 (1)	17.1	5.80E-01	2.00E+01	6.47E+02	7.78E+01	7.83E+02	2.59E+02	3.90E+03	0.00E+00	5.21E+02	4.37E+03	6.30E-01	6.36E-01	1.00E+00	**2.73**	**2.72**	-0.01
A0A1D3K0Z6	3 (1)	17.2	1.17E-01	2.22E+04	2.18E+04	2.44E+04	2.69E+04	2.59E+04	3.16E+04	2.36E+04	2.78E+04	2.12E+04	1.14E-01	8.10E-01	2.53E-01	0.30	0.09	-0.22
A0A1D3K106	2 (0)	9.2	NA	NA	NA	NA	NA	NA	NA	NA	NA	NA	NA	NA	NA	**NA**	**NA**	**NA**
A0A1D3K109	7 (1)	43.7	5.79E-03	4.46E+04	4.27E+04	4.47E+04	6.64E+04	6.11E+04	6.65E+04	3.22E+04	4.94E+04	4.72E+04	1.06E-02	9.73E-01	8.41E-03	0.56	-0.03	-0.59
A0A1D3K115	8 (2)	61.6	NA	NA	NA	NA	NA	NA	NA	NA	NA	NA	NA	NA	NA	**NA**	**NA**	**NA**
A0A1D3K116	14 (5)	100.7	7.81E-01	1.98E+04	1.98E+04	2.67E+04	2.32E+04	1.97E+04	2.07E+04	2.56E+04	3.52E+04	1.40E+04	9.85E-01	8.64E-01	7.79E-01	-0.06	0.17	0.23
A0A1D3K117	7 (5)	54.3	1.34E-02	2.13E+05	1.46E+05	1.93E+05	1.91E+05	2.34E+05	1.89E+05	3.18E+05	2.52E+05	3.15E+05	7.40E-01	1.46E-02	3.49E-02	0.15	0.68	0.53
A0A1D3K119	8 (3)	57.2	3.41E-01	5.70E+03	1.68E+04	8.47E+03	1.46E+04	1.17E+04	3.22E+04	1.03E+04	2.50E+04	3.26E+04	5.18E-01	3.36E-01	9.20E-01	**0.92**	**1.13**	0.21
A0A1D3K120	15 (3)	98.2	4.10E-02	5.81E+05	4.90E+05	5.55E+05	4.23E+05	4.43E+05	3.96E+05	4.85E+05	3.54E+05	3.14E+05	1.05E-01	4.13E-02	7.48E-01	-0.36	-0.49	-0.13

A0A1D3K125	21 (1)	155.6	7.70E-01	3.41E+04	2.39E+04	2.72E+04	2.74E+04	3.50E+04	2.65E+04	4.97E+04	1.24E+04	4.38E+04	9.91E-01	7.76E-01	8.42E-01	0.06	0.32	0.25
A0A1D3K127	2 (0)	11.3	NA	NA	NA	NA	NA	NA	NA	NA	NA	NA	NA	NA	NA	**NA**	**NA**	**NA**
A0A1D3K130	10 (2)	67.4	2.66E-01	2.05E+04	1.23E+04	1.73E+04	1.84E+04	1.86E+04	1.20E+04	2.45E+04	1.56E+04	3.01E+04	9.97E-01	3.39E-01	3.10E-01	-0.03	0.49	0.52
A0A1D3K133	25 (8)	161.4	6.39E-01	8.50E+05	6.99E+05	7.99E+05	7.00E+05	7.77E+05	6.46E+05	8.97E+05	6.90E+05	6.62E+05	6.15E-01	9.05E-01	8.50E-01	-0.15	-0.06	0.08
A0A1D3K135	19 (4)	133.7	5.44E-03	3.26E+05	3.44E+05	3.32E+05	2.25E+05	2.16E+05	2.63E+05	2.33E+05	2.29E+05	1.48E+05	1.96E-02	5.44E-03	4.84E-01	-0.51	-0.72	-0.21
A0A1D3K138	8 (0)	46.5	NA	NA	NA	NA	NA	NA	NA	NA	NA	NA	NA	NA	NA	**NA**	**NA**	**NA**
A0A1D3K160	2 (1)	17.2	4.34E-01	2.06E+03	1.61E+03	3.51E+04	1.50E+03	2.54E+03	1.04E+03	3.13E+03	1.70E+03	2.35E+03	4.76E-01	5.16E-01	9.97E-01	**-2.93**	**-2.43**	0.50
A0A1D3K167	8 (1)	42.7	2.74E-03	2.90E+03	3.78E+03	3.95E+03	1.76E+03	1.58E+03	2.18E+03	1.74E+03	1.23E+03	6.48E+02	1.22E-02	2.62E-03	3.24E-01	**-0.95**	**-1.55**	-0.60
A0A1D3K174	2 (0)	11.9	NA	NA	NA	NA	NA	NA	NA	NA	NA	NA	NA	NA	NA	**NA**	**NA**	**NA**
A0A1D3K181	5 (1)	58.5	6.53E-05	6.70E+03	7.02E+03	6.47E+03	7.12E+03	7.48E+03	8.55E+03	1.24E+04	1.35E+04	1.19E+04	2.23E-01	7.59E-05	2.17E-04	0.20	**0.91**	0.71
A0A1D3K184	23 (7)	207.4	1.52E-02	1.02E+06	8.85E+05	1.13E+06	5.70E+05	6.16E+05	4.88E+05	8.04E+05	7.48E+05	4.52E+05	1.51E-02	4.83E-02	6.06E-01	**-0.86**	-0.60	0.26
A0A1D3K197	52 (16)	408.3	1.94E-02	1.38E+06	1.20E+06	1.35E+06	1.08E+06	1.09E+06	1.01E+06	1.12E+06	1.01E+06	8.52E+05	5.14E-02	2.04E-02	7.29E-01	-0.31	-0.40	-0.09
A0A1D3K1A4	44 (12)	228.1	2.67E-01	3.56E+05	3.04E+05	3.44E+05	2.97E+05	3.25E+05	2.65E+05	3.35E+05	3.00E+05	2.90E+05	2.53E-01	4.88E-01	8.41E-01	-0.18	-0.12	0.06
A0A1D3K1B1	3 (1)	17.1	2.22E-02	2.50E+04	1.97E+04	2.54E+04	3.29E+04	3.66E+04	2.93E+04	3.60E+04	4.96E+04	6.29E+04	3.91E-01	1.93E-02	1.08E-01	0.49	**1.08**	0.59
A0A1D3K1B4	17 (6)	103.1	1.60E-02	1.20E+05	1.17E+05	1.27E+05	1.51E+05	1.47E+05	1.73E+05	1.60E+05	2.20E+05	1.84E+05	1.36E-01	1.32E-02	2.08E-01	0.38	0.63	0.26
A0A1D3K1B5	3 (0)	10.9	NA	NA	NA	NA	NA	NA	NA	NA	NA	NA	NA	NA	NA	**NA**	**NA**	**NA**
A0A1D3K1D3	6 (3)	48.8	7.43E-01	2.75E+04	5.56E+04	4.18E+04	4.57E+04	3.63E+04	8.86E+04	1.49E+04	5.89E+04	6.58E+04	7.31E-01	9.67E-01	8.62E-01	0.45	0.16	-0.29
A0A1D3K1E0	2 (1)	10.9	2.70E-02	3.49E+04	3.45E+04	3.92E+04	3.47E+04	3.48E+04	3.76E+04	5.08E+04	4.88E+04	3.95E+04	9.87E-01	4.48E-02	3.70E-02	-0.02	0.36	0.38
A0A1D3K1E4	15 (3)	136.3	7.72E-01	3.05E+05	1.69E+05	1.32E+05	1.73E+05	1.78E+05	1.60E+05	1.65E+05	1.67E+05	2.08E+05	7.62E-01	8.76E-01	9.73E-01	-0.25	-0.17	0.08
A0A1D3K1E8	9 (1)	51.7	NA	NA	NA	NA	NA	NA	NA	NA	NA	NA	NA	NA	NA	**NA**	**NA**	**NA**
A0A1D3K1F0	6 (1)	36.4	2.82E-01	3.08E+03	1.42E+03	2.61E+03	3.00E+03	3.74E+03	3.78E+03	1.05E+04	0.00E+00	1.42E+04	9.44E-01	2.89E-01	4.22E-01	0.57	**1.80**	**1.23**
A0A1D3K1G9	28 (5)	67.4	NA	NA	NA	NA	NA	NA	NA	NA	NA	NA	NA	NA	NA	**NA**	**NA**	**NA**
A0A1D3K1I0	7 (1)	39.0	5.86E-01	5.70E+01	7.04E+03	3.35E+01	9.04E+01	2.94E+01	3.14E+03	0.00E+00	0.00E+00	4.02E+02	8.15E-01	5.61E-01	8.93E-01	**-1.13**	**-4.15**	**-3.02**
A0A1D3K1I8	12 (4)	48.3	NA	NA	NA	NA	NA	NA	NA	NA	NA	NA	NA	NA	NA	**NA**	**NA**	**NA**
A0A1D3K1J2	2 (0)	9.7	NA	NA	NA	NA	NA	NA	NA	NA	NA	NA	NA	NA	NA	**NA**	**NA**	**NA**
A0A1D3K1J5	11 (6)	87.5	1.61E-01	3.58E+05	2.95E+05	3.33E+05	2.73E+05	2.91E+05	2.26E+05	4.52E+05	3.06E+05	3.20E+05	3.57E-01	7.75E-01	1.51E-01	-0.32	0.13	0.45
A0A1D3K1K2	7 (2)	49.3	8.06E-02	6.57E+04	6.72E+04	6.45E+04	4.35E+04	4.66E+04	6.16E+04	5.61E+04	6.00E+04	4.63E+04	8.08E-02	1.79E-01	8.12E-01	-0.38	-0.28	0.10
A0A1D3K1K6	9 (3)	79.4	8.16E-02	1.92E+05	1.80E+05	2.01E+05	1.58E+05	1.76E+05	1.72E+05	1.89E+05	1.74E+05	1.74E+05	7.03E-02	3.45E-01	4.60E-01	-0.18	-0.09	0.08
A0A1D3K1L8	3 (1)	56.3	NA	NA	NA	NA	NA	NA	NA	NA	NA	NA	NA	NA	NA	**NA**	**NA**	**NA**
A0A1D3K1P0	4 (1)	20.7	3.01E-02	1.18E+03	1.34E+03	3.85E+02	6.19E+00	5.14E-01	1.66E-01	5.39E+02	2.70E-01	0.00E+00	3.25E-02	7.03E-02	8.10E-01	**-8.72**	**-2.43**	**6.29**
A0A1D3K1Q3	2 (1)	9.6	5.42E-04	1.43E+03	2.08E+03	1.78E+03	9.00E+02	7.39E+02	1.12E+03	6.59E+01	3.62E+02	7.65E+01	1.14E-02	4.30E-04	1.95E-02	**-0.94**	**-3.39**	**-2.45**
A0A1D3K1R8	2 (1)	10.8	3.19E-02	1.20E+04	1.55E+04	1.48E+04	1.37E+04	1.33E+04	1.71E+04	5.24E+03	9.75E+03	1.07E+04	9.45E-01	5.86E-02	3.95E-02	0.06	-0.72	-0.78
A0A1D3K1T5	3 (1)	20.4	2.90E-02	2.74E+04	2.62E+04	3.23E+04	5.07E+04	5.10E+04	4.60E+04	6.03E+04	5.26E+04	3.49E+04	4.34E-02	4.31E-02	1.00E+00	0.78	0.78	0.00
A0A1D3K1U5	26 (6)	167.6	4.24E-04	3.75E+04	3.13E+04	3.10E+04	3.68E+04	4.09E+04	2.88E+04	6.34E+04	7.82E+04	7.76E+04	9.05E-01	6.39E-04	8.75E-04	0.09	**1.13**	**1.04**
A0A1D3K1V3	6 (1)	37.3	8.80E-05	3.91E+04	3.60E+04	4.50E+04	1.00E+05	1.04E+05	8.99E+04	5.96E+04	6.93E+04	7.19E+04	6.94E-05	4.57E-03	2.18E-03	**1.29**	0.74	-0.55
A0A1D3K1V5	7 (1)	63.9	4.42E-04	2.67E+04	2.51E+04	2.44E+04	3.58E+04	3.24E+04	3.74E+04	4.33E+04	5.07E+04	4.33E+04	1.50E-02	3.49E-04	1.05E-02	0.47	**0.85**	0.38
A0A1D3K1V7	7 (1)	62.6	2.70E-02	6.02E+04	7.19E+04	6.78E+04	1.15E+05	1.17E+05	1.39E+05	9.77E+04	1.61E+05	1.85E+05	9.55E-02	2.50E-02	5.52E-01	**0.89**	**1.15**	0.26
A0A1D3K1V9	10 (1)	64.7	3.79E-01	1.95E+03	3.61E+03	3.60E+03	1.80E+03	1.95E+03	2.38E+03	9.64E+02	5.61E+03	5.79E+03	7.52E-01	7.30E-01	3.50E-01	-0.58	0.43	**1.01**
A0A1D3K1W1	4 (1)	45.0	1.15E-01	1.97E+03	5.24E+03	2.94E+03	8.56E+03	6.10E+03	1.81E+04	7.92E+02	5.93E+03	4.94E+03	1.40E-01	9.88E-01	1.70E-01	**1.69**	0.20	**-1.49**
A0A1D3K1Y7	44 (7)	218.7	7.90E-02	1.73E+07	1.35E+07	1.70E+07	1.11E+07	1.28E+07	9.64E+06	2.15E+07	1.87E+07	1.30E+07	1.99E-01	7.37E-01	7.55E-02	-0.51	0.16	0.67
A0A1D3K1Y8	10 (1)	55.3	1.07E-02	2.21E+03	1.46E+03	2.43E+03	1.45E+03	1.46E+03	1.06E+03	2.88E+03	3.26E+03	2.40E+03	1.60E-01	1.08E-01	8.76E-03	-0.62	0.49	**1.11**
A0A1D3K202	5 (0)	34.6	NA	NA	NA	NA	NA	NA	NA	NA	NA	NA	NA	NA	NA	**NA**	**NA**	**NA**
A0A1D3K205	15 (5)	56.0	NA	NA	NA	NA	NA	NA	NA	NA	NA	NA	NA	NA	NA	**NA**	**NA**	**NA**
A0A1D3K207	12 (3)	60.1	8.45E-02	3.19E+04	2.64E+04	3.35E+04	2.20E+04	2.26E+04	1.78E+04	3.17E+04	1.97E+04	2.67E+04	7.27E-02	4.48E-01	3.67E-01	-0.56	-0.23	0.32
A0A1D3K218	39 (12)	260.2	2.24E-03	3.05E+05	3.32E+05	3.12E+05	2.62E+05	2.68E+05	3.00E+05	2.30E+05	2.32E+05	1.93E+05	9.66E-02	1.85E-03	2.27E-02	-0.19	-0.53	-0.34
A0A1D3K222	13 (2)	71.6	2.03E-01	1.99E+05	1.78E+05	1.99E+05	1.74E+05	1.72E+05	1.67E+05	1.92E+05	1.69E+05	1.46E+05	2.81E-01	2.33E-01	9.87E-01	-0.17	-0.18	-0.02
A0A1D3K227	6 (3)	28.4	NA	NA	NA	NA	NA	NA	NA	NA	NA	NA	NA	NA	NA	**NA**	**NA**	**NA**
A0A1D3K228	19 (6)	134.2	1.51E-01	4.53E+05	3.61E+05	4.01E+05	2.93E+05	3.24E+05	2.12E+05	3.96E+05	3.02E+05	1.86E+05	1.65E-01	2.43E-01	9.50E-01	-0.55	-0.46	0.09
A0A1D3K241	2 (1)	13.7	1.38E-01	1.22E+05	1.14E+05	1.34E+05	1.04E+05	1.02E+05	9.29E+04	1.24E+05	9.53E+04	8.43E+04	1.67E-01	1.99E-01	9.89E-01	-0.30	-0.28	0.02
A0A1D3K244	3 (2)	20.9	1.01E-01	2.89E+04	1.77E+04	2.11E+04	3.12E+04	3.64E+04	3.24E+04	4.48E+04	2.55E+04	3.89E+04	2.08E-01	1.03E-01	8.47E-01	0.56	0.69	0.13
A0A1D3K251	2 (1)	10.9	3.62E-03	3.36E+04	2.83E+04	3.36E+04	7.00E+04	7.08E+04	6.73E+04	5.08E+04	6.00E+04	7.91E+04	4.18E-03	9.80E-03	6.82E-01	**1.12**	**0.99**	-0.13
A0A1D3K261	11 (2)	68.4	3.74E-01	6.96E+04	1.19E+05	6.43E+04	6.66E+04	7.70E+04	1.25E+05	3.18E+04	7.54E+04	6.33E+04	9.71E-01	5.01E-01	3.89E-01	0.09	-0.57	-0.66

A0A1D3K275	8 (2)	54.8	7.55E-01	3.36E+04	3.52E+04	4.37E+04	3.17E+04	2.99E+04	4.68E+04	3.04E+04	3.84E+04	3.14E+04	9.64E-01	7.41E-01	8.76E-01	-0.06	-0.17	-0.11
A0A1D3K278	21 (5)	69.4	NA	NA	NA	NA	NA	NA	NA	NA	NA	NA	NA	NA	NA	**NA**	**NA**	**NA**
A0A1D3K280	18 (7)	118.7	1.45E-01	2.18E+05	2.46E+05	2.20E+05	2.49E+05	2.39E+05	3.20E+05	2.49E+05	3.13E+05	3.22E+05	3.83E-01	1.31E-01	6.77E-01	0.24	0.37	0.13
A0A1D3K286	3 (0)	24.1	NA	NA	NA	NA	NA	NA	NA	NA	NA	NA	NA	NA	NA	**NA**	**NA**	**NA**
A0A1D3K290	9 (3)	51.5	NA	NA	NA	NA	NA	NA	NA	NA	NA	NA	NA	NA	NA	**NA**	**NA**	**NA**
A0A1D3K298	6 (1)	63.6	NA	NA	NA	NA	NA	NA	NA	NA	NA	NA	NA	NA	NA	**NA**	**NA**	**NA**
A0A1D3K2A7	5 (0)	25.3	NA	NA	NA	NA	NA	NA	NA	NA	NA	NA	NA	NA	NA	**NA**	**NA**	**NA**
A0A1D3K2A9	2 (1)	11.2	5.62E-03	4.74E+03	3.62E+03	4.31E+03	6.35E+03	5.19E+03	6.06E+03	6.49E+03	7.44E+03	6.29E+03	3.40E-02	4.90E-03	2.47E-01	0.47	0.67	0.20
A0A1D3K2B2	8 (4)	61.6	2.76E-05	4.00E+05	4.09E+05	3.76E+05	2.90E+05	2.59E+05	2.96E+05	2.00E+05	2.21E+05	1.97E+05	4.23E-04	2.22E-05	3.51E-03	-0.49	**-0.94**	-0.45
A0A1D3K2B6	2 (0)	14.3	NA	NA	NA	NA	NA	NA	NA	NA	NA	NA	NA	NA	NA	**NA**	**NA**	**NA**
A0A1D3K2B9	4 (1)	15.2	6.70E-01	2.36E+05	1.89E+05	2.19E+05	1.93E+05	2.24E+05	1.43E+05	2.74E+05	1.73E+05	1.90E+05	6.95E-01	9.97E-01	7.39E-01	-0.20	-0.02	0.19
A0A1D3K2C5	6 (0)	44.4	NA	NA	NA	NA	NA	NA	NA	NA	NA	NA	NA	NA	NA	**NA**	**NA**	**NA**
A0A1D3K2C8	8 (1)	64.7	NA	NA	NA	NA	NA	NA	NA	NA	NA	NA	NA	NA	NA	**NA**	**NA**	**NA**
A0A1D3K2E2	2 (0)	17.8	NA	NA	NA	NA	NA	NA	NA	NA	NA	NA	NA	NA	NA	**NA**	**NA**	**NA**
A0A1D3K2F0	2 (1)	29.2	5.48E-01	1.18E+05	3.41E+05	1.78E+05	1.91E+05	2.67E+05	5.07E+05	4.16E+04	3.48E+05	1.93E+05	6.50E-01	9.88E-01	5.67E-01	0.60	-0.13	-0.73
A0A1D3K2F3	3 (1)	17.1	1.05E-01	3.88E+02	1.90E+03	1.33E+03	8.57E+01	1.29E+02	1.05E+03	0.00E+00	2.30E+02	0.00E+00	2.61E-01	9.81E-02	7.32E-01	**-1.52**	**-3.97**	**-2.46**
A0A1D3K2F5	10 (3)	45.9	NA	NA	NA	NA	NA	NA	NA	NA	NA	NA	NA	NA	NA	**NA**	**NA**	**NA**
A0A1D3K2G0	12 (5)	99.4	3.71E-01	3.57E+05	2.18E+05	3.04E+05	4.62E+05	5.00E+05	3.88E+05	1.00E+06	2.06E+05	5.57E+05	7.09E-01	3.42E-01	7.62E-01	0.62	**1.01**	0.39
A0A1D3K2G3	6 (1)	26.0	2.10E-03	1.76E+04	1.31E+04	1.87E+04	4.89E+03	5.57E+03	2.50E+03	9.29E+03	4.13E+03	7.13E+03	2.23E-03	7.17E-03	4.67E-01	-1.93	-1.27	0.66
A0A1D3K2G8	6 (0)	33.4	NA	NA	NA	NA	NA	NA	NA	NA	NA	NA	NA	NA	NA	NA	NA	NA
A0A1D3K2H4	15 (2)	56.8	NA	NA	NA	NA	NA	NA	NA	NA	NA	NA	NA	NA	NA	NA	NA	NA
A0A1D3K2H8	11 (3)	64.6	9.83E-01	5.78E+04	5.49E+04	6.53E+04	5.30E+04	5.55E+04	6.76E+04	6.76E+04	5.51E+04	5.66E+04	9.93E-01	9.97E-01	9.81E-01	-0.02	0.01	0.03
A0A1D3K2I0	21 (5)	132.6	9.52E-01	2.35E+05	1.65E+05	1.99E+05	1.94E+05	2.26E+05	1.56E+05	2.57E+05	1.47E+05	2.02E+05	9.71E-01	9.98E-01	9.52E-01	-0.06	0.02	0.08
A0A1D3K2I2	8 (3)	42.6	4.15E-02	1.11E+05	8.66E+04	1.02E+05	8.88E+04	9.48E+04	6.35E+04	1.47E+05	1.06E+05	1.34E+05	4.67E-01	1.75E-01	3.63E-02	-0.28	0.37	0.65
A0A1D3K2I7	13 (6)	128.9	3.61E-02	2.12E+05	2.58E+05	2.53E+05	4.52E+05	4.35E+05	7.43E+05	2.66E+05	3.69E+05	3.90E+05	3.26E-02	5.28E-01	1.34E-01	1.17	0.50	-0.67
A0A1D3K2J8	5 (0)	30.8	NA	NA	NA	NA	NA	NA	NA	NA	NA	NA	NA	NA	NA	NA	NA	NA
A0A1D3K2J9	7 (3)	44.0	1.13E-03	4.05E+04	3.95E+04	4.30E+04	3.08E+04	2.93E+04	3.32E+04	4.58E+04	4.38E+04	4.03E+04	3.70E-03	4.55E-01	1.24E-03	-0.40	0.08	0.48
A0A1D3K2K2	9 (1)	62.5	NA	NA	NA	NA	NA	NA	NA	NA	NA	NA	NA	NA	NA	NA	NA	NA
A0A1D3K2K3	35 (10)	203.0	1.98E-04	8.82E+04	7.98E+04	8.22E+04	9.75E+04	1.00E+05	9.12E+04	1.49E+05	1.28E+05	1.37E+05	1.44E-01	2.00E-04	8.91E-04	0.21	0.73	0.52
A0A1D3K2K5	8 (4)	38.4	1.05E-01	7.49E+04	6.13E+04	6.88E+04	8.31E+04	8.90E+04	6.75E+04	9.50E+04	8.13E+04	8.32E+04	3.09E-01	9.41E-02	6.35E-01	0.22	0.34	0.12
A0A1D3K2K7	11 (3)	54.4	1.55E-01	5.40E+04	5.30E+04	4.47E+04	6.06E+04	6.99E+04	4.29E+04	6.39E+04	9.28E+04	6.44E+04	7.75E-01	1.45E-01	3.45E-01	0.19	0.54	0.35
A0A1D3K2L3	2 (0)	10.1	NA	NA	NA	NA	NA	NA	NA	NA	NA	NA	NA	NA	NA	NA	NA	NA
A0A1D3K2L4	21 (6)	137.6	2.51E-01	4.43E+05	4.26E+05	5.27E+05	5.07E+05	5.68E+05	4.22E+05	6.82E+05	5.81E+05	4.81E+05	8.63E-01	2.42E-01	4.48E-01	0.10	0.32	0.22
A0A1D3K2N2	6 (1)	30.7	1.69E-02	5.38E+03	6.20E+03	6.27E+03	5.58E+03	4.84E+03	4.85E+03	4.88E+03	3.81E+03	3.65E+03	2.06E-01	1.40E-02	1.47E-01	-0.22	-0.53	-0.31
A0A1D3K2P8	5 (0)	35.0	NA	NA	NA	NA	NA	NA	NA	NA	NA	NA	NA	NA	NA	NA	NA	NA
A0A1D3K2Q8	12 (2)	63.6	NA	NA	NA	NA	NA	NA	NA	NA	NA	NA	NA	NA	NA	NA	NA	NA
A0A1D3K2S6	8 (0)	57.3	NA	NA	NA	NA	NA	NA	NA	NA	NA	NA	NA	NA	NA	NA	NA	NA
A0A1D3K2S7	12 (3)	69.0	3.48E-01	3.90E+02	6.02E+03	9.50E+02	3.76E+03	3.84E+03	2.96E+04	0.00E+00	2.20E+03	4.84E+03	4.13E-01	1.00E+00	4.06E-01	2.34	-0.06	-2.40
A0A1D3K2S8	4 (2)	27.9	2.81E-01	7.12E+03	1.16E+04	9.73E+03	1.37E+04	1.13E+04	2.43E+04	7.17E+03	1.24E+04	1.58E+04	2.65E-01	8.39E-01	5.10E-01	0.79	0.31	-0.48
A0A1D3K2T8	5 (2)	20.1	7.43E-04	8.06E+04	5.94E+04	7.02E+04	1.37E+05	1.28E+05	1.25E+05	7.65E+04	8.62E+04	9.96E+04	6.90E-04	1.53E-01	4.17E-03	0.89	0.32	-0.57
A0A1D3K2T9	3 (1)	15.4	2.03E-01	7.95E+03	1.93E+03	1.50E+03	5.02E+02	7.48E+02	4.44E+02	2.00E+03	0.00E+00	1.04E+02	2.44E-01	2.68E-01	9.97E-01	-2.75	-2.44	0.31
A0A1D3K2U2	26 (5)	201.3	3.73E-03	2.35E+05	2.16E+05	2.33E+05	2.29E+05	2.78E+05	1.82E+05	3.98E+05	3.41E+05	3.51E+05	9.98E-01	5.98E-03	6.37E-03	0.01	0.67	0.66
A0A1D3K2U3	12 (5)	60.2	1.11E-01	4.44E+05	3.66E+05	4.11E+05	3.08E+05	3.29E+05	2.22E+05	4.18E+05	3.19E+05	2.75E+05	9.69E-02	3.72E-01	5.59E-01	-0.51	-0.27	0.24
A0A1D3K2U4	7 (1)	41.4	1.12E-02	2.18E+04	2.04E+04	2.57E+04	1.36E+04	1.27E+04	1.69E+04	2.15E+04	2.70E+04	2.20E+04	2.27E-02	9.16E-01	1.44E-02	-0.65	0.06	0.71
A0A1D3K2U5	18 (1)	94.8	2.63E-01	1.02E+05	8.79E+04	1.05E+05	1.07E+05	1.10E+05	9.14E+04	1.22E+05	1.07E+05	1.06E+05	8.46E-01	2.50E-01	4.78E-01	0.06	0.19	0.13
A0A1D3K2U9	15 (5)	81.0	9.55E-01	3.92E+05	3.01E+05	2.40E+05	0.00E+00	1.02E+06	0.00E+00	3.28E+05	3.31E+05	5.30E+05	9.95E-01	9.52E-01	9.78E-01	0.12	0.35	0.22
A0A1D3K2V0	14 (6)	121.8	9.71E-01	2.12E+06	2.22E+06	3.28E+06	3.05E+06	3.25E+06	2.94E+06	0.00E+00	0.00E+00	9.21E+06	9.75E-01	9.76E-01	1.00E+00	0.28	0.27	0.00
A0A1D3K2V5	24 (4)	151.5	7.54E-02	1.21E+05	1.42E+05	1.48E+05	1.74E+05	1.74E+05	2.23E+05	1.45E+05	1.90E+05	1.89E+05	7.14E-02	1.98E-01	7.15E-01	0.47	0.35	-0.12
A0A1D3K2V8	2 (0)	9.2	NA	NA	NA	NA	NA	NA	NA	NA	NA	NA	NA	NA	NA	NA	NA	NA
A0A1D3K2W1	6 (0)	31.3	NA	NA	NA	NA	NA	NA	NA	NA	NA	NA	NA	NA	NA	NA	NA	NA
A0A1D3K2W4	13 (4)	74.1	1.08E-01	4.01E+05	3.18E+05	3.86E+05	2.90E+05	3.15E+05	2.57E+05	3.55E+05	2.72E+05	2.65E+05	1.23E-01	1.78E-01	9.55E-01	-0.36	-0.31	0.05
A0A1D3K2W7	23 (6)	136.3	1.70E-01	2.78E+05	2.30E+05	2.32E+05	1.79E+05	2.07E+05	1.54E+05	2.64E+05	1.84E+05	1.62E+05	1.56E-01	3.97E-01	7.38E-01	-0.46	-0.28	0.18

A0A1D3K2X0	4 (1)	21.6	8.56E-05	1.94E+04	1.76E+04	2.09E+04	7.48E+03	8.94E+03	6.06E+03	1.04E+04	9.68E+03	1.11E+04	8.51E-05	4.19E-04	7.99E-02	-1.37	-0.89	0.47
A0A1D3K2X4	3 (0)	19.0	NA	NA	NA	NA	NA	NA	NA	NA	NA	NA	NA	NA	NA	NA	NA	NA
A0A1D3K2Y0	3 (1)	15.6	2.82E-02	1.14E+05	1.14E+05	9.25E+04	1.23E+05	1.47E+05	1.46E+05	1.57E+05	3.22E+05	3.18E+05	7.71E-01	2.98E-02	7.01E-02	0.38	1.31	0.94
A0A1D3K2Y2	12 (2)	78.8	4.46E-03	3.06E+04	2.01E+04	2.45E+04	6.42E+04	6.93E+04	6.03E+04	7.46E+04	9.70E+04	1.31E+05	6.32E-02	3.60E-03	8.59E-02	1.37	2.01	0.64
A0A1D3K2Y3	36 (12)	196.0	1.42E-01	2.25E+06	1.03E+06	2.08E+06	2.23E+06	2.03E+06	1.92E+06	1.37E+06	1.36E+06	1.20E+06	6.98E-01	3.63E-01	1.29E-01	0.20	-0.45	-0.65
A0A1D3K2Z9	14 (6)	86.7	5.27E-02	9.42E+04	9.23E+04	9.37E+04	6.77E+04	6.74E+04	6.78E+04	9.38E+04	6.81E+04	5.90E+04	5.29E-02	1.28E-01	7.71E-01	-0.47	-0.34	0.12
A0A1D3K314	2 (1)	11.8	8.01E-05	3.31E+03	4.23E+03	3.63E+03	1.77E+03	1.48E+03	1.91E+03	6.97E+02	9.17E+02	9.95E+02	5.37E-04	7.32E-05	3.57E-02	-1.11	-2.10	-0.98
A0A1D3K322	3 (0)	19.9	NA	NA	NA	NA	NA	NA	NA	NA	NA	NA	NA	NA	NA	NA	NA	NA
A0A1D3K323	4 (0)	20.5	NA	NA	NA	NA	NA	NA	NA	NA	NA	NA	NA	NA	NA	NA	NA	NA
A0A1D3K332	32 (10)	250.1	3.87E-04	6.31E+05	7.42E+05	7.46E+05	5.51E+05	5.30E+05	5.94E+05	3.39E+05	3.84E+05	2.74E+05	3.22E-02	3.18E-04	4.65E-03	-0.34	-1.09	-0.75
A0A1D3K334	9 (4)	83.4	1.79E-02	4.29E+05	5.38E+05	4.53E+05	3.62E+05	3.77E+05	3.11E+05	3.84E+05	3.35E+05	3.41E+05	2.61E-02	2.90E-02	9.96E-01	-0.43	-0.42	0.01
A0A1D3K335	74 (21)	480.5	5.27E-01	1.77E+06	4.93E+06	1.56E+06	2.00E+06	3.11E+06	1.67E+06	1.38E+06	2.02E+06	1.37E+06	8.73E-01	5.00E-01	7.79E-01	-0.28	-0.79	-0.51
A0A1D3K342	30 (11)	176.0	1.61E-03	1.08E+06	1.16E+06	1.10E+06	1.38E+06	1.42E+06	2.30E+06	2.65E+06	2.92E+06	3.14E+06	1.54E-01	1.42E-03	1.06E-02	0.62	1.16	0.54
A0A1D3K345	12 (3)	53.4	2.18E-03	1.67E+05	1.20E+05	1.36E+05	2.01E+05	2.81E+05	1.61E+05	3.28E+05	3.36E+05	3.58E+05	1.29E-01	1.85E-03	1.75E-02	0.61	1.27	0.67
A0A1D3K353	6 (2)	31.0	8.99E-04	1.40E+04	1.38E+04	1.27E+04	7.72E+03	1.01E+04	8.58E+03	2.06E+04	2.48E+04	2.96E+04	1.67E-01	4.99E-03	8.32E-04	-0.62	0.89	1.51
A0A1D3K358	3 (0)	13.8	NA	NA	NA	NA	NA	NA	NA	NA	NA	NA	NA	NA	NA	NA	NA	NA
A0A1D3K362	20 (5)	124.5	6.03E-01	6.87E+05	1.06E+06	4.47E+05	9.50E+05	9.26E+05	7.88E+05	7.84E+05	8.23E+05	9.41E+05	5.99E-01	7.41E-01	9.66E-01	0.28	0.22	-0.06
A0A1D3K366	13 (4)	56.7	NA	NA	NA	NA	NA	NA	NA	NA	NA	NA	NA	NA	NA	NA	NA	NA
A0A1D3K367	10 (1)	59.0	5.19E-01	1.27E+02	6.26E+01	3.94E+03	1.03E+02	1.35E+02	3.05E+01	6.15E+02	5.61E+01	1.06E+03	4.96E-01	7.48E-01	8.94E-01	-3.95	-1.25	2.69
A0A1D3K371	2 (1)	12.2	5.53E-06	2.34E+04	2.09E+04	2.28E+04	9.78E+03	8.75E+03	9.56E+03	9.76E+03	9.87E+03	8.07E+03	9.89E-06	9.32E-06	9.88E-01	-1.26	-1.28	-0.02
A0A1D3K372	5 (0)	29.9	NA	NA	NA	NA	NA	NA	NA	NA	NA	NA	NA	NA	NA	NA	NA	NA
A0A1D3K378	6 (2)	31.2	9.87E-01	7.66E+04	5.59E+04	5.78E+04	5.16E+04	9.02E+04	5.99E+04	7.43E+04	1.55E+04	1.05E+05	9.86E-01	9.98E-01	9.95E-01	0.08	0.03	-0.05
A0A1D3K382	11 (4)	62.2	2.03E-01	1.81E+05	1.37E+05	1.59E+05	1.67E+05	1.74E+05	1.67E+05	1.51E+05	1.18E+05	1.53E+05	7.46E-01	4.55E-01	1.86E-01	0.09	-0.17	-0.26
A0A1D3K388	12 (2)	73.8	3.52E-03	1.23E+05	1.00E+05	1.28E+05	1.48E+05	1.48E+05	1.19E+05	1.73E+05	1.80E+05	1.84E+05	2.07E-01	3.08E-03	2.24E-02	0.24	0.61	0.37
A0A1D3K395	5 (0)	28.5	NA	NA	NA	NA	NA	NA	NA	NA	NA	NA	NA	NA	NA	NA	NA	NA
A0A1D3K397	9 (0)	77.5	NA	NA	NA	NA	NA	NA	NA	NA	NA	NA	NA	NA	NA	NA	NA	NA
A0A1D3K398	2 (1)	10.5	1.40E-01	5.56E+04	4.49E+04	5.87E+04	4.61E+04	4.51E+04	4.66E+04	4.93E+04	4.23E+04	3.97E+04	2.71E-01	1.40E-01	8.62E-01	-0.21	-0.28	-0.07
A0A1D3K399	6 (0)	44.6	NA	NA	NA	NA	NA	NA	NA	NA	NA	NA	NA	NA	NA	NA	NA	NA
A0A1D3K3A4	48 (20)	378.2	9.22E-05	6.25E+05	5.12E+05	5.67E+05	7.14E+05	7.58E+05	6.56E+05	1.19E+06	1.22E+06	1.40E+06	1.60E-01	9.98E-05	3.57E-04	0.32	1.16	0.84
A0A1D3K3A7	6 (1)	47.5	NA	NA	NA	NA	NA	NA	NA	NA	NA	NA	NA	NA	NA	NA	NA	NA
A0A1D3K3B1	23 (6)	158.5	4.34E-01	3.28E+05	1.83E+05	1.85E+05	2.16E+05	1.81E+05	1.70E+05	1.71E+05	1.34E+05	2.10E+05	6.24E-01	4.24E-01	9.24E-01	-0.30	-0.43	-0.14
A0A1D3K3B3	2 (0)	4.8	NA	NA	NA	NA	NA	NA	NA	NA	NA	NA	NA	NA	NA	NA	NA	NA
A0A1D3K3C7	12 (4)	82.2	2.56E-01	2.77E+05	2.31E+05	2.60E+05	3.05E+05	3.28E+05	2.59E+05	3.77E+05	3.07E+05	2.65E+05	4.75E-01	2.43E-01	8.37E-01	0.22	0.31	0.09
A0A1D3K3D5	6 (1)	38.3	1.65E-02	4.62E+03	4.25E+03	4.78E+03	2.85E+03	2.60E+03	2.00E+03	4.20E+03	3.08E+03	2.36E+03	1.45E-02	8.22E-02	3.72E-01	-0.87	-0.50	0.37
A0A1D3K3F7	9 (1)	56.3	NA	NA	NA	NA	NA	NA	NA	NA	NA	NA	NA	NA	NA	NA	NA	NA
A0A1D3K3F8	3 (0)	15.9	NA	NA	NA	NA	NA	NA	NA	NA	NA	NA	NA	NA	NA	NA	NA	NA
A0A1D3K3G0	3 (0)	14.1	NA	NA	NA	NA	NA	NA	NA	NA	NA	NA	NA	NA	NA	NA	NA	NA
A0A1D3K3G5	3 (0)	15.9	NA	NA	NA	NA	NA	NA	NA	NA	NA	NA	NA	NA	NA	NA	NA	NA
A0A1D3K3G8	2 (0)	11.6	NA	NA	NA	NA	NA	NA	NA	NA	NA	NA	NA	NA	NA	NA	NA	NA
A0A1D3K3H2	14 (5)	94.7	3.87E-01	1.77E+05	1.57E+05	1.80E+05	1.65E+05	1.69E+05	1.68E+05	2.42E+05	1.90E+05	1.57E+05	9.83E-01	4.97E-01	4.10E-01	-0.03	0.20	0.23
A0A1D3K3I7	2 (1)	11.2	3.01E-03	3.81E+03	2.41E+03	2.84E+03	9.12E+02	8.00E+02	5.89E+02	1.20E+03	1.36E+03	1.90E+03	2.70E-03	1.71E-02	2.32E-01	-1.98	-1.03	0.95
A0A1D3K3J3	9 (2)	48.6	NA	NA	NA	NA	NA	NA	NA	NA	NA	NA	NA	NA	NA	NA	NA	NA
A0A1D3K3K1	18 (9)	117.3	5.51E-01	1.40E+05	1.50E+05	1.48E+05	1.46E+05	1.38E+05	2.07E+05	1.47E+05	1.50E+05	1.38E+05	6.23E-01	9.98E-01	5.92E-01	0.16	-0.01	-0.17
A0A1D3K3K8	5 (3)	46.0	2.11E-03	4.12E+04	4.93E+04	4.96E+04	2.91E+04	2.65E+04	3.45E+04	2.12E+04	2.85E+04	2.70E+04	7.33E-03	2.22E-03	4.54E-01	-0.64	-0.87	-0.23
A0A1D3K3K9	7 (1)	84.3	NA	NA	NA	NA	NA	NA	NA	NA	NA	NA	NA	NA	NA	NA	NA	NA
A0A1D3K3L5	3 (0)	15.8	NA	NA	NA	NA	NA	NA	NA	NA	NA	NA	NA	NA	NA	NA	NA	NA
A0A1D3K3L8	3 (1)	68.4	2.02E-02	7.51E+04	1.46E+05	1.05E+05	1.68E+05	1.81E+05	1.67E+05	1.79E+05	1.57E+05	1.71E+05	2.81E-02	3.43E-02	9.85E-01	0.68	0.66	-0.03
A0A1D3K3M6	4 (1)	22.6	8.45E-04	8.77E+03	6.82E+03	9.84E+03	3.35E+03	2.45E+03	1.61E+03	3.04E+03	1.28E+03	1.17E+03	1.95E-03	1.14E-03	7.94E-01	-1.78	-2.21	-0.43
A0A1D3K3M8	4 (0)	15.3	NA	NA	NA	NA	NA	NA	NA	NA	NA	NA	NA	NA	NA	NA	NA	NA
A0A1D3K3P0	7 (3)	41.2	3.81E-03	6.93E+04	7.21E+04	9.07E+04	4.02E+04	4.38E+04	3.25E+04	4.96E+04	5.64E+04	4.22E+04	3.62E-03	1.68E-02	3.51E-01	-0.99	-0.65	0.35
A0A1D3K3P2	13 (3)	56.7	NA	NA	NA	NA	NA	NA	NA	NA	NA	NA	NA	NA	NA	NA	NA	NA
A0A1D3K3P7	7 (2)	40.9	3.85E-02	8.00E+04	6.85E+04	7.58E+04	1.06E+05	1.07E+05	1.03E+05	9.55E+04	5.43E+04	5.25E+04	8.85E-02	8.15E-01	4.09E-02	0.50	-0.15	-0.65

A0A1D3K3P9	13 (2)	64.6	5.85E-03	1.57E+04	1.49E+04	1.78E+04	3.11E+04	2.94E+04	4.82E+04	5.40E+04	3.96E+04	5.58E+04	4.82E-02	4.87E-03	1.71E-01	0.75	0.99	0.24
A0A1D3K3Q9	36 (11)	250.2	7.78E-01	1.01E+06	9.64E+05	9.55E+05	8.78E+05	9.29E+05	6.95E+05	1.58E+06	7.87E+05	6.30E+05	8.39E-01	9.95E-01	7.88E-01	-0.23	0.04	0.26
A0A1D3K3R3	5 (0)	25.7	NA	NA	NA	NA	NA	NA	NA	NA	NA	NA	NA	NA	NA	NA	NA	NA
A0A1D3K3R6	20 (4)	102.5	1.11E-04	2.17E+05	2.45E+05	2.31E+05	3.21E+05	3.36E+05	2.88E+05	4.52E+05	4.00E+05	4.32E+05	8.52E-03	8.92E-05	1.92E-03	0.45	0.89	0.44
A0A1D3K3S6	14 (5)	77.6	7.91E-03	2.16E+05	1.70E+05	1.99E+05	3.37E+05	3.64E+05	2.91E+05	2.89E+05	2.11E+05	2.39E+05	6.74E-03	2.37E-01	5.27E-02	0.76	0.34	-0.43
A0A1D3K3T4	5 (2)	40.5	9.12E-01	3.41E+04	5.54E+04	3.80E+04	3.57E+04	3.23E+04	5.10E+04	3.64E+04	4.62E+04	4.45E+04	9.24E-01	1.00E+00	9.30E-01	-0.10	0.00	0.10
A0A1D3K3T6	5 (1)	56.7	NA	NA	NA	NA	NA	NA	NA	NA	NA	NA	NA	NA	NA	NA	NA	NA
A0A1D3K3U5	3 (1)	48.6	NA	NA	NA	NA	NA	NA	NA	NA	NA	NA	NA	NA	NA	NA	NA	NA
A0A1D3K3U7	64 (17)	454.4	9.00E-02	2.27E+06	1.60E+06	1.02E+06	1.11E+06	1.00E+06	1.16E+06	7.85E+05	9.18E+05	7.85E+05	2.49E-01	8.28E-02	6.78E-01	-0.58	-0.98	-0.39
A0A1D3K3U9	6 (1)	41.3	9.53E-01	1.61E+05	1.94E+05	2.75E+05	1.90E+05	2.06E+05	2.10E+05	2.18E+05	2.25E+05	1.87E+05	9.61E-01	1.00E+00	9.61E-01	-0.06	0.00	0.06
A0A1D3K3V1	7 (3)	48.0	9.77E-02	1.39E+05	1.70E+05	1.65E+05	1.28E+05	1.26E+05	1.45E+05	1.31E+05	1.48E+05	1.32E+05	1.07E-01	1.74E-01	9.24E-01	-0.25	-0.21	0.04
A0A1D3K3V4	13 (2)	77.9	1.11E-02	1.83E+05	1.40E+05	1.63E+05	8.44E+04	8.90E+04	5.18E+04	1.21E+05	7.47E+04	5.67E+04	1.41E-02	2.29E-02	9.06E-01	-1.11	-0.95	0.16
A0A1D3K3W3	24 (11)	225.8	4.83E-01	1.61E+06	1.36E+06	1.56E+06	1.49E+06	1.54E+06	1.29E+06	1.43E+06	1.52E+06	9.61E+05	9.05E-01	4.64E-01	6.99E-01	-0.07	-0.21	-0.14
A0A1D3K3W6	9 (2)	50.0	1.09E-01	1.45E+04	1.08E+04	1.37E+04	7.51E+03	9.50E+03	5.58E+03	1.35E+04	6.75E+03	7.24E+03	1.03E-01	2.62E-01	7.51E-01	-0.79	-0.51	0.28
A0A1D3K3X1	8 (5)	43.5	3.89E-02	1.83E+04	2.16E+04	1.89E+04	3.47E+04	3.17E+04	5.49E+04	5.34E+04	6.64E+04	1.22E+05	5.20E-01	3.48E-02	1.47E-01	1.04	2.04	0.99
A0A1D3K3Z2	6 (1)	59.4	NA	NA	NA	NA	NA	NA	NA	NA	NA	NA	NA	NA	NA	NA	NA	NA
A0A1D3K3Z6	20 (5)	142.2	5.83E-02	1.83E+05	1.81E+05	2.06E+05	2.81E+05	2.86E+05	2.79E+05	2.45E+05	3.09E+05	1.85E+05	5.09E-02	2.28E-01	5.02E-01	0.57	0.37	-0.20
A0A1D3K402	21 (7)	122.7	4.30E-04	1.39E+05	1.34E+05	1.56E+05	2.00E+05	2.06E+05	1.76E+05	3.98E+05	3.24E+05	4.45E+05	2.83E-01	4.58E-04	1.62E-03	0.44	1.44	1.00
A0A1D3K403	9 (1)	46.7	6.02E-01	5.55E+02	3.19E+03	9.07E+02	1.83E+03	6.71E+02	3.94E+03	0.00E+00	2.13E+03	6.73E+02	8.66E-01	8.57E-01	5.75E-01	0.47	-0.73	-1.20
A0A1D3K406	9 (0)	43.4	NA	NA	NA	NA	NA	NA	NA	NA	NA	NA	NA	NA	NA	NA	NA	NA
A0A1D3K408	16 (1)	85.8	5.52E-01	1.50E+03	5.46E+01	2.90E+03	1.74E+03	4.66E+03	4.68E+03	1.02E+03	0.00E+00	6.42E+03	5.25E-01	8.66E-01	8.11E-01	1.32	0.74	-0.57
A0A1D3K427	3 (2)	15.8	1.47E-01	8.61E+04	8.66E+04	9.37E+04	1.13E+05	1.12E+05	1.09E+05	7.74E+04	1.02E+05	1.17E+05	1.29E-01	5.77E-01	4.62E-01	0.33	0.16	-0.17
A0A1D3K431	7 (3)	41.3	1.33E-01	1.10E+05	1.36E+05	1.24E+05	1.77E+05	1.61E+05	2.34E+05	7.88E+04	1.75E+05	1.48E+05	1.44E-01	9.34E-01	2.25E-01	0.63	0.12	-0.51
A0A1D3K434	16 (4)	75.3	3.99E-01	3.27E+05	2.59E+05	3.08E+05	2.90E+05	3.18E+05	2.19E+05	3.74E+05	3.34E+05	2.80E+05	8.24E-01	6.85E-01	3.73E-01	-0.11	0.14	0.26
A0A1D3K439	2 (0)	10.5	NA	NA	NA	NA	NA	NA	NA	NA	NA	NA	NA	NA	NA	NA	NA	NA
A0A1D3K442	8 (1)	38.0	1.11E-01	9.94E+04	8.16E+04	9.50E+04	8.45E+04	8.20E+04	7.95E+04	8.40E+04	8.12E+04	7.85E+04	1.67E-01	1.34E-01	9.84E-01	-0.17	-0.18	-0.01
A0A1D3K443	28 (10)	184.6	2.41E-01	1.44E+05	1.34E+05	1.36E+05	1.30E+05	1.47E+05	1.87E+05	1.29E+05	1.23E+05	1.31E+05	5.02E-01	7.72E-01	2.22E-01	0.17	-0.11	-0.28
A0A1D3K444	2 (0)	11.3	NA	NA	NA	NA	NA	NA	NA	NA	NA	NA	NA	NA	NA	NA	NA	NA
A0A1D3K451	30 (9)	169.0	3.09E-02	2.05E+05	1.55E+05	1.79E+05	2.01E+05	2.21E+05	1.47E+05	4.33E+05	2.42E+05	3.48E+05	9.80E-01	4.12E-02	5.20E-02	0.07	0.92	0.85
A0A1D3K459	8 (2)	59.5	NA	NA	NA	NA	NA	NA	NA	NA	NA	NA	NA	NA	NA	NA	NA	NA
A0A1D3K465	8 (4)	65.4	3.69E-01	2.37E+05	1.83E+05	2.07E+05	1.75E+05	1.95E+05	1.24E+05	2.26E+05	1.46E+05	1.36E+05	3.98E-01	4.70E-01	9.87E-01	-0.34	-0.30	0.04
A0A1D3K476|	3 (1)	58.4	9.66E-03	1.87E+04	2.03E+04	1.86E+04	2.95E+04	2.59E+04	3.43E+04	2.93E+04	2.79E+04	2.44E+04	9.48E-03	3.40E-02	5.26E-01	0.64	0.50	-0.14
A0A1D3K477	6 (0)	39.6	NA	NA	NA	NA	NA	NA	NA	NA	NA	NA	NA	NA	NA	NA	NA	NA
A0A1D3K478	15 (4)	112.7	3.01E-02	1.62E+05	1.12E+05	1.46E+05	1.65E+05	1.74E+05	1.46E+05	2.53E+05	1.87E+05	2.05E+05	5.88E-01	2.82E-02	1.00E-01	0.21	0.62	0.41
A0A1D3K486	6 (2)	53.9	NA	NA	NA	NA	NA	NA	NA	NA	NA	NA	NA	NA	NA	NA	NA	NA
A0A1D3K488	11 (3)	50.1	1.05E-01	2.30E+04	2.24E+04	2.14E+04	2.56E+04	2.26E+04	2.71E+04	2.36E+04	3.72E+04	4.57E+04	8.63E-01	1.07E-01	2.09E-01	0.17	0.67	0.50
A0A1D3K496	20 (4)	61.4	NA	NA	NA	NA	NA	NA	NA	NA	NA	NA	NA	NA	NA	NA	NA	NA
A0A1D3K4A0	3 (1)	16.2	6.66E-02	4.14E+03	4.05E+03	3.80E+03	3.04E+03	3.84E+03	2.82E+03	2.69E+03	3.54E+03	2.37E+03	2.02E-01	6.10E-02	6.36E-01	-0.31	-0.48	-0.17
A0A1D3K4A8	4 (1)	23.4	1.18E-03	3.97E+04	2.77E+04	3.36E+04	4.11E+04	4.61E+04	3.54E+04	1.30E+05	8.59E+04	1.02E+05	8.05E-01	1.58E-03	2.72E-03	0.28	1.66	1.38
A0A1D3K4B7	2 (1)	48.4	NA	NA	NA	NA	NA	NA	NA	NA	NA	NA	NA	NA	NA	NA	NA	NA
A0A1D3K4C4	2 (0)	9.3	NA	NA	NA	NA	NA	NA	NA	NA	NA	NA	NA	NA	NA	NA	NA	NA
A0A1D3K4D2	4 (0)	25.0	NA	NA	NA	NA	NA	NA	NA	NA	NA	NA	NA	NA	NA	NA	NA	NA
A0A1D3K4D7	3 (0)	9.2	NA	NA	NA	NA	NA	NA	NA	NA	NA	NA	NA	NA	NA	NA	NA	NA
A0A1D3K4G0	3 (1)	11.0	5.29E-01	3.35E+04	2.14E+04	2.82E+04	1.98E+04	3.75E+04	2.55E+04	3.35E+04	3.14E+03	1.90E+04	1.00E+00	5.82E-01	5.89E-01	-0.01	-0.58	-0.57
A0A1D3K4G6	20 (7)	149.4	1.42E-03	8.94E+05	7.08E+05	8.33E+05	1.18E+06	1.27E+06	1.28E+06	1.12E+06	9.71E+05	1.06E+06	1.14E-03	2.12E-02	4.94E-02	0.62	0.37	-0.24
A0A1D3K4H2	3 (0)	15.2	NA	NA	NA	NA	NA	NA	NA	NA	NA	NA	NA	NA	NA	NA	NA	NA
A0A1D3K4H5	6 (3)	38.3	3.07E-04	1.74E+05	1.95E+05	1.77E+05	3.39E+05	3.20E+05	2.50E+05	4.83E+05	4.10E+05	4.33E+05	1.29E-02	2.43E-04	6.72E-03	0.74	1.28	0.54
A0A1D3K4H8	3 (0)	17.9	NA	NA	NA	NA	NA	NA	NA	NA	NA	NA	NA	NA	NA	NA	NA	NA
A0A1D3K4I6	14 (4)	123.2	7.34E-01	3.38E+05	2.66E+05	3.17E+05	3.14E+05	3.16E+05	2.54E+05	3.63E+05	2.26E+05	2.25E+05	9.59E-01	7.19E-01	8.65E-01	-0.06	-0.18	-0.12
A0A1D3K4I7	8 (1)	51.6	2.77E-01	1.76E+04	1.55E+04	1.94E+04	2.77E+04	3.05E+04	8.30E+03	1.19E+04	1.04E+04	1.31E+04	7.15E-01	6.13E-01	2.52E-01	0.34	-0.57	-0.91
A0A1D3K4J2	28 (8)	155.2	1.43E-01	1.52E+05	1.10E+05	1.31E+05	1.65E+05	1.81E+05	1.29E+05	1.72E+05	1.68E+05	1.61E+05	2.80E-01	1.42E-01	8.54E-01	0.27	0.35	0.08
A0A1D3K4J6	6 (1)	41.2	6.45E-02	9.74E+03	1.17E+04	1.13E+04	9.03E+03	7.90E+03	1.02E+04	7.45E+03	9.57E+03	6.75E+03	2.35E-01	5.69E-02	5.37E-01	-0.27	-0.46	-0.19

A0A1D3K4K1	8 (2)	69.2	7.84E-03	2.98E+05	3.35E+05	2.97E+05	4.00E+05	3.89E+05	4.26E+05	3.45E+05	3.61E+05	3.02E+05	7.47E-03	4.44E-01	3.13E-02	0.39	0.12	-0.27
A0A1D3K4K4	9 (1)	69.9	5.24E-01	1.08E+05	8.71E+04	1.03E+05	9.46E+04	9.68E+04	7.29E+04	1.12E+05	6.43E+04	6.87E+04	7.40E-01	5.03E-01	9.06E-01	-0.17	-0.28	-0.11
A0A1D3K4K6	6 (0)	35.2	NA	NA	NA	NA	NA	NA	NA	NA	NA	NA	NA	NA	NA	NA	NA	NA
A0A1D3K4K8	4 (2)	28.6	2.07E-03	6.00E+04	4.49E+04	4.80E+04	8.44E+04	9.68E+04	8.00E+04	7.40E+04	7.21E+04	7.77E+04	1.79E-03	1.45E-02	1.54E-01	0.77	0.55	-0.22
A0A1D3K4L0	3 (0)	15.9	NA	NA	NA	NA	NA	NA	NA	NA	NA	NA	NA	NA	NA	NA	NA	NA
A0A1D3K4L2	8 (4)	63.5	3.41E-02	2.21E+05	3.20E+05	2.41E+05	3.45E+05	3.01E+05	3.23E+05	2.03E+05	2.34E+05	2.38E+05	1.48E-01	4.53E-01	2.99E-02	0.31	-0.21	-0.52
A0A1D3K4L5	20 (6)	152.6	2.46E-02	5.65E+05	5.29E+05	5.80E+05	3.96E+05	4.29E+05	3.61E+05	4.97E+05	3.65E+05	3.09E+05	3.94E-02	3.52E-02	9.95E-01	-0.50	-0.51	-0.02
A0A1D3K4M1	60 (15)	359.2	1.50E-01	4.36E+05	5.93E+05	4.44E+05	3.39E+05	3.39E+05	4.90E+05	3.36E+05	4.08E+05	2.97E+05	3.24E-01	1.43E-01	7.95E-01	-0.33	-0.50	-0.17
A0A1D3K4M2	7 (1)	59.7	3.44E-02	1.24E+04	7.56E+03	6.46E+03	9.81E+03	1.19E+04	7.64E+03	1.46E+04	1.65E+04	1.39E+04	8.67E-01	3.86E-02	7.29E-02	0.15	0.77	0.62
A0A1D3K4M3	5 (0)	27.1	NA	NA	NA	NA	NA	NA	NA	NA	NA	NA	NA	NA	NA	NA	NA	NA
A0A1D3K4M5	11 (2)	76.3	4.84E-03	3.28E+03	4.76E+03	5.08E+03	9.89E+03	9.59E+03	1.35E+04	7.58E+03	9.60E+03	8.15E+03	4.08E-03	3.76E-02	1.75E-01	1.33	0.95	-0.38
A0A1D3K4M9	13 (5)	82.8	2.50E-02	3.58E+05	2.65E+05	3.06E+05	4.56E+05	5.08E+05	3.47E+05	3.08E+05	1.97E+05	1.84E+05	1.29E-01	3.71E-01	2.15E-02	0.50	-0.43	-0.93
A0A1D3K4N2	8 (4)	61.1	3.25E-02	3.73E+05	2.93E+05	3.57E+05	2.49E+05	2.70E+05	2.06E+05	2.87E+05	2.11E+05	2.05E+05	5.55E-02	4.23E-02	9.74E-01	-0.50	-0.54	-0.04
A0A1D3K4N4	8 (3)	74.9	7.73E-01	3.86E+05	3.79E+05	4.02E+05	4.10E+05	3.98E+05	4.11E+05	4.18E+05	4.23E+05	3.25E+05	8.07E-01	1.00E+00	8.07E-01	0.06	0.00	-0.06
A0A1D3K4N7	24 (7)	211.9	3.82E-01	6.25E+05	6.21E+05	6.80E+05	7.35E+05	7.23E+05	7.64E+05	8.03E+05	8.32E+05	5.61E+05	4.16E-01	4.76E-01	9.92E-01	0.21	0.19	-0.02
A0A1D3K4N8	3 (2)	32.1	5.45E-05	9.40E+04	9.96E+04	8.95E+04	1.42E+05	1.53E+05	1.78E+05	4.39E+04	3.79E+04	3.05E+04	1.55E-03	2.68E-03	4.27E-05	0.74	-1.33	-2.07
A0A1D3K4N9	2 (1)	10.3	6.66E-01	9.90E+04	1.10E+05	1.03E+05	1.15E+05	9.84E+04	1.50E+05	1.00E+05	1.49E+05	9.58E+04	6.47E-01	8.34E-01	9.39E-01	0.22	0.15	-0.08
A0A1D3K4P2	3 (1)	20.9	2.62E-02	5.26E+03	9.18E+03	8.43E+03	3.47E+04	3.76E+04	8.69E+04	8.19E+03	5.93E+03	9.14E+03	3.91E-02	1.00E+00	3.96E-02	2.80	0.02	-2.77
A0A1D3K4P3	17 (8)	130.9	1.73E-02	4.72E+05	4.50E+05	3.23E+05	5.94E+05	6.65E+05	5.47E+05	4.63E+05	4.50E+05	4.13E+05	2.01E-02	8.48E-01	3.85E-02	0.54	0.09	-0.45
A0A1D3K4P6	5 (1)	32.1	5.72E-01	4.08E+01	3.62E+03	3.45E+02	6.15E+02	5.17E+02	1.47E+04	2.39E+01	9.73E+02	3.39E+03	6.18E-01	9.99E-01	6.36E-01	1.98	0.13	-1.85
A0A1D3K4P8	6 (2)	37.1	1.15E-01	3.18E+05	2.59E+05	2.95E+05	3.11E+05	3.11E+05	2.31E+05	2.70E+05	1.88E+05	1.61E+05	9.82E-01	1.37E-01	1.73E-01	-0.03	-0.50	-0.46
A0A1D3K4Q0	7 (3)	26.1	1.06E-02	8.41E+04	7.88E+04	8.94E+04	1.04E+05	9.53E+04	1.12E+05	7.34E+04	7.54E+04	8.67E+04	3.23E-02	6.23E-01	1.09E-02	0.31	-0.10	-0.41
A0A1D3K4Q1	10 (5)	35.5	3.25E-01	4.99E+05	4.23E+05	4.88E+05	4.46E+05	4.71E+05	4.29E+05	4.96E+05	3.56E+05	3.32E+05	8.95E-01	3.15E-01	5.21E-01	-0.07	-0.25	-0.19
A0A1D3K4Q3	13 (4)	91.8	1.04E-01	1.02E+05	7.50E+04	9.09E+04	5.52E+04	6.10E+04	4.36E+04	9.10E+04	5.18E+04	4.11E+04	1.05E-01	2.12E-01	8.50E-01	-0.74	-0.54	0.20
A0A1D3K4Q4	6 (3)	34.7	3.13E-02	1.27E+05	2.06E+05	1.30E+05	5.13E+04	4.40E+04	6.43E+04	4.75E+04	1.11E+05	1.16E+05	2.71E-02	1.47E-01	4.16E-01	-1.53	-0.75	0.78
A0A1D3K4Q5	5 (1)	48.6	NA	NA	NA	NA	NA	NA	NA	NA	NA	NA	NA	NA	NA	NA	NA	NA
A0A1D3K4Q7	15 (7)	98.7	1.98E-02	5.97E+05	5.59E+05	6.17E+05	4.68E+05	4.85E+05	4.69E+05	5.06E+05	4.49E+05	3.48E+05	6.19E-02	1.94E-02	6.19E-01	-0.32	-0.44	-0.13
A0A1D3K4Q9	11 (4)	67.7	8.73E-02	1.15E+05	1.36E+05	1.22E+05	9.51E+04	1.03E+05	1.32E+05	1.06E+05	8.34E+04	8.47E+04	5.07E-01	7.58E-02	3.33E-01	-0.17	-0.44	-0.27
A0A1D3K4R1	9 (4)	62.0	1.73E-01	3.24E+05	2.81E+05	3.35E+05	3.27E+05	3.54E+05	2.64E+05	5.77E+05	2.93E+05	4.87E+05	1.00E+00	2.18E-01	2.26E-01	0.01	0.53	0.52
A0A1D3K4R6	5 (1)	37.5	5.33E-03	2.73E+04	3.12E+04	2.64E+04	3.49E+04	3.08E+04	3.85E+04	1.58E+04	2.38E+04	1.85E+04	1.45E-01	4.94E-02	4.40E-03	0.30	-0.55	-0.84
A0A1D3K4R7	7 (2)	54.9	2.99E-01	1.91E+04	2.29E+04	2.11E+04	2.88E+04	2.70E+04	6.16E+04	2.05E+04	4.30E+04	3.59E+04	2.83E-01	5.31E-01	8.46E-01	0.90	0.66	-0.24
A0A1D3K4R8	11 (3)	65.4	1.72E-02	4.41E+03	7.67E+03	5.94E+03	1.15E+03	1.15E+03	2.83E+03	1.87E+03	2.42E+03	4.26E+03	1.66E-02	5.82E-02	5.70E-01	-1.81	-1.08	0.73
A0A1D3K4S6	12 (5)	55.2	9.98E-03	2.55E+04	2.75E+04	2.59E+04	2.16E+04	1.93E+04	2.33E+04	1.93E+04	2.14E+04	2.20E+04	2.00E-02	1.30E-02	9.22E-01	-0.30	-0.33	-0.03
A0A1D3K4S9	15 (1)	104.4	3.87E-01	1.51E+05	1.11E+05	1.18E+05	1.03E+05	1.10E+05	7.22E+04	1.44E+05	8.89E+04	7.36E+04	3.87E-01	5.46E-01	9.45E-01	-0.42	-0.31	0.11
A0A1D3K4T9	10 (4)	85.7	2.79E-03	1.69E+05	1.48E+05	1.64E+05	2.60E+05	2.64E+05	2.69E+05	1.89E+05	2.34E+05	2.58E+05	2.38E-03	2.05E-02	1.58E-01	0.73	0.50	-0.22
A0A1D3K4U0	5 (0)	27.7	NA	NA	NA	NA	NA	NA	NA	NA	NA	NA	NA	NA	NA	NA	NA	NA
A0A1D3K4U2	7 (2)	44.0	6.95E-04	1.38E+05	1.08E+05	1.23E+05	2.09E+05	2.40E+05	1.92E+05	3.67E+05	2.76E+05	3.53E+05	3.35E-02	5.60E-04	1.04E-02	0.80	1.43	0.64
A0A1D3K4U3	8 (1)	54.6	7.39E-01	1.48E+04	2.82E+04	2.03E+04	2.10E+04	1.75E+04	3.53E+04	6.02E+03	2.98E+04	1.92E+04	8.99E-01	9.35E-01	7.19E-01	0.22	-0.20	-0.42
A0A1D3K4U4	2 (1)	6.1	1.03E-03	3.36E+03	2.97E+03	3.43E+03	7.10E+03	7.05E+03	6.98E+03	1.06E+04	7.44E+03	8.27E+03	6.36E-03	9.30E-04	1.46E-01	1.11	1.43	0.31
A0A1D3K4U9	45 (14)	327.2	1.42E-01	7.00E+05	5.96E+05	6.51E+05	4.85E+05	5.33E+05	4.43E+05	7.03E+05	5.26E+05	4.50E+05	1.25E-01	4.51E-01	5.75E-01	-0.41	-0.21	0.20
A0A1D3K4V1	48 (17)	296.6	4.95E-05	4.44E+05	3.93E+05	4.49E+05	5.00E+05	5.10E+05	4.76E+05	7.69E+05	7.09E+05	8.02E+05	1.18E-01	5.36E-05	1.94E-04	0.21	0.82	0.62
A0A1D3K4V5	5 (1)	58.3	NA	NA	NA	NA	NA	NA	NA	NA	NA	NA	NA	NA	NA	NA	NA	NA
A0A1D3K4W0	4 (1)	22.7	1.19E-01	2.30E+04	3.40E+04	2.98E+04	2.37E+04	2.20E+04	3.35E+04	1.21E+04	2.23E+04	1.93E+04	8.49E-01	1.19E-01	2.39E-01	-0.13	-0.69	-0.56
A0A1D3K4W5	5 (0)	27.0	NA	NA	NA	NA	NA	NA	NA	NA	NA	NA	NA	NA	NA	NA	NA	NA
A0A1D3K4W6	6 (0)	32.6	NA	NA	NA	NA	NA	NA	NA	NA	NA	NA	NA	NA	NA	NA	NA	NA
A0A1D3K4W8	22 (3)	121.4	7.37E-01	2.97E+05	2.79E+05	3.12E+05	2.95E+05	3.01E+05	2.71E+05	2.94E+05	2.95E+05	2.69E+05	8.54E-01	7.26E-01	9.68E-01	-0.03	-0.05	-0.02
A0A1D3K4X6	6 (1)	30.1	1.34E-02	2.36E+03	1.97E+03	2.19E+03	4.21E+03	5.65E+03	2.44E+03	1.10E+04	1.24E+04	5.86E+03	5.66E-01	1.31E-02	4.50E-02	0.92	2.17	1.26
A0A1D3K4Y0	14 (4)	114.8	2.76E-01	1.16E+06	8.71E+05	1.09E+06	1.24E+06	1.35E+06	1.04E+06	1.20E+06	7.70E+05	8.58E+05	5.32E-01	8.04E-01	2.57E-01	0.22	-0.14	-0.36
A0A1D3K4Z6	5 (1)	68.4	NA	NA	NA	NA	NA	NA	NA	NA	NA	NA	NA	NA	NA	NA	NA	NA
A0A1D3K4Z9	4 (1)	30.2	1.30E-01	5.42E+04	4.76E+04	5.93E+04	4.45E+04	4.64E+04	3.48E+04	5.13E+04	3.13E+04	3.19E+04	2.60E-01	1.29E-01	8.48E-01	-0.36	-0.49	-0.13
A0A1D3K500	70 (12)	430.3	6.09E-01	3.36E+05	4.35E+05	7.29E+05	3.85E+05	4.35E+05	4.13E+05	4.54E+05	4.30E+05	3.37E+05	6.72E-01	6.50E-01	9.99E-01	-0.28	-0.30	-0.01
A0A1D3K508	73 (16)	469.6	2.55E-03	3.34E+06	3.25E+06	2.77E+06	1.94E+06	2.03E+06	2.33E+06	2.13E+06	2.32E+06	2.06E+06	3.61E-03	5.15E-03	9.25E-01	-0.57	-0.52	0.05

A0A1D3K511	27 (3)	178.7	6.15E-01	1.40E+05	1.11E+05	1.27E+05	1.19E+05	1.35E+05	9.65E+04	1.46E+05	8.19E+04	8.73E+04	8.97E-01	5.90E-01	8.37E-01	-0.11	-0.26	-0.15
A0A1D3K526	7 (0)	52.6	NA	NA	NA	NA	NA	NA	NA	NA	NA	NA	NA	NA	NA	NA	NA	NA
A0A1D3K530	6 (1)	39.2	2.54E-01	1.21E+04	8.86E+03	1.23E+04	6.48E+03	7.79E+03	4.15E+03	1.25E+04	2.70E+03	9.38E+03	2.32E-01	5.55E-01	7.35E-01	-0.85	-0.44	0.42
A0A1D3K535	3 (2)	14.8	1.83E-02	6.78E+04	6.99E+04	7.93E+04	8.51E+04	8.47E+04	8.97E+04	6.88E+04	7.79E+04	6.72E+04	3.28E-02	9.67E-01	2.45E-02	0.26	-0.02	-0.28
A0A1D3K540	9 (2)	55.2	3.41E-01	5.52E+03	7.73E+03	4.39E+03	3.52E+03	5.17E+03	4.57E+03	5.15E+03	4.97E+03	2.53E+03	4.48E-01	3.67E-01	9.83E-01	-0.41	-0.48	-0.07
A0A1D3K541	3 (0)	15.7	NA	NA	NA	NA	NA	NA	NA	NA	NA	NA	NA	NA	NA	NA	NA	NA
A0A1D3K546	25 (5)	129.5	3.05E-01	1.45E+05	1.48E+05	1.66E+05	1.58E+05	1.64E+05	1.90E+05	1.18E+05	1.75E+05	1.33E+05	5.86E-01	7.92E-01	2.82E-01	0.15	-0.11	-0.26
A0A1D3K548	39 (8)	297.7	1.85E-01	7.67E+05	5.15E+05	6.50E+05	5.37E+05	5.67E+05	4.38E+05	8.42E+05	7.06E+05	5.73E+05	3.97E-01	7.81E-01	1.73E-01	-0.33	0.14	0.46
A0A1D3K549	2 (1)	8.7	3.38E-03	2.68E+02	6.81E+02	4.70E+02	2.63E+03	2.77E+03	2.74E+03	0.00E+00	1.67E+03	3.90E+02	4.48E-03	8.73E-01	7.32E-03	2.52	0.54	-1.98
A0A1D3K558	10 (2)	50.9	7.55E-05	3.50E+05	2.70E+05	3.37E+05	5.24E+05	5.83E+05	4.50E+05	1.12E+06	1.00E+06	1.26E+06	7.26E-02	7.49E-05	3.74E-04	0.70	1.82	1.12
A0A1D3K560	9 (2)	57.0	9.57E-01	8.65E+04	6.47E+04	7.31E+04	7.07E+04	8.37E+04	5.74E+04	1.01E+05	3.83E+04	7.11E+04	9.67E-01	9.61E-01	1.00E+00	-0.08	-0.09	-0.01
A0A1D3K563	2 (1)	14.9	7.17E-06	1.89E+03	2.09E+03	2.19E+03	2.27E+02	2.65E+02	2.86E+02	1.93E+02	4.06E+02	0.00E+00	1.40E-05	1.14E-05	8.77E-01	-2.99	-3.36	-0.38
A0A1D3K565	4 (1)	25.6	4.24E-02	8.22E+02	5.18E+02	4.86E+02	3.19E+03	4.71E+03	3.61E+03	2.43E+04	3.75E+04	9.21E+04	9.80E-01	5.52E-02	6.99E-02	2.65	6.40	3.74
A0A1D3K566	2 (0)	10.0	NA	NA	NA	NA	NA	NA	NA	NA	NA	NA	NA	NA	NA	NA	NA	NA
A0A1D3K569	10 (3)	75.2	7.43E-04	1.27E+05	1.12E+05	1.27E+05	4.80E+04	5.81E+04	3.31E+04	7.63E+04	6.55E+04	4.57E+04	7.83E-04	2.79E-03	3.28E-01	-1.39	-0.96	0.43
A0A1D3K571	4 (1)	45.7	NA	NA	NA	NA	NA	NA	NA	NA	NA	NA	NA	NA	NA	NA	NA	NA
A0A1D3K577	2 (0)	12.8	NA	NA	NA	NA	NA	NA	NA	NA	NA	NA	NA	NA	NA	NA	NA	NA
A0A1D3K578	14 (3)	100.1	2.34E-02	5.04E+05	4.44E+05	4.85E+05	3.93E+05	3.92E+05	3.86E+05	4.10E+05	4.04E+05	3.18E+05	4.81E-02	2.78E-02	8.93E-01	-0.29	-0.34	-0.05
A0A1D3K579	4 (0)	30.1	NA	NA	NA	NA	NA	NA	NA	NA	NA	NA	NA	NA	NA	NA	NA	NA
A0A1D3K591	30 (8)	186.2	5.22E-01	6.48E+05	6.64E+05	7.13E+05	5.28E+05	5.77E+05	5.23E+05	1.15E+06	6.39E+05	4.51E+05	7.30E-01	9.13E-01	5.02E-01	-0.32	0.14	0.46
A0A1D3K596	3 (1)	15.1	2.49E-04	4.16E+04	3.43E+04	3.80E+04	9.74E+03	1.52E+04	4.60E+03	1.09E+04	9.50E+03	3.46E+03	5.31E-04	3.71E-04	8.60E-01	-1.95	-2.26	-0.31
A0A1D3K5A5	5 (1)	48.6	NA	NA	NA	NA	NA	NA	NA	NA	NA	NA	NA	NA	NA	NA	NA	NA
A0A1D3K5A9	4 (1)	20.4	1.59E-02	5.87E+04	4.79E+04	5.69E+04	2.66E+04	3.35E+04	1.79E+04	3.55E+04	2.93E+04	8.97E+03	2.74E-02	2.23E-02	9.83E-01	-1.07	-1.15	-0.08
A0A1D3K5B8	14 (1)	105.9	3.79E-04	2.53E+03	5.22E+03	2.93E+03	4.69E+02	7.82E+02	1.15E+03	9.85E+03	1.12E+04	1.44E+04	1.67E-01	1.79E-03	3.74E-04	-4.99	2.00	6.98
A0A1D3K5C8	2 (1)	11.1	1.19E-02	1.19E+04	1.32E+04	1.24E+04	1.01E+04	9.32E+03	1.02E+04	1.25E+04	1.27E+04	1.08E+04	1.29E-02	7.20E-01	3.17E-02	-0.34	-0.06	0.28
A0A1D3K5D1	12 (3)	61.5	NA	NA	NA	NA	NA	NA	NA	NA	NA	NA	NA	NA	NA	NA	NA	NA
A0A1D3K5D5	16 (5)	111.9	1.13E-02	8.93E+05	7.37E+05	8.62E+05	9.71E+05	1.03E+06	8.45E+05	1.15E+06	1.08E+06	1.10E+06	2.13E-01	9.39E-03	8.83E-02	0.19	0.42	0.23
A0A1D3K5D8	21 (10)	156.5	6.14E-04	2.50E+05	3.37E+05	2.98E+05	1.15E+05	1.28E+05	1.41E+05	1.08E+05	1.33E+05	7.38E+04	1.57E-03	7.85E-04	6.63E-01	-1.20	-1.49	-0.29
A0A1D3K5F0	3 (2)	21.2	1.90E-02	2.56E+04	1.65E+04	1.99E+04	4.70E+04	4.63E+04	4.55E+04	2.98E+04	5.38E+04	5.72E+04	3.07E-02	2.77E-02	9.96E-01	1.16	1.18	0.02
A0A1D3K5G0	11 (5)	99.7	2.12E-03	2.83E+05	2.17E+05	2.62E+05	4.24E+05	4.47E+05	3.80E+05	3.23E+05	3.45E+05	3.75E+05	1.73E-03	2.47E-02	7.95E-02	0.72	0.45	-0.26
A0A1D3K5H0	8 (2)	57.0	3.03E-01	1.24E+04	1.93E+04	1.08E+04	1.81E+04	2.34E+04	2.19E+04	1.14E+04	2.41E+04	2.52E+04	3.27E-01	4.09E-01	9.80E-01	0.57	0.51	-0.06
A0A1D3K5I5	18 (2)	110.7	2.95E-02	2.12E+04	1.84E+04	2.34E+04	1.79E+04	1.94E+04	1.32E+04	1.58E+04	9.42E+03	9.34E+03	3.10E-01	2.48E-02	1.84E-01	-0.32	-0.87	-0.55
A0A1D3K5J1	2 (0)	10.1	NA	NA	NA	NA	NA	NA	NA	NA	NA	NA	NA	NA	NA	NA	NA	NA
A0A1D3K5K2	7 (3)	38.2	2.74E-01	1.11E+05	1.25E+05	1.18E+05	1.11E+05	9.92E+04	1.36E+05	1.01E+05	1.09E+05	9.29E+04	9.62E-01	2.89E-01	3.95E-01	-0.03	-0.22	-0.19
A0A1D3K5K4	10 (2)	73.7	1.68E-01	1.61E+04	1.26E+04	1.36E+04	1.11E+04	1.17E+04	6.66E+03	1.41E+04	1.30E+04	9.31E+03	1.49E-01	5.94E-01	5.02E-01	-0.52	-0.22	0.30
A0A1D3K5K5	6 (0)	29.3	NA	NA	NA	NA	NA	NA	NA	NA	NA	NA	NA	NA	NA	NA	NA	NA
A0A1D3K5L2	27 (9)	240.8	1.88E-04	6.09E+05	4.93E+05	5.68E+05	8.86E+05	9.78E+05	9.50E+05	1.83E+06	1.40E+06	1.66E+06	3.05E-02	1.60E-04	1.78E-03	0.75	1.55	0.80
A0A1D3K5L4	3 (1)	24.7	3.55E-03	8.40E+04	5.56E+04	8.45E+04	1.27E+05	1.53E+05	9.96E+04	3.03E+05	1.89E+05	2.97E+05	3.39E-01	3.37E-03	1.58E-02	0.76	1.82	1.06
A0A1D3K5L7	3 (1)	17.7	3.25E-01	5.65E+03	3.28E+03	2.38E+03	3.11E+03	6.06E+03	4.42E+03	1.87E+03	3.60E+03	2.53E+03	7.88E-01	6.19E-01	3.00E-01	0.27	-0.50	-0.76
A0A1D3K5L8	21 (5)	166.8	1.54E-03	2.88E+05	2.70E+05	3.10E+05	3.48E+05	3.75E+05	3.43E+05	2.55E+05	2.55E+05	2.78E+05	7.79E-03	2.31E-01	1.44E-03	0.30	-0.14	-0.44
A0A1D3K5L9	4 (0)	27.9	NA	NA	NA	NA	NA	NA	NA	NA	NA	NA	NA	NA	NA	NA	NA	NA
A0A1D3K5M3	3 (1)	16.6	6.72E-01	1.67E+05	1.95E+05	1.87E+05	1.90E+05	1.79E+05	2.38E+05	1.66E+05	2.59E+05	1.95E+05	7.67E-01	6.80E-01	9.87E-01	0.15	0.18	0.03
A0A1D3K5M7	2 (0)	9.5	NA	NA	NA	NA	NA	NA	NA	NA	NA	NA	NA	NA	NA	NA	NA	NA
A0A1D3K5N7	2 (0)	5.7	NA	NA	NA	NA	NA	NA	NA	NA	NA	NA	NA	NA	NA	NA	NA	NA
A0A1D3K5N9	14 (3)	56.3	NA	NA	NA	NA	NA	NA	NA	NA	NA	NA	NA	NA	NA	NA	NA	NA
A0A1D3K5P0	3 (0)	22.2	NA	NA	NA	NA	NA	NA	NA	NA	NA	NA	NA	NA	NA	NA	NA	NA
A0A1D3K5P2	15 (5)	94.4	1.08E-02	4.06E+05	3.75E+05	4.38E+05	4.54E+05	4.34E+05	4.71E+05	5.03E+05	5.37E+05	6.17E+05	3.81E-01	9.71E-03	5.02E-02	0.16	0.44	0.29
A0A1D3K5P5	8 (2)	47.4	7.36E-02	6.41E+04	4.48E+04	5.66E+04	3.75E+04	3.91E+04	3.11E+04	5.63E+04	3.96E+04	4.07E+04	6.28E-02	3.81E-01	3.79E-01	-0.62	-0.28	0.34
A0A1D3K5Q0	14 (5)	84.0	3.34E-01	1.63E+05	1.69E+05	1.90E+05	1.53E+05	1.57E+05	1.44E+05	2.26E+05	1.87E+05	1.42E+05	5.66E-01	8.61E-01	3.17E-01	-0.20	0.09	0.29
A0A1D3K5Q1	3 (1)	22.3	9.12E-02	4.79E+04	3.67E+04	4.34E+04	4.36E+04	5.08E+04	3.26E+04	6.88E+04	6.42E+04	4.69E+04	9.99E-01	1.28E-01	1.21E-01	-0.01	0.49	0.50
A0A1D3K5R1	3 (1)	10.7	4.89E-05	1.50E+05	9.53E+04	1.02E+05	1.40E+06	1.74E+06	1.22E+06	2.66E+06	3.02E+06	3.49E+06	3.04E-03	3.85E-05	1.15E-03	3.65	4.72	1.08
A0A1D3K5R3	5 (1)	42.0	1.19E-01	1.13E+04	8.50E+03	9.41E+03	7.13E+03	7.75E+03	5.02E+03	8.96E+03	7.67E+03	5.17E+03	1.23E-01	2.25E-01	8.84E-01	-0.55	-0.42	0.13

A0A1D3K5R5	5 (2)	29.7	6.12E-01	4.44E+02	8.55E+03	9.98E+02	1.49E+03	8.67E+02	1.60E+04	1.11E+02	1.75E+03	2.32E+03	8.21E-01	9.09E-01	5.88E-01	0.88	-1.26	-2.14
A0A1D3K5S1	4 (1)	14.3	1.37E-03	4.15E+04	3.27E+04	3.65E+04	3.29E+04	3.37E+04	3.72E+04	8.07E+04	8.03E+04	1.14E+05	9.67E-01	2.60E-03	2.10E-03	-0.09	1.31	1.40
A0A1D3K5S6	5 (1)	42.4	5.61E-05	2.57E+03	2.48E+03	2.47E+03	1.91E+03	1.89E+03	1.36E+03	6.76E+02	4.67E+02	6.23E+02	5.87E-03	4.53E-05	8.80E-04	-0.54	-2.09	-1.55
A0A1D3K5S7	2 (1)	13.8	6.53E-01	4.51E+03	3.31E+03	2.96E+03	2.55E+03	3.77E+03	1.60E+03	2.84E+03	7.84E+03	1.90E+03	8.34E-01	9.30E-01	6.32E-01	-0.44	0.22	0.67
A0A1D3K5T3	13 (2)	42.5	NA	NA	NA	NA	NA	NA	NA	NA	NA	NA	NA	NA	NA	NA	NA	NA
A0A1D3K5U2	13 (2)	72.9	1.17E-05	2.27E+04	2.24E+04	2.37E+04	1.65E+04	1.59E+04	1.70E+04	1.18E+04	1.36E+04	1.15E+04	1.73E-04	9.05E-06	1.82E-03	-0.47	-0.90	-0.42
A0A1D3K5U9	2 (0)	10.4	NA	NA	NA	NA	NA	NA	NA	NA	NA	NA	NA	NA	NA	NA	NA	NA
A0A1D3K5Y5	5 (2)	30.4	1.70E-04	7.79E+03	6.56E+03	7.53E+03	1.61E+04	1.76E+04	1.02E+04	3.35E+04	2.74E+04	3.12E+04	4.89E-02	1.53E-04	1.21E-03	1.00	2.07	1.07
A0A1D3K5Z6	5 (3)	26.9	1.79E-02	2.62E+04	2.44E+04	2.83E+04	3.24E+04	3.19E+04	3.49E+04	2.82E+04	3.27E+04	2.94E+04	1.49E-02	1.30E-01	2.49E-01	0.33	0.19	-0.13
A0A1D3K5Z7	22 (8)	197.8	8.09E-04	3.51E+05	3.47E+05	3.31E+05	5.58E+05	5.65E+05	6.02E+05	4.62E+05	5.75E+05	5.70E+05	9.28E-04	2.49E-03	4.89E-01	0.75	0.64	-0.10
A0A1D3K5Z8	3 (0)	26.0	NA	NA	NA	NA	NA	NA	NA	NA	NA	NA	NA	NA	NA	NA	NA	NA
A0A1D3K607	9 (1)	49.0	9.32E-01	5.91E+04	7.15E+04	7.75E+04	6.75E+04	6.58E+04	6.87E+04	6.86E+04	8.11E+04	5.96E+04	9.53E-01	9.98E-01	9.35E-01	-0.04	0.01	0.05
A0A1D3K616	5 (1)	33.8	1.51E-02	2.35E+04	2.04E+04	2.16E+04	8.32E+03	9.75E+03	6.97E+03	1.25E+04	1.65E+04	2.54E+04	1.45E-02	5.39E-01	5.35E-02	-1.38	-0.26	1.12
A0A1D3K628	2 (1)	43.3	NA	NA	NA	NA	NA	NA	NA	NA	NA	NA	NA	NA	NA	NA	NA	NA
A0A1D3K635	7 (0)	39.2	NA	NA	NA	NA	NA	NA	NA	NA	NA	NA	NA	NA	NA	NA	NA	NA
A0A1D3K642	4 (0)	23.8	NA	NA	NA	NA	NA	NA	NA	NA	NA	NA	NA	NA	NA	NA	NA	NA
A0A1D3K653	4 (1)	20.2	7.47E-02	2.47E+04	2.16E+04	2.68E+04	2.43E+04	2.80E+04	2.32E+04	1.66E+04	2.27E+04	1.82E+04	9.41E-01	1.31E-01	8.58E-02	0.04	-0.35	-0.39
A0A1D3K654	16 (2)	88.5	5.70E-03	1.82E+05	1.71E+05	1.96E+05	1.95E+05	1.98E+05	1.90E+05	2.36E+05	2.45E+05	2.92E+05	7.46E-01	6.64E-03	1.45E-02	0.09	0.49	0.41
A0A1D3K666	2 (0)	13.0	NA	NA	NA	NA	NA	NA	NA	NA	NA	NA	NA	NA	NA	NA	NA	NA
A0A1D3K671	12 (2)	91.8	5.89E-02	1.27E+05	1.12E+05	1.09E+05	7.59E+04	8.49E+04	6.30E+04	1.18E+05	9.34E+04	6.91E+04	5.01E-02	2.97E-01	3.92E-01	-0.64	-0.31	0.33
A0A1D3K673	17 (4)	101.1	8.07E-01	6.14E+05	4.98E+05	5.71E+05	5.51E+05	5.88E+05	4.35E+05	6.39E+05	4.84E+05	4.29E+05	8.66E-01	8.14E-01	9.94E-01	-0.10	-0.12	-0.02
A0A1D3K674	5 (3)	29.0	7.53E-02	3.99E+05	3.96E+05	4.59E+05	2.97E+05	3.22E+05	3.05E+05	3.90E+05	3.11E+05	2.16E+05	1.08E-01	1.01E-01	9.98E-01	-0.44	-0.45	-0.01
A0A1D3K679	2 (1)	5.0	2.89E-03	3.90E+03	3.12E+03	3.70E+03	1.51E+03	1.67E+03	1.21E+03	3.38E+03	3.15E+03	2.24E+03	2.62E-03	2.42E-01	1.58E-02	-1.29	-0.29	1.00
A0A1D3K684	3 (0)	22.1	NA	NA	NA	NA	NA	NA	NA	NA	NA	NA	NA	NA	NA	NA	NA	NA
A0A1D3K692	2 (0)	10.2	NA	NA	NA	NA	NA	NA	NA	NA	NA	NA	NA	NA	NA	NA	NA	NA
A0A1D3K696	3 (1)	52.5	NA	NA	NA	NA	NA	NA	NA	NA	NA	NA	NA	NA	NA	NA	NA	NA
A0A1D3K699	6 (1)	46.6	3.51E-03	1.75E+05	1.43E+05	1.68E+05	2.26E+05	2.36E+05	2.05E+05	2.25E+05	2.12E+05	2.35E+05	6.20E-03	5.48E-03	9.91E-01	0.46	0.47	0.01
A0A1D3K6A1	19 (8)	134.3	9.06E-01	2.66E+05	2.21E+05	2.48E+05	2.57E+05	2.65E+05	2.14E+05	3.18E+05	2.48E+05	2.06E+05	1.00E+00	9.19E-01	9.24E-01	0.00	0.07	0.07
A0A1D3K6A5	10 (4)	62.2	6.72E-03	9.51E+05	7.66E+05	9.41E+05	5.67E+05	5.86E+05	4.71E+05	7.13E+05	5.41E+05	5.62E+05	7.36E-03	1.90E-02	6.67E-01	-0.71	-0.55	0.16
A0A1D3K6A7	4 (2)	22.2	4.04E-04	1.99E+04	1.28E+04	1.65E+04	4.01E+04	4.21E+04	3.50E+04	3.82E+04	3.24E+04	3.80E+04	5.13E-04	1.07E-03	6.02E-01	1.25	1.14	-0.11
A0A1D3K6A9	5 (1)	30.2	5.27E-01	4.49E+02	3.17E+03	6.81E+02	1.09E+03	3.21E+02	3.11E+03	6.19E+02	3.10E+02	4.25E+02	9.97E-01	6.06E-01	5.65E-01	0.07	-1.67	-1.74
A0A1D3K6E1	2 (0)	10.9	NA	NA	NA	NA	NA	NA	NA	NA	NA	NA	NA	NA	NA	NA	NA	NA
A0A1D3K6E4	6 (1)	49.1	2.06E-01	3.22E+03	6.91E+03	4.63E+03	2.46E+03	2.26E+03	5.70E+03	8.22E+02	3.22E+03	2.24E+03	5.79E-01	1.84E-01	6.06E-01	-0.50	-1.23	-0.73
A0A1D3K6E7	4 (0)	21.8	NA	NA	NA	NA	NA	NA	NA	NA	NA	NA	NA	NA	NA	NA	NA	NA
A0A1D3K6F2	4 (1)	20.8	1.40E-03	6.69E+03	5.99E+03	7.22E+03	2.61E+03	1.93E+03	1.65E+03	3.48E+03	1.30E+03	6.94E+02	2.73E-03	2.10E-03	9.51E-01	-1.68	-1.86	-0.18
A0A1D3K6H5	6 (1)	48.5	NA	NA	NA	NA	NA	NA	NA	NA	NA	NA	NA	NA	NA	NA	NA	NA
A0A1D3K6I0	14 (3)	100.3	8.76E-02	2.00E+05	1.71E+05	1.90E+05	1.37E+05	1.41E+05	1.15E+05	1.86E+05	1.40E+05	1.04E+05	8.92E-02	1.84E-01	8.41E-01	-0.51	-0.38	0.13
A0A1D3K6L5	3 (0)	16.3	NA	NA	NA	NA	NA	NA	NA	NA	NA	NA	NA	NA	NA	NA	NA	NA
A0A1D3K6M0	2 (0)	10.6	NA	NA	NA	NA	NA	NA	NA	NA	NA	NA	NA	NA	NA	NA	NA	NA
A0A1D3K6N0	6 (1)	35.5	4.35E-02	1.35E+03	2.77E+03	1.37E+03	3.13E+03	2.79E+03	2.69E+03	3.17E+03	3.09E+03	4.33E+03	1.89E-01	3.78E-02	4.55E-01	0.65	0.95	0.30
A0A1D3K6N5	29 (5)	236.0	1.33E-01	4.19E+05	4.23E+05	4.73E+05	4.95E+05	4.98E+05	5.36E+05	4.07E+05	5.11E+05	4.67E+05	1.23E-01	7.30E-01	3.25E-01	0.22	0.08	-0.14
A0A1D3K6Q5	4 (0)	29.3	NA	NA	NA	NA	NA	NA	NA	NA	NA	NA	NA	NA	NA	NA	NA	NA
A0A1D3K6Q6	34 (13)	258.5	1.43E-02	1.38E+06	1.25E+06	1.31E+06	1.34E+06	1.43E+06	1.37E+06	1.20E+06	1.16E+06	1.00E+06	5.35E-01	5.09E-02	1.37E-02	0.08	-0.23	-0.30
A0A1D3K6R9	17 (7)	121.9	2.57E-01	1.90E+04	4.14E+04	2.00E+04	3.67E+04	3.34E+04	6.59E+04	1.26E+04	3.71E+04	2.78E+04	3.29E-01	9.96E-01	2.99E-01	0.76	-0.05	-0.81
A0A1D3K6U7	9 (6)	67.5	8.05E-01	4.83E+05	3.33E+05	4.14E+05	3.98E+05	4.17E+05	3.16E+05	5.08E+05	4.00E+05	3.34E+05	8.52E-01	9.98E-01	8.20E-01	-0.12	0.01	0.13
A0A1D3K6V4	4 (0)	56.7	NA	NA	NA	NA	NA	NA	NA	NA	NA	NA	NA	NA	NA	NA	NA	NA
A0A1D3K6V8	15 (2)	58.4	NA	NA	NA	NA	NA	NA	NA	NA	NA	NA	NA	NA	NA	NA	NA	NA
A0A1D3K6V9	11 (3)	58.3	4.70E-01	6.27E+04	6.81E+04	7.44E+04	6.14E+04	6.04E+04	7.37E+04	7.38E+04	8.04E+04	6.43E+04	8.48E-01	7.44E-01	4.42E-01	-0.07	0.09	0.16
A0A1D3K6X1	2 (0)	9.6	NA	NA	NA	NA	NA	NA	NA	NA	NA	NA	NA	NA	NA	NA	NA	NA
A0A1D3K710	2 (0)	14.4	NA	NA	NA	NA	NA	NA	NA	NA	NA	NA	NA	NA	NA	NA	NA	NA
A0A1D3K723	2 (0)	9.7	NA	NA	NA	NA	NA	NA	NA	NA	NA	NA	NA	NA	NA	NA	NA	NA
A0A1D3K730	7 (2)	53.9	1.22E-02	7.81E+04	8.01E+04	7.57E+04	9.60E+04	9.83E+04	1.01E+05	1.10E+05	1.29E+05	1.62E+05	3.09E-01	1.05E-02	6.88E-02	0.34	0.78	0.44

A0A1D3K738	2 (1)	49.3	NA	NA	NA	NA	NA	NA	NA	NA	NA	NA	NA	NA	NA	NA	NA	NA
A0A1D3K740	4 (0)	25.7	NA	NA	NA	NA	NA	NA	NA	NA	NA	NA	NA	NA	NA	NA	NA	NA
A0A1D3K765	14 (4)	62.8	NA	NA	NA	NA	NA	NA	NA	NA	NA	NA	NA	NA	NA	NA	NA	NA
A0A1D3K7B5	5 (1)	24.6	1.39E-02	3.75E+03	4.22E+03	4.14E+03	2.68E+03	1.92E+03	4.32E+03	9.94E+00	1.66E+03	1.24E+03	3.61E-01	1.22E-02	6.98E-02	-0.44	-2.06	-1.62
A0A1D3K7D2	5 (1)	83.5	NA	NA	NA	NA	NA	NA	NA	NA	NA	NA	NA	NA	NA	NA	NA	NA
A0A1D3K7I1	12 (3)	62.6	2.28E-03	7.06E+03	8.21E+03	1.17E+04	1.55E+04	1.52E+04	1.74E+04	1.63E+04	1.77E+04	1.57E+04	4.71E-03	3.19E-03	9.09E-01	0.87	0.95	0.08
A0A1D3K7N6	2 (1)	51.2	NA	NA	NA	NA	NA	NA	NA	NA	NA	NA	NA	NA	NA	NA	NA	NA
A0A1D3K7P1	2 (0)	9.0	NA	NA	NA	NA	NA	NA	NA	NA	NA	NA	NA	NA	NA	NA	NA	NA
A0A1D3K7S1	4 (1)	56.3	NA	NA	NA	NA	NA	NA	NA	NA	NA	NA	NA	NA	NA	NA	NA	NA
A0A1D3K7T3	2 (0)	14.6	NA	NA	NA	NA	NA	NA	NA	NA	NA	NA	NA	NA	NA	NA	NA	NA
A0A1D3K7Z3	2 (1)	5.9	1.54E-01	5.97E+04	4.27E+04	4.80E+04	3.76E+04	3.88E+04	2.69E+04	4.91E+04	2.66E+04	2.38E+04	2.20E-01	1.83E-01	9.88E-01	-0.54	-0.60	-0.05
A0A1D3K820	2 (0)	15.5	NA	NA	NA	NA	NA	NA	NA	NA	NA	NA	NA	NA	NA	NA	NA	NA
A0A1D3K825	5 (1)	56.2	NA	NA	NA	NA	NA	NA	NA	NA	NA	NA	NA	NA	NA	NA	NA	NA
A0A1D3K830	6 (1)	49.5	NA	NA	NA	NA	NA	NA	NA	NA	NA	NA	NA	NA	NA	NA	NA	NA
A0A1D3K842	4 (0)	16.2	NA	NA	NA	NA	NA	NA	NA	NA	NA	NA	NA	NA	NA	NA	NA	NA
A0A1D3K887	3 (1)	9.8	7.06E-02	4.02E+03	5.06E+03	5.48E+03	9.68E+03	9.99E+03	1.14E+04	1.19E+03	5.90E+03	9.55E+03	8.13E-02	9.40E-01	1.25E-01	1.09	0.19	-0.90
A0A1D3K8A2	2 (0)	11.2	NA	NA	NA	NA	NA	NA	NA	NA	NA	NA	NA	NA	NA	NA	NA	NA
A0A1D3K8B8	6 (1)	48.3	NA	NA	NA	NA	NA	NA	NA	NA	NA	NA	NA	NA	NA	NA	NA	NA
A0A1D3K8L4	2 (0)	9.5	NA	NA	NA	NA	NA	NA	NA	NA	NA	NA	NA	NA	NA	NA	NA	NA
A0A1D3K8L6	4 (1)	51.3	NA	NA	NA	NA	NA	NA	NA	NA	NA	NA	NA	NA	NA	NA	NA	NA
A0A1D3K8Q8	13 (3)	67.5	NA	NA	NA	NA	NA	NA	NA	NA	NA	NA	NA	NA	NA	NA	NA	NA
A0A1D3K8S2	3 (0)	15.3	NA	NA	NA	NA	NA	NA	NA	NA	NA	NA	NA	NA	NA	NA	NA	NA
A0A1D3K8V4	2 (0)	10.6	NA	NA	NA	NA	NA	NA	NA	NA	NA	NA	NA	NA	NA	NA	NA	NA
A0A1D3K906	2 (1)	9.9	2.07E-04	1.62E+03	1.72E+03	2.11E+03	4.20E+03	4.91E+03	5.03E+03	3.83E+03	4.25E+03	4.70E+03	2.45E-04	6.40E-04	3.80E-01	1.50	0.99	-0.51
A0A1D3K908	3 (1)	56.2	NA	NA	NA	NA	NA	NA	NA	NA	NA	NA	NA	NA	NA	NA	NA	NA
A0A1D3K930	27 (8)	51.6	4.14E-04	2.50E+04	2.71E+04	2.64E+04	3.05E+04	2.93E+04	2.30E+04	4.33E+04	4.27E+04	4.06E+04	7.76E-01	5.75E-04	9.55E-04	0.17	0.67	0.50
A0A1D3K961	3 (1)	37.5	NA	NA	NA	NA	NA	NA	NA	NA	NA	NA	NA	NA	NA	NA	NA	NA
A0A1D3K963	2 (0)	16.3	NA	NA	NA	NA	NA	NA	NA	NA	NA	NA	NA	NA	NA	NA	NA	NA
A0A1D3K967	11 (0)	64.1	NA	NA	NA	NA	NA	NA	NA	NA	NA	NA	NA	NA	NA	NA	NA	NA
A0A1D3K973	13 (4)	51.6	NA	NA	NA	NA	NA	NA	NA	NA	NA	NA	NA	NA	NA	NA	NA	NA
A0A1D3K985	12 (3)	49.5	NA	NA	NA	NA	NA	NA	NA	NA	NA	NA	NA	NA	NA	NA	NA	NA
A0A1D3K990	5 (1)	31.5	NA	NA	NA	NA	NA	NA	NA	NA	NA	NA	NA	NA	NA	NA	NA	NA
A0A1D3K9A2	4 (2)	27.6	3.97E-02	3.86E+04	6.69E+04	3.61E+04	3.50E+04	1.86E+04	3.74E+04	1.18E+04	2.06E+04	7.75E+03	2.81E-01	3.33E-02	2.79E-01	-0.64	-1.81	-1.18
A0A1D3K9A5	3 (0)	36.7	NA	NA	NA	NA	NA	NA	NA	NA	NA	NA	NA	NA	NA	NA	NA	NA
A0A1D3K9C6	8 (2)	56.6	NA	NA	NA	NA	NA	NA	NA	NA	NA	NA	NA	NA	NA	NA	NA	NA
A0A1D3K9G1	4 (1)	19.9	2.27E-02	1.54E+04	1.21E+04	1.60E+04	1.26E+04	1.21E+04	1.00E+04	1.74E+04	2.75E+04	2.05E+04	5.57E-01	7.93E-02	2.14E-02	-0.33	0.59	0.91
A0A1D3K9G5	21 (5)	63.7	6.70E-01	6.04E+04	5.91E+04	8.39E+04	0.00E+00	1.55E+05	1.45E+05	1.07E+05	9.70E+04	1.02E+05	7.31E-01	7.03E-01	9.99E-01	0.56	0.59	0.03
A0A1D3K9L9	6 (0)	39.0	NA	NA	NA	NA	NA	NA	NA	NA	NA	NA	NA	NA	NA	NA	NA	NA
A0A1D3K9P9	14 (4)	62.5	NA	NA	NA	NA	NA	NA	NA	NA	NA	NA	NA	NA	NA	NA	NA	NA
A0A1D3K9V8	3 (1)	15.3	1.79E-01	5.31E+04	4.56E+04	6.13E+04	4.38E+04	4.84E+04	3.90E+04	5.09E+04	3.88E+04	3.68E+04	2.78E-01	1.95E-01	9.57E-01	-0.29	-0.34	-0.05
A0A1D3K9Y9	69 (9)	61.5	NA	NA	NA	NA	NA	NA	NA	NA	NA	NA	NA	NA	NA	NA	NA	NA
A0A1D3K9Z4	3 (0)	13.7	NA	NA	NA	NA	NA	NA	NA	NA	NA	NA	NA	NA	NA	NA	NA	NA
A0A1D3KA55	4 (1)	51.5	NA	NA	NA	NA	NA	NA	NA	NA	NA	NA	NA	NA	NA	NA	NA	NA
A0A1D3KA82	2 (0)	9.9	NA	NA	NA	NA	NA	NA	NA	NA	NA	NA	NA	NA	NA	NA	NA	NA
A0A1D3KA96	3 (1)	42.4	NA	NA	NA	NA	NA	NA	NA	NA	NA	NA	NA	NA	NA	NA	NA	NA
A0A1D3KAA8	2 (0)	11.6	NA	NA	NA	NA	NA	NA	NA	NA	NA	NA	NA	NA	NA	NA	NA	NA
A0A1D3KAD4	3 (0)	24.1	NA	NA	NA	NA	NA	NA	NA	NA	NA	NA	NA	NA	NA	NA	NA	NA
A0A1D3KAD8	5 (0)	30.8	NA	NA	NA	NA	NA	NA	NA	NA	NA	NA	NA	NA	NA	NA	NA	NA
A0A1D3KAH4	2 (1)	15.6	3.53E-01	1.60E+04	1.01E+04	1.25E+04	1.56E+04	1.61E+04	1.26E+04	1.56E+04	1.77E+04	1.41E+04	6.12E-01	3.31E-01	8.38E-01	0.19	0.29	0.10
A0A1D3KAH6	4 (1)	20.2	3.30E-01	2.23E+03	5.07E+03	2.80E+03	2.12E+03	1.15E+03	4.87E+03	3.28E+01	2.63E+03	1.16E+03	8.73E-01	3.16E-01	5.49E-01	-0.31	-1.40	-1.09
A0A1D3KAI3	3 (0)	14.5	NA	NA	NA	NA	NA	NA	NA	NA	NA	NA	NA	NA	NA	NA	NA	NA
A0A1D3KAI6	18 (4)	48.5	NA	NA	NA	NA	NA	NA	NA	NA	NA	NA	NA	NA	NA	NA	NA	NA

A0A1D3KAI8	2 (1)	51.8	NA	NA	NA	NA	NA	NA	NA	NA	NA	NA	NA	NA	NA	NA	NA	NA
A0A1D3KAJ5	2 (1)	9.1	2.47E-01	1.00E+05	9.37E+04	1.12E+05	1.11E+05	1.14E+05	1.12E+05	1.15E+05	1.07E+05	1.02E+05	2.25E-01	5.49E-01	7.28E-01	0.14	0.08	-0.06
A0A1D3KAP2	2 (0)	8.8	NA	NA	NA	NA	NA	NA	NA	NA	NA	NA	NA	NA	NA	NA	NA	NA

Supplementary Table S4. Annotations of identified and quantified proteins of *P. veronii* . Matched against Pseudomonas veronii 1YdBTEX2 reference strain. Functional distribution: C-Energy production and conversion; D-Cell cycle control, cell division, chromosome partitioning; E-Amino acid metabolism and transport; F-Nucleotide metabolism and transport; H-Coenzyme transport and metabolism; I-Lipid transport and metabolism; K-Transcription; M-Cell wall/membrane/envelop biogenesis; N-Cell motility; O-Post-translational modification, protein turnover, and chaperone functions; P-Inorganic ion transport and metabolism; Q-Secondary metabolites biosynthesis, transport, and catabolism; T-Signal transduction mechanisms; U-Intracellular trafficking, secretion, and vesicular transport; V-Defense mechanisms; S-Function unknown; MX-Multiple functions (more than 3). Localization: CY-cytoplasm; IM-inner membrane; PR-periplasm; OM-outer membrane; EX-extracellular; UK-unknown; ML-multiple localizations. Y - identified.

UNIPROT Accession	Protein name	Identified in			MW [Da]	pI	Localizati SOSUIGramN	PSORTb	Function COGS
		Control	Exp_15days	Exp_30days					
A0A1D3JPF4	Beta sliding clamp OS=Pseudomonas veronii 1YdBTEX2	Y	Y	Y	40518	4.9043	CY	CY	L
A0A1D3JPI1	Glycine/betaine ABC transporter substrate-binding protein OS=Pseudomonas veronii 1YdBTEX2		Y		33114	5.5752	CY	CY	ET
A0A1D3JPJ0	Hypothetical membrane protein OS=Pseudomonas veronii 1YdBTEX2	Y	Y		11711	7.396	CY	UK	T
A0A1D3JPK6	Cytochrome c oxidase subunit 2 OS=Pseudomonas veronii 1YdBTEX2	Y	Y	Y	38671.82	6.23	IM	ML (C, IM)	C
A0A1D3JPL1	Peroxiredoxin OsmC OS=Pseudomonas veronii 1YdBTEX2	Y	Y	Y	14856	5.1709	CY	CY	O
A0A1D3JPL2	DNA gyrase subunit B OS=Pseudomonas veronii 1YdBTEX2	Y	Y	Y	90406.19	5.52	CY	CY	L
A0A1D3JPL6	Probable D-methionine-binding lipoprotein MetQ OS=Pseudomonas veronii 1YdBTEX2	Y	Y	Y	27869	7.8135	PR	IM	P
A0A1D3JPN3	Ferric uptake regulation protein OS=Pseudomonas veronii 1YdBTEX2			Y	17321	6.583	CY	CY	P
A0A1D3JPP4	Short-chain dehydrogenase OS=Pseudomonas veronii 1YdBTEX2	Y	Y	Y	26581	6.4263	CY	CY	IQ
A0A1D3JPP9	Cytochrome c4 OS=Pseudomonas veronii 1YdBTEX2		Y		20884	7.2891	PR	PR	C
A0A1D3JPQ0	ABC transporter permease OS=Pseudomonas veronii 1YdBTEX2			Y	33575	7.8237	OM	UK	QT
A0A1D3JPQ1	Cytochrome P450 OS=Pseudomonas veronii 1YdBTEX2		Y	Y	44493	5.6118	CY	CY	CQ
A0A1D3JPQ6|	Shikimate dehydrogenase (NADP(+)) OS=Pseudomonas veronii 1YdBTEX2			Y	29089	4.8405	CY	CY	EH

A0A1D3JPR1	Glycine--tRNA ligase alpha subunit OS=Pseudomonas veronii 1YdBTEX2	Y	Y	Y	36210	4.749	UK	CY	J
A0A1D3JPR5	Thiol:disulfide interchange protein OS=Pseudomonas veronii 1YdBTEX2	Y			22935	7.3125	PR	PR	CQ
A0A1D3JPS1	Methyl-accepting chemotaxis protein PctA OS=Pseudomonas veronii 1YdBTEX2	Y			68696	4.8809	IM	IM	NT(mx)
A0A1D3JPS8	LysR family transcriptional regulator OS=Pseudomonas veronii 1YdBTEX2			Y	33973	6.7852	CY	CY	K
A0A1D3JPT2	D-galactonate dehydratase OS=Pseudomonas veronii 1YdBTEX2	Y	Y	Y	42130	5.0024	CY	CY	M
A0A1D3JPT7	Taurine dioxygenase OS=Pseudomonas veronii 1YdBTEX2	Y			33467	6.5186	CY	CY	Q
A0A1D3JPT9	Transporter OS=Pseudomonas veronii 1YdBTEX2	Y		Y	55429.58	6.3	PR	UK	MU
A0A1D3JPU4	Glutaryl-CoA dehydrogenase_ mitochondrial OS=Pseudomonas veronii 1YdBTEX2	Y	Y	Y	43127	5.9297	CY	CY	IC
A0A1D3JPU5	Glycine--tRNA ligase beta subunit OS=Pseudomonas veronii 1YdBTEX2	Y	Y		74930	5.2134	CY	CY	J
A0A1D3JPU8	Uncharacterized protein OS=Pseudomonas veronii 1YdBTEX2	Y	Y	Y	52599	4.7754	IM	IM	S
A0A1D3JPX3	Catalase OS=Pseudomonas veronii 1YdBTEX2	Y	Y	Y	78223	6.4277	CY	CY	C
A0A1D3JPY5	Hypothetical secreted protein OS=Pseudomonas veronii 1YdBTEX2	Y	Y	Y	9128.72	4.96	UK	UK	M
A0A1D3JPY6	Curli production assembly/transport component CsgG OS=Pseudomonas veronii 1YdBTEX2		Y	Y	24045	9.3105	OM	UK	M
A0A1D3JPY9	5-aminovalerate aminotransferase DavT OS=Pseudomonas veronii 1YdBTEX2	Y		Y	44808	6.0176	CY	CY	EH(mx)
A0A1D3JPZ3	Membrane protein OS=Pseudomonas veronii 1YdBTEX2	Y	Y	Y	88292.62	5.31	OM	OM	NU(mx)
A0A1D3JPZ6	Succinate--hydroxymethylglutarate CoA-transferase OS=Pseudomonas veronii 1YdBTEX2	Y	Y	Y	43230	5.5151	CY	CY	C
A0A1D3JQ04	L-glyceraldehyde 3-phosphate reductase OS=Pseudomonas veronii 1YdBTEX2	Y	Y		37980.77	6.2	CY	CY	C
A0A1D3JQ11	Butanediol dehydrogenase OS=Pseudomonas veronii 1YdBTEX2		Y	Y	38566	5.6162	UK	CY	CE(mx)

A0A1D3JQ20	Transcriptional regulatory protein OmpR OS=Pseudomonas veronii 1YdBTEX2	Y		Y	27327	5.5371	CY	CY	KT
A0A1D3JQ23	Serine O-acetyltransferase OS=Pseudomonas veronii 1YdBTEX2	Y	Y	Y	33408.43	6.15	CY	CY	EQ
A0A1D3JQ27	Cystine-binding periplasmic protein OS=Pseudomonas veronii 1YdBTEX2	Y	Y	Y	28611	9.271	CY	PR	ET
A0A1D3JQ35	Haemagg_act domain-containing protein OS=Pseudomonas veronii 1YdBTEX2	Y	Y	Y	237969.4	5.73	OM	ML (Ex, OM	UT(mx)
A0A1D3JQ49	Probable D-methionine-binding lipoprotein MetQ OS=Pseudomonas veronii 1YdBTEX2	Y	Y	Y	25944.02	6.96	PR	IM	P
A0A1D3JQ52	Alpha-ketoglutarate-dependent taurine dioxygenase OS=Pseudomonas veronii 1YdBTEX2	Y			31131	5.7656	CY	CY	Q
A0A1D3JQ54	Uncharacterized protein OS=Pseudomonas veronii 1YdBTEX2		Y		28458.57	5.82	CY	CY	H
A0A1D3JQ60	Uncharacterized protein OS=Pseudomonas veronii 1YdBTEX2	Y			16406.15	5.3	EX	UK	X
A0A1D3JQ61	Glucose-1-phosphate thymidylyltransferase OS=Pseudomonas veronii 1YdBTEX2	Y	Y	Y	32222	5.0479	IM	CY	JM
A0A1D3JQ62	Peptidoglycan-binding protein LysM OS=Pseudomonas veronii 1YdBTEX2	Y	Y	Y	15612	5.2266	CY	UK	NUD
A0A1D3JQ68	Glutarate-semialdehyde dehydrogenase DavD OS=Pseudomonas veronii 1YdBTEX2	Y	Y	Y	51524	5.4873	CY	CY	C
A0A1D3JQ71	dTDP-glucose 4_6-dehydratase OS=Pseudomonas veronii 1YdBTEX2	Y	Y	Y	40098	5.2251	CY	CY	MG
A0A1D3JQ78	Sulfate/thiosulfate import ATP-binding protein CysA OS=Pseudomonas veronii 1YdBTEX2	Y	Y	Y	36633.05	6.1	CY	IM	PQ(mx)
A0A1D3JQ85	Amino acid ABC transporter OS=Pseudomonas veronii 1YdBTEX2	Y	Y	Y	27708	5.2778	EX	PR	ET
A0A1D3JQ86	D-cysteine desulfhydrase OS=Pseudomonas veronii 1YdBTEX2		Y		35083	4.6743	CY	CY	E
A0A1D3JQ87	Cell envelope biogenesis protein AsmA OS=Pseudomonas veronii 1YdBTEX2	Y	Y		79821.25	6.41	IM	OM	MD

A0A1D3JQA3	Uncharacterized protein OS=Pseudomonas veronii 1YdBTEX2	Y			16484	8.9458	UK	UK	S
A0A1D3JQA5	Carboxy-terminal-processing protease OS=Pseudomonas veronii 1YdBTEX2		Y		46345	5.7466	UK	IM	MO
A0A1D3JQA7	Phosphoenolpyruvate carboxykinase (ATP) OS=Pseudomonas veronii 1YdBTEX2	Y	Y		55316	5.1753	UK	CY	H
A0A1D3JQB0	Glutamine synthetase OS=Pseudomonas veronii 1YdBTEX2	Y	Y	Y	51647	4.9526	CY	CY	E
A0A1D3JQB2	Porin D OS=Pseudomonas veronii 1YdBTEX2	Y	Y	Y	46511	6.1582	OM	OM	S
A0A1D3JQC6	TetR family transcriptional regulator OS=Pseudomonas veronii 1YdBTEX2	Y	Y		20041	5.2046	CY	CY	K
A0A1D3JQD1	Histidine ammonia-lyase OS=Pseudomonas veronii 1YdBTEX2	Y	Y	Y	53882	5.3525	UK	CY	E
A0A1D3JQD4	Histidine ABC transporter substrate-binding protein OS=Pseudomonas veronii 1YdBTEX2	Y	Y	Y	35088	6.0117	PR	PR	E
A0A1D3JQE3	Glucans biosynthesis protein G OS=Pseudomonas veronii 1YdBTEX2	Y	Y		65087	6.1816	OM	PR	P
A0A1D3JQE4	GTP-binding protein TypA/BipA OS=Pseudomonas veronii 1YdBTEX2	Y	Y	Y	67050	5.2002	CY	CY	TJ(mx)
A0A1D3JQE6	Channel protein TolC OS=Pseudomonas veronii 1YdBTEX2		Y	Y	49965	6.0278	OM	OM	MU
A0A1D3JQE9	Amino acid ABC transporter substrate-binding protein OS=Pseudomonas veronii 1YdBTEX2	Y	Y		29769	5.978	PR	PR	ET
A0A1D3JQG4	2_3-bisphosphoglycerate-independent phosphoglycerate mutase OS=Pseudomonas veronii 1YdBTEX2	Y	Y		54919	5.1167	CY	CY	GP
A0A1D3JQH2	50S ribosomal protein L31 OS=Pseudomonas veronii 1YdBTEX2	Y	Y	Y	8322	8.4478	CY	CY	J
A0A1D3JQH4	Methyl-accepting chemotaxis protein McpS OS=Pseudomonas veronii 1YdBTEX2	Y	Y	Y	68360	4.8105	IM	IM	NT(mx)
A0A1D3JQJ5	Fructose-1_6-bisphosphatase class 1 OS=Pseudomonas veronii 1YdBTEX2	Y			37150.81	5.66	CY	CY	G
A0A1D3JQK4	Alpha-1_4 glucan phosphorylase OS=Pseudomonas veronii 1YdBTEX2	Y	Y	Y	91798	5.792	CY	CY	G
A0A1D3JQK6	Chemotaxis protein OS=Pseudomonas veronii 1YdBTEX2	Y			58603	4.667	IM	IM	NT(mx)

A0A1D3JQL2	Acetyltransferase component of pyruvate dehydrogenase complex OS=Pseudomonas veronii 1YdBTEX2	Y	Y	Y	56142	5.4302	UK	CY	C
A0A1D3JQL3	Peptide methionine sulfoxide reductase MsrA OS=Pseudomonas veronii 1YdBTEX2	Y	Y	Y	23472	4.6597	UK	UK	O
A0A1D3JQM4	Cobyric acid synthase OS=Pseudomonas veronii 1YdBTEX2	Y		Y	28585	4.938	UK	CY	D
A0A1D3JQM5	ATP-dependent protease ATPase subunit HslU OS=Pseudomonas veronii 1YdBTEX2	Y	Y		49580	5.5063	CY	CY	OL
A0A1D3JQN4	Protein-export protein SecB OS=Pseudomonas veronii 1YdBTEX2	Y	Y	Y	17600	4.2979	CY	CY	U
A0A1D3JQP1	Poly(3-hydroxyalkanoate) granule-associated protein PhaF OS=Pseudomonas veronii 1YdBTEX2	Y	Y		28565	10.8486	CY	CY	S
A0A1D3JQP4	Pyruvate dehydrogenase E1 component OS=Pseudomonas veronii 1YdBTEX2	Y	Y	Y	99244	5.3584	CY	CY	CG
A0A1D3JQP5	NADP-dependent malic enzyme OS=Pseudomonas veronii 1YdBTEX2	Y	Y	Y	44964	4.9526	UK	CY	C
A0A1D3JQQ0	Urocanate hydratase OS=Pseudomonas veronii 1YdBTEX2	Y	Y		61323	5.5034	CY	CY	E
A0A1D3JQR5	Proline iminopeptidase OS=Pseudomonas veronii 1YdBTEX2	Y	Y		36759	5.5693	CY	CY	EI(mx)
A0A1D3JQT2	Chemotaxis protein OS=Pseudomonas veronii 1YdBTEX2	Y		Y	67606	5.4946	IM	IM	NT(mx)
A0A1D3JQT8	50S ribosomal protein L9 OS=Pseudomonas veronii 1YdBTEX2	Y	Y	Y	15431	5.2646	CY	CY	J
A0A1D3JQU0	O-acetylhomoserine (Thiol)-lyase OS=Pseudomonas veronii 1YdBTEX2	Y			45683	5.8213	CY	CY	EP
A0A1D3JQU3	RNA-binding protein Hfq OS=Pseudomonas veronii 1YdBTEX2	Y	Y	Y	9404	9.2314	CY	CY	S
A0A1D3JQU4	Protein HflC OS=Pseudomonas veronii 1YdBTEX2	Y	Y	Y	32896	9.293	IM	CY	O
A0A1D3JQU6	Arginine--tRNA ligase OS=Pseudomonas veronii 1YdBTEX2	Y	Y	Y	63767	5.5737	CY	CY	J
A0A1D3JQU8	NH(3)-dependent NAD(+) synthetase OS=Pseudomonas veronii 1YdBTEX2	Y	Y	Y	29844	5.2134	CY	CY	H
A0A1D3JQV8	Shikimate kinase OS=Pseudomonas veronii 1YdBTEX2	Y			18280	8.8931	CY	CY	FG
A0A1D3JQW0	Glycosyl transferase OS=Pseudomonas veronii 1YdBTEX2	Y			33315.45	7.82	CY	ML (C, IM)	MJ
A0A1D3JQW6	DUF3298 domain-containing protein OS=Pseudomonas veronii 1YdBTEX2		Y		25178.01	7.14	PR	UK	G
A0A1D3JQW8	GTPase HflX OS=Pseudomonas veronii 1YdBTEX2	Y			49021.83	5.64	UK	CY	OT

A0A1D3JQX6	30S ribosomal protein S6 OS=Pseudomonas veronii 1YdBTEX2	Y	Y	Y	16289	4.7388	CY	CY	J
A0A1D3JQX8	Channel protein TolC OS=Pseudomonas veronii 1YdBTEX2	Y	Y	Y	52670	5.6777	OM	OM	MU
A0A1D3JQZ1	Thioredoxin OS=Pseudomonas veronii 1YdBTEX2	Y	Y		15757	8.6543	CY	CY	CO
A0A1D3JQZ2	Adenylosuccinate synthetase OS=Pseudomonas veronii 1YdBTEX2	Y	Y		46557	5.4697	CY	CY	F
A0A1D3JQZ5	Leu/Ile/Val-binding protein homolog 1 OS=Pseudomonas veronii 1YdBTEX2	Y	Y	Y	40087	5.874	PR	PR	E
A0A1D3JR00	Bifunctional protein PutA OS=Pseudomonas veronii 1YdBTEX2	Y	Y		142427	5.6968	CY	CY	CE
A0A1D3JR12	Biotin carboxylase OS=Pseudomonas veronii 1YdBTEX2	Y	Y	Y	49353	5.8945	CY	CY	IF
A0A1D3JR13	Phosphate starvation-inducible protein PsiF OS=Pseudomonas veronii 1YdBTEX2		Y		8341.13	9.65	PR	UK	S
A0A1D3JR14	Protein HflK OS=Pseudomonas veronii 1YdBTEX2	Y	Y	Y	43149	5.2178	IM	UK	O
A0A1D3JR17	Azurin OS=Pseudomonas veronii 1YdBTEX2	Y	Y	Y	15812	6.4834	PR	PR	C
A0A1D3JR22	30S ribosomal protein S18 OS=Pseudomonas veronii 1YdBTEX2	Y	Y		8911	10.9336	CY	CY	J
A0A1D3JR26	Alpha-2-macroglobulin OS=Pseudomonas veronii 1YdBTEX2	Y	Y	Y	178036	5.3042	OM	OM	PU
A0A1D3JR27	Ribonuclease R OS=Pseudomonas veronii 1YdBTEX2	Y	Y	Y	97499.63	8.69	CY	CY	JKO
A0A1D3JR37	Methyl-accepting chemotaxis protein CtpL OS=Pseudomonas veronii 1YdBTEX2	Y	Y	Y	69417	4.9614	IM	OM	NT(mx)
A0A1D3JR39	DUF4136 domain-containing protein OS=Pseudomonas veronii 1YdBTEX2	Y	Y	Y	20761	9.0337	PR	UK	S
A0A1D3JR45	Acetyl-CoA acetyltransferase OS=Pseudomonas veronii 1YdBTEX2	Y	Y	Y	45417.81	7.66	CY	CY	S
A0A1D3JR46	Aldehyde dehydrogenase family 7 member A1 homolog OS=Pseudomonas veronii 1YdBTEX2	Y			53453	5.3379	CY	CY	CE
A0A1D3JR49	Copper-exporting P-type ATPase A OS=Pseudomonas veronii 1YdBTEX2	Y	Y	Y	76667	6.5039	IM	IM	PC
A0A1D3JR62	Adenine deaminase OS=Pseudomonas veronii 1YdBTEX2	Y	Y	Y	35996	4.7417	CY	CY	FQ
A0A1D3JR63	Omega-amino acid--pyruvate aminotransferase OS=Pseudomonas veronii 1YdBTEX2	Y	Y	Y	48614	6.0015	CY	CY	EH(mx)

A0A1D3JR74	Zinc-type alcohol dehydrogenase-like protein OS=Pseudomonas veronii 1YdBTEX2			Y	36112	6.0015	CY	CY	CIE
A0A1D3JR75	[Ribosomal protein S18]-alanine N-acetyltransferase OS=Pseudomonas veronii 1YdBTEX2	Y			16922.48	5.19	CY	CY	K
A0A1D3JR76	Biotin carboxyl carrier protein of acetyl-CoA carboxylase OS=Pseudomonas veronii 1YdBTEX2	Y	Y	Y	16248	4.9189	PR	CY	CIE
A0A1D3JR85	TetR family transcriptional regulator OS=Pseudomonas veronii 1YdBTEX2	Y			24403	7.9907	EX	CY	K
A0A1D3JR88	TonB-dependent receptor OS=Pseudomonas veronii 1YdBTEX2		Y	Y	74763	5.4038	OM	OM	PH
A0A1D3JR94	Uncharacterized HTH-type transcriptional regulator PA4778 OS=Pseudomonas veronii 1YdBTEX2	Y	Y	Y	15095	7.2583	CY	CY	KT
A0A1D3JRA5	Fic/DOC family protein OS=Pseudomonas veronii 1YdBTEX2	Y			43394.47	6.9	CY	CY	KD
A0A1D3JRA8	Bifunctional purine biosynthesis protein PurH OS=Pseudomonas veronii 1YdBTEX2		Y		57264	6.1011	CY	CY	F
A0A1D3JRA9	Methyl-accepting chemotaxis protein PctA OS=Pseudomonas veronii 1YdBTEX2	Y			67759.56	5.14	IM	IM	NT
A0A1D3JRB0	AraC family transcriptional regulator OS=Pseudomonas veronii 1YdBTEX2	Y			30364.87	8.88	CY	UK	K
A0A1D3JRB1	Bacterioferritin OS=Pseudomonas veronii 1YdBTEX2	Y	Y	Y	19810	4.4414	CY	CY	PH
A0A1D3JRB2	3-oxopropanoate dehydrogenase OS=Pseudomonas veronii 1YdBTEX2	Y		Y	53395	5.7012	CY	CY	CE
A0A1D3JRB4	Porin OS=Pseudomonas veronii 1YdBTEX2	Y	Y	Y	48295	6.1582	EX	OM	S
A0A1D3JRB5	Biosynthetic arginine decarboxylase OS=Pseudomonas veronii 1YdBTEX2	Y	Y		71314.15	5.32	CY	CY	HE
A0A1D3JRB8	Uncharacterized protein OS=Pseudomonas veronii 1YdBTEX2	Y	Y	Y	39373	5.814	OM	UK	S
A0A1D3JRC4	HPr OS=Pseudomonas veronii 1YdBTEX2	Y			13827	3.8481	CY	CY	S
A0A1D3JRC8	3-ketoacyl-ACP reductase OS=Pseudomonas veronii 1YdBTEX2	Y		Y	47210	7.4121	PR	CY	IQ
A0A1D3JRD8	Monooxygenase OS=Pseudomonas veronii 1YdBTEX2		Y	Y	11078	5.7876	CY	UK	C
A0A1D3JRE5	Short-chain dehydrogenase OS=Pseudomonas veronii 1YdBTEX2	Y	Y	Y	24432.75	7.3	CY	EX	IQ

A0A1D3JRF7	Phosphoribosylamine--glycine ligase OS=Pseudomonas veronii 1YdBTEX2	Y	Y	Y	45403	4.8223	CY	CY	FI
A0A1D3JRI0	Ribose-phosphate pyrophosphokinase OS=Pseudomonas veronii 1YdBTEX2	Y	Y	Y	33834	5.4551	CY	CY	FK
A0A1D3JRI3	Ribosome-binding ATPase YchF OS=Pseudomonas veronii 1YdBTEX2	Y			39761.49	4.9	CY	CY	JCO
A0A1D3JRJ4	Molybdopterin-synthase adenylyltransferase OS=Pseudomonas veronii 1YdBTEX2	Y			26470.95	5.66	UK	CY	H
A0A1D3JRL0	Acyltransferase OS=Pseudomonas veronii 1YdBTEX2	Y			34806	9.7822	IM	IM	I
A0A1D3JRL1	Peptidyl-prolyl cis-trans isomerase OS=Pseudomonas veronii 1YdBTEX2	Y	Y	Y	16934	4.2393	CY	UK	O
A0A1D3JRL2	30S ribosomal protein S20 OS=Pseudomonas veronii 1YdBTEX2		Y		10032	11.3628	CY	CY	J
A0A1D3JRL7	50S ribosomal protein L25 OS=Pseudomonas veronii 1YdBTEX2	Y	Y	Y	21203	5.8228	CY	CY	J
A0A1D3JRM3	Outer membrane protein assembly factor BamD OS=Pseudomonas veronii 1YdBTEX2	Y	Y		38551	5.2148	PR	OM	E
A0A1D3JRM8	Lipid A deacylase OS=Pseudomonas veronii 1YdBTEX2	Y	Y	Y	18801	5.313	PR	UK	M
A0A1D3JRN4	Chaperone protein ClpB OS=Pseudomonas veronii 1YdBTEX2	Y	Y	Y	95411	5.2017	CY	CY	O
A0A1D3JRP9	Catabolite repressor/activator OS=Pseudomonas veronii 1YdBTEX2	Y	Y		36325	6.75	UK	CY	KG
A0A1D3JRQ8	TPR repeat-containing protein PA4667 OS=Pseudomonas veronii 1YdBTEX2	Y	Y	Y	62481.09	4.9	UK	CY	MO(mx)
A0A1D3JRR2	Methyl-accepting chemotaxis protein OS=Pseudomonas veronii 1YdBTEX2	Y			72744	5.0962	IM	IM	NT(mx)
A0A1D3JRS0	Uncharacterized protein OS=Pseudomonas veronii 1YdBTEX2	Y	Y	Y	25611	7.5044	OM	UK	S
A0A1D3JRS4	Periplasmic dipeptide transport protein OS=Pseudomonas veronii 1YdBTEX2		Y	Y	58644	6.1831	PR	PR	E
A0A1D3JRS8	Periplasmic dipeptide transport protein OS=Pseudomonas veronii 1YdBTEX2	Y	Y	Y	60156	6.8218	PR	PR	E

A0A1D3JRT8	30S ribosomal protein S9 OS=Pseudomonas veronii 1YdBTEX2	Y	Y	Y	14606	11.7539	CY	CY	J
A0A1D3JRT9	Sulfate adenylyltransferase subunit 2 OS=Pseudomonas veronii 1YdBTEX2	Y	Y	Y	35198	5.9458	CY	CY	EH
A0A1D3JRU4	Ubiquinol-cytochrome c reductase iron-sulfur subunit OS=Pseudomonas veronii 1YdBTEX2	Y		Y	21090	8.2573	PR	IM	C
A0A1D3JRU5	Cytochrome c1 OS=Pseudomonas veronii 1YdBTEX2	Y		Y	28913	7.2495	IM	UK	C
A0A1D3JRW1	Porin OS=Pseudomonas veronii 1YdBTEX2		Y	Y	50660	6.1758	OM	OM	S
A0A1D3JRW6	Cell shape-determining protein MreB OS=Pseudomonas veronii 1YdBTEX2	Y	Y	Y	36980	5.127	CY	CY	DO
A0A1D3JRW8	Cytochrome D ubiquinol oxidase subunit III OS=Pseudomonas veronii 1YdBTEX2	Y	Y	Y	16331	6.2227	IM	UK	S
A0A1D3JRX0	Nucleotide-binding protein PVE_R1G0955 OS=Pseudomonas veronii 1YdBTEX2	Y	Y	Y	32052.75	6	CY	CY	H
A0A1D3JRX2	Aspartyl/glutamyl-tRNA(Asn/Gln) amidotransferase subunit B OS=Pseudomonas veronii 1YdBTEX2	Y	Y	Y	52842	4.8882	CY	CY	J
A0A1D3JRX5	Cytochrome b OS=Pseudomonas veronii 1YdBTEX2		Y		45982	8.3071	IM	IM	C
A0A1D3JRX9	Metalloprotease TldD homolog OS=Pseudomonas veronii 1YdBTEX2		Y		50228	5.1299	CY	CY	S
A0A1D3JRZ9	Organic solvent ABC transporter substrate-binding protein OS=Pseudomonas veronii 1YdBTEX2	Y	Y	Y	16378	5.6792	IM	UK	Q
A0A1D3JS01	Histidinol dehydrogenase OS=Pseudomonas veronii 1YdBTEX2	Y	Y		47356	4.9453	CY	CY	E
A0A1D3JS08	Probable malate:quinone oxidoreductase OS=Pseudomonas veronii 1YdBTEX2	Y			54634.09	6.88	PR	CY	E
A0A1D3JS14	50S ribosomal protein L13 OS=Pseudomonas veronii 1YdBTEX2	Y	Y	Y	15708	10.0737	CY	CY	J
A0A1D3JS15	Uncharacterized protein OS=Pseudomonas veronii 1YdBTEX2	Y	Y	Y	66959	5.2368	EX	OM	X
A0A1D3JS16	Lipopolysaccharide export system protein LptA OS=Pseudomonas veronii 1YdBTEX2	Y			16388.43	8.11	OM	UK	S
A0A1D3JS21	UPF0102 protein PVE_R1G1005 OS=Pseudomonas veronii 1YdBTEX2	Y			13982.23	7.79	CY	UK	L

A0A1D3JS27	UDP-N-acetylmuramoylalanine--D-glutamate ligase OS=Pseudomonas veronii 1YdBTEX2	Y	Y	Y	47515.42	5.64	CY	CY	MHJ
A0A1D3JS30	Uncharacterized protein PA3922 OS=Pseudomonas veronii 1YdBTEX2	Y	Y	Y	50686	9.208	PR	UK	S
A0A1D3JS31	Cell envelope biogenesis protein OmpA OS=Pseudomonas veronii 1YdBTEX2	Y			26804	9.5552	EX	ML (UK)	MN
A0A1D3JS34	Glutamyl-tRNA(Gln) amidotransferase subunit A OS=Pseudomonas veronii 1YdBTEX2	Y			51664.17	6.4	CY	CY	JD
A0A1D3JS35	UDP-N-acetylmuramate--L-alanine ligase OS=Pseudomonas veronii 1YdBTEX2	Y	Y	Y	52630	5.7817	CY	CY	MHJ
A0A1D3JS37	Porin OS=Pseudomonas veronii 1YdBTEX2	Y		Y	48337	5.2734	OM	OM	S
A0A1D3JS38	Ribosomal RNA small subunit methyltransferase H OS=Pseudomonas veronii 1YdBTEX2	Y			34353.94	5.88	CY	CY	JK
A0A1D3JS41	Uncharacterized protein OS=Pseudomonas veronii 1YdBTEX2	Y	Y	Y	65041	7.3447	PR	UK	M
A0A1D3JS51	D-alanine--D-alanine ligase OS=Pseudomonas veronii 1YdBTEX2	Y	Y	Y	34282	4.6377	CY	CY	FI
A0A1D3JS52	Cell division protein FtsZ OS=Pseudomonas veronii 1YdBTEX2	Y	Y	Y	41931	4.9688	CY	CY	D
A0A1D3JS54	Cell division protein FtsA OS=Pseudomonas veronii 1YdBTEX2	Y	Y	Y	44848	4.9761	CY	CY	DN(mx)
A0A1D3JS77	Uncharacterized protein OS=Pseudomonas veronii 1YdBTEX2	Y	Y	Y	14949	5.9165	CY	UK	S
A0A1D3JS79	Uncharacterized protein OS=Pseudomonas veronii 1YdBTEX2			Y	6873	3.5977	CY	UK	S
A0A1D3JSA4	Phosphoheptose isomerase OS=Pseudomonas veronii 1YdBTEX2			Y	21279	4.5396	CY	CY	GM
A0A1D3JSA5	Homogentisate 1_2-dioxygenase OS=Pseudomonas veronii 1YdBTEX2		Y	Y	47211	5.4653	CY	UK	Q
A0A1D3JSB3	UDP-N-acetylglucosamine--N-acetylmuramyl-(pentapeptide) pyrophosphoryl-undecaprenol N-acetylglucosamine transferase OS=Pseudomonas veronii 1YdBTEX2	Y			37737.1	9.3	UK	ML (C, IM)	MG(mx)

A0A1D3JSC3	Fumarylacetoacetase OS=Pseudomonas veronii 1YdBTEX2	Y	Y		46311	5.1489	UK	CY	Q
A0A1D3JSC7	Leucine dehydrogenase OS=Pseudomonas veronii 1YdBTEX2			Y	35315	5.7598	CY	CY	EH
A0A1D3JSD1	Uncharacterized protein OS=Pseudomonas veronii 1YdBTEX2	Y	Y	Y	20438	9.8936	PR	UK	M
A0A1D3JSD2	Amino-acid ABC transporter-binding protein YhdW OS=Pseudomonas veronii 1YdBTEX2	Y	Y	Y	36602	6.6826	OM	PR	ET
A0A1D3JSD5	Ribosomal subunit interface protein OS=Pseudomonas veronii 1YdBTEX2	Y	Y	Y	15280	4.73	CY	CY	J
A0A1D3JSE9	Nucleoid-associated protein PFL_1060 OS=Pseudomonas veronii 1YdBTEX2			Y	26914	5.1782	CY	CY	S
A0A1D3JSF1	Probable cytosol aminopeptidase OS=Pseudomonas veronii 1YdBTEX2	Y	Y	Y	52329	7.9717	CY	CY	E
A0A1D3JSF6	Phospholipid-binding protein OS=Pseudomonas veronii 1YdBTEX2		Y		20407	9.2344	CY	CY	GM
A0A1D3JSF8	Uncharacterized ABC transporter ATP-binding protein YbhF OS=Pseudomonas veronii 1YdBTEX2	Y			63840	5.1255	UK	CY	V
A0A1D3JSG1	UDP-N-acetylmuramoyl-tripeptide--D-alanyl-D-alanine ligase OS=Pseudomonas veronii 1YdBTEX2	Y	Y	Y	47310	6.5405	CY	CY	MHJ
A0A1D3JSG8	Uncharacterized protein OS=Pseudomonas veronii 1YdBTEX2	Y			9743	4.5073	CY	UK	S
A0A1D3JSG9	ATPase OS=Pseudomonas veronii 1YdBTEX2	Y	Y	Y	51814	5.4756	PR	UK	S
A0A1D3JSH2	UDP-3-O-acyl-N-acetylglucosamine deacetylase OS=Pseudomonas veronii 1YdBTEX2	Y	Y	Y	33197	4.7241	CY	CY	M
A0A1D3JSJ1	IclR family transcriptional regulator OS=Pseudomonas veronii 1YdBTEX2	Y			27453	6.1802	CY	CY	K
A0A1D3JSJ6	Amidase domain-containing protein OS=Pseudomonas veronii 1YdBTEX2			Y	46914	6.7778	CY	CY	J
A0A1D3JSK7	Uncharacterized protein OS=Pseudomonas veronii 1YdBTEX2	Y	Y		9680	9.7808	CY	CY	L
A0A1D3JSM2	Uncharacterized amino-acid ABC transporter ATP-binding protein YhdZ OS=Pseudomonas veronii 1YdBTEX2	Y		Y	28829	7.2773	CY	ML (C, IM)	EV(mx)

A0A1D3JSM7	Chemotaxis protein CheY OS=Pseudomonas veronii 1YdBTEX2	Y	Y	Y	50725	4.9893	CY	CY	T
A0A1D3JSM9	Lon protease OS=Pseudomonas veronii 1YdBTEX2	Y			89425	5.9399	CY	CY	O
A0A1D3JSP6	Alkyl hydroperoxide reductase C OS=Pseudomonas veronii 1YdBTEX2	Y	Y	Y	21906	4.9438	CY	CY	O
A0A1D3JSP7	Glutamate/aspartate transport ATP-binding protein GltL OS=Pseudomonas veronii 1YdBTEX2	Y	Y	Y	26969	6.2637	CY	IM	EV(mx)
A0A1D3JSR6	Methionine--tRNA ligase OS=Pseudomonas veronii 1YdBTEX2	Y	Y	Y	75630.08	5.61	CY	CY	J
A0A1D3JSR8	Quinoprotein glucose dehydrogenase A OS=Pseudomonas veronii 1YdBTEX2		Y	Y	85911	5.9238	IM	PR	G
A0A1D3JSR9	Argininosuccinate synthase OS=Pseudomonas veronii 1YdBTEX2	Y	Y	Y	45399	5.3159	CY	CY	EJ
A0A1D3JSV2	NADPH dehydrogenase OS=Pseudomonas veronii	Y	Y	Y	40479	5.8887	OM	CY	C
A0A1D3JSV4	Ferredoxin--NADP reductase OS=Pseudomonas veronii 1YdBTEX2		Y	Y	29756	5.9092	CY	CY	C
A0A1D3JSV7	ABC transporter periplasmic-binding protein YdcS OS=Pseudomonas veronii 1YdBTEX2		Y	Y	41939	5.9854	PR	PR	EP
A0A1D3JSW3	Gamma-aminobutyraldehyde dehydrogenase OS=Pseudomonas veronii 1YdBTEX2	Y	Y		50933	5.4331	CY	CY	C
A0A1D3JSW4	Uncharacterized protein OS=Pseudomonas veronii 1YdBTEX2	Y	Y	Y	24939.95	5.73	PR	IM	TP
A0A1D3JSW6	Glutamate/aspartate periplasmic-binding protein OS=Pseudomonas veronii 1YdBTEX2	Y	Y	Y	33370	8.5986	PR	PR	ET
A0A1D3JSX5	Bacterioferritin OS=Pseudomonas veronii 1YdBTEX2		Y	Y	17901	4.6963	CY	CY	P
A0A1D3JSX6	Glycerol kinase OS=Pseudomonas veronii 1YdBTEX2	Y	Y	Y	55514	5.3013	CY	CY	CG
A0A1D3JSY3	Chromosome partitioning protein ParA OS=Pseudomonas veronii 1YdBTEX2	Y		Y	15198	9.479	OM	UK	M
A0A1D3JSY5	TetR family transcriptional regulator OS=Pseudomonas veronii 1YdBTEX2	Y	Y		26599	6.4043	CY	CY	K
A0A1D3JSZ2	Chemotaxis protein OS=Pseudomonas veronii 1YdBTEX2	Y		Y	58559.19	5.1	IM	IM	NT
A0A1D3JSZ5	Adenylate kinase OS=Pseudomonas veronii 1YdBTEX2	Y			23089	5.7056	PR	CY	F

A0A1D3JSZ7	Uncharacterized protein OS=Pseudomonas veronii 1YdBTEX2		Y		14314	8.6704	PR	UK	S
A0A1D3JT01	Glucans biosynthesis protein D OS=Pseudomonas veronii 1YdBTEX2	Y	Y	Y	60428	5.9121	PR	PR	P
A0A1D3JT07	Adenosylmethionine-8-amino-7-oxononanoate aminotransferase OS=Pseudomonas veronii 1YdBTEX2	Y	Y	Y	52137.2	5.77	CY	CY	EH
A0A1D3JT12	2_3_4_5-tetrahydropyridine-2_6-dicarboxylate N-succinyltransferase OS=Pseudomonas veronii 1YdBTEX2	Y		Y	36000	5.584	UK	CY	E
A0A1D3JT19	Lysine--tRNA ligase OS=Pseudomonas veronii 1YdBTEX2	Y		Y	56884	4.9556	CY	CY	JK
A0A1D3JT24	Methionine aminopeptidase OS=Pseudomonas veronii 1YdBTEX2		Y		28536	6.123	CY	CY	CE
A0A1D3JT28	Phosphoenolpyruvate carboxylase OS=Pseudomonas veronii 1YdBTEX2	Y		Y	98134	5.6558	CY	CY	C
A0A1D3JT33	Glutaredoxin OS=Pseudomonas veronii 1YdBTEX2	Y	Y	Y	12213	4.5	CY	UK	CO
A0A1D3JT34	Acetyl-coenzyme A carboxylase carboxyl transferase subunit alpha OS=Pseudomonas veronii 1YdBTEX2	Y	Y	Y	34840	5.7144	CY	CY	I
A0A1D3JT35	Skp-like protein OS=Pseudomonas veronii 1YdBTEX2	Y	Y	Y	18963	9.7324	UK	ML (UK)	M
A0A1D3JT43	30S ribosomal protein S2 OS=Pseudomonas veronii 1YdBTEX2	Y	Y	Y	27064	9.2915	CY	CY	J
A0A1D3JT44	2-dehydro-3-deoxyphosphooctonate aldolase OS=Pseudomonas veronii 1YdBTEX2	Y			30775.75	5.35	CY	CY	EM
A0A1D3JT50	dCTP deaminase OS=Pseudomonas veronii 1YdBTEX2	Y			21216	5.3496	CY	CY	F
A0A1D3JT61	Enolase OS=Pseudomonas veronii 1YdBTEX2	Y	Y	Y	45605	4.8003	CY	CY	G
A0A1D3JT64	Lysine/arginine/ornithine-binding periplasmic protein OS=Pseudomonas veronii 1YdBTEX2		Y	Y	27426	6.189	PR	PR	ET
A0A1D3JT65	Membrane protein OS=Pseudomonas veronii 1YdBTEX2	Y	Y	Y	28646	9.5332	OM	OM	MP
A0A1D3JT69	Protein RecA OS=Pseudomonas veronii 1YdBTEX2	Y	Y		37526	5.4507	CY	CY	LT
A0A1D3JT71	Outer membrane protein assembly factor BamA OS=Pseudomonas veronii 1YdBTEX2	Y	Y	Y	87288	5.1841	OM	OM	M
A0A1D3JT78	CTP synthase OS=Pseudomonas veronii 1YdBTEX2	Y	Y	Y	59604.54	5.73	UK	CY	F
A0A1D3JT86	High-affinity branched-chain amino acid transport ATP-binding protein BraF OS=Pseudomonas veronii 1YdBTEX2	Y			28157	7.3682	CY	ML (C, IM)	EVP
A0A1D3JT88	Membrane protein OS=Pseudomonas veronii 1YdBTEX2	Y	Y	Y	15847	9.4834	PR	OM	MI

A0A1D3JT92	Leucine-_ isoleucine-_ valine-_ threonine-_ and alanine-binding protein OS=Pseudomonas veronii 1YdBTEX2	Y	Y	Y	39740	6.4468	PR	PR	E
A0A1D3JTA1	3-oxoadipate CoA-transferase OS=Pseudomonas veronii 1YdBTEX2			Y	26988	5.27	CY	CY	IC
A0A1D3JTA7	Protocatechuate 3_4-dioxygenase subunit alpha OS=Pseudomonas veronii 1YdBTEX2		Y	Y	20539	4.8369	CY	CY	Q
A0A1D3JTC1	CSD domain-containing protein OS=Pseudomonas veronii 1YdBTEX2		Y		7721	7.7212	CY	CY	KL
A0A1D3JTE0	Toluene efflux pump outer membrane protein TtgC OS=Pseudomonas veronii 1YdBTEX2		Y	Y	52598	8.2998	OM	OM	MU
A0A1D3JTE5	Membrane protein OS=Pseudomonas veronii 1YdBTEX2	Y	Y		23927	5.439	UK	OM	M
A0A1D3JTF4	Elongation factor Ts OS=Pseudomonas veronii 1YdBTEX2	Y	Y	Y	30258	5.1196	CY	CY	J
A0A1D3JTF9	Protein DJ-1 homolog D OS=Pseudomonas veronii 1YdBTEX2	Y			20515	5.4668	CY	CY	KQF
A0A1D3JTG6	3-hydroxyacyl-[acyl-carrier-protein] dehydratase FabZ OS=Pseudomonas veronii 1YdBTEX2	Y	Y	Y	16690	7.8398	CY	CY	IQ
A0A1D3JTI3	3-oxoadipate enol-lactonase 1 OS=Pseudomonas veronii 1YdBTEX2	Y			28351.37	5.15	CY	CY	EI
A0A1D3JTJ5	Toluene efflux pump periplasmic linker protein TtgA OS=Pseudomonas veronii 1YdBTEX2	Y	Y		38820.31	6.75	PR	IM	MVP
A0A1D3JTK4	RNA polymerase sigma factor OS=Pseudomonas veronii 1YdBTEX2	Y	Y	Y	22237	5.1504	CY	CY	K
A0A1D3JTK8	LemA family lipoprotein OS=Pseudomonas veronii 1YdBTEX2	Y	Y	Y	22496	8.5488	OM	UK	S
A0A1D3JTL2	Uncharacterized protein OS=Pseudomonas veronii 1YdBTEX2	Y	Y		40447	4.9702	OM	OM	M
A0A1D3JTM0	Sigma factor AlgU negative regulatory protein OS=Pseudomonas veronii 1YdBTEX2	Y			20867	5.3101	CY	IM	T
A0A1D3JTP0	Chemotaxis protein OS=Pseudomonas veronii 1YdBTEX2	Y		Y	57712	5.41	IM	ML (IM, OM	NT
A0A1D3JTP5	Efflux pump membrane transporter OS=Pseudomonas veronii 1YdBTEX2	Y		Y	112782.2	6.13	IM	IM	VU(mx)
A0A1D3JTR0	4-hydroxy-tetrahydrodipicolinate synthase OS=Pseudomonas veronii 1YdBTEX2	Y			31186	5.3218	CY	CY	EM

A0A1D3JTS7	Periplasmic serine endoprotease DegP-like OS=Pseudomonas veronii 1YdBTEX2	Y			50701	5.8989	PR	PR	O
A0A1D3JTT2	Cache domain containing protein OS=Pseudomonas veronii 1YdBTEX2	Y			28673.77	8.3	PR	UK	T
A0A1D3JTT4	Cysteine synthase OS=Pseudomonas veronii 1YdBTEX2	Y	Y	Y	34233	5.9839	CY	CY	EK
A0A1D3JTT6	Uncharacterized protein OS=Pseudomonas veronii 1YdBTEX2	Y			6481.26	4.81	UK	UK	S
A0A1D3JTU6	DNA topoisomerase 1 OS=Pseudomonas veronii 1YdBTEX2	Y	Y	Y	97213.29	8.5	CY	CY	LK
A0A1D3JTU9	Sulfur carrier protein TusA OS=Pseudomonas veronii 1YdBTEX2	Y			9333	4.9277	CY	CY	O
A0A1D3JTV5	Universal stress protein OS=Pseudomonas veronii 1YdBTEX2	Y	Y	Y	15787	6.2446	CY	UK	T
A0A1D3JTV9	Fatty acid oxidation complex subunit alpha OS=Pseudomonas veronii 1YdBTEX2	Y	Y	Y	76867.26	5.86	CY	CY	I
A0A1D3JTW0	Glyceraldehyde-3-phosphate dehydrogenase-like protein OS=Pseudomonas veronii 1YdBTEX2	Y	Y	Y	53122	8.4946	CY	CY	E
A0A1D3JTW1	Glycerophosphoryl diester phosphodiesterase OS=Pseudomonas veronii 1YdBTEX2	Y			26898.75	6.32	CY	CY	C
A0A1D3JTX7	Porin OS=Pseudomonas veronii 1YdBTEX2	Y			43318.6	6.22	EX	OM	S
A0A1D3JTY1	ATPase AAA OS=Pseudomonas veronii 1YdBTEX2	Y			32138	5.7964	CY	CY	LO
A0A1D3JTY9	Sigma factor AlgU regulatory protein MucB OS=Pseudomonas veronii 1YdBTEX2		Y		33101.87	5.53	UK	PR	T
A0A1D3JTZ0	Lytic transglycosylase OS=Pseudomonas veronii 1YdBTEX2	Y			71127.14	9.4	OM	PR	MK
A0A1D3JTZ7	Lipoprotein OS=Pseudomonas veronii 1YdBTEX2	Y			12878.43	8.89	PR	UK	M
A0A1D3JTZ9	Phosphoribosylaminoimidazole-succinocarboxamide synthase OS=Pseudomonas veronii 1YdBTEX2	Y	Y	Y	26905	5.4976	CY	CY	F
A0A1D3JU04	VacJ family lipoprotein OS=Pseudomonas veronii 1YdBTEX2	Y	Y		25711	5.1812	EX	OM	M
A0A1D3JU12	Diguanylate cyclase OS=Pseudomonas veronii 1YdBTEX2	Y	Y	Y	56132	5.707	PR	PR	E
A0A1D3JU16	LysR family transcriptional regulator OS=Pseudomonas veronii 1YdBTEX2			Y	35800	6.6475	CY	CY	K
A0A1D3JU17	Transaldolase OS=Pseudomonas veronii 1YdBTEX2	Y		Y	33672	5.4829	IM	CY	G
A0A1D3JU31	CSD domain-containing protein OS=Pseudomonas veronii 1YdBTEX2	Y	Y	Y	7782	7.6494	CY	CY	KL

A0A1D3JU39	Enoyl-[acyl-carrier-protein] reductase [NADH] OS=Pseudomonas veronii 1YdBTEX2	Y	Y		43068	4.9819	CY	CY	I
A0A1D3JU43	Uncharacterized protein C11D3.03c OS=Pseudomonas veronii 1YdBTEX2	Y	Y		32034	5.6763	CY	CY	P
A0A1D3JU50	Lipoprotein OS=Pseudomonas veronii 1YdBTEX2	Y	Y		9938	9.0059	PR	UK	M
A0A1D3JU53	Bifunctional chorismate mutase/prephenate dehydratase OS=Pseudomonas veronii 1YdBTEX2	Y	Y	Y	40336	6.1348	CY	CY	E
A0A1D3JU60	Methylthioribose-1-phosphate isomerase OS=Pseudomonas veronii 1YdBTEX2			Y	38489	5.3247	CY	CY	JG
A0A1D3JU80	Aconitate hydratase OS=Pseudomonas veronii 1YdBTEX2	Y	Y		99089	5.4712	CY	CY	CE
A0A1D3JU98	Hypothetical secreted protein OS=Pseudomonas veronii 1YdBTEX2	Y	Y	Y	8821	8.7642	OM	OM	S
A0A1D3JUA2	Protease HtpX OS=Pseudomonas veronii 1YdBTEX2	Y	Y	Y	32495.92	8.73	IM	IM	OM(mx)
A0A1D3JUB3	30S ribosomal protein S1 OS=Pseudomonas veronii 1YdBTEX2	Y	Y	Y	62410	4.6509	CY	CY	JK(mx)
A0A1D3JUB5	Elongation factor P OS=Pseudomonas veronii 1YdBTEX2	Y	Y	Y	21218	4.5322	CY	CY	J
A0A1D3JUB6	DNA gyrase subunit A OS=Pseudomonas veronii 1YdBTEX2	Y	Y	Y	97806	5.13	CY	CY	L
A0A1D3JUC7	Phospho-2-dehydro-3-deoxyheptonate aldolase OS=Pseudomonas veronii 1YdBTEX2	Y			49828.96	5.79	OM	CY	E
A0A1D3JUD1	Molybdenum cofactor biosynthesis protein B OS=Pseudomonas veronii 1YdBTEX2	Y	Y	Y	19184	5.4141	CY	CY	H
A0A1D3JUF4	Hypothetical secreted protein OS=Pseudomonas veronii 1YdBTEX2	Y	Y	Y	8117	5.0054	PR	UK	M
A0A1D3JUH6	Metalloprotease OS=Pseudomonas veronii 1YdBTEX2	Y			53470.4	6.11	EX	EX	PQ(mx)
A0A1D3JUH9	Chemotaxis sensory transducer OS=Pseudomonas veronii 1YdBTEX2	Y			72814	5.6177	UK	IM	MN(mx)
A0A1D3JUJ4	LysR family transcriptional regulator OS=Pseudomonas veronii 1YdBTEX2	Y	Y		31658.2	5.67	CY	CY	K
A0A1D3JUJ8	Methyl-accepting chemotaxis protein OS=Pseudomonas veronii 1YdBTEX2	Y	Y	Y	73016.82	5.46	IM	ᴧL (IM, OM	NT
A0A1D3JUN6	Neutral metalloproteinase OS=Pseudomonas veronii 1YdBTEX2		Y		38866	6.5669	CY	EX	EO

A0A1D3JUP1	tRNA pseudouridine synthase A OS=Pseudomonas veronii 1YdBTEX2	Y			31786.14	8.9	CY	CY	J
A0A1D3JUP9	Uncharacterized protein YafE OS=Pseudomonas veronii 1YdBTEX2		Y		27994	5.8462	UK	CY	QH
A0A1D3JUR6	Peptidoglycan-binding protein OS=Pseudomonas veronii 1YdBTEX2	Y			87335.58	4.47	IM	UK	NU
A0A1D3JUR8	Translational regulator CsrA OS=Pseudomonas veronii 1YdBTEX2	Y	Y	Y	6962.78	6.53	UK	UK	T
A0A1D3JUR9	Conserved hypothetical secreted protein OS=Pseudomonas veronii 1YdBTEX2		Y		8475.25	9.1	PR	PR	S
A0A1D3JUS2	Membrane protein OS=Pseudomonas veronii 1YdBTEX2	Y			11267	4.5	CY	UK	T
A0A1D3JUS8	Glutaminase-asparaginase OS=Pseudomonas veronii 1YdBTEX2	Y	Y	Y	38653	8.7612	UK	PR	EJ
A0A1D3JUT5	50S ribosomal protein L20 OS=Pseudomonas veronii 1YdBTEX2	Y	Y	Y	13305	12	CY	CY	J
A0A1D3JUT7	Uncharacterized protein OS=Pseudomonas veronii 1YdBTEX2	Y		Y	11264	8.0068	PR	UK	S
A0A1D3JUT9	LacI family transcriptional regulator OS=Pseudomonas veronii 1YdBTEX2	Y			33220	9.3164	CY	CY	G
A0A1D3JUU2	CSD domain-containing protein OS=Pseudomonas veronii 1YdBTEX2	Y			7651	7.708	CY	CY	KL
A0A1D3JUV1	Threonine--tRNA ligase OS=Pseudomonas veronii 1YdBTEX2	Y	Y	Y	72424	5.6997	CY	CY	J
A0A1D3JUW4	Aminotransferase OS=Pseudomonas veronii 1YdBTEX2	Y		Y	42929	6	CY	CY	EK
A0A1D3JUY1	Ftsk_gamma domain-containing protein OS=Pseudomonas veronii 1YdBTEX2	Y			38600.73	4.64	CY	ML (C, IM)	DJ
A0A1D3JUY3	Aspartate-semialdehyde dehydrogenase OS=Pseudomonas veronii 1YdBTEX2	Y	Y	Y	40530	5.2983	UK	CY	E
A0A1D3JV13	Uncharacterized protein OS=Pseudomonas veronii 1YdBTEX2	Y	Y	Y	63142.31	4.93	CY	UK	S
A0A1D3JV15	Uncharacterized protein OS=Pseudomonas veronii 1YdBTEX2			Y	11166	5.1064	UK	CY	S
A0A1D3JV23	Protein LkcJ OS=Pseudomonas veronii 1YdBTEX2	Y			58533.29	6.5	CY	CY	VH(mx)

A0A1D3JV29	Phenylalanine--tRNA ligase beta subunit OS=Pseudomonas veronii 1YdBTEX2	Y			86514.11	5.28	UK	UK	J
A0A1D3JV34	3-isopropylmalate dehydratase large subunit OS=Pseudomonas veronii 1YdBTEX2	Y	Y	Y	50696	5.5444	CY	CY	EC
A0A1D3JV37	Uncharacterized protein OS=Pseudomonas veronii 1YdBTEX2	Y			24180.41	9.77	CY	CY	S
A0A1D3JV41	TonB-denpendent receptor OS=Pseudomonas veronii 1YdBTEX2	Y	Y	Y	85753	5.25	OM	OM	PH
A0A1D3JV48	Acetyl-coenzyme A carboxylase carboxyl transferase subunit beta OS=Pseudomonas veronii 1YdBTEX2	Y			33382	8.2705	CY	CY	I
A0A1D3JV62	Uncharacterized protein OS=Pseudomonas veronii 1YdBTEX2	Y			36563.39	6.25	CY	UK	S
A0A1D3JV63	Uncharacterized protein OS=Pseudomonas veronii 1YdBTEX2	Y			54123.76	7.56	CY	CY	S
A0A1D3JV75	Dihydrolipoyl dehydrogenase OS=Pseudomonas veronii 1YdBTEX2		Y	Y	48777	6.4336	UK	CY	C
A0A1D3JV78	Translation initiation factor IF-3 OS=Pseudomonas veronii 1YdBTEX2	Y			20066	9.9023	CY	CY	J
A0A1D3JV86	2-oxoisovalerate dehydrogenase subunit alpha OS=Pseudomonas veronii 1YdBTEX2	Y	Y	Y	45043	5.8594	CY	CY	IC
A0A1D3JV89	Branched-chain amino acid ABC transporter substrate-binding protein OS=Pseudomonas veronii 1YdBTEX2	Y			40195.48	5.86	PR	CY	E
A0A1D3JV93	Phosphate import ATP-binding protein PstB OS=Pseudomonas veronii 1YdBTEX2	Y			28182.4	8.29	CY	IM	PE(mx)
A0A1D3JVA1	Uncharacterized protein OS=Pseudomonas veronii 1YdBTEX2		Y	Y	23214	4.9878	OM	EX	FT
A0A1D3JVA7	Dihydrolipoamide acetyltransferase component of pyruvate dehydrogenase complex OS=Pseudomonas veronii 1YdBTEX2	Y	Y	Y	45000	5.8945	CY	CY	CI
A0A1D3JVA8	Membrane protein OS=Pseudomonas veronii 1YdBTEX2		Y	Y	42373	4.6743	OM	OM	M
A0A1D3JVA9	GntR family transcriptional regulator OS=Pseudomonas veronii 1YdBTEX2	Y			28734.7	6.22	CY	CY	K

A0A1D3JVB2	2-oxoisovalerate dehydrogenase subunit beta OS=Pseudomonas veronii 1YdBTEX2	Y		Y	38408	4.96	CY	CY	CGH
A0A1D3JVB3	Amino acid ABC transporter substrate-binding protein OS=Pseudomonas veronii 1YdBTEX2	Y		Y	32896	4.7988	PR	PR	ET
A0A1D3JVC4	Hcp1 family type VI secretion system effector OS=Pseudomonas veronii 1YdBTEX2	Y		Y	10018	8.5898	CY	EX	X
A0A1D3JVC5	Membrane protein OS=Pseudomonas veronii 1YdBTEX2	Y	Y	Y	34907	5.7832	OM	UK	DZ(mx)
A0A1D3JVD0	Cytochrome C OS=Pseudomonas veronii 1YdBTEX2		Y	Y	16392	5.1475	PR	UK	CT
A0A1D3JVD1	Quinoprotein ethanol dehydrogenase OS=Pseudomonas veronii 1YdBTEX2		Y	Y	69501	6.7207	OM	PR	CH
A0A1D3JVD3	Aldehyde dehydrogenase OS=Pseudomonas veronii 1YdBTEX2	Y	Y	Y	55001	5.3672	CY	CY	CK
A0A1D3JVD5	Sulfur oxidation protein SoxZ OS=Pseudomonas veronii 1YdBTEX2			Y	27640	5.0669	PR	CY	S
A0A1D3JVF7	Porin-like protein GalP OS=Pseudomonas veronii 1YdBTEX2	Y	Y	Y	42987.21	6.17	OM	OM	S
A0A1D3JVI5	Branched-chain-amino-acid aminotransferase OS=Pseudomonas veronii 1YdBTEX2	Y	Y	Y	36830	5.8887	CY	CY	E
A0A1D3JVK4	Quinoprotein ethanol dehydrogenase OS=Pseudomonas veronii 1YdBTEX2		Y	Y	64522	8.0405	PR	PR	GC
A0A1D3JVL2	ATP-dependent Clp protease proteolytic subunit OS=Pseudomonas veronii 1YdBTEX2	Y	Y	Y	23420	5.2485	CY	CY	OUT
A0A1D3JVM1	Peptidylprolyl isomerase OS=Pseudomonas veronii 1YdBTEX2	Y	Y	Y	68710	5.0098	OM	IM	OM
A0A1D3JVM7	2_4-dienoyl-CoA reductase [NADPH] OS=Pseudomonas veronii 1YdBTEX2			Y	72979	6.6445	CY	CY	C
A0A1D3JVN9	ATP-dependent Clp protease ATP-binding subunit ClpX OS=Pseudomonas veronii 1YdBTEX2	Y	Y	Y	46937	4.9292	CY	CY	OF(mx)
A0A1D3JVP1	Lon protease OS=Pseudomonas veronii 1YdBTEX2	Y	Y	Y	88828.01	5.61	CY	CY	OT(mx)
A0A1D3JVP7	Chemotaxis protein OS=Pseudomonas veronii 1YdBTEX2	Y	Y	Y	58132	4.7593	IM	IM	NT(mx)
A0A1D3JVQ0	Gamma-carboxygeranoyl-CoA hydratase OS=Pseudomonas veronii 1YdBTEX2	Y	Y	Y	29401	5.644	CY	CY	IHE
A0A1D3JVQ9	Branched-chain amino acid ABC transporter substrate-binding protein OS=Pseudomonas veronii 1YdBTEX2		Y	Y	43111	5.9839	PR	UK	E

A0A1D3JVR7	Beta-lactamase OS=Pseudomonas veronii 1YdBTEX2		Y	Y	34183	5.1929	IM	UK	PQ
A0A1D3JVR8	Cysteine--tRNA ligase OS=Pseudomonas veronii 1YdBTEX2	Y			51619.61	5.48	CY	CY	J
A0A1D3JVS3	Biotin carboxylase OS=Pseudomonas veronii 1YdBTEX2	Y	Y	Y	68554	5.2808	CY	CY	IF(mx)
A0A1D3JVS5	Membrane protein OS=Pseudomonas veronii 1YdBTEX2		Y	Y	45848.11	8.5	OM	IM	MP
A0A1D3JVS6	Methylcrotonoyl-CoA carboxylase beta chain_ mitochondrial OS=Pseudomonas veronii 1YdBTEX2	Y	Y	Y	57377	6.4204	CY	CY	I
A0A1D3JVT4	Peptidyl-prolyl cis-trans isomerase OS=Pseudomonas veronii 1YdBTEX2		Y	Y	18212	5.5972	CY	CY	O
A0A1D3JVT8	Autotransporter assembly factor TamA OS=Pseudomonas veronii 1YdBTEX2	Y			61020	6.19	PR	OM	MU
A0A1D3JVU1	Major tail tube protein OS=Pseudomonas veronii 1YdBTEX2	Y	Y	Y	18080.1	5.49	CY	UK	S
A0A1D3JVU7	Glutamine--tRNA ligase OS=Pseudomonas veronii	Y	Y	Y	64514	5.8491	CY	CY	J
A0A1D3JVV0	NADH-quinone oxidoreductase OS=Pseudomonas veronii 1YdBTEX2	Y		Y	98237.33	5.4	CY	ML (C, IM)	C
A0A1D3JVV9	NADH-quinone oxidoreductase subunit C/D OS=Pseudomonas veronii 1YdBTEX2	Y	Y		67604	5.9502	CY	CY	C
A0A1D3JVW1	Trigger factor OS=Pseudomonas veronii 1YdBTEX2	Y	Y	Y	48288	4.5747	CY	CY	DO
A0A1D3JVX6	NADH-quinone oxidoreductase subunit F OS=Pseudomonas veronii 1YdBTEX2	Y		Y	48828	7.1118	CY	ML (CY, IM)	C
A0A1D3JVY0	Probable transcriptional regulatory protein PVE_R1G2279 OS=Pseudomonas veronii 1YdBTEX2	Y	Y	Y	25214.8	4.85	CY	CY	K
A0A1D3JVY4	NADH-quinone oxidoreductase subunit B OS=Pseudomonas veronii 1YdBTEX2	Y			25411.58	5.19	CY	IM	C
A0A1D3JVY8	NADH dehydrogenase I subunit E OS=Pseudomonas veronii 1YdBTEX2		Y	Y	18032	4.522	CY	CY	C
A0A1D3JVZ1	Uncharacterized protein ZMO1242 OS=Pseudomonas veronii 1YdBTEX2	Y	Y	Y	43321	6.0073	UK	CY	E
A0A1D3JW15	tRNA-cytidine(32) 2-sulfurtransferase OS=Pseudomonas veronii 1YdBTEX2	Y	Y		31093.94	6.9	CY	CY	FD(mx)
A0A1D3JW24	Uncharacterized protein OS=Pseudomonas veronii 1YdBTEX2		Y		11495	6.3267	EX	UK	X
A0A1D3JW27	Peroxiredoxin OS=Pseudomonas veronii 1YdBTEX2	Y	Y	Y	16768	5.0288	UK	PR	CO
A0A1D3JW33	Peroxidase OS=Pseudomonas veronii 1YdBTEX2			Y	31070	4.6787	CY	CY	P

A0A1D3JW46	ABC transporter substrate-binding protein OS=Pseudomonas veronii 1YdBTEX2	Y		Y	64816	5.8125	EX	OM	EP
A0A1D3JW48	Isovaleryl-CoA dehydrogenase_ mitochondrial OS=Pseudomonas veronii 1YdBTEX2	Y	Y	Y	42340	5.4829	CY	CY	I
A0A1D3JW51	Aldose 1-epimerase OS=Pseudomonas veronii 1YdBTEX2	Y			41454	7.0811	PR	PR	G
A0A1D3JW53	Isocitrase OS=Pseudomonas veronii 1YdBTEX2	Y	Y	Y	48389	5.2559	CY	CY	CG
A0A1D3JW69	Serine--tRNA ligase OS=Pseudomonas veronii 1YdBTEX2	Y			46786	5.0918	CY	CY	J
A0A1D3JW75	Disulfide-bond oxidoreductase YghU OS=Pseudomonas veronii 1YdBTEX2	Y	Y	Y	31018.84	5.9	CY	CY	OH
A0A1D3JW78	Glycos_trans_3N domain-containing protein OS=Pseudomonas veronii 1YdBTEX2	Y			37091.16	5.92	CY	CY	EF
A0A1D3JW79	General secretion pathway protein M OS=Pseudomonas veronii 1YdBTEX2	Y			18354.6	9.68	IM	IM	U
A0A1D3JW89	Aldehyde dehydrogenase OS=Pseudomonas veronii 1YdBTEX2	Y		Y	59606	6.1816	CY	CY	CE
A0A1D3JWA4	Arylesterase OS=Pseudomonas veronii 1YdBTEX2	Y	Y	Y	29815	5.061	CY	CY	IQ(mx)
A0A1D3JWA5	Protein-tyrosine-phosphatase OS=Pseudomonas veronii 1YdBTEX2			Y	17232	5.0625	CY	CY	TKQ
A0A1D3JWB7	Membrane protein OS=Pseudomonas veronii 1YdBTEX2	Y	Y		59739.63	5.27	OM	OM	KP
A0A1D3JWC1	Uncharacterized protein OS=Pseudomonas veronii 1YdBTEX2			Y	10743	6.3164	CY	UK	S
A0A1D3JWC4	Denitrification regulatory protein NirQ OS=Pseudomonas veronii 1YdBTEX2	Y			28964.83	5.8	CY	CY	HO(mx)
A0A1D3JWC5	Isocitrate dehydrogenase [NADP] OS=Pseudomonas veronii 1YdBTEX2	Y	Y	Y	45472	5.1226	CY	CY	CE
A0A1D3JWD4	AraC family transcriptional regulator OS=Pseudomonas veronii 1YdBTEX2	Y			27904	6.7881	CY	CY	KTG
A0A1D3JWE2	Siroheme decarboxylase OS=Pseudomonas veronii 1YdBTEX2	Y			18874.79	8.93	CY	CY	K
A0A1D3JWF2	DnrE protein OS=Pseudomonas veronii 1YdBTEX2	Y	Y	Y	25491.71	9.29	CY	CY	KT
A0A1D3JWI4	Methyl-accepting chemotaxis protein OS=Pseudomonas veronii 1YdBTEX2	Y	Y	Y	57991	4.9131	IM	IM	NT(mx)

A0A1D3JWJ9	L-lactate dehydrogenase OS=Pseudomonas veronii 1YdBTEX2			Y	42057	7.9834	CY	CY	CI(mx)
A0A1D3JWK9	Iditol 2-dehydrogenase OS=Pseudomonas veronii 1YdBTEX2	Y	Y	Y	41095.86	5.74	CY	CY	CE
A0A1D3JWL0	Transketolase OS=Pseudomonas veronii 1YdBTEX2		Y		74337	5.6221	CY	CY	GC
A0A1D3JWL4	Nitrite reductase OS=Pseudomonas veronii 1YdBTEX2	Y	Y	Y	61256	8.543	PR	PR	CO
A0A1D3JWM0	Molybdenum cofactor biosynthesis protein B OS=Pseudomonas veronii 1YdBTEX2			Y	19928	5.1167	CY	CY	H
A0A1D3JWM5	Protein NorD OS=Pseudomonas veronii 1YdBTEX2	Y			69732.04	7.67	PR	CY	PO(mx)
A0A1D3JWM7	D-beta-hydroxybutyrate dehydrogenase OS=Pseudomonas veronii 1YdBTEX2	Y			26539.77	5.41	PR	CY	IQ
A0A1D3JWN2	ABC transporter substrate-binding protein OS=Pseudomonas veronii 1YdBTEX2	Y			30872	6.0718	EX	OM	EP(mx)
A0A1D3JWQ7	Uncharacterized protein y4wM OS=Pseudomonas veronii 1YdBTEX2			Y	69217	5.8696	PR	ML (CY, PR)	E
A0A1D3JWQ8	Uncharacterized protein OS=Pseudomonas veronii 1YdBTEX2	Y		Y	35432.99	5.54	PR	UK	FG
A0A1D3JWR0	ABC transporter substrate-binding protein OS=Pseudomonas veronii 1YdBTEX2			Y	69737	5.4961	PR	PR	E
A0A1D3JWR4	Chromate reductase OS=Pseudomonas veronii 1YdBTEX2	Y	Y	Y	19761	7.3784	PR	CY	KI
A0A1D3JWR5	General secretion pathway protein GspG OS=Pseudomonas veronii 1YdBTEX2	Y	Y	Y	16298.32	5.28	IM	IM	NU
A0A1D3JWT3	Ribose/galactose/methyl galactoside import ATP-binding protein OS=Pseudomonas veronii 1YdBTEX2	Y			53406.75	6.44	UK	IM	EV(mx)
A0A1D3JWU0	Oxidoreductase OS=Pseudomonas veronii 1YdBTEX2		Y		18798	4.7051	CY	CY	EH
A0A1D3JWU1	Type VI secretion protein ImpG OS=Pseudomonas veronii 1YdBTEX2	Y	Y	Y	66999.47	6.12	CY	CY	S
A0A1D3JWU2	IclR family transcriptional regulator OS=Pseudomonas veronii 1YdBTEX2			Y	28428	5.8872	CY	CY	K
A0A1D3JWU4	Nitrous-oxide reductase OS=Pseudomonas veronii 1YdBTEX2	Y	Y	Y	70213	5.9575	PR	PR	CQP
A0A1D3JWU5	L-methionine gamma-lyase OS=Pseudomonas veronii 1YdBTEX2	Y	Y	Y	44506	6.6035	UK	CY	EP

A0A1D3JWV4	Nitrate reductase (quinone) OS=Pseudomonas veronii 1YdBTEX2	Y	Y	Y	140747	6.104	CY	ML (C, IM)	C
A0A1D3JWV7	Peptidylprolyl isomerase OS=Pseudomonas veronii 1YdBTEX2	Y	Y	Y	9865	5.4727	CY	CY	O
A0A1D3JWX1	3D-(3_5/4)-trihydroxycyclohexane-1_2-dione hydrolase OS=Pseudomonas veronii 1YdBTEX2	Y			69664.47	5.68	CY	CY	EH
A0A1D3JWX5	Acetoin:2_6-dichlorophenolindophenol oxidoreductase subunit alpha OS=Pseudomonas veronii 1YdBTEX2	Y		Y	34388	5.2427	CY	CY	IG
A0A1D3JWX6	Methyl-accepting chemotaxis protein OS=Pseudomonas veronii 1YdBTEX2	Y	Y	Y	57752	5.8198	IM	IM	NT(mx)
A0A1D3JWY3	SCP-2 family sterol carrier protein OS=Pseudomonas veronii 1YdBTEX2		Y		11059	3.9917	CY	UK	IQ
A0A1D3JWY7	Uncharacterized protein OS=Pseudomonas veronii 1YdBTEX2	Y	Y	Y	21052.62	6.71	CY	CY	E
A0A1D3JWZ7	Rhizopine-binding protein OS=Pseudomonas veronii 1YdBTEX2	Y	Y	Y	33598	8.8008	PR	PR	G
A0A1D3JX08	ABC transporter substrate-binding protein OS=Pseudomonas veronii 1YdBTEX2			Y	36811	5.6426	PR	PR	E
A0A1D3JX27	Acetoin:2_6-dichlorophenolindophenol oxidoreductase subunit beta OS=Pseudomonas veronii 1YdBTEX2			Y	36805	4.8296	CY	CY	CH
A0A1D3JX36	3-carboxymuconate cyclase OS=Pseudomonas veronii 1YdBTEX2			Y	42071	6.4204	PR	PR	GO
A0A1D3JX42	von Willebrand factor A OS=Pseudomonas veronii 1YdBTEX2	Y	Y	Y	40132.58	8.85	IM	IM	MH(mx)
A0A1D3JX46	Glutamate dehydrogenase OS=Pseudomonas veronii 1YdBTEX2	Y	Y	Y	181917	5.3818	CY	IM	E
A0A1D3JX49	Aconitate hydratase B OS=Pseudomonas veronii 1YdBTEX2	Y	Y	Y	94118	5.0757	CY	CY	CE
A0A1D3JX53	Pyruvate dehydrogenase [ubiquinone] OS=Pseudomonas veronii 1YdBTEX2	Y	Y	Y	62006.66	5.58	PR	ML (C, IM)	EH
A0A1D3JX55	Universal stress protein A OS=Pseudomonas veronii 1YdBTEX2	Y			31106.45	5.75	CY	CY	T
A0A1D3JXE2	Glycogen operon protein GlgX homolog OS=Pseudomonas veronii 1YdBTEX2	Y	Y	Y	81728	5.1533	CY	CY	G

A0A1D3JXE5	Chemotaxis protein OS=Pseudomonas veronii 1YdBTEX2	Y			56128	5.9619	IM	IM	NT(mx)
A0A1D3JXF1	Uncharacterized protein OS=Pseudomonas veronii 1YdBTEX2	Y			5537.88	9.51	UK	UK	S
A0A1D3JXF4	TonB-dependent receptor OS=Pseudomonas veronii 1YdBTEX2	Y	Y	Y	78594	4.8794	OM	OM	PH
A0A1D3JXG1	Biotin carboxylase OS=Pseudomonas veronii 1YdBTEX2	Y	Y	Y	61095	5.5371	UK	CY	IC
A0A1D3JXG4	5-oxoprolinase subunit A OS=Pseudomonas veronii 1YdBTEX2	Y			26823	6.4951	CY	UK	S
A0A1D3JXH4	HTH luxR-type domain-containing protein OS=Pseudomonas veronii 1YdBTEX2	Y			95930.06	6.81	CY	CY	KT
A0A1D3JXH6	Alpha/beta hydrolase OS=Pseudomonas veronii 1YdBTEX2			Y	41671	7.2	PR	UK	IC
A0A1D3JXK1	Methylhydantoinase OS=Pseudomonas veronii 1YdBTEX2	Y	Y	Y	59189	4.8721	EX	CY	EQ
A0A1D3JXK8	Uncharacterized protein OS=Pseudomonas veronii 1YdBTEX2	Y	Y	Y	56087	5.5811	CY	CY	E
A0A1D3JXL3	Uncharacterized protein OS=Pseudomonas veronii 1YdBTEX2	Y	Y	Y	12383	9.3237	PR	UK	M
A0A1D3JXM3	Alkyl hydroperoxide reductase C OS=Pseudomonas veronii 1YdBTEX2	Y	Y	Y	20438	4.7695	UK	CY	O
A0A1D3JXN6	4-hydroxyphenylpyruvate dioxygenase OS=Pseudomonas veronii 1YdBTEX2	Y		Y	40163	4.8076	CY	CY	E
A0A1D3JXQ1	Uncharacterized protein OS=Pseudomonas veronii 1YdBTEX2	Y	Y	Y	24073	9.5464	PR	UK	S
A0A1D3JXQ3	Uncharacterized protein OS=Pseudomonas veronii 1YdBTEX2	Y	Y	Y	17261	5.9414	CY	CY	X
A0A1D3JXQ4	UTP--glucose-1-phosphate uridylyltransferase OS=Pseudomonas veronii 1YdBTEX2	Y	Y	Y	30912	5.1519	CY	CY	JM
A0A1D3JXR9	Transcriptional regulator OS=Pseudomonas veronii 1YdBTEX2	Y	Y	Y	13321	10.084	CY	CY	I
A0A1D3JXS0	Probable acetyl-CoA acetyltransferase OS=Pseudomonas veronii 1YdBTEX2	Y	Y	Y	40823	6.394	CY	CY	I
A0A1D3JXT0	UDP-glucose 6-dehydrogenase OS=Pseudomonas veronii 1YdBTEX2	Y			51004	5.5913	UK	CY	M

A0A1D3JXT3	Uncharacterized protein OS=Pseudomonas veronii 1YdBTEX2		Y		29956	4.4385	OM	UK	S
A0A1D3JXT7	Acyl-CoA dehydrogenase OS=Pseudomonas veronii 1YdBTEX2	Y		Y	40594	5.0845	CY	CY	I
A0A1D3JXU0	Histidine kinase OS=Pseudomonas veronii 1YdBTEX2	Y	Y	Y	57737	5.0435	IM	IM	NT(mx)
A0A1D3JXW0	Uncharacterized protein OS=Pseudomonas veronii 1YdBTEX2			Y	21016	5.2676	CY	UK	S
A0A1D3JXW3	Bifunctional polymyxin resistance protein ArnA OS=Pseudomonas veronii 1YdBTEX2	Y		Y	74164	6.4746	UK	CY	GM
A0A1D3JXX3	Trehalose synthase OS=Pseudomonas veronii 1YdBTEX2	Y			75721.58	5.33	CY	CY	G
A0A1D3JXY5	Aldehyde-activating protein OS=Pseudomonas veronii 1YdBTEX2	Y	Y	Y	16947	6.0425	UK	PR	S
A0A1D3JY00	Acyl-CoA dehydrogenase OS=Pseudomonas veronii 1YdBTEX2	Y		Y	41589	5.4023	CY	CY	I
A0A1D3JY17	Chemotaxis protein OS=Pseudomonas veronii 1YdBTEX2	Y	Y	Y	58093	5.3848	IM	IM	NT(mx)
A0A1D3JY18	Uncharacterized protein OS=Pseudomonas veronii 1YdBTEX2	Y	Y	Y	40165	5.6353	IM	IM	I
A0A1D3JY20	Ribosome association toxin RatA OS=Pseudomonas veronii 1YdBTEX2			Y	16481	4.6128	CY	UK	I
A0A1D3JY28	Uncharacterized protein OS=Pseudomonas veronii 1YdBTEX2	Y	Y	Y	36406	6.3911	OM	UK	C
A0A1D3JY32	Uncharacterized protein OS=Pseudomonas veronii 1YdBTEX2		Y		18261	4.3037	CY	CY	K
A0A1D3JY35	NADP-dependent oxidoreductase OS=Pseudomonas veronii 1YdBTEX2	Y			36993	5.4272	CY	CY	C
A0A1D3JY37	Heat-shock protein OS=Pseudomonas veronii 1YdBTEX2	Y	Y		20428	6.3545	CY	CY	O
A0A1D3JY38	Short-chain dehydrogenase OS=Pseudomonas veronii 1YdBTEX2	Y			29457.86	9.15	UK	CY	IQ
A0A1D3JY39	Fe(3+)-pyochelin receptor OS=Pseudomonas veronii 1YdBTEX2		Y	Y	78087	5.6279	OM	OM	PH
A0A1D3JY42	GFO_IDH_MocA domain-containing protein OS=Pseudomonas veronii 1YdBTEX2	Y	Y	Y	37548	5.8887	CY	UK	T
A0A1D3JY45	60 kDa chaperonin OS=Pseudomonas veronii 1YdBTEX2	Y	Y	Y	56925.12	5.15	CY	CY	O

A0A1D3JY51	Phosphoribosyl transferase OS=Pseudomonas veronii 1YdBTEX2	Y	Y	Y	25309	5.1445	CY	UK	QF
A0A1D3JY52	Acetylornithine aminotransferase OS=Pseudomonas veronii 1YdBTEX2	Y	Y	Y	44912	6.3208	CY	CY	EH
A0A1D3JY58	Beta-Casp domain-containing protein OS=Pseudomonas veronii 1YdBTEX2	Y	Y	Y	51001	6.3765	UK	CY	JP
A0A1D3JY66	Probable enoyl-CoA hydratase OS=Pseudomonas veronii 1YdBTEX2	Y	Y	Y	27758	5.2939	CY	CY	IH(mx)
A0A1D3JY68	Esterase OS=Pseudomonas veronii 1YdBTEX2	Y	Y	Y	38026	5.2178	EX	CY	CI
A0A1D3JY71	Biofilm dispersion protein BdlA OS=Pseudomonas veronii 1YdBTEX2	Y		Y	49423	5.9678	CY	ML (UK)	NT(mx)
A0A1D3JY75	Hypothetical secreted protein OS=Pseudomonas veronii 1YdBTEX2	Y	Y	Y	8954	9.8804	PR	OM	S
A0A1D3JY85	Uncharacterized protein OS=Pseudomonas veronii 1YdBTEX2	Y	Y	Y	59879	9.7354	IM	IM	VE(mx)
A0A1D3JY92	Alcohol dehydrogenase OS=Pseudomonas veronii 1YdBTEX2	Y	Y		37431	5.3042	CY	CY	CP(mx)
A0A1D3JYA8	Hypothetical secreted protein OS=Pseudomonas veronii 1YdBTEX2	Y	Y	Y	9010	7.3184	PR	OM	S
A0A1D3JYB7	Universal stress protein OS=Pseudomonas veronii 1YdBTEX2	Y	Y	Y	33891	5.6001	UK	CY	T
A0A1D3JYC9	Phosphoglucomutase OS=Pseudomonas veronii 1YdBTEX2	Y	Y		58779	5.5239	CY	CY	GK
A0A1D3JYD7	Uncharacterized protein OS=Pseudomonas veronii 1YdBTEX2			Y	4724	3.3413	UK	UK	S
A0A1D3JYG5	Quercetin 2_3-dioxygenase PA1210 OS=Pseudomonas veronii 1YdBTEX2	Y		Y	25284	5.5825	CY	CY	OL
A0A1D3JYH3	Acetyltransferase PA5475 OS=Pseudomonas veronii 1YdBTEX2	Y			21135	6.0498	CY	CY	ME
A0A1D3JYH4	LysR family transcriptional regulator OS=Pseudomonas veronii 1YdBTEX2	Y			35145.22	6.7	CY	CY	K
A0A1D3JYJ0	2-hydroxymuconic semialdehyde dehydrogenase OS=Pseudomonas veronii 1YdBTEX2	Y			52872.45	5.84	CY	CY	C

A0A1D3JYJ6	2-hydroxyhexa-2_4-dienoate hydratase OS=Pseudomonas veronii 1YdBTEX2		Y	Y	28433.66	4.98	CY	UK	Q
A0A1D3JYN7	Phenol hydroxylase P1 protein OS=Pseudomonas veronii 1YdBTEX2			Y	37542	4.7241	CY	UK	C
A0A1D3JYP8	Aldehyde dehydrogenase PuuC OS=Pseudomonas veronii 1YdBTEX2	Y	Y	Y	53525	5.2046	CY	CY	CEK
A0A1D3JYP9	Spermidine/putrescine import ATP-binding protein PotA OS=Pseudomonas veronii 1YdBTEX2	Y	Y	Y	42409.75	6.15	CY	IM	PE
A0A1D3JYR3	3-phenylpropionate dioxygenase OS=Pseudomonas veronii 1YdBTEX2	Y			20583	7.3579	CY	CY	Q
A0A1D3JYR4	2-hydroxymuconate tautomerase OS=Pseudomonas veronii 1YdBTEX2		Y	Y	15693	5.1958	CY	CY	S
A0A1D3JYS7	Chemotaxis protein OS=Pseudomonas veronii 1YdBTEX2	Y	Y		58446.94	5.74	IM	IM	NT
A0A1D3JYS9	Gamma-glutamylputrescine synthetase PuuA OS=Pseudomonas veronii 1YdBTEX2	Y	Y	Y	50887	4.667	CY	CY	E
A0A1D3JYX3	C4-dicarboxylate-binding periplasmic protein OS=Pseudomonas veronii 1YdBTEX2	Y	Y		36837	8.8799	PR	PR	GQ
A0A1D3JYX6	50S ribosomal protein L21 OS=Pseudomonas veronii 1YdBTEX2	Y	Y	Y	18992.34	6.53	CY	CY	J
A0A1D3JYY5	Sugar ABC transporter substrate-binding protein OS=Pseudomonas veronii 1YdBTEX2	Y			33334	10.7227	UK	PR	PT
A0A1D3JYZ2	Uncharacterized protein OS=Pseudomonas veronii 1YdBTEX2	Y	Y	Y	13950.67	4.53	EX	UK	X
A0A1D3JYZ4	Phenol hydroxylase OS=Pseudomonas veronii 1YdBTEX2		Y	Y	13234	5.877	CY	UK	Q
A0A1D3JYZ7	Gamma-glutamyltranspeptidase OS=Pseudomonas veronii 1YdBTEX2	Y	Y	Y	60932	6.2212	OM	PR	E
A0A1D3JYZ8	TetR family transcriptional regulator OS=Pseudomonas veronii 1YdBTEX2			Y	21961	5.1724	CY	CY	K
A0A1D3JZ06	Maltokinase OS=Pseudomonas veronii 1YdBTEX2	Y	Y	Y	125167.5	5.69	CY	L (C, IM, OI	GM
A0A1D3JZ09	Uncharacterized protein OS=Pseudomonas veronii 1YdBTEX2	Y			8617.15	4.21	CY	CY	S
A0A1D3JZ13	Uncharacterized oxidoreductase YgfF OS=Pseudomonas veronii 1YdBTEX2	Y	Y	Y	26164.64	7.2	UK	CY	IQ

A0A1D3JZ21	Thiol peroxidase OS=Pseudomonas veronii 1YdBTEX2	Y	Y	Y	17643	4.3228	CY	PR	O
A0A1D3JZ23	Sugar ABC transporter substrate-binding protein OS=Pseudomonas veronii 1YdBTEX2	Y	Y	Y	48187	6.5244	PR	UK	G
A0A1D3JZ40	Methyltransferase OS=Pseudomonas veronii 1YdBTEX2		Y	Y	27870.26	6.3	CY	CY	QH(mx)
A0A1D3JZ43	C4-dicarboxylate ABC transporter OS=Pseudomonas veronii 1YdBTEX2			Y	33431	7.3594	OM	UK	GP(mx)
A0A1D3JZ60	Uncharacterized protein OS=Pseudomonas veronii 1YdBTEX2	Y	Y	Y	11471	5.2339	CY	UK	T
A0A1D3JZ66	Uncharacterized protein OS=Pseudomonas veronii 1YdBTEX2	Y			46511	4.8296	OM	UK	M
A0A1D3JZ78	Allergen V5/Tpx-1 family protein OS=Pseudomonas veronii 1YdBTEX2	Y			27915.8	5.56	PR	UK	O
A0A1D3JZ92	Uncharacterized protein OS=Pseudomonas veronii 1YdBTEX2			Y	15244	5.3745	CY	UK	X
A0A1D3JZA0	TonB-denpendent receptor OS=Pseudomonas veronii 1YdBTEX2		Y	Y	82573	5.1841	OM	OM	PH
A0A1D3JZE2	Ferripyoverdine receptor OS=Pseudomonas veronii 1YdBTEX2		Y	Y	88987	5.0303	OM	OM	PH
A0A1D3JZG0	Photosynthetic protein synthase I OS=Pseudomonas veronii 1YdBTEX2		Y		22064	6.8452	CY	UK	MP(mx)
A0A1D3JZG1	Type IV secretion protein DotU OS=Pseudomonas veronii 1YdBTEX2			Y	24916	5.0391	CY	UK	S
A0A1D3JZI1	2-oxoglutarate dioxygenase (ethylene-forming) OS=Pseudomonas veronii 1YdBTEX2			Y	37791	8.0024	CY	CY	CQ
A0A1D3JZI9	Urea ABC transporter ATP-binding protein OS=Pseudomonas veronii 1YdBTEX2	Y			25478.48	6.33	UK	ML (C, IM)	EV(mx)
A0A1D3JZJ4	Chemotaxis protein OS=Pseudomonas veronii 1YdBTEX2	Y	Y	Y	57552	5.1152	IM	IM	NTP
A0A1D3JZL5	6-phosphogluconate dehydrogenase YqeC OS=Pseudomonas veronii 1YdBTEX2	Y	Y	Y	35120	5.5854	OM	UK	GI
A0A1D3JZN3	Uncharacterized protein YcaC OS=Pseudomonas veronii 1YdBTEX2	Y	Y	Y	22549	4.6772	CY	UK	Q
A0A1D3JZN4	Uncharacterized protein OS=Pseudomonas veronii 1YdBTEX2	Y			86800.24	6.23	PR	CY	UTM

A0A1D3JZR0	Chemotaxis protein OS=Pseudomonas veronii 1YdBTEX2	Y	Y	Y	69464	4.9541	IM	IM	NTP
A0A1D3JZS8	2_3-dihydroxyphenylpropionate/2_3-dihydroxicinnamic acid 1_2-dioxygenase OS=Pseudomonas veronii 1YdBTEX2			Y	34222	5.6089	PR	UK	CQ
A0A1D3JZT1	Cell filamentation protein Fic OS=Pseudomonas veronii 1YdBTEX2			Y	26152	6.0645	CY	CY	DK
A0A1D3JZT7	Type IV secretion protein Rhs OS=Pseudomonas veronii 1YdBTEX2	Y	Y	Y	37504	9.145	EX	CY	M
A0A1D3JZV6	Mhp operon transcriptional activator OS=Pseudomonas veronii 1YdBTEX2	Y			29346	6.3618	CY	CY	KT
A0A1D3JZV9	Peptidase OS=Pseudomonas veronii 1YdBTEX2	Y			52542.15	7	PR	UK	EO
A0A1D3JZX4	Glucarate dehydratase OS=Pseudomonas veronii 1YdBTEX2			Y	49257	5.644	CY	CY	M
A0A1D3JZX8	DeoR faimly transcriptional regulator OS=Pseudomonas veronii 1YdBTEX2	Y			23735.65	6.44	UK	CY	QT(mx)
A0A1D3JZZ0	Methyl-accepting chemotaxis protein McpS OS=Pseudomonas veronii 1YdBTEX2	Y	Y	Y	68731	5.0845	IM	IM	NT(mx)
A0A1D3JZZ6	Alcohol dehydrogenase_ propanol-preferring OS=Pseudomonas veronii 1YdBTEX2	Y	Y	Y	35559	5.981	UK	CY	CP
A0A1D3K008	Type VI secretion system protein VgrGA OS=Pseudomonas veronii 1YdBTEX2	Y			75868	6.0498	CY	CY	T
A0A1D3K015	Tli4_C domain-containing protein OS=Pseudomonas veronii 1YdBTEX2			Y	46585	5.9561	PR	UK	S
A0A1D3K049	L-arabinose-binding periplasmic protein OS=Pseudomonas veronii 1YdBTEX2	Y	Y	Y	35506	7.1763	PR	PR	GK
A0A1D3K060	Succinate-semialdehyde dehydrogenase [NADP(+)] OS=Pseudomonas veronii 1YdBTEX2	Y	Y		49609	4.9966	CY	CY	CE(mx)
A0A1D3K076	Lysine/arginine/ornithine-binding periplasmic protein OS=Pseudomonas veronii 1YdBTEX2	Y		Y	28213	7.9233	PR	PR	ET
A0A1D3K077	3-oxoadipate CoA-transferase OS=Pseudomonas veronii 1YdBTEX2	Y	Y	Y	25043	5.1035	CY	CY	I
A0A1D3K079	Virulence factors putative positive transcription regulator BvgA OS=Pseudomonas veronii 1YdBTEX2	Y	Y		23017.24	8.86	CY	CY	KT
A0A1D3K096	DNA topoisomerase OS=Pseudomonas veronii 1YdBTEX2	Y			71115	9.854	CY	CY	LBK

A0A1D3K097	Uncharacterized protein OS=Pseudomonas veronii 1YdBTEX2	Y	Y		18757	6.3721	CY	CY	O
A0A1D3K0A2	3-oxoadipate CoA-transferase OS=Pseudomonas veronii 1YdBTEX2	Y	Y		23405	4.6172	CY	CY	IC
A0A1D3K0A7	Methyl-accepting chemotaxis protein OS=Pseudomonas veronii 1YdBTEX2	Y			76663	4.689	IM	IM	NT(mx)
A0A1D3K0C0	Methyl-accepting chemotaxis protein OS=Pseudomonas veronii 1YdBTEX2	Y			42326	4.9116	OM	IM	NT(mx)
A0A1D3K0C8	UPF0502 protein PVE_R1G3888 OS=Pseudomonas veronii 1YdBTEX2	Y			24947	5.3364	CY	CY	Q
A0A1D3K0D4	Methyl-accepting chemotaxis protein PA2652 OS=Pseudomonas veronii 1YdBTEX2		Y		59459	5.0845	IM	IM	NT
A0A1D3K0E9	TonB-dependent siderophore receptor OS=Pseudomonas veronii 1YdBTEX2	Y	Y	Y	78183	5.3218	OM	OM	PH
A0A1D3K0F0	Histidine kinase OS=Pseudomonas veronii 1YdBTEX2	Y	Y		16098.62	6.29	CY	CY	KT(mx)
A0A1D3K0G0	Delta-aminolevulinic acid dehydratase OS=Pseudomonas veronii 1YdBTEX2			Y	35410	5.4932	CY	CY	H
A0A1D3K0I8	Methyl-accepting chemotaxis protein OS=Pseudomonas veronii 1YdBTEX2	Y	Y	Y	73299.88	5.84	IM	IM	NT
A0A1D3K0K8	Methyl-accepting chemotaxis protein PA2652 OS=Pseudomonas veronii 1YdBTEX2	Y			59915	5.332	IM	IM	TQ(mx)
A0A1D3K0L5	Acetyl-CoA acetyltransferase OS=Pseudomonas veronii 1YdBTEX2	Y	Y	Y	40278	5.7979	CY	CY	IQC
A0A1D3K0M4	Tyrosyl-trna synthetase OS=Pseudomonas veronii 1YdBTEX2	Y	Y	Y	20536	9.0015	CY	UK	S
A0A1D3K0N5	Uncharacterized protein OS=Pseudomonas veronii 1YdBTEX2		Y		9883	9.3149	IM	UK	M
A0A1D3K0N9	Antibiotic biosynthesis monooxygenase OS=Pseudomonas veronii 1YdBTEX2			Y	13719	5.5518	CY	UK	S
A0A1D3K0P5	Glyoxalase OS=Pseudomonas veronii 1YdBTEX2	Y	Y	Y	16819	5.9268	CY	UK	E
A0A1D3K0P6	ABC transporter substrate-binding protein OS=Pseudomonas veronii 1YdBTEX2			Y	43826	6.5859	PR	PR	E

A0A1D3K0Q4	ABC transporter substrate-binding protein OS=Pseudomonas veronii 1YdBTEX2	Y	Y	Y	38151	5.9868	PR	PR	E
A0A1D3K0Q5	HAD family hydrolase OS=Pseudomonas veronii 1YdBTEX2	Y			24554.34	4.62	CY	CY	EI
A0A1D3K0Q8	Cro/Cl family transcriptional regulator OS=Pseudomonas veronii 1YdBTEX2		Y		12021.18	4.87	CY	UK	K
A0A1D3K0Q9	Hydroxycinnamoyl-CoA hydratase-lyase OS=Pseudomonas veronii 1YdBTEX2		Y	Y	31057.77	5.66	CY	CY	IH
A0A1D3K0S4	Response regulatory domain-containing protein OS=Pseudomonas veronii 1YdBTEX2			Y	16751	4.7622	CY	CY	TK
A0A1D3K0T2	Acyl-CoA dehydrogenase OS=Pseudomonas veronii 1YdBTEX2			Y	41492	5.4287	CY	CY	I
A0A1D3K0T9	Uncharacterized protein OS=Pseudomonas veronii 1YdBTEX2	Y	Y	Y	56103	6.2109	IM	UK	TN
A0A1D3K0V2	Recombination-associated protein RdgC OS=Pseudomonas veronii 1YdBTEX2	Y	Y	Y	34161	4.6919	CY	CY	L
A0A1D3K0W5	Cysteine protease OS=Pseudomonas veronii 1YdBTEX2	Y			24427.95	5.81	CY	UK	E
A0A1D3K0Y3	Inorganic pyrophosphatase OS=Pseudomonas veronii 1YdBTEX2	Y	Y	Y	19519	4.5981	CY	CY	C
A0A1D3K0Y4	Acetate/butyrate--CoA ligase AAE7_ peroxisomal OS=Pseudomonas veronii 1YdBTEX2	Y	Y	Y	59466.24	5.7	CY	CY	IQ
A0A1D3K0Y9	Quinone oxidoreductase PIG3 OS=Pseudomonas veronii 1YdBTEX2	Y		Y	33520	4.623	UK	ML (C, IM)	CP
A0A1D3K0Z6	Chemotaxis protein OS=Pseudomonas veronii 1YdBTEX2	Y	Y	Y	21133	4.9307	CY	IM	NT(mx)
A0A1D3K106	Alpha/beta hydrolase OS=Pseudomonas veronii 1YdBTEX2	Y			30522.96	6.67	CY	CY	IC
A0A1D3K109	DeoR family transcriptional regulator OS=Pseudomonas veronii 1YdBTEX2	Y	Y	Y	25657	5.6558	CY	UK	QE(mx)
A0A1D3K115	Pirin OS=Pseudomonas veronii 1YdBTEX2		Y		31361	5.3965	CY	UK	LO
A0A1D3K116	Succinate dehydrogenase iron-sulfur subunit OS=Pseudomonas veronii 1YdBTEX2	Y		Y	26104	7.6685	CY	IM	C
A0A1D3K117	Hypothetical secreted protein OS=Pseudomonas veronii 1YdBTEX2	Y	Y	Y	8351	5.6499	OM	UK	M
A0A1D3K119	3-hydroxydecanoyl-[acyl-carrier-protein] dehydratase OS=Pseudomonas veronii 1YdBTEX2	Y	Y	Y	18703	6.1362	CY	CY	I

A0A1D3K120	Dihydrolipoyllysine-residue succinyltransferase component of 2-oxoglutarate dehydrogenase complex OS=Pseudomonas veronii 1YdBTEX2	Y	Y	Y	42364	5.3101	CY	CY	C
A0A1D3K125	Dihydrolipoyl dehydrogenase OS=Pseudomonas veronii 1YdBTEX2	Y		Y	49961	6.2212	UK	CY	C
A0A1D3K127	Succinate dehydrogenase hydrophobic membrane anchor subunit OS=Pseudomonas veronii 1YdBTEX2	Y			13478.93	8.93	IM	IM	C
A0A1D3K130	Peptidyl-prolyl cis-trans isomerase OS=Pseudomonas veronii 1YdBTEX2	Y	Y	Y	25585	5.1987	PR	CY	O
A0A1D3K133	Succinate dehydrogenase flavoprotein subunit OS=Pseudomonas veronii 1YdBTEX2	Y	Y	Y	63567	5.729	CY	IM	CHN
A0A1D3K135	Citrate synthase OS=Pseudomonas veronii 1YdBTEX2	Y	Y	Y	47769	6.4966	IM	CY	C
A0A1D3K138	Glycine/betaine ABC transporter substrate-binding protein OS=Pseudomonas veronii 1YdBTEX2	Y			28244.73	7.1	UK	UK	EP
A0A1D3K160	Fumarate hydratase class II OS=Pseudomonas veronii 1YdBTEX2	Y	Y	Y	49123.11	5.67	CY	CY	CEF
A0A1D3K167	Peptidase M42 OS=Pseudomonas veronii 1YdBTEX2	Y	Y	Y	42559	4.9805	CY	CY	GE
A0A1D3K174	Peroxiredoxin OS=Pseudomonas veronii 1YdBTEX2			Y	15322	5.8081	CY	CY	OK
A0A1D3K181	Isochorismatase OS=Pseudomonas veronii 1YdBTEX2	Y	Y	Y	20683	6.4131	CY	CY	Q
A0A1D3K184	Succinate--CoA ligase [ADP-forming] subunit beta OS=Pseudomonas veronii 1YdBTEX2	Y	Y	Y	42946	5.9312	CY	CY	CF
A0A1D3K197	Chaperone protein HtpG OS=Pseudomonas veronii 1YdBTEX2	Y	Y	Y	71247	4.9834	CY	CY	OL
A0A1D3K1A4	Oxoglutarate dehydrogenase (succinyl-transferring) OS=Pseudomonas veronii 1YdBTEX2	Y	Y	Y	105962	6.0835	CY	CY	CI
A0A1D3K1B1	Chemotaxis protein OS=Pseudomonas veronii 1YdBTEX2	Y	Y		46935.2	4.99	IM	IM	NT
A0A1D3K1B4	Probable M18 family aminopeptidase 2 OS=Pseudomonas veronii 1YdBTEX2	Y	Y	Y	46877	5.5254	CY	CY	EG
A0A1D3K1B5	Cell division inhibitor MinD OS=Pseudomonas veronii 1YdBTEX2	Y			29228.35	5.4	CY	CY	DU
A0A1D3K1D3	3-oxoacyl-[acyl-carrier-protein] synthase 1 OS=Pseudomonas veronii 1YdBTEX2	Y	Y		43010	5.3452	CY	CY	IQ(mx)
A0A1D3K1E0	GTP cyclohydrolase 1 OS=Pseudomonas veronii 1YdBTEX2	Y		Y	20103	6.0996	CY	CY	H

A0A1D3K1E4	Succinate--CoA ligase [ADP-forming] subunit alpha OS=Pseudomonas veronii 1YdBTEX2	Y	Y	Y	29896	6.0762	CY	CY	C
A0A1D3K1E8	Exonuclease OS=Pseudomonas veronii 1YdBTEX2		Y		20672	7.6362	CY	CY	L
A0A1D3K1F0	Protein phosphatase CheZ OS=Pseudomonas veronii 1YdBTEX2	Y	Y	Y	29494	4.708	CY	CY	NT
A0A1D3K1G9	ABC transporter substrate-binding protein OS=Pseudomonas veronii 1YdBTEX2			Y	47156	5.0186	PR	PR	E
A0A1D3K1I0	ATPase AAA OS=Pseudomonas veronii 1YdBTEX2	Y	Y		55503	5.5708	CY	CY	KT(mx)
A0A1D3K1I8	Uncharacterized protein OS=Pseudomonas veronii 1YdBTEX2			Y	29458	4.4077	EX	CY	X
A0A1D3K1J2	Biotin carboxylase OS=Pseudomonas veronii 1YdBTEX2	Y			69142.72	6.15	UK	CY	IF
A0A1D3K1J5	Aromatic-amino-acid aminotransferase OS=Pseudomonas veronii 1YdBTEX2	Y	Y		42811	5.9956	CY	CY	EK
A0A1D3K1K2	Chemotaxis protein CheW OS=Pseudomonas veronii 1YdBTEX2	Y	Y	Y	17813	4.1499	CY	CY	NT
A0A1D3K1K6	Putative pterin-4-alpha-carbinolamine dehydratase OS=Pseudomonas veronii 1YdBTEX2	Y	Y	Y	13298	5.6353	CY	CY	H
A0A1D3K1L8	Metal-binding protein OS=Pseudomonas veronii 1YdBTEX2	Y			21796	3.7983	CY	CY	L
A0A1D3K1P0	Dehydrogenase OS=Pseudomonas veronii 1YdBTEX2	Y	Y	Y	26143	9.397	PR	PR	S
A0A1D3K1Q3	Cysteine synthase OS=Pseudomonas veronii 1YdBTEX2	Y	Y	Y	32416	5.0361	CY	CY	E
A0A1D3K1R8	Flagellin OS=Pseudomonas veronii 1YdBTEX2	Y	Y	Y	56065.34	4.87	EX	EX	N
A0A1D3K1T5	Chemotaxis protein OS=Pseudomonas veronii 1YdBTEX2	Y	Y	Y	56819	5.7246	CY	IM	NT(mx)
A0A1D3K1U5	Membrane protein OS=Pseudomonas veronii 1YdBTEX2		Y	Y	77979	5.0684	EX	OM	PH
A0A1D3K1V3	Cbb3-type cytochrome c oxidase subunit OS=Pseudomonas veronii 1YdBTEX2		Y	Y	35294	6.0688	IM	IM	C
A0A1D3K1V5	Extradiol dioxygenase OS=Pseudomonas veronii 1YdBTEX2		Y	Y	13312	4.83	CY	UK	E
A0A1D3K1V7	Peptidase S41 OS=Pseudomonas veronii 1YdBTEX2		Y	Y	22441	8.666	CY	UK	C
A0A1D3K1V9	NADPH-dependent curcumin reductase OS=Pseudomonas veronii 1YdBTEX2	Y		Y	36981	5.748	UK	CY	LC
A0A1D3K1W1	Peptidase S41 OS=Pseudomonas veronii 1YdBTEX2		Y	Y	22668	8.6616	IM	UK	C
A0A1D3K1Y7	Outer membrane porin F OS=Pseudomonas veronii 1YdBTEX2	Y	Y	Y	34658	5.7891	PR	OM	MNP

A0A1D3K1Y8	Cell division protein ZipA OS=Pseudomonas veronii 1YdBTEX2	Y	Y		31651	5.3467	IM	CY	X
A0A1D3K202	CmpX protein OS=Pseudomonas veronii 1YdBTEX2	Y			28967	6.1582	IM	IM	MT
A0A1D3K205	Alanine--tRNA ligase OS=Pseudomonas veronii 1YdBTEX2	Y			26846	5.6646	CY	CY	J
A0A1D3K207	Aconitate hydratase OS=Pseudomonas veronii 1YdBTEX2	Y	Y	Y	94030.25	5.48	CY	CY	CE
A0A1D3K218	Phosphoenolpyruvate synthase OS=Pseudomonas veronii 1YdBTEX2	Y	Y	Y	85861	4.8164	CY	CY	GT(mx)
A0A1D3K222	2-methylisocitrate lyase OS=Pseudomonas veronii 1YdBTEX2	Y	Y	Y	32137	5.5107	UK	CY	GC
A0A1D3K227	Cbb3-type cytochrome c oxidase subunit OS=Pseudomonas veronii 1YdBTEX2		Y		33804	5.9707	IM	IM	C
A0A1D3K228	Citrate synthase OS=Pseudomonas veronii 1YdBTEX2	Y	Y	Y	41627	5.9531	CY	CY	C
A0A1D3K241	Uncharacterized protein OS=Pseudomonas veronii 1YdBTEX2	Y	Y	Y	12797	4.437	CY	CY	S
A0A1D3K244	Acyltransferase Rv0859 OS=Pseudomonas veronii 1YdBTEX2	Y	Y	Y	42039.77	5.53	CY	CY	I
A0A1D3K251	Amidotransferase OS=Pseudomonas veronii 1YdBTEX2	Y	Y	Y	27763	5.2119	CY	CY	F
A0A1D3K261	Electron transfer flavoprotein subunit beta OS=Pseudomonas veronii 1YdBTEX2	Y	Y	Y	26298	9.4629	CY	UK	C
A0A1D3K275	Uncharacterized protein PA1579 OS=Pseudomonas veronii 1YdBTEX2	Y	Y	Y	22109	8.4082	PR	PR	I
A0A1D3K278	L-arabinose-binding periplasmic protein OS=Pseudomonas veronii 1YdBTEX2		Y		34896	6.0015	PR	PR	GK
A0A1D3K280	Electron transfer flavoprotein-ubiquinone oxidoreductase OS=Pseudomonas veronii 1YdBTEX2	Y	Y	Y	60641	5.4331	UK	CY	CH
A0A1D3K286	50S ribosomal protein L32 OS=Pseudomonas veronii 1YdBTEX2		Y		6738	10.4443	CY	CY	J
A0A1D3K290	Sugar kinase OS=Pseudomonas veronii 1YdBTEX2			Y	31397	4.9512	PR	CY	GH
A0A1D3K298	3-oxoacyl-[acyl-carrier-protein] reductase OS=Pseudomonas veronii 1YdBTEX2			Y	25556	5.8477	CY	CY	IQ
A0A1D3K2A7	Uncharacterized protein OS=Pseudomonas veronii 1YdBTEX2	Y			38950.02	5.35	EX	CY	P
A0A1D3K2A9	Peptidase S49 OS=Pseudomonas veronii 1YdBTEX2	Y	Y	Y	35993.71	6.93	OM	IM	OU

A0A1D3K2B2	4-hydroxy-4-methyl-2-oxoglutarate aldolase OS=Pseudomonas veronii 1YdBTEX2		Y	Y	17408	4.4604	CY	CY	H
A0A1D3K2B6	Flagellar brake protein YcgR OS=Pseudomonas veronii 1YdBTEX2	Y			28523.51	5.86	CY	CY	M
A0A1D3K2B9	Sensor histidine protein kinase/phosphatase PhoQ OS=Pseudomonas veronii 1YdBTEX2	Y	Y	Y	50445.66	6	IM	ML (C, IM)	TP
A0A1D3K2C5	Virulence transcriptional regulatory protein PhoP OS=Pseudomonas veronii 1YdBTEX2	Y			25547	5.2983	CY	CY	KT(mx)
A0A1D3K2C8	Flagellar basal-body rod protein FlgC OS=Pseudomonas veronii 1YdBTEX2	Y			15770	4.6318	EX	PR-flagellar	N
A0A1D3K2E2	Cyclic-di-GMP-binding biofilm dispersal mediator protein OS=Pseudomonas veronii 1YdBTEX2		Y		25193.02	5.43	UK	CY	IQ
A0A1D3K2F0	Acyl carrier protein OS=Pseudomonas veronii 1YdBTEX2	Y	Y	Y	8637	3.7515	CY	CY	IQ(mx)
A0A1D3K2F3	Electron transfer flavoprotein subunit alpha OS=Pseudomonas veronii 1YdBTEX2	Y	Y		31293	4.8838	CY	UK	C
A0A1D3K2F5	Low specificity L-threonine aldolase OS=Pseudomonas veronii 1YdBTEX2	Y			36113	6.1582	CY	CY	E
A0A1D3K2G0	Ribonuclease E OS=Pseudomonas veronii 1YdBTEX2	Y	Y		117039	4.7842	CY	CY	JG
A0A1D3K2G3	Arginine N-succinyltransferase subunit beta OS=Pseudomonas veronii 1YdBTEX2	Y		Y	37416	6.271	CY	CY	E
A0A1D3K2G8	N-succinylglutamate 5-semialdehyde dehydrogenase OS=Pseudomonas veronii 1YdBTEX2	Y			51407	4.9863	UK	CY	CE
A0A1D3K2H4	TetR family transcriptional regulator OS=Pseudomonas veronii 1YdBTEX2			Y	25780	6.2827	CY	CY	K
A0A1D3K2H8	N-succinylarginine dihydrolase OS=Pseudomonas veronii 1YdBTEX2	Y	Y	Y	48526	6.0571	CY	CY	E
A0A1D3K2I0	Acetyl-coenzyme A synthetase OS=Pseudomonas veronii 1YdBTEX2	Y	Y	Y	71555	5.9897	CY	CY	IQ(mx)
A0A1D3K2I2	AraC family transcriptional regulator OS=Pseudomonas veronii 1YdBTEX2	Y	Y		18114	5.5049	CY	CY	KT
A0A1D3K2I7	Lysine/arginine/ornithine-binding periplasmic protein OS=Pseudomonas veronii 1YdBTEX2	Y	Y	Y	28347	7.0386	PR	PR	ET

A0A1D3K2J8	Aldehyde dehydrogenase OS=Pseudomonas veronii 1YdBTEX2		Y		16918	6.0747	CY	CY	P
A0A1D3K2J9	Glycerophosphoryl diester phosphodiesterase OS=Pseudomonas veronii 1YdBTEX2	Y	Y	Y	38529.79	5.45	PR	PR	C
A0A1D3K2K2	3-oxoacyl-[acyl-carrier-protein] synthase 2 OS=Pseudomonas veronii 1YdBTEX2	Y			43263	5.5342	CY	IM	IQ(mx)
A0A1D3K2K3	Ribonucleoside-diphosphate reductase OS=Pseudomonas veronii 1YdBTEX2	Y	Y	Y	107325	5.5327	EX	CY	F
A0A1D3K2K5	Alanine--tRNA ligase OS=Pseudomonas veronii 1YdBTEX2	Y		Y	93722	5.3423	CY	CY	J
A0A1D3K2K7	Methyl-accepting chemotaxis protein CtpH OS=Pseudomonas veronii 1YdBTEX2	Y		Y	58333	5.5254	IM	IM	NT(mx)
A0A1D3K2L3	Uncharacterized HTH-type transcriptional regulator YhjC OS=Pseudomonas veronii 1YdBTEX2	Y			32955.27	6.25	CY	CY	K
A0A1D3K2L4	Probable sugar-binding periplasmic protein OS=Pseudomonas veronii 1YdBTEX2	Y	Y	Y	45582	6.1567	PR	PR	GP
A0A1D3K2N2	Long-chain-fatty-acid--CoA ligase OS=Pseudomonas veronii 1YdBTEX2	Y	Y	Y	61501.17	8.32	CY	CY	IQ
A0A1D3K2P8	Acetyl-CoA carboxylase OS=Pseudomonas veronii 1YdBTEX2	Y			13858	5.6045	CY	CY	S
A0A1D3K2Q8	AraC family transcriptional regulator OS=Pseudomonas veronii 1YdBTEX2			Y	39385	6.0015	CY	CY	K
A0A1D3K2S6	Carbamate kinase OS=Pseudomonas veronii 1YdBTEX2	Y			32790	5.1021	UK	CY	E
A0A1D3K2S7	Acetylornithine aminotransferase OS=Pseudomonas veronii 1YdBTEX2	Y	Y	Y	43245	5.7817	CY	CY	EH(mx)
A0A1D3K2S8	CAIB/BAIF family CoA transferase OS=Pseudomonas veronii 1YdBTEX2	Y	Y	Y	48084	6.9346	PR	CY	C
A0A1D3K2T8	CSD domain-containing protein OS=Pseudomonas veronii 1YdBTEX2	Y	Y	Y	7788	7.708	CY	CY	KL
A0A1D3K2T9	L-serine dehydratase OS=Pseudomonas veronii 1YdBTEX2	Y	Y	Y	48721.8	6.43	CY	CY	E
A0A1D3K2U2	Porin OS=Pseudomonas veronii 1YdBTEX2	Y	Y	Y	49479	5.9956	OM	OM	M
A0A1D3K2U3	Glycine cleavage system T protein OS=Pseudomonas veronii 1YdBTEX2	Y	Y	Y	40310	5.7056	CY	CY	E

A0A1D3K2U4	Methyl-accepting chemotaxis protein PctA OS=Pseudomonas veronii 1YdBTEX2	Y	Y	Y	67708	4.9702	IM	IM	NT(mx)
A0A1D3K2U5	Glycine dehydrogenase (decarboxylating) OS=Pseudomonas veronii 1YdBTEX2	Y	Y	Y	102259	5.6499	UK	CY	E
A0A1D3K2U9	Superoxide dismutase OS=Pseudomonas veronii 1YdBTEX2	Y		Y	21905	5.4961	CY	PR	CP
A0A1D3K2V0	Peptidoglycan-associated protein OS=Pseudomonas veronii 1YdBTEX2	Y	Y		17768	5.6748	OM	OM	MN
A0A1D3K2V5	Tol-Pal system protein TolB OS=Pseudomonas veronii 1YdBTEX2		Y	Y	45789	9.3501	CY	PR	U
A0A1D3K2V8	Arylsulfate sulfotransferase OS=Pseudomonas veronii 1YdBTEX2	Y			12578.54	9.59	CY	CY	OT
A0A1D3K2W1	Pyruvate kinase OS=Pseudomonas veronii 1YdBTEX2	Y			52052	5.9707	CY	CY	G
A0A1D3K2W4	Protein translocase subunit SecA OS=Pseudomonas veronii 1YdBTEX2	Y	Y	Y	102871	5.2559	CY	CY	UE(mx)
A0A1D3K2W7	Aspartate--tRNA(Asp/Asn) ligase OS=Pseudomonas veronii 1YdBTEX2	Y	Y	Y	66257	5.272	CY	CY	J
A0A1D3K2X0	UPF0234 protein PVE_R1G4785 OS=Pseudomonas veronii 1YdBTEX2	Y	Y	Y	18236	7.3169	CY	CY	S
A0A1D3K2X4	Exodeoxyribonuclease I OS=Pseudomonas veronii 1YdBTEX2	Y			54703.87	5.66	CY	CY	L
A0A1D3K2Y0	Methyl-accepting chemotaxis protein OS=Pseudomonas veronii 1YdBTEX2	Y	Y	Y	76074	4.8486	IM	IM	NT(mx)
A0A1D3K2Y2	Peptidase OS=Pseudomonas veronii 1YdBTEX2	Y	Y		47150	4.3711	UK	OM	P
A0A1D3K2Y3	Ornithine carbamoyltransferase OS=Pseudomonas veronii 1YdBTEX2	Y	Y	Y	37867	6.0117	CY	CY	EF
A0A1D3K2Z9	Proline--tRNA ligase OS=Pseudomonas veronii 1YdBTEX2	Y	Y	Y	63107	5.1343	CY	CY	J
A0A1D3K314	Glyceraldehyde-3-phosphate dehydrogenase OS=Pseudomonas veronii 1YdBTEX2	Y	Y	Y	36056	6.23	CY	CY	GE
A0A1D3K322	Arginine biosynthesis bifunctional protein ArgJ OS=Pseudomonas veronii 1YdBTEX2	Y			42253	5.0054	CY	CY	E
A0A1D3K323	Imelysin family lipoprotein OS=Pseudomonas veronii 1YdBTEX2		Y		38456	6.5244	PR	UK	S
A0A1D3K332	Arginine deiminase OS=Pseudomonas veronii 1YdBTEX2	Y	Y	Y	46343	5.2427	CY	CY	E

A0A1D3K334	10 kDa chaperonin OS=Pseudomonas veronii 1YdBTEX2	Y	Y	Y	10254	6.1318	CY	CY	O
A0A1D3K335	60 kDa chaperonin OS=Pseudomonas veronii 1YdBTEX2	Y	Y	Y	57069	4.7754	CY	CY	O
A0A1D3K342	Porin D OS=Pseudomonas veronii 1YdBTEX2	Y	Y	Y	46960	5.6396	OM	OM	S
A0A1D3K345	Secretion protein HylD OS=Pseudomonas veronii 1YdBTEX2	Y		Y	38015	8.9209	IM	IM	MV
A0A1D3K353	Threonine synthase OS=Pseudomonas veronii 1YdBTEX2	Y	Y	Y	51952	5.7393	CY	CY	EP
A0A1D3K358	GTP-binding protein OS=Pseudomonas veronii 1YdBTEX2	Y			22702.77	4.82	IM	UK	CO(mx)
A0A1D3K362	Uncharacterized protein slr1894 OS=Pseudomonas veronii 1YdBTEX2	Y	Y	Y	17764	5.2354	CY	CY	P
A0A1D3K366	Phosphoenolpyruvate--protein phosphotransferase OS=Pseudomonas veronii 1YdBTEX2	Y			88802	5.5474	CY	CY	GT
A0A1D3K367	Phosphoribosylformylglycinamidine synthase OS=Pseudomonas veronii 1YdBTEX2	Y	Y	Y	140552	4.8809	CY	CY	FT
A0A1D3K371	Iron-sulfur cluster assembly scaffold protein IscU OS=Pseudomonas veronii 1YdBTEX2	Y		Y	13861	5.0186	CY	CY	CP
A0A1D3K372	Nudix hydrolase domain-containing protein OS=Pseudomonas veronii 1YdBTEX2	Y			34075	5.3262	CY	CY	FH(mx)
A0A1D3K378	Protein translocase subunit SecD OS=Pseudomonas veronii 1YdBTEX2	Y	Y		67207	9.7251	IM	IM	UQ(mx)
A0A1D3K382	H-NS histone OS=Pseudomonas veronii 1YdBTEX2	Y	Y	Y	13999	9.9697	CY	CY	S
A0A1D3K388	Outer membrane protein assembly factor BamB OS=Pseudomonas veronii 1YdBTEX2	Y	Y		42902	5.5225	UK	OM	G
A0A1D3K395	2-isopropylmalate synthase OS=Pseudomonas veronii 1YdBTEX2		Y		61773	5.2236	CY	CY	E
A0A1D3K397	Nucleoside diphosphate kinase OS=Pseudomonas veronii 1YdBTEX2	Y			14943	5.2588	CY	EX	F
A0A1D3K398	Chaperone protein HscA homolog OS=Pseudomonas veronii 1YdBTEX2	Y	Y	Y	66169.28	4.78	CY	CY	OD
A0A1D3K399	50S ribosomal protein L19 OS=Pseudomonas veronii 1YdBTEX2	Y			12961	10.7021	CY	CY	J
A0A1D3K3A4	TonB-dependent receptor OS=Pseudomonas veronii 1YdBTEX2	Y	Y	Y	92999	6.4937	EX	OM	PH
A0A1D3K3A7	Uncharacterized protein OS=Pseudomonas veronii 1YdBTEX2	Y			7672	5.6089	CY	UK	S

A0A1D3K3B1	Cysteine desulfurase IscS OS=Pseudomonas veronii 1YdBTEX2	Y	Y	Y	44443	5.9165	CY	CY	E
A0A1D3K3B3	Dual-specificity RNA methyltransferase RlmN OS=Pseudomonas veronii 1YdBTEX2	Y			42244.54	8.6	CY	CY	JC
A0A1D3K3C7	Inosine-5'-monophosphate dehydrogenase OS=Pseudomonas veronii 1YdBTEX2	Y	Y	Y	51939	6.2534	CY	CY	FT(mx)
A0A1D3K3D5	30S ribosomal protein S16 OS=Pseudomonas veronii 1YdBTEX2	Y	Y	Y	9201	10.8369	CY	CY	J
A0A1D3K3F7	Rick_17kDa_Anti domain-containing protein OS=Pseudomonas veronii 1YdBTEX2		Y		18682	9.5347	PR	UK	IM
A0A1D3K3F8	12-oxophytodienoate reductase OS=Pseudomonas veronii 1YdBTEX2	Y			40370	6.0044	CY	CY	C
A0A1D3K3G0	Aminotransferase OS=Pseudomonas veronii 1YdBTEX2	Y			38920.08	8.61	PR	IM	EK
A0A1D3K3G5	Uncharacterized protein PA3922 OS=Pseudomonas veronii 1YdBTEX2	Y			48317.62	7.94	PR	UK	S
A0A1D3K3G8	Peptidyl-prolyl cis-trans isomerase OS=Pseudomonas veronii 1YdBTEX2	Y			21732	4.4194	CY	CY	O
A0A1D3K3H2	GMP synthase [glutamine-hydrolyzing] OS=Pseudomonas veronii 1YdBTEX2	Y	Y	Y	58261	5.4507	CY	CY	FH(mx)
A0A1D3K3I7	Iron-binding protein IscA OS=Pseudomonas veronii 1YdBTEX2	Y		Y	11648	4.6743	CY	CY	C
A0A1D3K3J3	Uncharacterized protein OS=Pseudomonas veronii 1YdBTEX2		Y		7492	3.835	CY	UK	S
A0A1D3K3K1	3-hydroxyisobutyrate dehydrogenase OS=Pseudomonas veronii 1YdBTEX2	Y	Y	Y	29998	5.5122	CY	CY	IG
A0A1D3K3K8	Glutathione peroxidase OS=Pseudomonas veronii	Y	Y	Y	17519	5.2383	CY	PR	CO
A0A1D3K3K9	Protein CreA OS=Pseudomonas veronii 1YdBTEX2		Y		16860	9.2183	CY	UK	S
A0A1D3K3L5	Methyl-accepting chemotaxis protein McpS OS=Pseudomonas veronii 1YdBTEX2	Y			68336.28	5.23	IM	IM	NT
A0A1D3K3L8	Muconolactone Delta-isomerase OS=Pseudomonas veronii 1YdBTEX2		Y	Y	11133	6.3193	CY	UK	Q
A0A1D3K3M6	RNA polymerase-binding transcription factor DksA OS=Pseudomonas veronii 1YdBTEX2	Y	Y		17100	4.9497	CY	CY	T

A0A1D3K3M8	PAS sensor protein OS=Pseudomonas veronii 1YdBTEX2		Y		17296.85	6.12	CY	UK	TK
A0A1D3K3P0	Anthranilate 1_2-dioxygenase small subunit OS=Pseudomonas veronii 1YdBTEX2	Y		Y	19536	6.8965	CY	CY	Q
A0A1D3K3P2	Hypothetical membrane protein OS=Pseudomonas veronii 1YdBTEX2	Y			12683	8.5503	CY	IM	H
A0A1D3K3P7	50S ribosomal protein L21 OS=Pseudomonas veronii 1YdBTEX2	Y	Y	Y	11596	10.2993	CY	CY	J
A0A1D3K3P9	Transcription termination/antitermination protein NusA OS=Pseudomonas veronii 1YdBTEX2	Y	Y	Y	54381	4.3506	CY	CY	KJ
A0A1D3K3Q9	Carbamoyl-phosphate synthase large chain OS=Pseudomonas veronii 1YdBTEX2	Y		Y	117424	4.9233	UK	UK	F
A0A1D3K3R3	APH domain-containing protein OS=Pseudomonas veronii 1YdBTEX2	Y			57785.94	5.79	CY	CY	M
A0A1D3K3R6	Catechol 1_2-dioxygenase OS=Pseudomonas veronii 1YdBTEX2	Y		Y	34546	4.8457	CY	CY	Q
A0A1D3K3S6	Ornithine aminotransferase OS=Pseudomonas veronii 1YdBTEX2		Y	Y	20369	9.936	OM	PR	S
A0A1D3K3T4	Thioredoxin reductase OS=Pseudomonas veronii 1YdBTEX2	Y		Y	33696	4.9512	PR	UK	C
A0A1D3K3T6	Cell envelope biogenesis protein OmpA OS=Pseudomonas veronii 1YdBTEX2		Y		17302	9.4072	PR	OM	MN
A0A1D3K3U5	50S ribosomal protein L27 OS=Pseudomonas veronii 1YdBTEX2		Y		9767	10.8545	CY	CY	J
A0A1D3K3U7	Chaperone protein DnaK OS=Pseudomonas veronii 1YdBTEX2	Y	Y	Y	68413	4.7124	CY	CY	ODE
A0A1D3K3U9	Ferric uptake regulation protein OS=Pseudomonas veronii 1YdBTEX2	Y	Y	Y	15117	5.1606	CY	CY	P
A0A1D3K3V1	Carbamoyl-phosphate synthase small chain OS=Pseudomonas veronii 1YdBTEX2	Y	Y	Y	40686	5.3481	CY	CY	FH(mx)
A0A1D3K3V4	Phosphoglucosamine mutase OS=Pseudomonas veronii 1YdBTEX2	Y	Y	Y	47719	5.5254	CY	CY	G
A0A1D3K3W3	Methylmalonate-semialdehyde dehydrogenase [acylating]_ mitochondrial OS=Pseudomonas veronii 1YdBTEX2	Y	Y	Y	53790	5.8125	CY	CY	CEK

A0A1D3K3W6	Chaperone protein DnaJ OS=Pseudomonas veronii 1YdBTEX2	Y		Y	40362	6.2065	OM	CY	O
A0A1D3K3X1	Acetone carboxylase gamma subunit OS=Pseudomonas veronii 1YdBTEX2		Y	Y	19482	5.3862	CY	CY	EQ
A0A1D3K3Z2	Transposase OS=Pseudomonas veronii 1YdBTEX2			Y	24110	8.9883	UK	UK	KL
A0A1D3K3Z6	Ketol-acid reductoisomerase (NADP(+)) OS=Pseudomonas veronii 1YdBTEX2	Y	Y	Y	36202	5.3525	CY	CY	IEH
A0A1D3K402	Acetone carboxylase alpha subunit OS=Pseudomonas veronii 1YdBTEX2	Y	Y	Y	86396	5.4595	CY	CY	EQ
A0A1D3K403	Glucose-6-phosphate isomerase OS=Pseudomonas veronii 1YdBTEX2	Y		Y	61455.19	6.4	IM	CY	G
A0A1D3K406	Serine hydroxymethyltransferase OS=Pseudomonas veronii 1YdBTEX2	Y			44825	5.9399	CY	CY	EH
A0A1D3K408	Energy-dependent translational throttle protein EttA OS=Pseudomonas veronii 1YdBTEX2	Y		Y	61421	5.187	CY	CY	VQ(mx)
A0A1D3K427	Catalase OS=Pseudomonas veronii 1YdBTEX2	Y	Y	Y	53782.22	6.97	CY	CY	C
A0A1D3K431	Chemotaxis protein OS=Pseudomonas veronii 1YdBTEX2	Y		Y	76464	4.7607	IM	IM	NT(mx)
A0A1D3K434	Translation initiation factor IF-2 OS=Pseudomonas veronii 1YdBTEX2	Y	Y	Y	90498	6.5156	CY	CY	JM(mx)
A0A1D3K439	Inner membrane protein YjiY OS=Pseudomonas veronii 1YdBTEX2	Y			73786.48	9.1	IM	IM	T
A0A1D3K442	Uncharacterized protein OS=Pseudomonas veronii 1YdBTEX2	Y	Y	Y	54124.43	6.81	CY	UK	S
A0A1D3K443	Polyribonucleotide nucleotidyltransferase OS=Pseudomonas veronii 1YdBTEX2	Y	Y	Y	74887	5.0259	UK	CY	J
A0A1D3K444	Transcription elongation factor GreA OS=Pseudomonas veronii 1YdBTEX2	Y			17371	5.1182	CY	CY	KJ
A0A1D3K451	Acetone carboxylase beta subunit OS=Pseudomonas veronii 1YdBTEX2		Y	Y	77668	5.0962	CY	CY	EQ
A0A1D3K459	TetR family transcriptional regulator OS=Pseudomonas veronii 1YdBTEX2			Y	25686	6.4365	CY	CY	K
A0A1D3K465	Protein GrpE OS=Pseudomonas veronii 1YdBTEX2	Y		Y	20671	4.4458	CY	CY	O

A0A1D3K476|	Shikimate dehydrogenase (NADP(+)) OS=Pseudomonas veronii 1YdBTEX2	Y		Y	30332	5.5869	CY	CY	EH
A0A1D3K477	Glutamate-1-semialdehyde 2_1-aminomutase OS=Pseudomonas veronii 1YdBTEX2	Y			45122	5.3306	CY	CY	HE(mx)
A0A1D3K478	LPS-assembly lipoprotein LptE OS=Pseudomonas veronii 1YdBTEX2	Y	Y	Y	22362	5.6133	OM	CY	M
A0A1D3K486	Membrane-bound lytic murein transglycosylase B OS=Pseudomonas veronii 1YdBTEX2		Y		37873	5.5034	CY	IM	M
A0A1D3K488	Leucine--tRNA ligase OS=Pseudomonas veronii 1YdBTEX2	Y	Y	Y	97123	5.3438	CY	CY	J
A0A1D3K496	ABC transporter substrate-binding protein OS=Pseudomonas veronii 1YdBTEX2			Y	27082	4.1982	PR	PR	E
A0A1D3K4A0	Magnesium and cobalt efflux protein CorC OS=Pseudomonas veronii 1YdBTEX2	Y	Y	Y	31413.24	4.93	CY	ML (C, IM)	PT
A0A1D3K4A8	AMP nucleosidase OS=Pseudomonas veronii 1YdBTEX2	Y	Y	Y	54477	6.3633	CY	CY	F
A0A1D3K4B7	Endoribonuclease YbeY OS=Pseudomonas veronii			Y	17235	4.0254	CY	CY	JC
A0A1D3K4C4	Uncharacterized protein OS=Pseudomonas veronii 1YdBTEX2	Y			56148.75	5.7	CY	UK	S
A0A1D3K4D2	Uncharacterized protein YraR OS=Pseudomonas veronii 1YdBTEX2	Y			23402	8.0112	CY	CY	GM
A0A1D3K4D7	Endopeptidase La OS=Pseudomonas veronii 1YdBTEX2	Y			89084.55	4.85	CY	CY	OP
A0A1D3K4G0	MaoC family dehydratase OS=Pseudomonas veronii 1YdBTEX2	Y	Y	Y	16781.79	5.9	UK	CY	CI
A0A1D3K4G6	Outer membrane protein W OS=Pseudomonas veronii 1YdBTEX2	Y	Y	Y	24771	8.7847	OM	OM	M
A0A1D3K4H2	Transcription antitermination protein NusB OS=Pseudomonas veronii 1YdBTEX2	Y			18865.78	5.56	CY	CY	J
A0A1D3K4H5	6_7-dimethyl-8-ribityllumazine synthase OS=Pseudomonas veronii 1YdBTEX2	Y	Y	Y	16437	5.6177	CY	CY	H
A0A1D3K4H8	Uncharacterized protein OS=Pseudomonas veronii 1YdBTEX2	Y			35413.98	9.37	CY	UK	S
A0A1D3K4I6	30S ribosomal protein S4 OS=Pseudomonas veronii 1YdBTEX2	Y	Y		23062	10.1177	CY	CY	J

A0A1D3K4I7	50S ribosomal protein L17 OS=Pseudomonas veronii 1YdBTEX2	Y	Y	Y	14406	11.0186	CY	CY	J
A0A1D3K4J2	Catalase OS=Pseudomonas veronii 1YdBTEX2	Y	Y	Y	53667	6.8833	PR	PR	CP
A0A1D3K4J6	50S ribosomal protein L6 OS=Pseudomonas veronii 1YdBTEX2	Y	Y	Y	19185	10.0459	CY	CY	J
A0A1D3K4K1	Inorganic pyrophosphatase OS=Pseudomonas veronii 1YdBTEX2	Y	Y	Y	19365	4.5981	CY	CY	C
A0A1D3K4K4	50S ribosomal protein L18 OS=Pseudomonas veronii 1YdBTEX2	Y	Y	Y	12634	10.7095	CY	CY	J
A0A1D3K4K6	Thioredoxin OS=Pseudomonas veronii 1YdBTEX2	Y			31627	4.4897	OM	CY	O
A0A1D3K4K8	50S ribosomal protein L22 OS=Pseudomonas veronii 1YdBTEX2	Y	Y		11903	10.6216	CY	CY	J
A0A1D3K4L0	Hydrogenase OS=Pseudomonas veronii 1YdBTEX2	Y			28113.39	5.82	OM	UK	HK
A0A1D3K4L2	50S ribosomal protein L2 OS=Pseudomonas veronii 1YdBTEX2	Y	Y	Y	29673	11.6367	CY	CY	J
A0A1D3K4L5	30S ribosomal protein S3 OS=Pseudomonas veronii 1YdBTEX2	Y	Y	Y	25736	10.4531	CY	CY	J
A0A1D3K4M1	DNA-directed RNA polymerase subunit beta OS=Pseudomonas veronii 1YdBTEX2	Y	Y	Y	150894	5.5151	OM	CY	K
A0A1D3K4M2	Single-stranded DNA-binding protein OS=Pseudomonas veronii 1YdBTEX2	Y	Y	Y	18977	5.8184	CY	CY	L
A0A1D3K4M3	30S ribosomal protein S12 OS=Pseudomonas veronii 1YdBTEX2		Y		13716.41	11.1	CY	CY	J
A0A1D3K4M5	30S ribosomal protein S7 OS=Pseudomonas veronii 1YdBTEX2		Y	Y	17636	10.5762	CY	CY	J
A0A1D3K4M9	50S ribosomal protein L15 OS=Pseudomonas veronii 1YdBTEX2	Y	Y	Y	15215	11.2471	CY	CY	J
A0A1D3K4N2	30S ribosomal protein S13 OS=Pseudomonas veronii 1YdBTEX2	Y	Y	Y	13272	11.3862	CY	CY	J
A0A1D3K4N4	50S ribosomal protein L14 OS=Pseudomonas veronii 1YdBTEX2	Y	Y	Y	13401	11.376	CY	CY	J
A0A1D3K4N7	Aldehyde dehydrogenase OS=Pseudomonas veronii 1YdBTEX2	Y	Y	Y	54882	5.8169	CY	CY	CEK

A0A1D3K4N8	30S ribosomal protein S17 OS=Pseudomonas veronii 1YdBTEX2	Y	Y	Y	10090	10.1455	CY	CY	J
A0A1D3K4N9	Putative 3-methyladenine DNA glycosylase OS=Pseudomonas veronii 1YdBTEX2	Y	Y	Y	25409	9.2065	CY	UK	L
A0A1D3K4P2	50S ribosomal protein L24 OS=Pseudomonas veronii 1YdBTEX2	Y	Y	Y	11338	10.5615	CY	CY	J
A0A1D3K4P3	50S ribosomal protein L3 OS=Pseudomonas veronii 1YdBTEX2	Y	Y	Y	22563	10.3638	CY	CY	J
A0A1D3K4P6	Ribulose-phosphate 3-epimerase OS=Pseudomonas veronii 1YdBTEX2	Y	Y	Y	24076	4.7725	CY	CY	GH
A0A1D3K4P8	30S ribosomal protein S11 OS=Pseudomonas veronii 1YdBTEX2	Y	Y		13607	11.2646	CY	CY	J
A0A1D3K4Q0	30S ribosomal protein S10 OS=Pseudomonas veronii 1YdBTEX2	Y	Y	Y	11745	10.248	CY	CY	J
A0A1D3K4Q1	50S ribosomal protein L4 OS=Pseudomonas veronii 1YdBTEX2	Y	Y	Y	21954	10.2363	CY	CY	J
A0A1D3K4Q3	50S ribosomal protein L1 OS=Pseudomonas veronii 1YdBTEX2	Y	Y	Y	24060	10.043	CY	CY	J
A0A1D3K4Q4	Anthranilate synthase component 1 OS=Pseudomonas veronii 1YdBTEX2	Y		Y	54351	4.8413	UK	CY	EH
A0A1D3K4Q5	Anthranilate synthase component 2 OS=Pseudomonas veronii 1YdBTEX2		Y		21643	5.064	CY	CY	EH
A0A1D3K4Q7	50S ribosomal protein L5 OS=Pseudomonas veronii 1YdBTEX2	Y	Y	Y	20444	10.1499	CY	CY	J
A0A1D3K4Q9	50S ribosomal protein L11 OS=Pseudomonas veronii 1YdBTEX2	Y	Y	Y	14902	10.2437	CY	CY	J
A0A1D3K4R1	50S ribosomal protein L10 OS=Pseudomonas veronii 1YdBTEX2	Y	Y	Y	17636	9.7061	CY	CY	J
A0A1D3K4R6	50S ribosomal protein L23 OS=Pseudomonas veronii 1YdBTEX2	Y	Y	Y	10938	10.5088	CY	CY	J
A0A1D3K4R7	Bacterioferritin OS=Pseudomonas veronii 1YdBTEX2	Y	Y	Y	18086	4.4692	CY	CY	P
A0A1D3K4R8	UPF0229 protein PVE_R1G5476 OS=Pseudomonas veronii 1YdBTEX2	Y	Y	Y	48607	6.4761	CY	CY	S

A0A1D3K4S6	50S ribosomal protein L7/L12 OS=Pseudomonas veronii 1YdBTEX2	Y	Y	Y	12458	4.4517	CY	CY	J
A0A1D3K4S9	30S ribosomal protein S5 OS=Pseudomonas veronii 1YdBTEX2	Y	Y	Y	17676	10.5454	CY	CY	J
A0A1D3K4T9	Spermidine/putrescine ABC transporter substrate-binding protein OS=Pseudomonas veronii 1YdBTEX2		Y	Y	37724	5.6572	PR	PR	EP
A0A1D3K4U0	50S ribosomal protein L16 OS=Pseudomonas veronii 1YdBTEX2	Y			15419	11.7642	CY	CY	J
A0A1D3K4U2	Chaperone SurA OS=Pseudomonas veronii 1YdBTEX2	Y		Y	49411	5.3818	CY	ML (C, P)	MO
A0A1D3K4U3	3_4-dihydroxy-2-butanone 4-phosphate synthase OS=Pseudomonas veronii 1YdBTEX2	Y	Y	Y	39382	5.0625	CY	CY	H
A0A1D3K4U4	Coenzyme PQQ synthesis protein B OS=Pseudomonas veronii 1YdBTEX2	Y	Y	Y	33073	4.9761	UK	CY	P
A0A1D3K4U9	Elongation factor G OS=Pseudomonas veronii 1YdBTEX2	Y	Y	Y	77048	5.0845	CY	CY	JT(mx)
A0A1D3K4V1	LPS-assembly protein LptD OS=Pseudomonas veronii 1YdBTEX2	Y	Y	Y	104426	5.2764	OM	OM	M
A0A1D3K4V5	Long-chain-alcohol dehydrogenase 1 OS=Pseudomonas veronii 1YdBTEX2			Y	39583	5.1504	CY	CY	C
A0A1D3K4W0	Glycine cleavage system transcriptional repressor OS=Pseudomonas veronii 1YdBTEX2	Y	Y		18200.53	5.3	CY	CY	ET
A0A1D3K4W5	Aminoglycoside phosphotransferase OS=Pseudomonas veronii 1YdBTEX2	Y			38908.08	5.2	CY	CY	M
A0A1D3K4W6	Amine oxidase OS=Pseudomonas veronii 1YdBTEX2	Y			62551	5.9341	CY	UK	EQ
A0A1D3K4W8	DNA-directed RNA polymerase subunit alpha OS=Pseudomonas veronii 1YdBTEX2	Y	Y	Y	36623	4.7183	CY	CY	K
A0A1D3K4X6	TonB-denpendent receptor OS=Pseudomonas veronii 1YdBTEX2	Y	Y	Y	60843	5.3994	OM	OM	PH
A0A1D3K4Y0	30S ribosomal protein S8 OS=Pseudomonas veronii 1YdBTEX2	Y	Y	Y	14106	9.8394	CY	CY	J
A0A1D3K4Z6	Sarcosine oxidase subunit delta OS=Pseudomonas veronii 1YdBTEX2		Y		11499	5.9282	CY	CY	E
A0A1D3K4Z9	50S ribosomal protein L29 OS=Pseudomonas veronii 1YdBTEX2	Y	Y	Y	7167	11.1021	CY	CY	J

A0A1D3K500	DNA-directed RNA polymerase subunit beta' OS=Pseudomonas veronii 1YdBTEX2	Y	Y	Y	154570	6.8789	CY	CY	K
A0A1D3K508	Elongation factor Tu OS=Pseudomonas veronii 1YdBTEX2	Y	Y	Y	43551	5.1943	CY	CY	JT(mx)
A0A1D3K511	Malate synthase G OS=Pseudomonas veronii 1YdBTEX2		Y	Y	78913	5.5269	CY	CY	C
A0A1D3K526	N-acetyl-gamma-glutamyl-phosphate reductase OS=Pseudomonas veronii 1YdBTEX2	Y			36618	6.3281	UK	CY	E
A0A1D3K530	Serine hydroxymethyltransferase OS=Pseudomonas veronii 1YdBTEX2	Y	Y	Y	44671	6.1904	UK	CY	E
A0A1D3K535	Indole-3-glycerol phosphate synthase OS=Pseudomonas veronii 1YdBTEX2	Y		Y	30252.05	4.99	CY	CY	E
A0A1D3K540	Phosphoglycerate kinase OS=Pseudomonas veronii 1YdBTEX2	Y	Y		40167	4.8706	UK	CY	FG
A0A1D3K541	Peptidase M19 OS=Pseudomonas veronii 1YdBTEX2			Y	35862	5.3218	CY	CY	E
A0A1D3K546	Transketolase OS=Pseudomonas veronii 1YdBTEX2	Y	Y	Y	72573	4.9248	CY	CY	CGH
A0A1D3K548	Uncharacterized protein YeaG OS=Pseudomonas veronii 1YdBTEX2	Y	Y	Y	73763	5.54	CY	CY	T
A0A1D3K549	Sarcosine oxidase subunit beta OS=Pseudomonas veronii 1YdBTEX2	Y	Y	Y	45155.73	8.2	UK	CY	EH(mx)
A0A1D3K558	RNA polymerase sigma factor RpoD OS=Pseudomonas veronii 1YdBTEX2	Y	Y	Y	69291	4.8516	CY	CY	K
A0A1D3K560	Glutamate/aspartate periplasmic-binding protein OS=Pseudomonas veronii 1YdBTEX2	Y	Y	Y	32887	7.9395	UK	PR	ET
A0A1D3K563	Rhamnosyltransferase 1 subunit A OS=Pseudomonas veronii 1YdBTEX2	Y	Y	Y	33028	6.4922	CY	CY	IC
A0A1D3K565	Pyrroloquinoline-quinone synthase OS=Pseudomonas veronii 1YdBTEX2	Y		Y	29030	6.4482	CY	CY	H
A0A1D3K566	Adenosylmethionine-8-amino-7-oxononanoate aminotransferase OS=Pseudomonas veronii 1YdBTEX2	Y			52365.34	5.8	CY	CY	EH
A0A1D3K569	Glutathione-independent formaldehyde dehydrogenase OS=Pseudomonas veronii 1YdBTEX2	Y		Y	42186	6.1626	CY	CY	CE(mx)
A0A1D3K571	Malonyl CoA-acyl carrier protein transacylase OS=Pseudomonas veronii 1YdBTEX2	Y			32206	6.6929	UK	CY	IQ

A0A1D3K577	Hydrolase in pqqF 5'region OS=Pseudomonas veronii 1YdBTEX2	Y			28745	4.6421	CY	CY	TH(mx)
A0A1D3K578	S-adenosylmethionine synthase OS=Pseudomonas veronii 1YdBTEX2	Y	Y	Y	45682	5.3408	CY	CY	H
A0A1D3K579	Methylenetetrahydrofolate reductase OS=Pseudomonas veronii 1YdBTEX2	Y			31287	5.9795	CY	CY	E
A0A1D3K591	Adenosylhomocysteinase OS=Pseudomonas veronii 1YdBTEX2	Y	Y	Y	51282	5.2734	CY	CY	H
A0A1D3K596	Aspartate carbamoyltransferase OS=Pseudomonas veronii 1YdBTEX2	Y	Y	Y	36358.97	7.14	CY	CY	EF
A0A1D3K5A5	Uncharacterized protein OS=Pseudomonas veronii 1YdBTEX2			Y	7179	5.8682	PR	UK	X
A0A1D3K5A9	Thioesterase OS=Pseudomonas veronii 1YdBTEX2	Y	Y	Y	15057	7.314	CY	CY	IQ
A0A1D3K5B8	Aldehyde dehydrogenase OS=Pseudomonas veronii 1YdBTEX2	Y		Y	52119	8.8096	IM	CY	C
A0A1D3K5C8	Thiazole synthase OS=Pseudomonas veronii 1YdBTEX2	Y	Y	Y	28288	5.0376	CY	CY	H
A0A1D3K5D1	Dihydroxy-acid dehydratase OS=Pseudomonas veronii 1YdBTEX2	Y			65664	5.7085	CY	CY	EG
A0A1D3K5D5	UPF0312 protein PVE_R1G5612 OS=Pseudomonas veronii 1YdBTEX2	Y	Y	Y	21272	7.8135	PR	UK	O
A0A1D3K5D8	4-deoxy-4-formamido-L-arabinose-phosphoundecaprenol deformylase ArnD OS=Pseudomonas veronii 1YdBTEX2	Y	Y	Y	79551	6.2769	IM	IM	PC
A0A1D3K5F0	RNA pyrophosphohydrolase OS=Pseudomonas veronii 1YdBTEX2	Y	Y	Y	18868.82	9.3	CY	CY	LF
A0A1D3K5G0	ATPase OS=Pseudomonas veronii 1YdBTEX2	Y	Y	Y	15344	9.3003	PR	UK	S
A0A1D3K5H0	Spermidine-binding periplasmic protein SpuE OS=Pseudomonas veronii 1YdBTEX2	Y	Y	Y	39932	5.9912	PR	PR	P
A0A1D3K5I5	Fructose-1_6-bisphosphate aldolase OS=Pseudomonas veronii 1YdBTEX2	Y		Y	38385	5.291	PR	CY	G
A0A1D3K5J1	Ribosomal RNA small subunit methyltransferase E OS=Pseudomonas veronii 1YdBTEX2	Y			26271	5.7671	CY	UK	J
A0A1D3K5K2	Glutamine synthetase OS=Pseudomonas veronii 1YdBTEX2	Y	Y	Y	51023	4.8311	CY	CY	E

A0A1D3K5K4	Gamma aminobutyrate transaminase 1_ mitochondrial OS=Pseudomonas veronii 1YdBTEX2	Y	Y	Y	49859	5.6689	CY	CY	EH(mx)
A0A1D3K5K5	Probable binding protein component of ABC iron transporter PA5217 OS=Pseudomonas veronii 1YdBTEX2	Y			36676	6.2651	PR	PR	P
A0A1D3K5L2	Anaerobically-induced outer membrane porin OprE OS=Pseudomonas veronii 1YdBTEX2	Y	Y	Y	47187	5.6455	OM	OM	S
A0A1D3K5L4	Transporter OS=Pseudomonas veronii 1YdBTEX2	Y	Y	Y	34637	7.6758	OM	IM	MVI
A0A1D3K5L7	Dihydroorotase-like protein OS=Pseudomonas veronii 1YdBTEX2	Y	Y	Y	44042	5.2983	UK	CY	FQ(mx)
A0A1D3K5L8	Putrescine-binding periplasmic protein SpuD OS=Pseudomonas veronii 1YdBTEX2	Y	Y	Y	40054	6.5391	CY	PR	PE
A0A1D3K5L9	Thioredoxin OS=Pseudomonas veronii 1YdBTEX2	Y			11764	4.6553	CY	CY	O
A0A1D3K5M3	Gamma-glutamylputrescine synthetase PuuA OS=Pseudomonas veronii 1YdBTEX2	Y	Y	Y	50968	4.6538	CY	CY	E
A0A1D3K5M7	Exopolyphosphatase OS=Pseudomonas veronii 1YdBTEX2	Y			56026	6.1025	CY	CY	FP
A0A1D3K5N7	UPF0149 protein PFL_5969 OS=Pseudomonas veronii 1YdBTEX2	Y			19768.75	4.2	CY	UK	L
A0A1D3K5N9	Uncharacterized ABC transporter ATP-binding protein YheS OS=Pseudomonas veronii 1YdBTEX2			Y	70801	5.3496	CY	ML (CY, OM	VH(mx)
A0A1D3K5P0	Gamma-glutamylcyclotransferase OS=Pseudomonas veronii 1YdBTEX2	Y			24595.21	7.7	CY	CY	P
A0A1D3K5P2	D-3-phosphoglycerate dehydrogenase OS=Pseudomonas veronii 1YdBTEX2	Y	Y	Y	44303	6.0747	CY	CY	EH(mx)
A0A1D3K5P5	Uroporphyrin-III C-methyltransferase OS=Pseudomonas veronii 1YdBTEX2	Y	Y		40390	5.0713	IM	IM	H
A0A1D3K5Q0	GMC family oxidoreductase OS=Pseudomonas veronii 1YdBTEX2	Y		Y	57177	6.7383	UK	UK	EC
A0A1D3K5Q1	UDP-4-amino-4-deoxy-L-arabinose--oxoglutarate aminotransferase OS=Pseudomonas veronii 1YdBTEX2	Y	Y	Y	41290	5.2119	CY	CY	E
A0A1D3K5R1	Cell division protein ZapA OS=Pseudomonas veronii 1YdBTEX2	Y	Y	Y	11579.9	5.64	CY	UK	D
A0A1D3K5R3	Probable 3-mercaptopyruvate sulfurtransferase OS=Pseudomonas veronii 1YdBTEX2	Y		Y	30795	5.105	CY	CY	P

A0A1D3K5R5	Delta-aminolevulinic acid dehydratase OS=Pseudomonas veronii 1YdBTEX2	Y	Y		36996	4.9438	CY	CY	H
A0A1D3K5S1	sn-glycerol-3-phosphate transporter OS=Pseudomonas veronii 1YdBTEX2	Y		Y	17731	9.5112	EX	UK	S
A0A1D3K5S6	Uncharacterized protein OS=Pseudomonas veronii 1YdBTEX2	Y	Y	Y	34757	11.0405	CY	UK	S
A0A1D3K5S7	Polyphosphate kinase OS=Pseudomonas veronii 1YdBTEX2	Y	Y	Y	82954.62	6.78	OM	ML (C, IM)	P
A0A1D3K5T3	Aminomethyltransferase OS=Pseudomonas veronii 1YdBTEX2	Y			39553	5.7788	CY	CY	EC
A0A1D3K5U2	Spermidine/putrescine import ATP-binding protein PotA OS=Pseudomonas veronii 1YdBTEX2	Y	Y	Y	42374	5.9355	CY	IM	PE(mx)
A0A1D3K5U9	Argininosuccinate lyase OS=Pseudomonas veronii 1YdBTEX2		Y		51568	5.521	CY	CY	EF
A0A1D3K5Y5	Chemotaxis protein OS=Pseudomonas veronii 1YdBTEX2	Y		Y	70053	5.1577	IM	IM	NT(mx)
A0A1D3K5Z6	Transcription termination factor Rho OS=Pseudomonas veronii 1YdBTEX2	Y	Y	Y	46938	7.5352	CY	CY	KU(mx)
A0A1D3K5Z7	Aldehyde dehydrogenase PuuC OS=Pseudomonas veronii 1YdBTEX2	Y	Y	Y	52651	5.4478	CY	CY	CK
A0A1D3K5Z8	Glycine cleavage system H protein OS=Pseudomonas veronii 1YdBTEX2	Y			13179.61	4.22	CY	UK	E
A0A1D3K607	Phosphomannomutase/phosphoglucomutase OS=Pseudomonas veronii 1YdBTEX2	Y	Y	Y	50548	5.3657	CY	CY	GJM
A0A1D3K616	Biopolymer transport protein ExbB OS=Pseudomonas veronii 1YdBTEX2	Y	Y	Y	29676.73	6.6	IM	IM	U
A0A1D3K628	Uncharacterized protein OS=Pseudomonas veronii 1YdBTEX2	Y			6569	5.0669	CY	UK	T
A0A1D3K635	Serine hydroxymethyltransferase OS=Pseudomonas veronii 1YdBTEX2	Y			46173	6.2871	CY	CY	E
A0A1D3K642	Glyoxalase OS=Pseudomonas veronii 1YdBTEX2		Y		23116.76	4.92	CY	CY	Q
A0A1D3K653	LysR family transcriptional regulator OS=Pseudomonas veronii 1YdBTEX2	Y	Y		33503.74	6.49	CY	CY	K
A0A1D3K654	Protein ClpV1 OS=Pseudomonas veronii 1YdBTEX2	Y		Y	96909	5.4185	CY	CY	OL(mx)

A0A1D3K666	Biopolymer transport protein ExbD OS=Pseudomonas veronii 1YdBTEX2	Y			15211	4.6714	PR	IM	U
A0A1D3K671	EvpB family type VI secretion protein OS=Pseudomonas veronii 1YdBTEX2	Y		Y	55675	5.1196	CY	CY	K
A0A1D3K673	Aspartate ammonia-lyase OS=Pseudomonas veronii 1YdBTEX2	Y	Y	Y	51058	5.3745	CY	CY	ECF
A0A1D3K674	Uncharacterized protein OS=Pseudomonas veronii 1YdBTEX2	Y		Y	18388	5.1064	UK	CY	K
A0A1D3K679	Type VI secretion protein ImpA OS=Pseudomonas veronii 1YdBTEX2	Y	Y		36751.68	4.56	CY	UK	M
A0A1D3K684	N5-carboxyaminoimidazole ribonucleotide mutase OS=Pseudomonas veronii 1YdBTEX2		Y		16907	6.3237	UK	UK	F
A0A1D3K692	Phosphate regulon transcriptional regulatory protein PhoB OS=Pseudomonas veronii 1YdBTEX2	Y			25565.33	5.25	CY	CY	KT
A0A1D3K696	LysR family transcriptional regulator OS=Pseudomonas veronii 1YdBTEX2			Y	36345	8.2529	CY	CY	K
A0A1D3K699	Uncharacterized protein OS=Pseudomonas veronii 1YdBTEX2		Y	Y	13695	8.7466	PR	PR	P
A0A1D3K6A1	Pyruvate carboxylase subunit B OS=Pseudomonas veronii 1YdBTEX2	Y	Y	Y	65353	5.4243	CY	CY	CQ(mx)
A0A1D3K6A5	Protein hcp1 OS=Pseudomonas veronii 1YdBTEX2	Y	Y	Y	17507	6.5068	CY	EX	S
A0A1D3K6A7	D-amino acid dehydrogenase OS=Pseudomonas veronii 1YdBTEX2	Y	Y	Y	47149.32	8.18	PR	ML (C a IM)	EH(mx)
A0A1D3K6A9	Phosphate-binding protein OS=Pseudomonas veronii 1YdBTEX2	Y	Y	Y	34609	9.1538	OM	IM	P
A0A1D3K6E1	Cytochrome c domain-containing protein OS=Pseudomonas veronii 1YdBTEX2			Y	9064.5	6.8	CY	UK	C
A0A1D3K6E4	DNA-binding protein HU-alpha OS=Pseudomonas veronii 1YdBTEX2	Y	Y	Y	9814	10.6743	CY	CY	LK
A0A1D3K6E7	Gly-zipper_YMGG domain-containing protein OS=Pseudomonas veronii 1YdBTEX2	Y			32460	9.5142	OM	UK	MI
A0A1D3K6F2	Chemotaxis protein CheY OS=Pseudomonas veronii 1YdBTEX2	Y	Y	Y	32512.18	8.95	CY	CY	TK

A0A1D3K6H5	DNA-directed RNA polymerase subunit omega OS=Pseudomonas veronii 1YdBTEX2		Y		9820	4.103	CY	CY	K
A0A1D3K6I0	Probable fatty acid methyltransferase OS=Pseudomonas veronii 1YdBTEX2	Y		Y	44080	5.272	CY	CY	QH(mx)
A0A1D3K6L5	Biotin carboxylase OS=Pseudomonas veronii 1YdBTEX2	Y			51846.68	6.13	CY	CY	IF
A0A1D3K6M0	Uncharacterized protein OS=Pseudomonas veronii 1YdBTEX2	Y			51938.76	6.1	CY	CY	S
A0A1D3K6N0	Bifunctional protein GlmU OS=Pseudomonas veronii 1YdBTEX2	Y	Y	Y	48747.57	6.4	CY	CY	JM
A0A1D3K6N5	ATP synthase subunit beta OS=Pseudomonas veronii 1YdBTEX2	Y	Y		49488	4.7241	CY	CY	CNU
A0A1D3K6Q5	ATP synthase subunit delta OS=Pseudomonas veronii 1YdBTEX2	Y			19302.22	5.42	CY	CY	TU
A0A1D3K6Q6	ATP synthase subunit alpha OS=Pseudomonas veronii 1YdBTEX2	Y	Y	Y	55301	5.3027	CY	CY	CNU
A0A1D3K6R9	ATP synthase gamma chain OS=Pseudomonas veronii 1YdBTEX2	Y	Y	Y	31434	8.1577	CY	CY	C
A0A1D3K6U7	ATP synthase subunit b OS=Pseudomonas veronii	Y	Y	Y	16832	6.1055	IM	IM	S
A0A1D3K6V4	Aldehyde dehydrogenase OS=Pseudomonas veronii 1YdBTEX2	Y			18024	5.8491	OM	UK	M
A0A1D3K6V8	Transposase OS=Pseudomonas veronii 1YdBTEX2			Y	57419	8.5063	CY	CY	KL
A0A1D3K6V9	Glutamine--fructose-6-phosphate aminotransferase [isomerizing] OS=Pseudomonas veronii 1YdBTEX2	Y	Y	Y	66081	5.7437	CY	CY	MF
A0A1D3K6X1	DNA polymerase OS=Pseudomonas veronii 1YdBTEX2	Y			17365	7.1162	CY	UK	X
A0A1D3K710	Tn7 transposition protein B OS=Pseudomonas veronii 1YdBTEX2	Y			82430.26	8.35	CY	CY	L
A0A1D3K723	PLDc_2 domain-containing protein OS=Pseudomonas veronii 1YdBTEX2	Y			37719.17	6.1	CY	CY	L
A0A1D3K730	Aldehyde dehydrogenase OS=Pseudomonas veronii 1YdBTEX2		Y	Y	18054	7.377	OM	UK	M
A0A1D3K738	Uncharacterized protein OS=Pseudomonas veronii 1YdBTEX2			Y	7719	9.2578	CY	UK	S
A0A1D3K740	Membrane protein OS=Pseudomonas veronii 1YdBTEX2	Y			26561.88	5.99	IM	UK	MU

A0A1D3K765	Catabolite gene activator protein OS=Pseudomonas veronii 1YdBTEX2			Y	27055	8.6865	CY	UK	KT
A0A1D3K7B5	Uncharacterized protein OS=Pseudomonas veronii 1YdBTEX2	Y	Y	Y	62364.94	5.61	CY	CY	CI
A0A1D3K7D2	Twitching motility protein OS=Pseudomonas veronii 1YdBTEX2			Y	38383	9.2417	CY	CY	NU
A0A1D3K7I1	Fe2OG dioxygenase domain-containing protein OS=Pseudomonas veronii 1YdBTEX2		Y	Y	24186	5.2207	CY	UK	O
A0A1D3K7N6	Hypothetical membrane protein OS=Pseudomonas veronii 1YdBTEX2		Y		8460	9.1069	IM	IM	M
A0A1D3K7P1	Uncharacterized protein OS=Pseudomonas veronii 1YdBTEX2	Y			11486.82	8.84	PR	UK	J
A0A1D3K7S1	AraC family transcriptional regulator OS=Pseudomonas veronii 1YdBTEX2			Y	21295	9.8979	CY	CY	K
A0A1D3K7T3	Helicase OS=Pseudomonas veronii 1YdBTEX2	Y			36795.03	9.8	CY	UK	L
A0A1D3K7Z3	Starvation protein A OS=Pseudomonas veronii 1YdBTEX2	Y	Y		21635.01	6.2	UK	CY	O
A0A1D3K820	Uncharacterized protein OS=Pseudomonas veronii 1YdBTEX2	Y			13768.92	6.21	CY	UK	S
A0A1D3K825	Uncharacterized protein OS=Pseudomonas veronii 1YdBTEX2			Y	6552	3.5977	UK	UK	X
A0A1D3K830	Uncharacterized protein OS=Pseudomonas veronii 1YdBTEX2			Y	27756	4.2715	CY	UK	X
A0A1D3K842	Uncharacterized protein OS=Pseudomonas veronii 1YdBTEX2		Y		35771	7.2935	CY	CY	K
A0A1D3K887	Uncharacterized protein OS=Pseudomonas veronii 1YdBTEX2	Y	Y	Y	17064.53	6.28	CY	UK	X
A0A1D3K8A2	Transposase OS=Pseudomonas veronii 1YdBTEX2	Y			36904.55	9.75	CY	CY	L
A0A1D3K8B8	Uncharacterized protein OS=Pseudomonas veronii 1YdBTEX2		Y		13999	4.6538	CY	CY	X
A0A1D3K8L4	Uncharacterized protein OS=Pseudomonas veronii 1YdBTEX2	Y			14936.91	4.34	CY	UK	X
A0A1D3K8L6	Hypothetical secreted protein OS=Pseudomonas veronii 1YdBTEX2		Y		5362	6.1772	UK	IM	X

A0A1D3K8Q8	Chemotaxis protein OS=Pseudomonas veronii 1YdBTEX2			Y	34682	4.6626	UK	ML (CY, IM)	NT
A0A1D3K8S2	Methyl-accepting chemotaxis protein OS=Pseudomonas veronii 1YdBTEX2	Y			28643	4.4443	UK	ML (C, IM)	NT
A0A1D3K8V4	Transposase OS=Pseudomonas veronii 1YdBTEX2	Y			41498.22	9.73	PR	UK	KL
A0A1D3K906	CatO2ase OS=Pseudomonas veronii 1YdBTEX2	Y		Y	35016	5.5122	CY	CY	E
A0A1D3K908	4-hydroxy-2-oxovalerate aldolase OS=Pseudomonas veronii 1YdBTEX2			Y	37151	5.5459	CY	CY	EQ
A0A1D3K930	Isopropylbenzene dioxygenase_ iron-sulfur protein_ small subunit OS=Pseudomonas veronii 1YdBTEX2		Y	Y	21759	5.7993	CY	CY	Q
A0A1D3K961	Uncharacterized protein OS=Pseudomonas veronii 1YdBTEX2		Y		7550	3.9756	CY	UK	X
A0A1D3K963	Uncharacterized protein OS=Pseudomonas veronii 1YdBTEX2			Y	13499	6.1318	CY	UK	S
A0A1D3K967	Transposase OS=Pseudomonas veronii 1YdBTEX2	Y			115041.3	6.89	CY	CY	L
A0A1D3K973	Acetaldehyde dehydrogenase OS=Pseudomonas veronii 1YdBTEX2			Y	32661	5.4946	UK	UK	Q
A0A1D3K985	Uncharacterized protein OS=Pseudomonas veronii 1YdBTEX2	Y			13829	5.0684	CY	CY	S
A0A1D3K990	Uncharacterized protein OS=Pseudomonas veronii 1YdBTEX2			Y	21096	9.5273	UK	UK	S
A0A1D3K9A2	Integrase OS=Pseudomonas veronii 1YdBTEX2	Y	Y		76864.24	7.96	CY	CY	L
A0A1D3K9A5	Single-stranded DNA-binding protein OS=Pseudomonas veronii 1YdBTEX2		Y		18424	7.1763	CY	CY	L
A0A1D3K9C6	Ferredoxin_ plant-type OS=Pseudomonas veronii 1YdBTEX2			Y	12510	7.7285	CY	CY	C
A0A1D3K9G1	Hydrogenase transcriptional regulatory protein HoxA OS=Pseudomonas veronii 1YdBTEX2	Y	Y	Y	54378.59	6.35	CY	CY	TK
A0A1D3K9G5	Multidrug ABC transporter ATP-binding protein OS=Pseudomonas veronii 1YdBTEX2	Y		Y	11137	4.3652	CY	IM	VE(mx)
A0A1D3K9L9	PLD phosphodiesterase domain-containing protein OS=Pseudomonas veronii 1YdBTEX2			Y	46914	6.6152	CY	UK	LI
A0A1D3K9P9	Porin D OS=Pseudomonas veronii 1YdBTEX2	Y			48747	4.9512	OM	OM	S
A0A1D3K9V8	Probable family 20 transposase OS=Pseudomonas veronii 1YdBTEX2	Y	Y		38487.71	10.37	CY	CY	L

A0A1D3K9Y9	Inner membrane transport permease YhhJ OS=Pseudomonas veronii 1YdBTEX2			Y	41656	5.647	IM	IM	VM(mx)
A0A1D3K9Z4	Uncharacterized oxidoreductase Mb2924c OS=Pseudomonas veronii 1YdBTEX2	Y			66645.04	6.58	CY	CY	C
A0A1D3KA55	Twitching motility protein PilT OS=Pseudomonas veronii 1YdBTEX2	Y			15176	7.3198	CY	UK	G
A0A1D3KA82	Resolvase OS=Pseudomonas veronii 1YdBTEX2	Y			34495.98	9.12	CY	CY	L
A0A1D3KA96	Uncharacterized protein OS=Pseudomonas veronii 1YdBTEX2			Y	13485	8.228	CY	CY	S
A0A1D3KAA8	Hypothetical secreted protein OS=Pseudomonas veronii 1YdBTEX2	Y			10097.72	5.65	OM	UK	S
A0A1D3KAD4	Uncharacterized protein YhiI OS=Pseudomonas veronii 1YdBTEX2	Y			38102.14	5.32	OM	IM	MU
A0A1D3KAD8	InaA protein OS=Pseudomonas veronii 1YdBTEX2	Y			26991.18	9.97	CY	CY	QT(mx)
A0A1D3KAH4	Transposase OS=Pseudomonas veronii 1YdBTEX2	Y		Y	30804.09	7.71	CY	CY	L
A0A1D3KAH6	Sex pilus assembly protein OS=Pseudomonas veronii 1YdBTEX2	Y	Y	Y	24950.81	9.2	CY	UK	S
A0A1D3KAI3	Conjugal transfer protein OS=Pseudomonas veronii 1YdBTEX2	Y			33481.02	6.34	PR	PR	CO
A0A1D3KAI6	ATPase OS=Pseudomonas veronii 1YdBTEX2	Y			38095	6.5186	EX	UK	KTL
A0A1D3KAI8	Transposase OS=Pseudomonas veronii 1YdBTEX2			Y	13916	10.0342	UK	UK	L
A0A1D3KAJ5	Cysteine peptidase OS=Pseudomonas veronii 1YdBTEX2	Y	Y	Y	25674.31	6.22	CY	CY	S
A0A1D3KAP2	Uncharacterized protein OS=Pseudomonas veronii 1YdBTEX2	Y			37771.66	5.55	CY	CY	D

Supplementary Table S5. Annotations of significantly changed (fold>1.8; ANOVA p<0.05; fitted Tukey´s HSD thresholds) proteins of *P. veronii* . Functional distribution: C-Energy production and conversion; D-Cell cycle control, cell division, chromosome partitioning; E-Amino acid metabolism and transport; F-Nucleotide metabolism and transport; H-Coenzyme transport and metabolism; I-Lipid transport and metabolism; K-Transcription; M-Cell wall/membrane/envelop biogenesis; N-Cell motility; O-Post-translational modification, protein turnover, and chaperone functions; P-Inorganic ion transport and metabolism; Q-Secondary metabolites biosynthesis, transport, and

Upregulated

UNIPROT Accession	Protein name	MW [Da]	pI	Localizat SOSUIGr amN	PSORTb	Function COGS
A0A1D3JPP4	Short-chain dehydrogenase OS=Pseudomonas veronii 1YdBTEX2	26581	6.4263	CY	CY	IQ
A0A1D3JPQ1	Cytochrome P450 OS=Pseudomonas veronii 1YdBTEX2	44493	5.6118	CY	CY	CQ
A0A1D3JPT9	Transporter OS=Pseudomonas veronii 1YdBTEX2	55429.58	6.3	PR	UK	MU
A0A1D3JQ11	Butanediol dehydrogenase OS=Pseudomonas veronii 1YdBTEX2	38566	5.6162	UK	CY	CE(mx)
A0A1D3JQ27	Cystine-binding periplasmic protein OS=Pseudomonas veronii 1YdBTEX2	28611	9.271	CY	PR	ET
A0A1D3JQ78	Sulfate/thiosulfate import ATP-binding protein CysA OS=Pseudomonas veronii 1YdBTEX2	36633.05	6.1	CY	IM	PQ(mx)
A0A1D3JQB2	Porin D OS=Pseudomonas veronii 1YdBTEX2	46511	6.1582	OM	OM	S
A0A1D3JQC6	TetR family transcriptional regulator OS=Pseudomonas veronii 1YdBTEX2	20041	5.2046	CY	CY	K
A0A1D3JRB4	Porin OS=Pseudomonas veronii 1YdBTEX2	48295	6.1582	EX	OM	S
A0A1D3JRE5	Short-chain dehydrogenase OS=Pseudomonas veronii 1YdBTEX2	24432.75	7.3	CY	EX	IQ

A0A1D3JRS4	Periplasmic dipeptide transport protein OS=Pseudomonas veronii 1YdBTEX2	58644	6.1831	PR	PR	E
A0A1D3JRW1	Porin OS=Pseudomonas veronii 1YdBTEX2	50660	6.1758	OM	OM	S
A0A1D3JS15	Uncharacterized protein OS=Pseudomonas veronii 1YdBTEX2	66959	5.2368	EX	OM	X
A0A1D3JSC3	Fumarylacetoacetase OS=Pseudomonas veronii 1YdBTEX2	46311	5.1489	UK	CY	Q
A0A1D3JSV4	Ferredoxin--NADP reductase OS=Pseudomonas veronii 1YdBTEX2	29756	5.9092	CY	CY	C
A0A1D3JSW6	Glutamate/aspartate periplasmic-binding protein OS=Pseudomonas veronii 1YdBTEX2	33370	8.5986	PR	PR	ET
A0A1D3JSX5	Bacterioferritin OS=Pseudomonas veronii 1YdBTEX2	17901	4.6963	CY	CY	P
A0A1D3JT07	Adenosylmethionine-8-amino-7-oxononanoate aminotransferase OS=Pseudomonas veronii 1YdBTEX2	52137.2	5.77	CY	CY	EH
A0A1D3JT64	Lysine/arginine/ornithine-binding periplasmic protein OS=Pseudomonas veronii 1YdBTEX2	27426	6.189	PR	PR	ET
A0A1D3JT88	Membrane protein OS=Pseudomonas veronii 1YdBTEX2	15847	9.4834	PR	OM	MI
A0A1D3JT92	Leucine-_ isoleucine-_ valine-_ threonine-_ and alanine-binding protein OS=Pseudomonas veronii 1YdBTEX2	39740	6.4468	PR	PR	E
A0A1D3JTA7	Protocatechuate 3_4-dioxygenase subunit alpha OS=Pseudomonas veronii 1YdBTEX2	20539	4.8369	CY	CY	Q
A0A1D3JTJ5	Toluene efflux pump periplasmic linker protein TtgA OS=Pseudomonas veronii 1YdBTEX2	38820.31	6.75	PR	IM	MVP
A0A1D3JU31	CSD domain-containing protein OS=Pseudomonas veronii 1YdBTEX2	7782	7.6494	CY	CY	KL
A0A1D3JUF4	Hypothetical secreted protein OS=Pseudomonas veronii 1YdBTEX2	8117	5.0054	PR	UK	M
A0A1D3JUR8	Translational regulator CsrA OS=Pseudomonas veronii 1YdBTEX2	6962.78	6.53	UK	UK	T

A0A1D3JV13	Uncharacterized protein OS=Pseudomonas veronii 1YdBTEX2	63142.31	4.93	CY	UK	S
A0A1D3JVA1	Uncharacterized protein OS=Pseudomonas veronii 1YdBTEX2	23214	4.9878	OM	EX	FT
A0A1D3JVA8	Membrane protein OS=Pseudomonas veronii 1YdBTEX2	42373	4.6743	OM	OM	M
A0A1D3JVB3	Amino acid ABC transporter substrate-binding protein OS=Pseudomonas veronii 1YdBTEX2	32896	4.7988	PR	PR	ET
A0A1D3JVC5	Membrane protein OS=Pseudomonas veronii 1YdBTEX2	34907	5.7832	OM	UK	DZ(mx)
A0A1D3JVD0	Cytochrome C OS=Pseudomonas veronii 1YdBTEX2	16392	5.1475	PR	UK	CT
A0A1D3JVD1	Quinoprotein ethanol dehydrogenase OS=Pseudomonas veronii 1YdBTEX2	69501	6.7207	OM	PR	CH
A0A1D3JVD3	Aldehyde dehydrogenase OS=Pseudomonas veronii 1YdBTEX2	55001	5.3672	CY	CY	CK
A0A1D3JVK4	Quinoprotein ethanol dehydrogenase OS=Pseudomonas veronii 1YdBTEX2	64522	8.0405	PR	PR	GC
A0A1D3JVQ9	Branched-chain amino acid ABC transporter substrate-binding protein OS=Pseudomonas veronii 1YdBTEX2	43111	5.9839	PR	UK	E
A0A1D3JW46	ABC transporter substrate-binding protein OS=Pseudomonas veronii 1YdBTEX2	64816	5.8125	EX	OM	EP
A0A1D3JWQ8	Uncharacterized protein OS=Pseudomonas veronii 1YdBTEX2	35432.99	5.54	PR	UK	FG
A0A1D3JWV4	Nitrate reductase (quinone) OS=Pseudomonas veronii 1YdBTEX2	140747	6.104	CY	ML (C, IM)	C
A0A1D3JX42	von Willebrand factor A OS=Pseudomonas veronii 1YdBTEX2	40132.58	8.85	IM	IM	MH(mx)
A0A1D3JXF4	TonB-dependent receptor OS=Pseudomonas veronii 1YdBTEX2	78594	4.8794	OM	OM	PH
A0A1D3JXG1	Biotin carboxylase OS=Pseudomonas veronii 1YdBTEX2	61095	5.5371	UK	CY	IC

A0A1D3JXL3	Uncharacterized protein OS=Pseudomonas veronii 1YdBTEX2	12383	9.3237	PR	UK	M
A0A1D3JY28	Uncharacterized protein OS=Pseudomonas veronii 1YdBTEX2	36406	6.3911	OM	UK	C
A0A1D3JY68	Esterase OS=Pseudomonas veronii 1YdBTEX2	38026	5.2178	EX	CY	CI
A0A1D3JY75	Hypothetical secreted protein OS=Pseudomonas veronii 1YdBTEX2	8954	9.8804	PR	OM	S
A0A1D3JYA8	Hypothetical secreted protein OS=Pseudomonas veronii 1YdBTEX2	9010	7.3184	PR	OM	S
A0A1D3JYZ2	Uncharacterized protein OS=Pseudomonas veronii 1YdBTEX2	13950.67	4.53	EX	UK	X
A0A1D3JZ23	Sugar ABC transporter substrate-binding protein OS=Pseudomonas veronii 1YdBTEX2	48187	6.5244	PR	UK	G
A0A1D3JZA0	TonB-denpendent receptor OS=Pseudomonas veronii 1YdBTEX2	82573	5.1841	OM	OM	PH
A0A1D3JZE2	Ferripyoverdine receptor OS=Pseudomonas veronii 1YdBTEX2	88987	5.0303	OM	OM	PH
A0A1D3K049	L-arabinose-binding periplasmic protein OS=Pseudomonas veronii 1YdBTEX2	35506	7.1763	PR	PR	GK
A0A1D3K079	Virulence factors putative positive transcription regulator BvgA OS=Pseudomonas veronii 1YdBTEX2	23017.24	8.86	CY	CY	KT
A0A1D3K0Q4	ABC transporter substrate-binding protein OS=Pseudomonas veronii 1YdBTEX2	38151	5.9868	PR	PR	E
A0A1D3K0Q9	Hydroxycinnamoyl-CoA hydratase-lyase OS=Pseudomonas veronii 1YdBTEX2	31057.77	5.66	CY	CY	IH
A0A1D3K181	Isochorismatase OS=Pseudomonas veronii 1YdBTEX2	20683	6.4131	CY	CY	Q
A0A1D3K1B1	Chemotaxis protein OS=Pseudomonas veronii 1YdBTEX2	46935.2	4.99	IM	IM	NT
A0A1D3K1U5	Membrane protein OS=Pseudomonas veronii 1YdBTEX2	77979	5.0684	EX	OM	PH

A0A1D3K1V3	Cbb3-type cytochrome c oxidase subunit OS=Pseudomonas veronii 1YdBTEX2	35294	6.0688	IM	IM	C
A0A1D3K1V5	Extradiol dioxygenase OS=Pseudomonas veronii 1YdBTEX2	13312	4.83	CY	UK	E
A0A1D3K1V7	Peptidase S41 OS=Pseudomonas veronii 1YdBTEX2	22441	8.666	CY	UK	C
A0A1D3K1Y8	Cell division protein ZipA OS=Pseudomonas veronii 1YdBTEX2	31651	5.3467	IM	CY	X
A0A1D3K251	Amidotransferase OS=Pseudomonas veronii 1YdBTEX2	27763	5.2119	CY	CY	F
A0A1D3K2I7	Lysine/arginine/ornithine-binding periplasmic protein OS=Pseudomonas veronii 1YdBTEX2	28347	7.0386	PR	PR	ET
A0A1D3K2T8	CSD domain-containing protein OS=Pseudomonas veronii 1YdBTEX2	7788	7.708	CY	CY	KL
A0A1D3K2Y0	Methyl-accepting chemotaxis protein OS=Pseudomonas veronii 1YdBTEX2	76074	4.8486	IM	IM	NT(mx)
A0A1D3K2Y2	Peptidase OS=Pseudomonas veronii 1YdBTEX2	47150	4.3711	UK	OM	P
A0A1D3K342	Porin D OS=Pseudomonas veronii 1YdBTEX2	46960	5.6396	OM	OM	S
A0A1D3K345	Secretion protein HylD OS=Pseudomonas veronii 1YdBTEX2	38015	8.9209	IM	IM	MV
A0A1D3K353	Threonine synthase OS=Pseudomonas veronii 1YdBTEX2	51952	5.7393	CY	CY	EP
A0A1D3K3A4	TonB-dependent receptor OS=Pseudomonas veronii 1YdBTEX2	92999	6.4937	EX	OM	PH
A0A1D3K3P9	Transcription termination/antitermination protein NusA OS=Pseudomonas veronii 1YdBTEX2	54381	4.3506	CY	CY	KJ
A0A1D3K3R6	Catechol 1_2-dioxygenase OS=Pseudomonas veronii 1YdBTEX2	34546	4.8457	CY	CY	Q
A0A1D3K3X1	Acetone carboxylase gamma subunit OS=Pseudomonas veronii 1YdBTEX2	19482	5.3862	CY	CY	EQ

A0A1D3K402	Acetone carboxylase alpha subunit OS=Pseudomonas veronii 1YdBTEX2	86396	5.4595	CY	CY	EQ
A0A1D3K451	Acetone carboxylase beta subunit OS=Pseudomonas veronii 1YdBTEX2	77668	5.0962	CY	CY	EQ
A0A1D3K4A8	AMP nucleosidase OS=Pseudomonas veronii 1YdBTEX2	54477	6.3633	CY	CY	F
A0A1D3K4H5	6_7-dimethyl-8-ribityllumazine synthase OS=Pseudomonas veronii 1YdBTEX2	16437	5.6177	CY	CY	H
A0A1D3K4M5	30S ribosomal protein S7 OS=Pseudomonas veronii 1YdBTEX2	17636	10.5762	CY	CY	J
A0A1D3K4P2	50S ribosomal protein L24 OS=Pseudomonas veronii 1YdBTEX2	11338	10.5615	CY	CY	J
A0A1D3K4U2	Chaperone SurA OS=Pseudomonas veronii 1YdBTEX2	49411	5.3818	CY	ML (C, P)	MO
A0A1D3K4U4	Coenzyme PQQ synthesis protein B OS=Pseudomonas veronii 1YdBTEX2	33073	4.9761	UK	CY	P
A0A1D3K4X6	TonB-denpendent receptor OS=Pseudomonas veronii 1YdBTEX2	60843	5.3994	OM	OM	PH
A0A1D3K549	Sarcosine oxidase subunit beta OS=Pseudomonas veronii 1YdBTEX2	45155.73	8.2	UK	CY	EH(mx)
A0A1D3K558	RNA polymerase sigma factor RpoD OS=Pseudomonas veronii 1YdBTEX2	69291	4.8516	CY	CY	K
A0A1D3K5B8	Aldehyde dehydrogenase OS=Pseudomonas veronii 1YdBTEX2	52119	8.8096	IM	CY	C
A0A1D3K5F0	RNA pyrophosphohydrolase OS=Pseudomonas veronii 1YdBTEX2	18868.82	9.3	CY	CY	LF
A0A1D3K5L2	Anaerobically-induced outer membrane porin OprE OS=Pseudomonas veronii 1YdBTEX2	47187	5.6455	OM	OM	S
A0A1D3K5L4	Transporter OS=Pseudomonas veronii 1YdBTEX2	34637	7.6758	OM	IM	MVI
A0A1D3K5R1	Cell division protein ZapA OS=Pseudomonas veronii 1YdBTEX2	11579.9	5.64	CY	UK	D

A0A1D3K5S1	sn-glycerol-3-phosphate transporter OS=Pseudomonas veronii 1YdBTEX2	17731	9.5112	EX	UK	S
A0A1D3K5Y5	Chemotaxis protein OS=Pseudomonas veronii 1YdBTEX2	70053	5.1577	IM	IM	NT(mx)
A0A1D3K6A7	D-amino acid dehydrogenase OS=Pseudomonas veronii 1YdBTEX2	47149.32	8.18	PR	ML (C a IM)	EH(mx)
A0A1D3K6N0	Bifunctional protein GlmU OS=Pseudomonas veronii 1YdBTEX2	48747.57	6.4	CY	CY	JM
A0A1D3K7I1	Fe2OG dioxygenase domain-containing protein OS=Pseudomonas veronii 1YdBTEX2	24186	5.2207	CY	UK	O
A0A1D3K906	CatO2ase OS=Pseudomonas veronii 1YdBTEX2	35016	5.5122	CY	CY	E
A0A1D3K9G1	Hydrogenase transcriptional regulatory protein HoxA OS=Pseudomonas veronii 1YdBTEX2	54378.59	6.35	CY	CY	TK
**Downregulated**						
**UNIPROT Accession**	**Protein name**	**MW [Da]**	**pI**	**Localization SOSUIGramN**	**PSORTb**	**Function COGS**
A0A1D3JPL2	DNA gyrase subunit B OS=Pseudomonas veronii 1YdBTEX2	90406.19	5.52	CY	CY	L
A0A1D3JQH4	Methyl-accepting chemotaxis protein McpS OS=Pseudomonas veronii 1YdBTEX2	68360	4.8105	IM	IM	NT(mx)

A0A1D3JQM4	Cobyric acid synthase OS=Pseudomonas veronii 1YdBTEX2	28585	4.938	UK	CY	D
A0A1D3JQN4	Protein-export protein SecB OS=Pseudomonas veronii 1YdBTEX2	17600	4.2979	CY	CY	U
A0A1D3JR37	Methyl-accepting chemotaxis protein CtpL OS=Pseudomonas veronii 1YdBTEX2	69417	4.9614	IM	OM	NT(mx)
A0A1D3JR94	Uncharacterized HTH-type transcriptional regulator PA4778 OS=Pseudomonas veronii 1YdBTEX2	15095	7.2583	CY	CY	KT
A0A1D3JRP9	Catabolite repressor/activator OS=Pseudomonas veronii 1YdBTEX2	36325	6.75	UK	CY	KG
A0A1D3JRT9	Sulfate adenylyltransferase subunit 2 OS=Pseudomonas veronii 1YdBTEX2	35198	5.9458	CY	CY	EH
A0A1D3JS27	UDP-N-acetylmuramoylalanine--D-glutamate ligase OS=Pseudomonas veronii 1YdBTEX2	47515.42	5.64	CY	CY	MHJ
A0A1D3JS51	D-alanine--D-alanine ligase OS=Pseudomonas veronii 1YdBTEX2	34282	4.6377	CY	CY	FI
A0A1D3JS54	Cell division protein FtsA OS=Pseudomonas veronii 1YdBTEX2	44848	4.9761	CY	CY	DN(mx)
A0A1D3JSG1	UDP-N-acetylmuramoyl-tripeptide--D-alanyl-D-alanine ligase OS=Pseudomonas veronii 1YdBTEX2	47310	6.5405	CY	CY	MHJ
A0A1D3JUJ8	Methyl-accepting chemotaxis protein OS=Pseudomonas veronii 1YdBTEX2	73016.82	5.46	IM	ML (IM, OM)	NT
A0A1D3JV34	3-isopropylmalate dehydratase large subunit OS=Pseudomonas veronii 1YdBTEX2	50696	5.5444	CY	CY	EC
A0A1D3JVI5	Branched-chain-amino-acid aminotransferase OS=Pseudomonas veronii 1YdBTEX2	36830	5.8887	CY	CY	E
A0A1D3JVU1	Major tail tube protein OS=Pseudomonas veronii 1YdBTEX2	18080.1	5.49	CY	UK	S
A0A1D3JW75	Disulfide-bond oxidoreductase YghU OS=Pseudomonas veronii 1YdBTEX2	31018.84	5.9	CY	CY	OH
A0A1D3JW89	Aldehyde dehydrogenase OS=Pseudomonas veronii 1YdBTEX2	59606	6.1816	CY	CY	CE

A0A1D3JWI4	Methyl-accepting chemotaxis protein OS=Pseudomonas veronii 1YdBTEX2	57991	4.9131	IM	IM	NT(mx)
A0A1D3JXE2	Glycogen operon protein GlgX homolog OS=Pseudomonas veronii 1YdBTEX2	81728	5.1533	CY	CY	G
A0A1D3JXK1	Methylhydantoinase OS=Pseudomonas veronii 1YdBTEX2	59189	4.8721	EX	CY	EQ
A0A1D3JXN6	4-hydroxyphenylpyruvate dioxygenase OS=Pseudomonas veronii 1YdBTEX2	40163	4.8076	CY	CY	E
A0A1D3JXS0	Probable acetyl-CoA acetyltransferase OS=Pseudomonas veronii 1YdBTEX2	40823	6.394	CY	CY	I
A0A1D3JXT7	Acyl-CoA dehydrogenase OS=Pseudomonas veronii 1YdBTEX2	40594	5.0845	CY	CY	I
A0A1D3JXY5	Aldehyde-activating protein OS=Pseudomonas veronii 1YdBTEX2	16947	6.0425	UK	PR	S
A0A1D3JY52	Acetylornithine aminotransferase OS=Pseudomonas veronii 1YdBTEX2	44912	6.3208	CY	CY	EH
A0A1D3JY66	Probable enoyl-CoA hydratase OS=Pseudomonas veronii 1YdBTEX2	27758	5.2939	CY	CY	IH(mx)
A0A1D3JY92	Alcohol dehydrogenase OS=Pseudomonas veronii 1YdBTEX2	37431	5.3042	CY	CY	CP(mx)
A0A1D3JYB7	Universal stress protein OS=Pseudomonas veronii 1YdBTEX2	33891	5.6001	UK	CY	T
A0A1D3JZZ6	Alcohol dehydrogenase_ propanol-preferring OS=Pseudomonas veronii 1YdBTEX2	35559	5.981	UK	CY	CP
A0A1D3K076	Lysine/arginine/ornithine-binding periplasmic protein OS=Pseudomonas veronii 1YdBTEX2	28213	7.9233	PR	PR	ET
A0A1D3K167	Peptidase M42 OS=Pseudomonas veronii 1YdBTEX2	42559	4.9805	CY	CY	GE
A0A1D3K184	Succinate--CoA ligase [ADP-forming] subunit beta OS=Pseudomonas veronii 1YdBTEX2	42946	5.9312	CY	CY	CF
A0A1D3K1P0	Dehydrogenase OS=Pseudomonas veronii 1YdBTEX2	26143	9.397	PR	PR	S

A0A1D3K1Q3	Cysteine synthase OS=Pseudomonas veronii 1YdBTEX2	32416	5.0361	CY	CY	E
A0A1D3K2B2	4-hydroxy-4-methyl-2-oxoglutarate aldolase OS=Pseudomonas veronii 1YdBTEX2	17408	4.4604	CY	CY	H
A0A1D3K2G3	Arginine N-succinyltransferase subunit beta OS=Pseudomonas veronii 1YdBTEX2	37416	6.271	CY	CY	E
A0A1D3K2X0	UPF0234 protein PVE_R1G4785 OS=Pseudomonas veronii 1YdBTEX2	18236	7.3169	CY	CY	S
A0A1D3K314	Glyceraldehyde-3-phosphate dehydrogenase OS=Pseudomonas veronii 1YdBTEX2	36056	6.23	CY	CY	GE
A0A1D3K332	Arginine deiminase OS=Pseudomonas veronii 1YdBTEX2	46343	5.2427	CY	CY	E
A0A1D3K371	Iron-sulfur cluster assembly scaffold protein IscU OS=Pseudomonas veronii 1YdBTEX2	13861	5.0186	CY	CY	CP
A0A1D3K3D5	30S ribosomal protein S16 OS=Pseudomonas veronii 1YdBTEX2	9201	10.8369	CY	CY	J
A0A1D3K3I7	Iron-binding protein IscA OS=Pseudomonas veronii 1YdBTEX2	11648	4.6743	CY	CY	C
A0A1D3K3K8	Glutathione peroxidase OS=Pseudomonas veronii 1YdBTEX2	17519	5.2383	CY	PR	CO
A0A1D3K3M6	RNA polymerase-binding transcription factor DksA OS=Pseudomonas veronii 1YdBTEX2	17100	4.9497	CY	CY	T
A0A1D3K3P0	Anthranilate 1_2-dioxygenase small subunit OS=Pseudomonas veronii 1YdBTEX2	19536	6.8965	CY	CY	Q
A0A1D3K3V4	Phosphoglucosamine mutase OS=Pseudomonas veronii 1YdBTEX2	47719	5.5254	CY	CY	G
A0A1D3K4M9	50S ribosomal protein L15 OS=Pseudomonas veronii 1YdBTEX2	15215	11.2471	CY	CY	J
A0A1D3K4N8	30S ribosomal protein S17 OS=Pseudomonas veronii 1YdBTEX2	10090	10.1455	CY	CY	J
A0A1D3K4Q4	Anthranilate synthase component 1 OS=Pseudomonas veronii 1YdBTEX2	54351	4.8413	UK	CY	EH

A0A1D3K4R6	50S ribosomal protein L23 OS=Pseudomonas veronii 1YdBTEX2	10938	10.5088	CY	CY	J
A0A1D3K4R8	UPF0229 protein PVE_R1G5476 OS=Pseudomonas veronii 1YdBTEX2	48607	6.4761	CY	CY	S
A0A1D3K563	Rhamnosyltransferase 1 subunit A OS=Pseudomonas veronii 1YdBTEX2	33028	6.4922	CY	CY	IC
A0A1D3K569	Glutathione-independent formaldehyde dehydrogenase OS=Pseudomonas veronii 1YdBTEX2	42186	6.1626	CY	CY	CE(mx)
A0A1D3K596	Aspartate carbamoyltransferase OS=Pseudomonas veronii 1YdBTEX2	36358.97	7.14	CY	CY	EF
A0A1D3K5A9	Thioesterase OS=Pseudomonas veronii 1YdBTEX2	15057	7.314	CY	CY	IQ
A0A1D3K5D8	4-deoxy-4-formamido-L-arabinose-phosphoundecaprenol deformylase ArnD OS=Pseudomonas veronii 1YdBTEX2	79551	6.2769	IM	IM	PC
A0A1D3K5I5	Fructose-1_6-bisphosphate aldolase OS=Pseudomonas veronii 1YdBTEX2	38385	5.291	PR	CY	G
A0A1D3K5S6	Uncharacterized protein OS=Pseudomonas veronii 1YdBTEX2	34757	11.0405	CY	UK	S
A0A1D3K5U2	Spermidine/putrescine import ATP-binding protein PotA OS=Pseudomonas veronii 1YdBTEX2	42374	5.9355	CY	IM	PE(mx)
A0A1D3K616	Biopolymer transport protein ExbB OS=Pseudomonas veronii 1YdBTEX2	29676.73	6.6	IM	IM	U
A0A1D3K679	Type VI secretion protein ImpA OS=Pseudomonas veronii 1YdBTEX2	36751.68	4.56	CY	UK	M
A0A1D3K6F2	Chemotaxis protein CheY OS=Pseudomonas veronii 1YdBTEX2	32512.18	8.95	CY	CY	TK
A0A1D3K7B5	Uncharacterized protein OS=Pseudomonas veronii 1YdBTEX2	62364.94	5.61	CY	CY	CI
A0A1D3K9A2	Integrase OS=Pseudomonas veronii 1YdBTEX2	76864.24	7.96	CY	CY	L

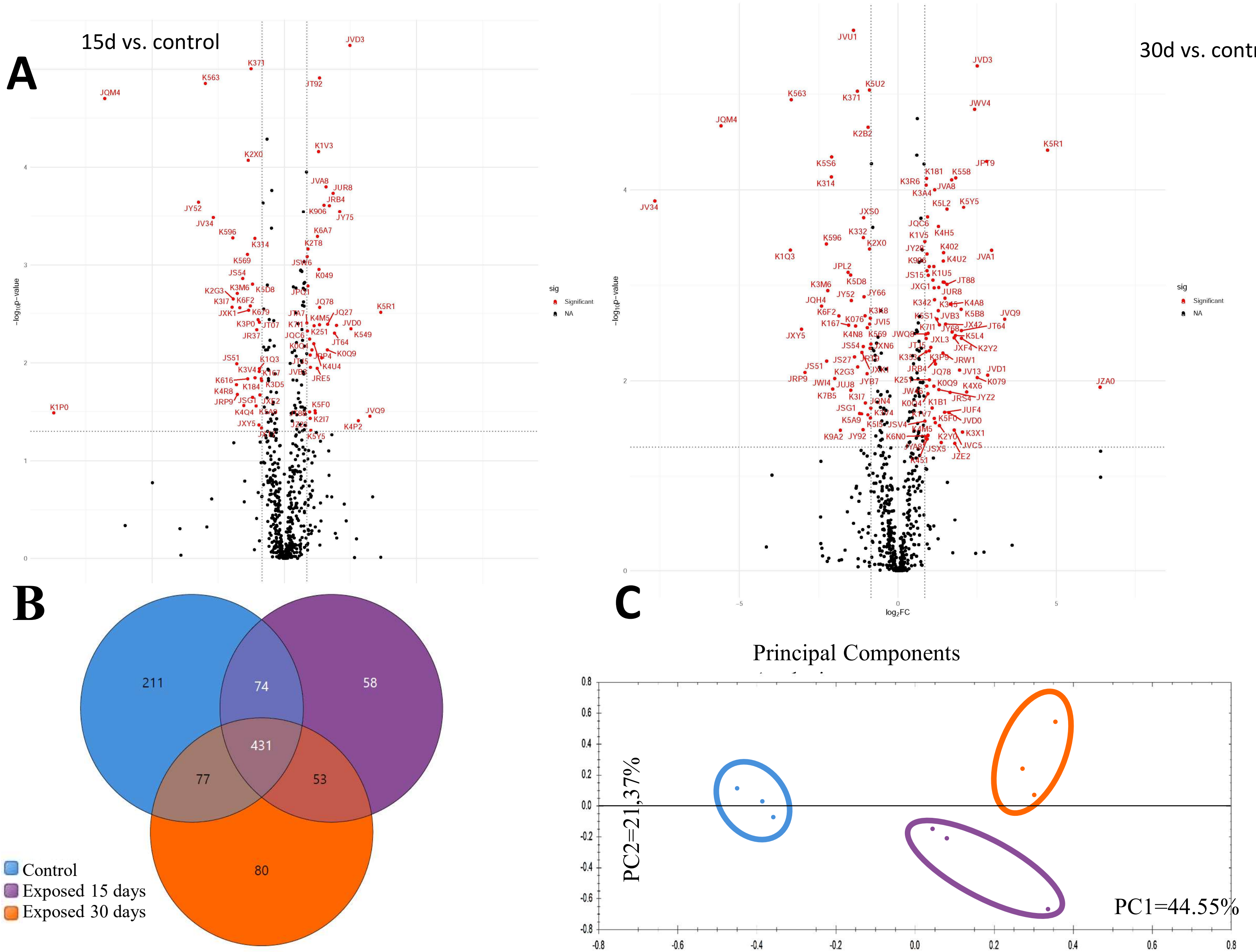

Supplementary Figure S10. Distribution of identified proteins of *P. veronii* among sampling points. ***A***. *Volcano plots of proteins identified in exposed samples (after 15 and 30 days) versus control (cells before PHE exposure).* **B**. *Venn diagram of the number of identified proteins in exposed P. veronii SM-20 compared to control. Overlapping regions indicate the number of proteins that are shared among the different samples under analysis.* **C.** *Principal component analysis, calculated and visualized using the functions integrated into Progenesis QI, confirmed clustering of biological replicates into representative groups.*

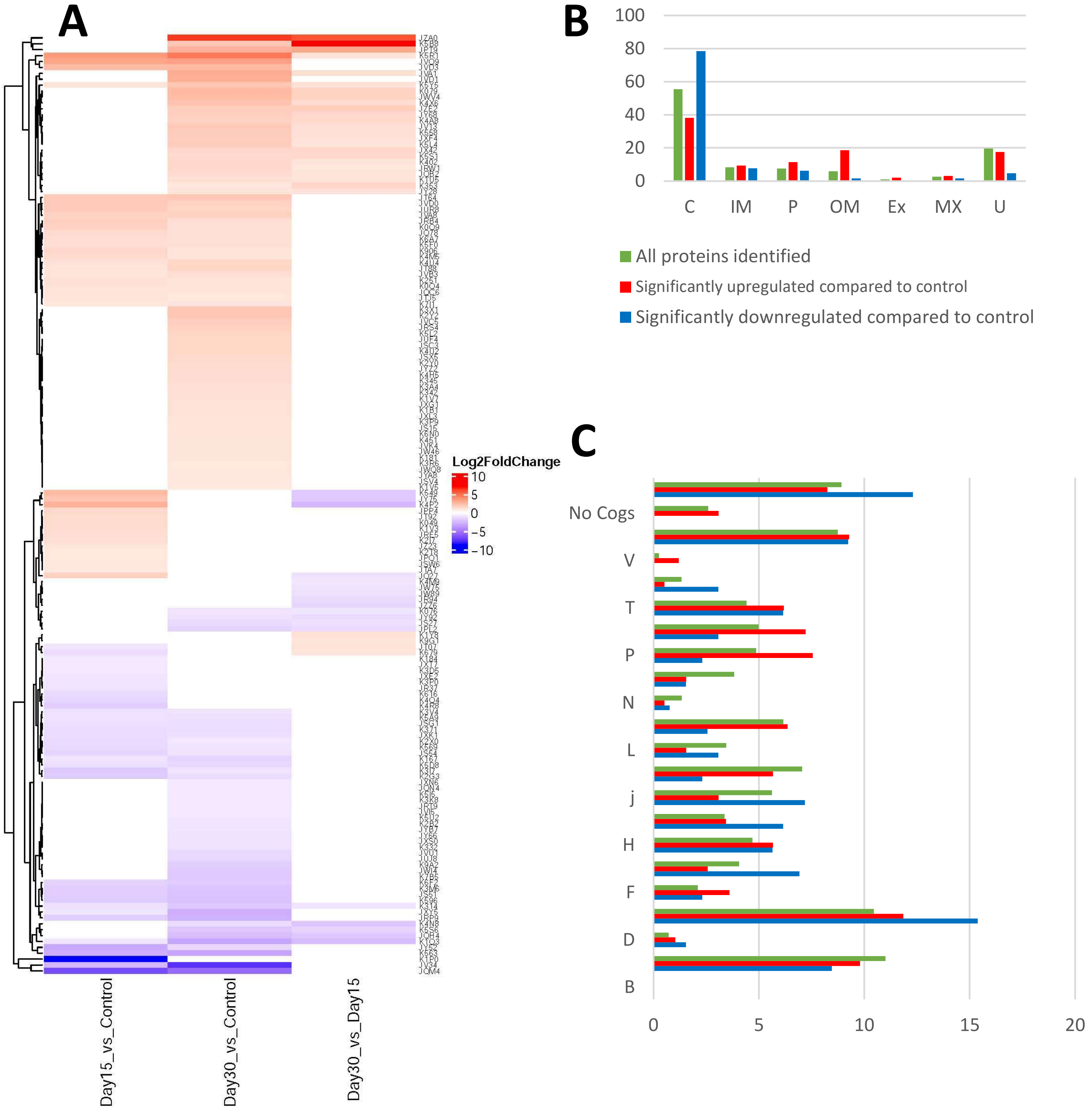

Supplementary Figure S11. Heat map **(A)** of statistically significant upregulated (red) and downregulated (blue) proteins of *P. veronii* and their classification based on subcellular localization **(B)** and clusters of orthologous groups (COGs) **(C)** in contrast to all identified (green) proteins. *Subcellular localization (in percentages) according to PSORTb prediction tool (C-Cytoplasmic; IM-Inner membrane; P-Periplasmic; OM-Outer membrane; Ex-Extracellular; MX-Multiple Localization U-Unknown localization). Functional distribution (in percentages) by the EggNOG v5.0 database (B-Chromatin Structure and dynamics; C-Energy production and conversion; D-Cell cycle control, cell division, chromosome partitioning; E-Amino acid metabolism and transport; F-Nucleotide metabolism and transport; G-Carbohydrate metabolism and transport; H-Coenzyme transport and metabolism; I-Lipid transport and metabolism; J-Translation, ribosomal structures, and biogenesis; K-Transcription; L-Replication, recombination, and repair; M-Cell wall/membrane/envelop biogenesis; N-Cell motility; O-Post-translational modification, protein turnover, and chaperone functions; P-Inorganic ion transport and metabolism; Q-Secondary metabolites biosynthesis, transport, and catabolism; T-Signal transduction mechanisms; U-Intracellular trafficking, secretion, and vesicular transport; V-Defense mechanisms; S-Function unknown; MX-Multiple functions (more than 3).*
